# Bio-inspired iron-catalyzed oxidation of alkylarenes enables late-stage oxidation of complex methylarenes to arylaldehydes

**DOI:** 10.1038/s41467-019-10414-7

**Published:** 2019-06-03

**Authors:** Penghui Hu, Mingxi Tan, Lu Cheng, Hongyuan Zhao, Rui Feng, Wei-Jin Gu, Wei Han

**Affiliations:** 1 Jiangsu Collaborative Innovation Center of Biomedical Functional Materials, Jiangsu Key Laboratory of Biofunctional Materials, Key Laboratory of Applied Photochemistry, Nanjing Normal University, Wenyuan Road No.1, 210023 Nanjing, China; 2 School of Chemistry and Materials Science, Nanjing Normal University, Wenyuan Road No.1, 210023 Nanjing, China

**Keywords:** Homogeneous catalysis, Reaction mechanisms, Synthetic chemistry methodology

## Abstract

It is a long-standing challenge to achieve efficient and highly selective aerobic oxidation of methylarenes to benzaldehydes, owing to overoxidation problem stemming from the oxidizability of benzaldehyde far higher than the toluene under usual aerobic conditions. Herein we report a bio-inspired iron-catalyzed polymethylhydrosiloxane-promoted aerobic oxidation of methylarenes to benzaldehydes with high yields and selectivities. Notably, this method can tolerate oxidation-labile and reactive boronic acid group, which is normally required to be transformed immediately after its introduction, and represents a significant advance in the area of the chemistry of organoboronic acids, including the ability to incorporate both aldehyde and ketone functionalities into unprotected arylboronic acids, a class that can be difficult to access by current means. The robustness of this protocol is demonstrated on the late-stage oxidation of complex bioactive molecules, including dehydroabietic acid, Gemfibrozil, Tocopherol nicotinate, a complex polyol structure, and structurally complex arylboronic acids.

## Abstract (web summary)

Oxidation of toluenes to benzaldehydes is usually accompanied by overoxidation products. Here, the authors report an iron-catalysed aerobic oxidation of methylarenes to benzaldehydes with high yields and selectivities, even in presence of boronic acid groups and in complex natural products and drugs.

## Introduction

Selective oxygenation of sp^3^ C–H bonds to carbonyls is one of the most fundamental transformations for its wide applications in the manufacture of fine chemicals and pharmaceuticals^1–3^. An important subclass of these reactions is the oxygenation of alkyl aromatics that are among the most inexpensive and readily available raw materials. For instance, toluene, the simplest and the most readily available alkylarene, can be oxidized to benzaldehyde, a commercially significant as a versatile intermediate in organic synthesis. However, a selective, high yield of this transformation remains a considerable challenge owing to overoxidation stemming from the oxidizability of benzaldehyde far higher than the toluene under usual aerobic conditions^4^. Currently, the main industrial process for the production of benzaldehyde is liquid phase chlorination of toluene followed by saponification^5^. Obviously, this process produce large quantities of halide waste. Single step effective generation of ArCHO from ArCH_3_ using stoichiometric reagents, such as heteropolyacids^6^, *o*-iodoxybenzoic acid (IBX)^7^, chromyl chloride^8^, cerium oxidants^9^, pyridinium chlorochromate (PCC)^10^, and manganese oxidants^11^ has been reported. However, the use of stoichiometric amounts of reagents makes these processes environmentally unfriendly.

Attempts to overcome these problems have prompted investigation of the use of catalytic oxidation systems for the reactions and to date these have used photocatalytic oxygenation^12^, or transition-metal complexes catalysts^13^, but all of these show low activity and/or selectivity and a quite narrow scope of methylarenes. More recently, Pappo and co-workers reported an elegant study on the direct oxidation of methylarenes to benzaldehydes in high selectivities and yields using cobalt catalysis^14^. However, the catalytic system is probed only on simple methylarenes. Single step highly selective catalytic formation of ArCHO from beyond simple ArCH_3_, like methylheteroarenes and complex molecules bearing primary benzylic C–H bonds has not been demonstrated, but could have significant impact on organic synthesis^15^.

Iron is an ideal candidate for economical and environmental benign catalysis because of its abundant availability and nontoxicity^16–19^. Although iron-based heme and non-heme catalysts have been the most studied compounds for oxygenation of *sp*^3^ C–H^13,20–22^, development of effective iron catalysis for oxidation of methylaromatics to arylaldehydes that have greatly improved activity while retaining high selectivity remains a long-standing goal^23–38^. Herein, we report a highly efficient iron-catalyzed aerobic oxidation of challenging alkylaromatics, methylaromatics in particular. Notably, this method employs inexpensive and nontoxic reagents and enables late-stage oxidation of complex molecules bearing benzylic C–H bonds, which has never been demonstrated for any other catalytic oxidations of alkylaromatics (see below).

**Fig. 1:**
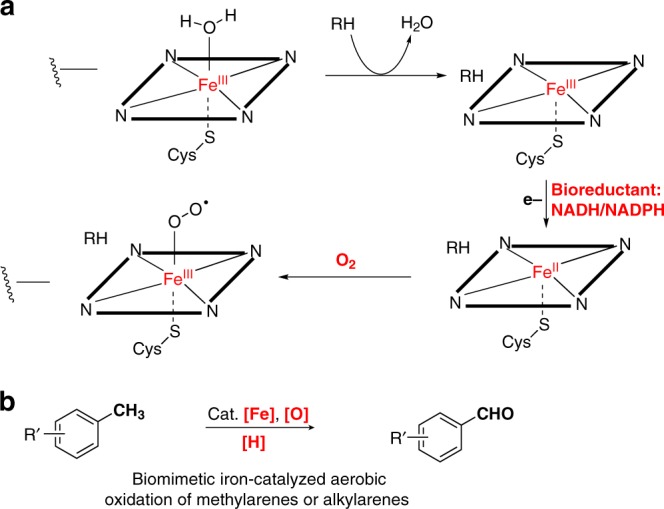
Biocatalysis and biomimetic oxidation of hydrocarbons. **a** Biocatalysis: cytochrome P-450 cycle driven by a reductase or bioreductant. **b** This work: biomimetic iron catalysis

We devise an iron catalysis inspired by cytochrome P-450-catalyzed oxidation of alkanes^21,39^, which involves reductive activation of molecular oxygen on its iron proto-porphyrin IX (hemin) in the presence of a reductase (Fig. 1a). Driven by our interest in iron-catalyzed oxidation reactions^40^, we explore the possibility of direct iron-catalyzed oxidation of methylaromatics to arylaldehydes with high efficiency and broad substrate scope.

## Results

### Optimization of the reaction conditions

Initially, toluene was selected as the benchmark substrate to optimize reaction conditions (Table 1). Essential for the success of the reaction is the use of polymethylhydrosiloxane (PMHS)^41^, which is compatible with oxidative system and can function as an effective turnover reagent, like a reductase in cytochrome P-450-catalyzed oxidations^22,39^. Investigation of a range of parameters showed that the best results are obtained using the combination (inspired by a biocatalytic system using a flavoheme dehydrogenase as catalyst and ferrocene as mediator for oxidation of *p*-alkylphenols into the corresponding aldehydes^42^) of iron(II) phthalocyanine (1 mol%) and ferrocene^43^ (10 mol%) as the catalyst, K_2_S_2_O_8_ (1.0 equiv) (a useful oxidant because of its appreciated properties of low cost, low toxicity, and good stability)^44^ as the oxidant, PMHS as the activator, and CH_3_CN/H_2_O (1:1) as the solvent under ambient air (or 1 atm of O_2_) at 80 °C (entries 1 and 6) (for details, see Supplementary Table 1). Control experiments conducted in the absence of either the iron catalyst, PMHS, or water resulted in little desired product **2a** (entries 2–4). Moreover, in the absence of PMHS the over-oxidation level increased much (entry 3). When the reaction was performed in N_2_, **2a** was not observed (entry 5). The use of Fe(acac)_2_, FeSO_4_·7H_2_O or FeCl_2_ instead of iron(II) phthalocyanine and ferrocene gave a much poorer result (entries 6–9). The yield significantly diminished when other silane such as Et_3_SiH or (EtO)_3_SiH was used (entries 10–11). Changing the oxidant to *m*-CPBA, DTBP, or TBHP led to <5% yield of **2a** (entry 12). Replacing acetonitrile with DCE, 1,4-dioxane, or DMF proved not to be beneficial (for details, see Supplementary Table 1).

**Table 1:** Select screening experiments for iron-catalyzed aerobic oxidation of toluene (**1a**)^a^


Entry	Variations from optimal conditions	Yield (2a/2A) (%)
1	None	86/2
2	No ferrocene and Fe(II)Pc	4/–
3	No PMHS	16/10
4	No H_2_O	4/1
5	N_2_ instead of air	–/–
6	O_2_ instead of air	89/2
7	Fe(acac)_2_ instead of ferrocene and Fe(II)Pc	81/3
8	FeSO_4_.7H_2_O instead of ferrocene and Fe(II)Pc	61/3
9	FeCl_2_ instead of ferrocene and Fe(II)Pc	27/6
10^b^	Et_3_SiH instead of PMHS	35/3
11^b^	(EtO)_3_SiH instead of PMHS	17/2
12^b^	*m*-CPBA, DTBP or TBHP instead of K_2_S_2_O_8_	<5/–

*m*-*CPBA* *m*-chloroperoxybenzoic acid, *DTBP* di-*t*-butyl peroxide, *TBHP* *tert*-butyl hydroperoxide, *Fe(II)Pc* iron(II) phthalocyanine

^a^Yields were determined by ^1^H NMR using chlorobenzene as the internal standard

^b^Fe(acac)_2_ (ferrous acetylacetonate) as the catalyst

### Substrate scope

**Fig. 2:**
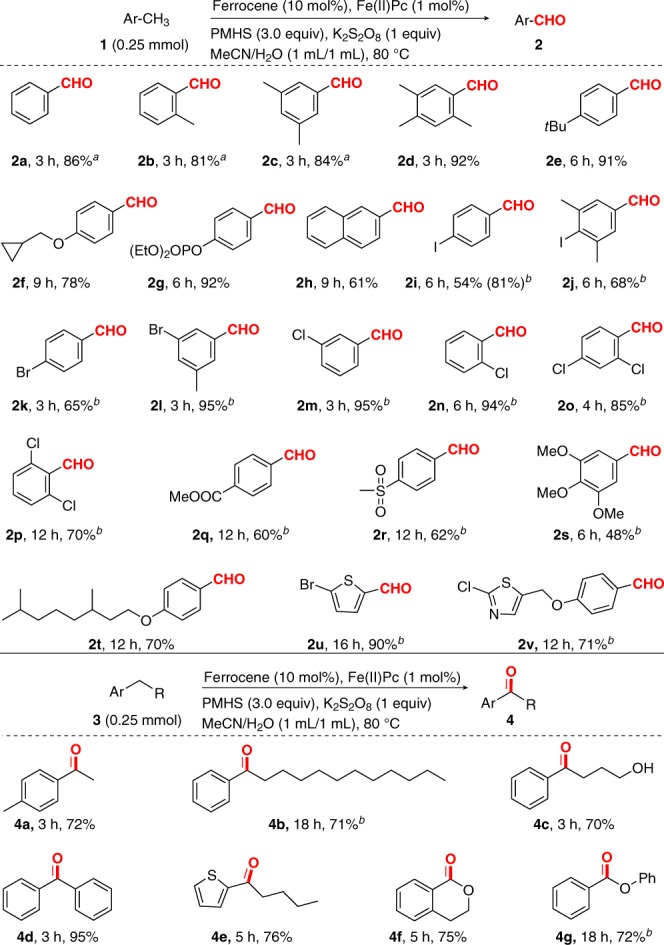
Iron-catalyzed oxidations of methyl aromatics and other alkyl aromatics. The substrate scope of methyl aromatics and other alkyl aromatics. Reaction conditions: substrate **1** or **3** (0.25 mmol), ferrocene (10 mol%), Fe(II) phthalocyanine (1 mol%), K_2_S_2_O_8_ (0.25 mmol), PMHS (0.75 mmol), CH_3_CN/H_2_O (1:1, 2.0 mL), 80 °C, and air; Yields of the isolated products are given. ^a^Based on ^1^H NMR analysis on the crude reaction mixture with chlorobenzene as the internal standard. ^b^K_2_S_2_O_8_ (0.75 mmol)

With the optimized reaction conditions in hand, we turned our attention to validate the generality of our oxidation of methylaromatics to aryl aldehydes protocol (Fig. 2). Toluene and polymethyl benzenes were selectively oxidized to corresponding aryl monoaldehydes in similarly high yields and selectivities (**2a**–**2d**), regardless of the positions of methyl groups in the toluene ring. To our delight, 4-*tert*-butyltoluene (**1e**) was submitted to the optimized reaction conditions to generate **2e** (91% yield), a key synthon for commercial perfume Lilial^45^. Previous synthese of **2e** based on 4-*tert*-butyltoluene (**1e**) suffered from efficiency and selectivity problems^45^. Similarly, diethyl 4-formylphenyl phosphate (**2g**), which is an important intermediate for the synthesis of a two-photon fluorogenic probe^46^, was also obtained in 92% yield in this oxidation system. Its manufacturing is known to be accessible from air-sensitive reagents (4-hydroxybenzaldehyde and diethyl phosphorochloridate)^46^. It is noteworthy that 2-methylnaphthalene, although known to undergo iron-catalyzed oxidation to the corresponding quinone^47^, can be well accommodated in a moderate yield (**2h**). When toluene ring had an electronically deactivated group, the reaction proceeded slowly but could achieve a high yield of arylaldehyde by increasing the loading of K_2_S_2_O_8_ to 3 equiv. Under the newly established conditions, methylaromatics bearing electron-withdrawing substituents, such as iodo, bromo, chloro, ester, and sulfonyl can be oxidized with good to excellent efficacies (**2i**–**2r**). It is notable that 2,6-dichlorobenzaldehyde (**2p)**, the direct precursor to dicloxacillin in the penicillin family of medications, has been notoriously difficult to prepare from 2,6-dichlorotoluene (**1p**) due to the strong steric hindrance of the *ortho*-dichloro substituents. Nevertheless, under our reaction conditions, this transformation was easily realized to provide **2p** in 70% yield. Methyl 4-methylbenzoate (**1q**) bearing a strong electron-withdrawing group also provided a moderate yield of methyl 4-formylbenzoate (**2q**). Analogously, methyl 4-tolyl sulfone (**1r**) satisfactorily yielded 4-(methyl sulfonyl)benzaldehyde (**2r**), an important synthon for the synthesis of trademarked drugs florfenicol and thiamphenicol. The reported synthese of this compound requires two steps (*N*-bromosuccinimide-mediated bromination followed by oxidation using *N*-methylmorpholine *N*-oxide as the oxidant) from methyl 4-tolyl sulfone (**1r**)^48^. Furthermore, 3,4,5-trimethoxybenzaldehyde (**2s**), a versatile starting material in the synthesis of some pharmaceutical drugs including trimethoprim, cintriamide, roletamide, trimethoquinol, and trimazosin, as well as some psychedelic phenethylamines, can be produced in a synthetically useful yield by our method. Previous preparation of **2s** from 1,2,3-trimethoxy-5-methylbenzene (**1s**) suffered from efficiency and selectivity problems^49^. When a molecule (**1t**) containing benzylic C–H and two aliphatic tertiary C–H was subjected to the reaction conditions, the C–H oxidation occurred site-selectively at the benzylic position. Remarkably, 2-bromo-5-methylthiophene which did not arrest catalysis can be easily oxidized to the thenaldehyde **2u** (90% yield) widely applied in the synthesis of a chemotherapeutic medication teniposide, a common hepatoprotectant tenylidone, and an insectifuge pyrantel on the World Health Organization’s List of Essential Medicines. To the best of our knowledge, thenaldehydes have not previously been accomplished by oxidation of the corresponding methylthiophenes in one step. Additionally, a heterocyclic substrate **1v** containing a heterobenzylic moiety and a benzylic position displayed completely selectivity for the benzylic position. It was noteworthy that the current a single-step route to these valuable molecules offers an appealing alternative to traditional pathways suffering from low efficiency, poor selectivity, and/or lengthy synthetic steps.

When toluene bearing a benzylic methylene group was subjected to oxidation under the above reaction conditions, it is expected that a ketone product **4a** derived from the benzylic methylene oxidation was obtained. Encouraged by this successful result, we examined representative alkylarenes with benzylic methylene moieties (Fig. 2). A challenging substrate dodecyl benzene (**3b**)^50^ for benzylic C–H oxidation proceeded well by increasing the amount of K_2_S_2_O_8_ to 3 equiv. (entry 2). Notably, the benzylic C–H oxidation took place efficiently in the presence of an unprotected primary alcohol (**3c**); this functionality typically interferes with currently used benzylic C–H oxidation protocols^37^. Diphenyl methane **3d** was also a suitable substrate. Gratifyingly, heteroarenes such as 2-pentylthiophene (**3e**) and isochromane (**3f**) can be easily oxidized to the ketone **4e** and the lactone **4f**, respectively. Phenylbenzoate (**4g**) can also be obtained in 72% yield through oxidation of benzylic C–H.

Aldehydoarylboronic acid possesses amphoteric properties with high synthetic versatility from its both nucleophilic (C–B bond) and electrophilic (C=O bond) moieties and attracts considerable interests across a wide spectrum of sciences from chemistry, materials, and biology to medicine^51^. For instance, 2-aldehydoarylboronic acids are readily transformed into approved drugs, such as tavaborole (for the treatment of onychomycosis), crisaborole (for the treatment of atopic dermatitis), and epetraborole (for the treatment of serious Gram-negative infections), and clinical trial drugs AN2898 (for the topical treatment of plaque and atopic psoriasis), and AN2718 (for the treatment of tinea pedis)^52^. To the best of our knowledge, a direct and general oxidation strategy for the construction of valuable aldehydoarylboronic acids has not been achieved to date, though a few examples of oxidation of prefunctionalized hydroxymethyl phenylborons to aldehydophenylborons have been reported^53,54^. Indeed, it is a long-standing challenge to make C–B bonds of organoboronic acids intact after subjecting to reactions with frequently used reagents like nucleophiles, bases, and organic acids, particularly with oxidants, owing to the unstable tricoordinate nature of their boron centers^55^.

Encouraged by the above gratifying results, we wondered whether the protocol could be extended to oxidation of methyl arylborons to aldehydoarylboronic acids. When 4-methylphenylboronic acid (**5a**) was employed as the model substrate, the corresponding aldehyde **6a** was obtained in 74% yield with satisfying chemoselectivity, accompanied by just 4% yield of overoxidation product acid (**6a′**) and little 4-methylphenol (**6a″**) under modified reaction conditions [The combination of iron(II) phthalocyanine (1 mol%) and ferrocene (10 mol%) taking the place of FeCl_2_ leads to a little bit more overoxidized phenol (originated from oxidation of C–B bond)] (for details, see Supplementary Table 2). Notably, addition of a sub-stoichiometric amount of tetraalkyl ammonium bromide (50 mol%) that has been demonstrated to react with peroxydisulfate producing a bis(tetraalkyl ammonium) peroxydisulfate, which is readily convertible to the tetraalkylammonium sulfate radical anions^56^, led to identification of tetra-*n*-butyl-ammonium bromide (TBAB) as a highly effective promotor, leading to **6a** in 83% yield.

**Fig. 3:**
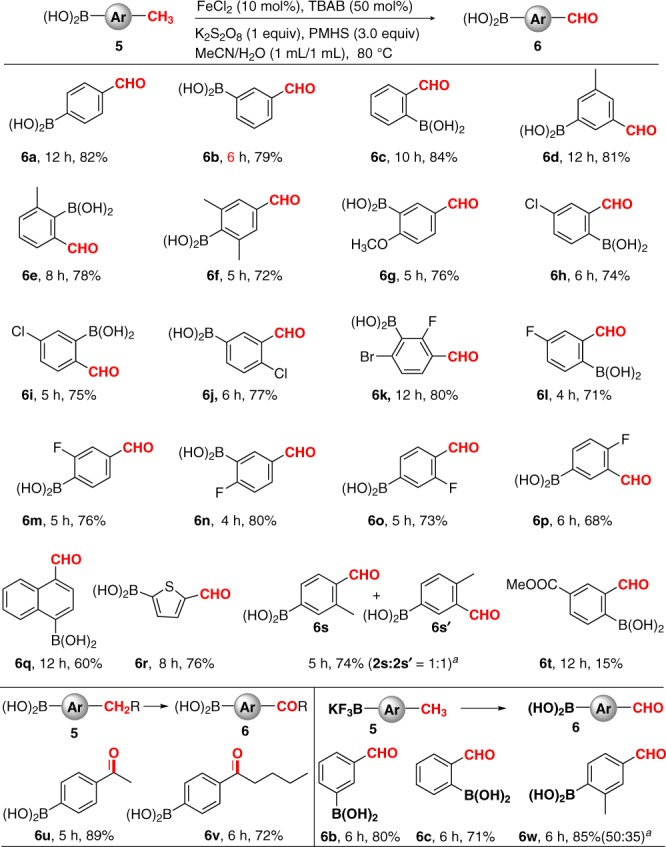
Substrate scope. The substrate scope of alkyl arylboronic acids and potassium methyl aryltrifluoroborates. Reaction conditions: substrate **5** (0.25 mmol), FeCl_2_ (10 mol%), TBAB (50 mol%), K_2_S_2_O_8_ (0.25 mmol), PMHS (0.75 mmol), CH_3_CN/H_2_O (1:1, 2.0 mL), 80 °C, and air; yields of the isolated products are given. ^a^Based on ^1^H NMR analysis

Next, we examined the substrate scope of alkyl arylborons as shown in Fig. 3. Methyl phenylboronic acids provided the corresponding aldehydophenylboronic acids in high yields (**6a–6c**). The position of the methyl substituent seems to have no influence on the product yield. Notably, multimethyl-substituted phenylboronic acids also readily participated in this oxidation reaction with satisfactory selectivity. For example, nonsterically hindered 3,5-dimethylphenylboronic acid and sterically hindered 2,6-dimethylphenylboronic acid were employed, delivering the desired products in 81% and 78% yields (**6d–6e**), respectively. When 2,4,6-trimethyl phenylboronic acid was used as a substrate, 4-formyl-2,6-dimethyl phenylboronic acid (**6f**) was obtained in 72% yield, thus indicating that nonsterically demanding methyl is more reactive. Additionally, two methyl groups having similar steric hindrance led to almost identical amounts of the corresponding two regioisomers like **6s**. The oxidation of methyl phenylboronic acid **5g** bearing a strong electron-donating methoxy group proceeded well. Furthermore, a series of methyl phenylboronic acids bearing electronically deactivated moieties, such as F, Cl, and Br, gave the corresponding aldehydophenylboronic acids in moderate to good yields with high selectivities (**6h**–**6p**), regardless of their positions on the benzene ring. Notably, these products possessed multiple orthogonal reactive groups [halo, B(OH)_2_, and CHO], which can undergo diverse transformations and would serve as versatile building blocks for construction of complex molecules. Additionally, 4-chloro-2-formylphenylboronic acid (**6h**), a key intermediate in the synthesis of a clinical trial drug AN2718 used for the treatment of tinea pedis, was produced in 74% yield under our reaction conditions. Similarly, 4-fluoro-2-formylphenylboronic acid (**6l**) which is the direct precursor to tavaborole used for the treatment of onychomycosis, was also obtained in 71% yield. Generally, **6h** and **6l** were prepared by a four-step route involving aldehyde protection, metallation (Li or Mg), borylation, and deprotection. To our delight, a naphthylboronic acid and a thiopheneboronic acid took part in the oxidation reactions smoothly to provide aldehydo arylboronic acids **6q** and **6r**, respectively. Unfortunately, 4-(methoxycarbonyl)-2-methylphenyl boronic acid was poorly reactive and gave the desired product in only 15% yield (**6t**). In addition to methyl arylboronic acids, alkyl arylboronic acids **5u**–**5v** also furnished the corresponding products **6u**–**6v** in good yields. Interestingly, when potassium methyl aryltrifluoroborates were subjected to the standard oxidation conditions, oxidation-hydrolysis products aldehydoarylboronic acids (**6b**–**6c** and **6w**) were obtained in high yields and high chemoselectivities, further broadening the scope of this oxidation method. For the **5w** containing two methyl groups, oxidation occurred more rapidly at less sterically hindered one, leading to provide **6w** as a 50:35 mixture of regioisomers.

**Fig. 4:**
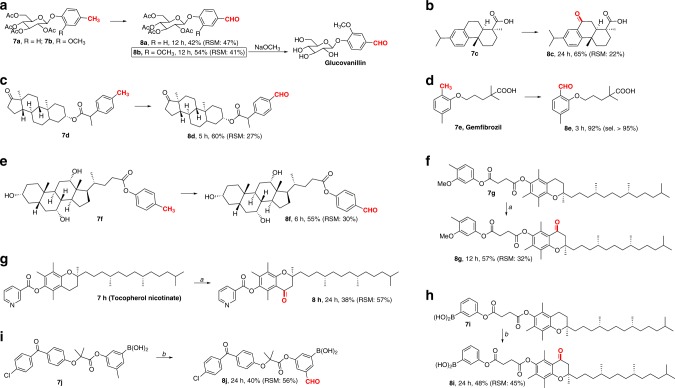
Late-stage oxidation of complex molecules. **a** Oxidation of **7a** and **7b**; **b** Oxidation of **7c**; **c** Oxidation of **7d**; **d** Oxidation of **7e**; **e** Oxidation of **7f**; **f** Oxidation of **7g**; **g** Oxidation of **7h**; **h** Oxidation of **7i**; **i** Oxidation of **7j**. Reported yields are for the isolated products. Reaction conditions: substrate **7** (0.25 mmol), ferrocene (10 mol%), Fe(II) phthalocyanine (1 mol%), K_2_S_2_O_8_ (0.25 mmol), PMHS (0.75 mmol), CH_3_CN/H_2_O (1:1, 2.0 mL), 80 °C, and air; RSM recovered starting material. ^a^K_2_S_2_O_8_ (0.75 mmol). ^b^Substrate (0.25 mmol), FeCl_2_ (10 mol%), TBAB (0.5 equiv), K_2_S_2_O_8_ (0.25 mmol), PMHS (3.0 equiv), CH_3_CN/H_2_O (1:1), 90 °C, O_2_ (1 atm)

To highlight the synthetic utility and versatility of this methodology, we conducted 5 mmol scale reactions of **1e** and **5a**, and isolated the desired products **2e** and **6a** in 90% and 79% yields, respectively. Furthermore, the robustness of this protocol was evaluated in the late-stage oxidation of an array of complex bioactive molecules (Fig. 4). Bioactive compounds **7a** and **7b** containing carbohydrate fragments were competent substrates and gave the corresponding aldehydes **8a** and **8b** in preparative yields. It is worth noting that the **8b** readily undergoes hydrolysis to afford a natural product Glucovanillin that is present in the green seed pods of *Vanilla planifolia* and is widely used in pharmaceutic aid. Additionally, dehydroabietic acid **7c**, a natural product can be oxidized efficiently to the compound **8c**, a BK channel-opening activity molecule^57^. The traditional synthetic method for **8c** based on **7c** requires the use of toxic and highly corrosive hexavalent compound chromium trioxide in stoichiometric excess^57^. Epiandrosterone-derived methylarene **7d** can also be easily oxidized to the aldehyde **8d** in 60% yield. Gemfibrozil (**7e**), an oral drug used to lower lipid levels, was also oxidized in 92% yield with more than 95% regioselectivity. Remarkably, cholic acid-derived methylarene **7f** having three hydroxy groups, did not compete with the efficacy of the oxidation event. As expected, secondary benzyl position was more reactive than the primary. For instance, a derivative of α-tocopheryl succinate with multiple reactive benzylic positions produced ketone **8g** in 57% yield with high selectivity; to our delight, a heterocyclic drug, tocopherol nicotinate **7h** provided oxidized product **8h** in a synthetically useful yield; and arylboronic acid **7i** containing a vitamin E fragment was selectively oxidized to the corresponding ketone **8i** in satisfactory yield by increasing the reaction temperature to 90 °C under O_2_ atmosphere (1 atm). A drug fenofibrate-derived arylboronic acid **7j** also proceeded well to provide the aldehyde **8j** in synthetic useful yield. To the best of our knowledge, the late-stage C–H oxidation of complex arylboronic acids has not been yet demonstrated, despite the great potential of this approach to drug synthesis and construction of complex molecules.

To highlight the unique advantage of the protocol in late-stage synthetic applications, a comparison of the newly established iron/PMHS catalytic system with previously reported benzylic oxygenation methods was investigated by oxidation of the oral drug gemfibrozil (**7e**) (Table 2). An efficient oxidation protocol for aryl(di)azinylmethanes that is based on base metal catalysts (Cu and Fe) totally failed to oxidize **7e** (entries 1 and 4)^25^. We also examined a typical catalyst system for benzylic aerobic oxidation of (hetero)arenes. Consequently, this catalyst system led to significant overoxidation (entry 2)^36^. Recently, a seminal advance in chemoselective oxidation of methylarenes to benzaldehydes was reported by Pappo group^14^. As reported in the literature, oxidation of gemfibrozil (**7e**) resulted in a 2:1 mixture of regioisomers with a moderate yield (entry 4)^14^. These results showcase the practicality of our protocol in organic synthesis.

**Table 2:** Comparison of catalyst systems


Entry	Reaction system	Ref.	Regioselectivity^a^	Yield (%)^b^
1	CuI/AcOH/DMSO	30	–	–
2	Co(OAc)_2_/NHPI/BuOAc	41	1:2	15(69)^c^
3	Co(OAc)_2_/NHPI/HFIP	16	2:1	67
4	FeCl_2_/AcOH/DMSO	30	–	–
5	Ferrocene-Fe(II)Pc/K_2_S_2_O_8_/PMHS	This work	>95:1	95

*NHPI N*-Hydroxyphthalimide, *HFIP* 1,1,1,3,3,3-Hexafluoro-2-propanol

^a^Ratios of **8e**/**8e′** were determined by ^1^H NMR with anisole as an internal standard

^b^Yields were determined by ^1^H NMR with anisole as an internal standard

^c^A mixture of overoxidized products with a ratio of 1:2

### Mechanistic studies

**Fig. 5:**
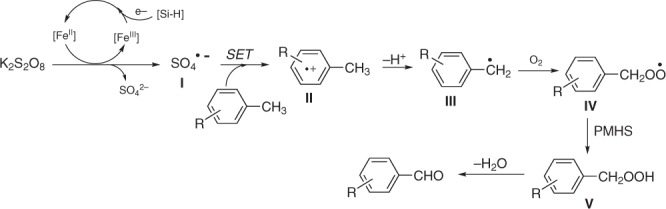
Proposed mechanistic cycle. This reaction begins with the oxidation of Fe(II) by persulfate to generate the Fe(III) species and the sulfate radical anion **I** that reacts with alkylarene by single electron transfer (SET) to produce the alkylaromatic radical cation **II**

**Fig. 6:**
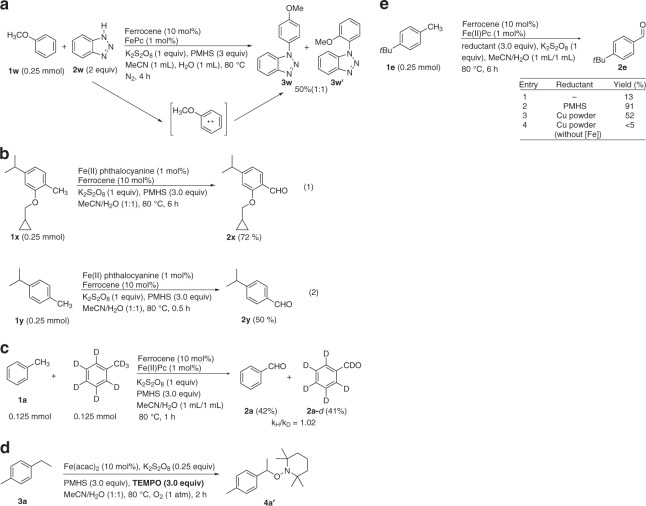
Mechanistic experiments. **a** Probation for the presence of aromantic radical cation in the catalytic system. **b** Comparison of the reactivity between methyl and isopropyl groups in the catalytic system. **c** Intermolecular kinetic isotope effect. **d** Radical capture experiment. **e** Control experiments proving the reducing property of PMHS in the catalytic system

On the basis of our results and previous studies^4,58–68^, a plausible mechanistic proposal for this reaction is depicted in Fig. 5. This reaction begins with the oxidation of Fe(II) by persulfate to generate the Fe(III) species and the sulfate radical anion **I**^58^ that reacts with alkylarene by single electron transfer (SET) to produce the alkylaromatic radical cation **II**^59–61^. Intermediate **II** is extremely acidic and readily undergoes benzylic deprotonation to give the benzyl radical **III**^59–61^. Anisole (**1w**) can suffer one-electron oxidation to form the corresponding radical cation, which is attacked by nucleophilic benzotriazole to provide aryl amine^62,63^. Submission of **1w** to our normal reaction conditions also obtained the aryl amine in 50% yield (regioisomers: *p*:*o* = 1:1) (Fig. 6a), suggesting that aromatic radical cation can be produced in our catalytic system. Moreover, probe substrates (**1x** and **1y**) containing isopropyl and methyl substitutes were subjected to the standard reaction conditions and gave the corresponding single methyl oxidation products **2x** (72%) and **2y** (50%) with the retention of the isopropyl groups (Fig. 6b). This findings rule out that sulfate radical anion **I** may abstract a hydrogen atom from alkylarene to produce the benzyl radical **III** (because oxidation of tertiary benzylic C–H is much easier than primary benzylic C–H by hydrogen atom transfer mechanism)^64^ and further support the process of SET to produce the alkylaromatic radical cation^60^. The kinetic isotope effect (KIE) for oxidation of toluene and toluene-*d*_8_ in separate vessels from initial reaction rates was observed to be 1.02 (Fig. 6c), indicating that the deprotonation is rapid. Furthermore, the presence of **III** was confirmed by the capture of the carbon-centered radical intermediate with the radical scavenger 2,2,6,6-tetramethylpiperidinooxy (TEMPO) (Fig. 6d). Copper that is commonly used in the reduction of ferric ions to ferrous ions^66^ taking the place of PMHS in the oxidation of **1e** enables generation of **2e** in a moderate yield (Fig. 6e), while in the absence of any reductant or iron catalyst little product was obtained (entries 1 and 4). These results imply that PMHS maybe an effective reducing agent to achieve recycling of the iron^III^ end species in this oxidation reaction. Subsequently, the benzyl radical **III** readily reacts with molecular oxygen to produce the benzyl peroxide radical **IV**^14,67^, followed by abstraction of a proton from PMHS (supported by large drop off in yield when the loading of PMHS was lowered to 2.0 equiv, see Supplementary Table 1)^65^ to generate the hydroperoxide **V**. Considering that tertiary benzylic C–H can be survived under our system (Fig. 6b), abstraction of a proton from a substrate could be ruled out. Finally, the elimination of H_2_O from the hydroperoxide **V** may occur and yields arylaldehyde^4,14^. However, we cannot exclude the possibility that aldehyde formation occurs via the self-reaction of two benzylperoxyl radicals^68^.

**Fig. 7:**
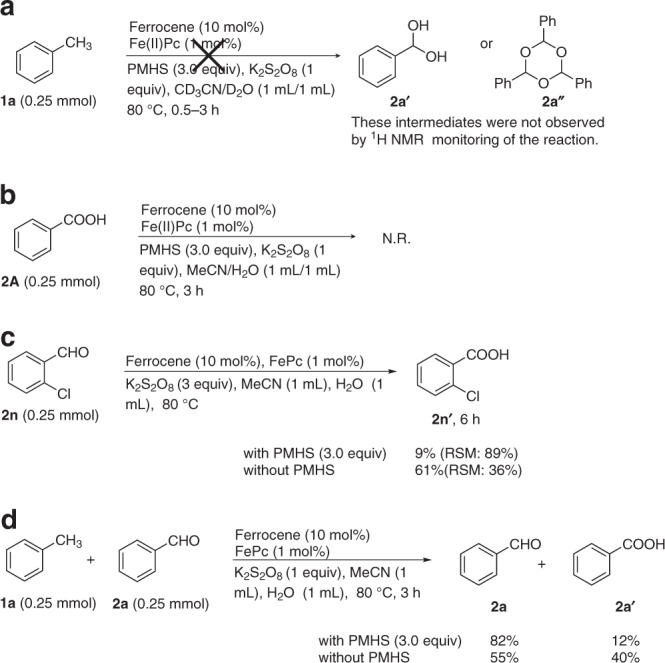
Control experiments for probing the unique chemoselectivity for aldehydes. **a** ^1^H NMR monitoring the potential intermediates of the model reaction. **b** The reaction using benzoic acid as a substrate. **c** The effect of PMHS on the oxidation of **2n**; RSM recovered starting material. **d** Competition experiments between toluene (**1a**) and benzaldehyde (**2a**) in the presence of K_2_S_2_O_8_ and the iron catalyst with and without PMHS

According to the above electron transfer mechanism, we readily conclude that alkylbenzenes are more easily oxidizable than arylaldehydes in our catalytic system because arenes bearing strong electron-withdrawing groups (e.g. CHO) are difficult to produce the aromatic radical cations^59–61^. This is the intrinsic factor of the unique chemoselectivity for arylaldehydes in the present catalytic aerobic oxidation of methylarenes system. The experimental observations on the exceptional high regioselectivity can also be fully rationalized. For instance, oxidation of substrate (**1t**, **3b**, **7a–d**, or **7f–i**) occurs selectively at the benzylic position in the presence of multiple secondary and tertiary C–H bonds in line with the intermediacy of an aromatic radical cation; the benzylic oxidation of substrate **1v** (Fig. 2) is selective for reaction at the more electron-rich aromatic ring, again in line with the electron transfer mechanism; selective benzylic oxidation over aliphatic alcohol oxidation observed with substrate **3c** is again indicative of a reaction that proceeds via electron transfer from the aromatic ring and not via hydrogen atom transfer; and the benzylic selectivity observed in the oxidation of substrate **3f** or **7e** can be attributed to enhance the stability of the radical cation intermediate in the presence of the adjacent alkoxy substituent. Additionally, some control experiments were designed and investigated to probe other factors for the high chemoselectivity for aldehydes (Fig. 7). First, we used ^1^H NMR to monitor the reaction of **1a** under normal conditions and did not observe the presence of the aldehyde hydrate **2a′** and the aldehyde trimer **2aʺ** that might protect the aldehyde from further oxidation (Fig. 7a). PMHS has been reported as a reductant in the iron-catalyzed reduction of carboxylic acids to aldehydes^69^. In order to probe the possibility of this process in our catalytic system, over-oxidized product **2A** was subjected to the standard reaction conditions, and 100% **2A** was recovered without any aldehyde **2a** (Fig. 7b). This control experiment demonstrated that the high chemoselectivity for aldehyde did not result from reduction of over-oxidized carboxylic acid to aldehyde. Next, exposing product aldehyde **2n** to our reaction conditions resulted in only 9% yield of acid **2n′** along with recovery of 89% aldehyde **2n**. In contrast, in the absence of PMHS the reaction yielded the over-oxidized product **2n**′ high up to 61% (Fig. 7c). Additionally, competition experiments between toluene (**1a**) and benzaldehyde (**2a**) in the presence of K_2_S_2_O_8_ and the iron catalyst with and without PMHS were conducted and also gave similar results, as shown in Fig. 7d. These observations indicate PMHS suppresses the overoxidation of aldehyde, implying that PMHS plays an important role in achieving high chemoselectivity for aldehyde in the present transformation. To investigate whether there was an interaction between PMHS and benzaldehyde, we used the Job’s method^70^ of continuous variations for determining the PMHS/benzaldehyde complex by recording the ^13^C NMR spectra of the aldehyde group with a constant total concentration of PMHS and benzaldehyde. The resulting curves, called Job’s plots, did not yield a maximum^14^, indicating nonexistence of a strong binding between PMHS and the aldehyde, and thus implying that PMHS interacting with the aldehyde and protecting it towards further oxidation could be ruled out.

## Discussion

In summary, we have developed a general and chemoselective iron-catalyzed aerobic oxidation of benzylic C–H bonds through a bio-inspired PMHS-driven oxidation process. In this transformation, methylarenes and alkylarenes are employed as limiting reagents to provide desired aryl-aldehydes, aryl-ketones, and aryl-esters efficiently. (Although this reaction is exceedingly general in its current form, substrates such as nitrotoluenes, alkyl pyridines, and alkyl phenols, result in <20% yields of the desired products under our reaction conditions.) This work also overcomes the long-standing challenge that boronic acid group is normally required to be transformed immediately after its introduction, and represents a significant advance in the area of the chemistry of organoboronic acids, including the ability to incorporate both aldehyde and ketone functionalities into unprotected arylboronic acids, a class that can be difficult to access by current means. Notably, this method allows for tolerance toward protic functional groups and is particularly useful in late-stage oxidation of complex small molecules, which has not been demonstrated for any other catalytic oxidation of methylarenes to date. Overall, the results offer an excellent option toward establishing a horizon for oxidation of inexpensive, readily available methyl-arenes and alkyl-arenes. Further studies into the use of the powerful bio-inspired oxidation system for other oxidation reactions, as well as more mechanistic investigations, are ongoing in our laboratory.

## Methods

### General procedure for the oxidation of alkylarene

A 25-mL flask was charged with ferrocene (4.7 mg, 0.025 mmol), ferrous phthalocyanine (1.5 mg, 0.0025 mmol), K_2_S_2_O_8_ (68.3 mg, 0.25 mmol), methylarene or alkylarene (0.25 mmol), MeCN (1.0 mL), H_2_O (1.0 mL), and PMHS (170 μL, 0.75 mmol). The mixture was stirred under 80 ℃ and atmospheric pressure for the indicated time. Then 0.5 mL ammonia water was added into the mixture with vigorous stirring at room temperature. The reaction mixture was diluted with a saturated aqueous NaCl solution (10 mL) and then extracted with diethyl ether (3 × 10 mL). The organic phases were combined and evaporated under reduced pressure. The residue was purified by column chromatography (petroleum ether/diethyl ether) on silica gel to afford the corresponding product.

### General procedure for the oxidation of alkyl arylboron

A 25 mL flask was charged with FeCl_2_ (3.2 mg, 0.025 mmol), arylboron (0.25 mmol), K_2_S_2_O_8_ (68.3 mg, 0.25 mmol), TBAB (40.7 mg, 0.125 mmol), MeCN (1 mL), H_2_O (1 mL), and PMHS (170 μL, 0.75 mmol). The reaction mixture was stirred under atmospheric pressure at 80 °C until the reaction was complete (observed by TLC). After the mixture was cooled to room temperature, the reaction mixture was diluted with 10 mL brine and extracted with ethyl acetate (3 × 10 mL). The organic phases were combined and concentrated to give the crude product. The residue was purified by column chromatography (petroleum ether/ethyl acetate) on silica gel to afford the corresponding product.

## Supplementary information

Supplementary Information

## Data Availability

Experimental details, characterization of compounds, copies of NMR data and details of mechanistic experiments are available in the Supplementary Information.
