# Supplementary material for: Bio-inspired iron-catalyzed oxidation of alkylarenes enables late-stage oxidation of complex methylarenes to arylaldehydes
Source: Nat Commun. 2019 Jun 3;10:2425. doi: 10.1038/s41467-019-10414-7 (PMC6546739; doi:10.1038/s41467-019-10414-7)
Supplement: Supplementary file 1 — Supplementary Information [file 41467_2019_10414_MOESM1_ESM.pdf]

– Supporting Information –

Bio-inspired iron-catalyzed oxidation of alkylarenes enables late-stage oxidation of complex methylarenes to arylaldehydes

Hu *et al.*

Supplementary Notes

General information

All reactions were carried out under air atmosphere unless otherwise noted.All reagents and solvents were obtained from commercial suppliers such as $FeCl_2$ (anhydrous, 99.99% pure) from Aldrich, Fe(acac)$_2$(99.95% pure) from Aldrich, PMHS [CAS:9004-73-3, 15 to 40 mPa.s (at 20°C)] from Acros, CH_3CN (distilled) from Adamas-beta Ltd. and used without further purification. Reactions were monitored by TLC on silica gel plates (GF254). ^{1}H (400 MHz) and ^{13}C NMR spectra (100 MHz) of solutions in $CDCl_3$, Acetone-d_6 or DMSO-d_6 were recorded on a Bruker Avance400 NMR spectrometer. Chemical shifts were expressed in parts per million (ppm) downfield from tetramethylsilane and refer to the solvent signals ($CDCl_3$: δ_H 7.28 and δ_C 77.0 ppm; Acetone-d_6: δ_H 2.05 and δ_C 29.84, 206.26 ppm; DMSO-d_6: δ_H 2.50 and δ_C 39.50 ppm). The signals of water were observed at about 1.58 ppm in $CDCl_3$, 2.84 ppm inAcetone-d_6 and 3.33 ppm in DMSO-d_6, respectively. Abbreviations for signal couplings are: br, broad; s, singlet; d, doublet; t, triplet; m, multiplet; dd, doublet of doublets; dt, triplet of doublets;td, doublet of triplets; tt, triplet of triplets; tdd, doublet of doublet of triplets. Coupling constants, *J,* were reported in hertz unit (Hz). HRMS was performed on a Q-TOF mass spectrometer. Infrared spectra of neat substances were recorded on a Thermo Nicolet Corporation GC-FTIR NEXUS670 spectrometer. GC-MS were determined with Agilent 7890-5975C. ***Note: The boron-bound carbon was not observed due to quadrupolar relaxation.***

General procedures for Fe-catalyzed oxidation of alkylarenes

General Procedure A: A 25-ml flask was charged with Ferrocene (4.7 mg, 0.025 mmol), Ferrous phthalocyanine (1.5 mg, 0.0025 mmol), $K_2S_2O_8$ (68.3 mg, 0.25 mmol), methylarene or alkylarene (0.25 mmol), MeCN (1.0 mL), H_2O (1.0 mL), and PMHS (170 μL, 0.75 mmol). The mixture was stirred under 80℃ and atmospheric pressure for the indicated time. Then 0.5 mL ammonia water was added into the mixture with vigorous stirring at room temperature. The reaction mixture was diluted with a saturated aqueous NaCl solution (10 mL) and then extracted with diethyl ether (3 × 10 ml). The organic phases were combined and evaporated under reduced pressure. The residue was purified by column chromatography (Petroleum ether/ diethyl ether) on silica gel to afford the corresponding product.

General Procedure B: A 25 mL flask was charged with $FeCl_2$ (3.2 mg, 0.025 mmol), arylboron (0.25 mmol), $K_2S_2O_8$ (68.3 mg, 0.25 mmol), TBAB (40.7 mg, 0.125 mmol), MeCN (1 mL), H_2O (1mL), and PMHS (170 μL, 0.75 mmol). The reaction mixture was stirred under atmospheric pressure at 80 °C until the reaction was complete (observed by TLC). After the mixture was cooled to room temperature, the reaction mixture was diluted with 10 mL brine and extracted with ethyl acetate (3 × 10 mL). The organic phases were combined and concentrated to give the crude product. The residue was purified by column

chromatography (Petroleum ether/ ethyl acetate) on silica gel to afford the corresponding product.

Experimental data for the products

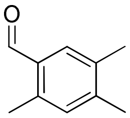

2,4,5-Trimethylbenzaldehyde (2d): Following the *general procedure A*, **2d** was isolated as a white solid (33.9 mg, 92%), known compound. The NMR spectroscopic data agree with those described in ref.[S1]. ^{1}H NMR (400 MHz, $CDCl_3$) δ: 10.21 (s, 1H), 7.57 (s, 1H), 7.04 (s, 1H), 2.61 (s, 3H), 2.31 ppm (s, 6H); ^{13}C NMR (100 MHz, $CDCl_3$) δ: 192.6, 143.4, 138.1, 134.6, 133.1, 133.0, 132.0, 19.9, 19.0, 18.9 ppm; mp: 41.0 - 43.2 ℃.

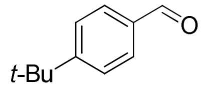

4-(*Tert*-butyl)benzaldehyde (2e): Following the *general procedure A*, **2e** was isolated as a colorless liquid (36.4 mg, 91%), known compound. The NMRspectroscopic data agree with those described in ref.[S2]. ^{1}H NMR (400 MHz, $CDCl_3$) δ: 10.0 (s, 1H), 7.85 (d, *J* = 8.55 Hz, 2H), 7.58 (d, *J* = 8.55 Hz, 2H), 1.37 ppm (s, 9H); ^{13}C NMR (100 MHz, $CDCl_3$) δ: 192.1, 158.5, 134.0, 129.7, 125.9, 35.4, 31.1 ppm.

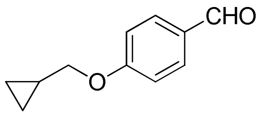

4-**(Cyclopropylmethoxy)benzaldehyde (2f):** Following the *general procedure A*, **2f** was isolated as colorless oil (34.4 mg, 78%), known compound. The NMR spectroscopic data agree those described in ref.[S3]. ^{1}H NMR (400 MHz, $CDCl_3$): δ 9.86 (s, 1H), 7.81 (d, *J* = 8.6 Hz, 2H), 6.98 (d, *J* = 8.6 Hz, 2H), 3.87 (d, *J* = 7.0 Hz, 2H), 1.28 (m, 1H), 0.66 (m, 2H), 0.36 ppm (m, 2H); ^{13}C NMR (100 MHz, $CDCl_3$): δ 190.8, 164.0, 131.9, 129.7, 114.7, 73.1, 10.0, 3.2 ppm.

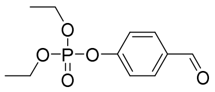

Diethyl 4-formylphenyl phosphate (**2g**). Following the *general procedure A*, **2g** was isolated as light yellow oil (59.0 mg, 92%). The NMR spectroscopic data agree with those described in ref.[S4]. ^{1}H NMR (400 MHz, $CDCl_3$): δ 9.97 (s, 1 H), 7.89 (d, *J*=8.4 Hz, 2H), 7.38 (d, *J*= 8.4 Hz, 2 H), 4.28-4.20 (m, 4 H), 1.37 ppm (dt, *J*= 7.2, 0.8 Hz, 6 H); ^{13}C NMR (100 MHz, $CDCl_3$): δ 190.7, 155.4 (d, *J* = 6.6 Hz), 133.2, 131.7, 120.5 (d, *J* = 5.2 Hz), 65.0 (d, *J* = 6.1 Hz), 16.1 ppm (d, *J* = 6.6 Hz). ^{31}P NMR (162 MHz, $CDCl_3$): δ -6.64.

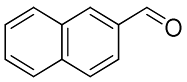

2-Naphthaldehyde (2h): Following the *general procedure A*, **2h** was isolated as a white solid (23.6 mg, 61%), known compound. The NMR spectroscopic data agree with those described in ref.[S5]. ^{1}H NMR (400 MHz, $CDCl_3$) δ: 10.17, (s, 1H),

8.34 (s, 1H), 8.02 - 7.90 (m, 4H), 7.68 - 7.58 ppm (m, 2H); ^{13}C NMR (100 MHz, $CDCl_3$) δ: 192.2, 136.4, 134.5, 134.0, 132.6, 129.5, 129.1, 129.0, 128.0, 127.0, 122.7 ppm; mp: 58.8 - 60.1℃.

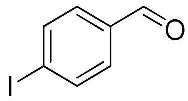

4-Iodobenzaldehyde (2i): Following the *general procedure A* except with $K_2S_2O_8$ (204.9 mg, 0.75 mmol), **2i** was isolated as a yellow liquid (46.8 mg, 81%), known compound. The NMR spectroscopic data agree with those described in ref.[S6]. ^{1}H NMR (400 MHz, CDCl3): δ 9.98 (s, 1H), 7.94 (d, J = 8.24 Hz, 2H), 7.62 ppm (d, J = 8.24 Hz, 2H); ^{13}C NMR (100 MHz, $CDCl_3$): δ 191.4, 138.4, 135.5, 130.8, 102.8 ppm.

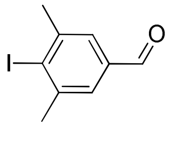

4-Iodo-3,5-dimethylbenzaldehyde (2j): Following the *general procedure A* except with $K_2S_2O_8$ (204.9 mg, 0.75 mmol), **2j** was isolated as colorless liquid (44.1 mg, 68%), known compound. The NMR spectroscopic data agree with those described in ref.[S7]. ^{1}H NMR (400 MHz, $CDCl_3$): δ 9.96 (s, 1H), 7.54 (s, 2H), 2.58 ppm (s, 6H); ^{13}C NMR (100 MHz, $CDCl_3$): δ 191.9, 143.4, 135.5, 127.2, 116.8, 29.7 ppm.

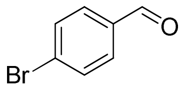

4-Bromobenzaldehyde (2k): Following the *general procedure A* except with $K_2S_2O_8$ (204.9 mg, 0.75 mmol), **2k** was isolated as a white solid (30.0 mg, 65%), known compound. The NMR spectroscopic data agree with those described in ref.[S8]. ^{1}H NMR (400 MHz, $CDCl_3$): δ 9.98 (s, 1H), 7.76 (d, J = 8.32 Hz, 2H), 7.70 ppm (d, J = 8.32 Hz, 2H); ^{13}C NMR (100 MHz, $CDCl_3$): δ 191.0, 135.0, 132.4, 130.9, 129.7 ppm; mp: 55.4 - 56.3 ℃.

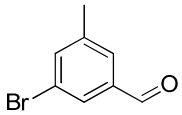

3-Bromo-5-methylbenzaldehyde (2l): Following the *general procedure A* except with $K_2S_2O_8$ (204.9 mg, 0.75 mmol), **2l** was isolated as yellow liquid (47.2 mg, 95%), known compound. The NMR spectroscopic data agree with those described in ref.[S9]. ^{1}H NMR (400 MHz, $CDCl_3$): δ 9.94 (s, 1H), 7.83 (s, 1H), 7.63 (s, 1H), 7.61 (s, 1H), 2.44 ppm (s, 3H); ^{13}C NMR (100 MHz, $CDCl_3$): δ 190.9, 141.1, 137.9, 137.8, 129.7, 128.9, 123.1, 20.9 ppm.

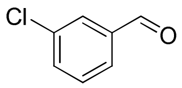

3-Chlorobenzaldehyde (2m): Following the *general procedure A* except with $K_2S_2O_8$ (204.9 mg, 0.75 mmol), **2m** was isolated as colorless liquid (33.4 mg, 95%), known compound. The NMR spectroscopic data agree with those described in

ref.[S10]. ^{1}H NMR (400 MHz, $CDCl_3$): δ 9.95 (s, 1H), 7.82 (s, 1H), 7.74 (d, J = 7.60 Hz, 1H), 7.57 (dd, J = 7.60, 3.6 Hz, 1H), 7.46 ppm (td, J = 7.60, 2.0 Hz, 1H); ^{13}C NMR (100 MHz, $CDCl_3$): δ 190.8, 137.6, 135.3, 134.2, 130.3, 129.1, 127.9 ppm.

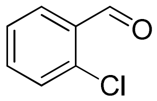

2-Chlorobenzaldehyde (2n): Following the *general procedure A* except with $K_2S_2O_8$ (204.9 mg, 0.75 mmol), **2n** was isolated as colorless liquid (32.9 mg, 94%), known compound. The NMR spectroscopic data agree with those described in ref.[S10]. ^{1}H NMR (400 MHz, $CDCl_3$): δ 10.48 (s, 1H), 7.93 (dd, J_1 = 7.75 Hz, J_2 = 1.69 Hz, 1H), 7.53 - 7.51 (m, 1H), 7.45 (d, J = 7.60 Hz, 1H), 7.40 ppm (t, J = 7.63 Hz, 1H); ^{13}C NMR (100 MHz, $CDCl_3$): δ 189.7, 137.8, 135.1, 132.3, 130.5, 129.3, 127.2 ppm.

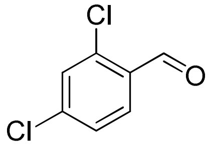

2,4-Dichlorobenzaldehyde (2o): Following the *general procedure A* except with $K_2S_2O_8$ (204.9 mg, 0.75 mmol), **2o** was isolated as a yellow solid (36.9 mg, 85%), known compound. The NMR spectroscopic data agree with those described in ref.[S11]. ^{1}H NMR (400 MHz, $CDCl_3$): δ 10.44 (s, 1H), 7.91 (d, J = 8.40 Hz, 1H), 7.51 (d, J = 2.0 Hz, 1H), 7.40 ppm (ddd, J_1 = 8.40, 2.0, 0.8 Hz, 1H); ^{13}C NMR (100 MHz, $CDCl_3$): δ 188.5, 141.1, 138.5, 130.9, 130.5, 130.3, 127.9 ppm; mp: 68.2 - 69.3 ℃.

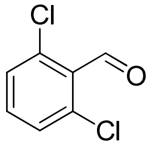

2,6-Dichlorobenzaldehyde (2p): Following the *general procedure A* except with $K_2S_2O_8$ (204.9 mg, 0.75 mmol), **2p** was isolated as a white solid (30.4 mg, 70%), known compound. The NMR spectroscopic data agree with those described in ref.[S12]. ^{1}H NMR (400 MHz, $CDCl_3$) δ: 10.52 (s, 1H), 7.42 ppm (s, 3H); ^{13}C NMR (100 MHz, $CDCl_3$) : δ 188.8, 136.9, 133.6, 130.4, 129.7 ppm; mp: 69.3 - 70.2 ℃.

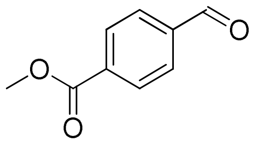

Methyl 4-formylbenzoate (2q): Following the *general procedure A* except with $K_2S_2O_8$ (204.9 mg, 0.75 mmol), **2q** was isolated as a white solid (24.5 mg, 60%), known compound. The NMR spectroscopic data agree with those described in ref.[S13]. ^{1}H NMR (400 MHz, $CDCl_3$): δ 10.10 (s, 1H), 8.20 (d, J = 8.40 Hz, 2H), 7.95 (d, J = 8.40 Hz, 2H), 3.96 ppm (s, 3H); ^{13}C NMR (100 MHz, $CDCl_3$): δ 191.6, 166.0, 139.1, 135.1, 130.2, 129.5, 52.6 ppm; mp: 62.2-63.1℃.

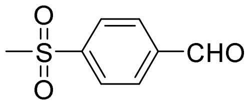

4-(Methylsulfonyl)benzaldehyde (**2r**): Following the *general procedure A* except with $K_2S_2O_8$ (204.9 mg, 0.75 mmol), **2r** was isolated as a white solid (28.5 mg, 62%), known compound. The NMR spectroscopic data agree those described in ref.[14]. ^{1}H NMR (400 MHz, acetone-d_6): δ 10.2 (s, 1H), 8.20(d, J = 8.8 Hz, 2H), 8.18 (d, J = 8.8 Hz, 2H), 3.22 ppm (s, 3H); ^{13}C NMR (100 MHz, acetone-d_6): δ 192.5, 146.9, 140.8, 131.1, 129.0, 44.1 ppm; mp: 155.1-156.3 ℃.

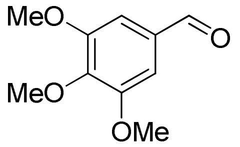

3,4,5-Trimethoxybenzaldehyde (2s): Following the *general procedure A* except with $K_2S_2O_8$ (204.9 mg, 0.75 mmol), **2s** was isolated as a white solid (23.5 mg, 48%), known compound. The NMR spectroscopic data agree with those described in ref.[S15]. ^{1}H NMR (400 MHz, $CDCl_3$): δ 9.87 (s, 1H), 7.14 (s, 2H), 3.95 (s, 3H), 3.94 ppm (s, 6H); ^{13}C NMR (100 MHz, $CDCl_3$): δ 191.0, 153.6, 143.6, 131.7, 106.7, 60.9, 56.2 ppm; mp: 72.4 - 74.3 ℃.

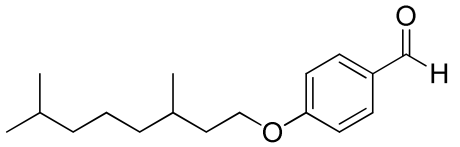

4-((3,7-Dimethyloctyl)oxy)benzaldehyde (2t): Following the *general procedure A*, **2t** was isolated as a yellow liquid (45.6 mg, 70%), known compound. The NMR spectroscopic data agree those described in ref.[S16]. ^{1}H NMR (400 MHz, $CDCl_3$): δ 9.87 (s, 1H), 7.82 (d, J = 8.8 Hz, 2H), 6.99 (d, J = 8.8 Hz, 2H), 4.09-4.05 (m, 2H), 1.87-1.81 (m, 1H), 1.65-1.60 (m, 2H), 1.56-1.49 (m, 1H), 1.35-1.14 (m, 6H), 0.94 (d, J = 6.8 Hz, 3H), 0.86 ppm (d, J = 6.8 Hz, 6H); ^{13}C NMR (100 MHz, $CDCl_3$): δ 190.8, 164.2, 132.0, 129.7, 114.7, 66.7, 39.2, 37.2, 35.9, 29.8, 27.9, 24.6, 22.7, 22.6, 19.6 ppm.

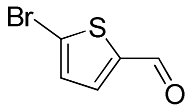

5-Bromothiophene-2-carbaldehyde (2u): Following the *general procedure A* except with $K_2S_2O_8$ (204.9 mg, 0.75 mmol), **2u** was isolated as yellow liquid (42.8 mg, 90%), known compound. The NMR spectroscopic data agree with those described in ref.[S17]. ^{1}H NMR (400 MHz, $CDCl_3$): δ 9.79 (s, 1H), 7.55 (d, J = 4.0 Hz, 2H), 7.22 ppm (d, J = 4.0 Hz, 1H); ^{13}C NMR (100 MHz, $CDCl_3$): δ 181.8, 145.1, 136.5, 131.4, 125.0 ppm .

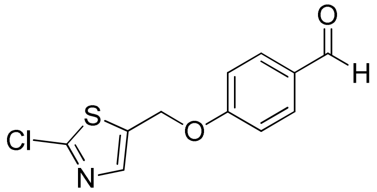

4-((2-Chlorothiazol-5-yl)methoxy)benzaldehyde (2v): Following the *general procedure A* except with $K_2S_2O_8$ (204.9 mg, 0.75 mmol), **2v** was isolated as a Yellow liquid (57.8 mg, 71%), known compound. The NMR spectroscopic data agree those described in ref.[S18]. ^{1}H NMR (400 MHz, $CDCl_3$): δ 9.90 (s, 1H), 7.86 (d, J = 8.6 Hz, 2H), 7.58 (s, 1H), 7.05 (d, J = 8.6 Hz, 2H), 5.27 ppm (s, 2H); ^{13}C NMR (100 MHz, $CDCl_3$): δ 190.7, 162.4, 153.2, 140.5, 135.2, 132.0, 130.7, 115.0, 62.6 ppm; mp: 103.3-104.2℃.

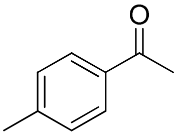

1-(*p*-Tolyl)ethanone (4a): Following the *general procedure A*, **4a** was isolated as colorless liquid (24.1 mg, 72%), known compound. The NMR spectroscopic data agree with those described in ref.[S19]. ^{1}H NMR (400 MHz, $CDCl_3$): δ 7.89 (d, *J* = 8.18 Hz, 2H), 7.28 (d, *J* = 8.18 Hz, 2H), 2.60 (s, 3H), 2.43 ppm (s, 3H); ^{13}C NMR (100 MHz, $CDCl_3$): δ 197.9, 143.9, 134.7, 129.2, 128.5, 26.6, 21.7 ppm.

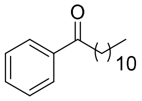

Dodecanophenone (4b): Following the *general procedure A* except with $K_2S_2O_8$ (204.9 mg, 0.75 mmol), **4b** was isolated as a white solid (45.8 mg, 71%), known compound. The NMR spectroscopic data agree with those described in ref.[S20]. ^{1}H NMR (400 MHz, $CDCl_3$): δ 7.98 (d, *J* = 7.54 Hz, 2H), 7.57 (t, *J* = 7.20 Hz, 1H), 7.50 (t, *J* = 7.20 Hz, 2H), 3.00 (t, *J* = 7.43 Hz, 2H), 1.79 (m, 2H), 1.45 - 1.28 (m, 16H), 0.91 ppm (t, *J* = 6.83 Hz, 3H); ^{13}C NMR (100 MHz, $CDCl_3$): δ 200.6, 137.1, 132.8, 128.5, 128.0, 38.6, 31.9, 29.6, 29.5, 29.4, 29.3, 29.3, 24.4, 22.7, 14.1, 1.0 ppm; mp: 45.3 - 47.2 ℃.

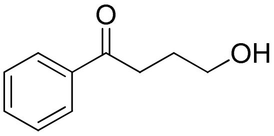

4-Hydroxy-1-phenylbutan-1-one (4c): Following the *general procedure A*, **4c** was isolated as colorless liquid (28.6 mg, 70%), known compound. The NMR spectroscopic data agree with those described in ref.[S21]. ^{1}H NMR (400 MHz, $CDCl_3$): δ 8.0 (d, *J* = 7.60 Hz, 2H), 7.59 (t, *J* = 7.60 Hz, 1H), 7.49 (t, *J* = 7.60 Hz, 2H), 3.77 (s, 2H), 3.17 (t, *J* = 6.40 Hz, 2H), 2.07 - 2.01 (m, 2H), 1.79 ppm (brs, 1H); ^{13}C NMR (100 MHz, $CDCl_3$): δ 200.5, 136.9, 133.1, 128.6, 128.1, 62.3, 35.3, 26.9 ppm.

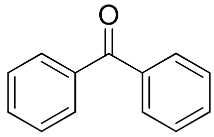

Benzophenone (4d): Following the *general procedure A*, **4d** was isolated as a white solid (43.1 mg, 95%), known compound. The NMR spectroscopic data agree with those described in ref.[S22]. ^{1}H NMR (400 MHz, $CDCl_3$): δ 7.85 - 7.52 (m, 2H), 7.61 (tt, *J* = 7.20, 1.20 Hz, 1H), 7.52 - 7.49 ppm (m, 2H); ^{13}C NMR (100 MHz, $CDCl_3$): δ 196.8, 137.6, 132.4, 130.1, 128.3 ppm; mp: 48.3 - 50.2 ℃.

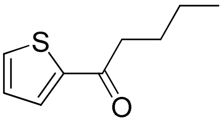

1-(Thiophen-2-yl)pentan-1-one (4e): Following the *general procedure A*, **4e** was isolated as a yellow liquid (31.7 mg, 76%), known compound. The NMR spectroscopic data agree with those described in ref.[S23]. ^{1}H NMR (400 MHz, $CDCl_3$): δ 7.73 (d, *J* = 3.60 Hz, 1H), 7.64 (d, *J* = 5.2 Hz, 1H), 7.14 (t, *J* = 3.60 Hz, 1H), 2.92 (t, *J* = 7.60 Hz, 2H), 1.79 (quint, *J* = 7.60 Hz, 2H),

1.43 (sext, J = 7.60 Hz, 2H), 0.98 ppm (t, J = 7.20 Hz, 3H); ^{13}C NMR (100 MHz, $CDCl_3$): δ 193.6, 144.5, 133.3, 131.6, 128.0, 39.1, 26.9, 22.5, 13.9 ppm.

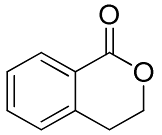

Isochroman-1-one (4f): Following the *general procedure A*, **4f** was isolated as a yellow liquid (27.6 mg, 75%), known compound. The NMR spectroscopic data agree with those described in ref.[S24]. ^{1}H NMR (400 MHz, $CDCl_3$): δ 8.12 (d, J = 8.0 Hz, 1H), 7.56 (td, J= 7.60, 1.20 Hz, 1H), 7.41 (t, J = 7.60 Hz, 1H), 7.28 (d, J = 7.60 Hz, 1H), 4.56 (t, J = 6.0 Hz, 2H), 3.08 ppm (t, J = 6.0 Hz, 2H); ^{13}C NMR (100 MHz, $CDCl_3$): δ 165.1, 139.5, 133.6, 130.4, 127.7, 127.2, 125.3, 67.3, 27.8 ppm.

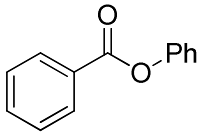

Phenyl benzoate (4g): Following the *general procedure A* except with $K_2S_2O_8$ (204.9 mg, 0.75 mmol), **4g** was isolated as a white solid (35.5 mg, 72%), known compound. The NMR spectroscopic data agree with those described in ref.[S25]. ^{1}H NMR (400 MHz, $CDCl_3$): δ 8.26 (dd, J = 8.40, 1.20 Hz, 2H), 7.68 (tt, J = 7.20, 1.20 Hz, 1H), 7.55 (t, J = 8.0 Hz, 2H), 7.48 (t, J = 8.4 Hz, 2H), 7.34 - 7.30 (m, 1H), 7.27 ppm (d, J = 8.40 Hz, 2H); ^{13}C NMR (100 MHz, $CDCl_3$): δ 165.1, 150.9, 133.5, 130.1, 129.5, 129.4, 128.5, 125.8, 121.6 ppm; mp: 69.4 - 70.5 ℃.

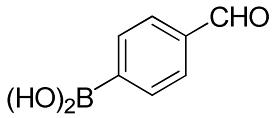

(4-Formylphenyl)boronic acid (6a): Following the *general procedure B*, **6a** was isolated as a white solid (30.8 mg, 82%), known compound. The NMR spectroscopic data agree those described in ref.[26]. ^{1}H NMR (400 MHz, acetone-d_6): δ 10.08 (s, 1 H), 8.06 (d, J = 8.1 Hz, 2 H), 7.90 (d, J = 8.1 Hz, 2 H), 7.46 ppm (s, 2 H); ^{13}C NMR (100 MHz, acetone-d_6): δ 193.3, 138.8, 135.5, 129.2; ^{11}B NMR (128 MHz, acetone-d_6): δ 28.5 ppm; Mp: 202.1-203.0 ℃.

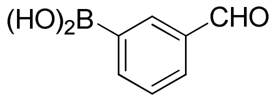

(3-Formylphenyl)boronic acid (6b): Following the *general procedure B*, **6b** was isolated as a white solid (29.6 mg, 79%), known compound (CAS: 87199-16-4). ^{1}H NMR (400 MHz, acetone-d_6): δ 10.07 (s, 1 H), 8.40 (s, 1 H), 8.17 (dt, J = 7.4, 1.2 Hz, 1 H), 7.97 (dt, J = 7.7, 1.5 Hz, 1 H), 7.59 (t, J = 7.5 Hz, 1 H), 7.44 ppm (s, 2 H); ^{13}C NMR (100 MHz, acetone-d_6): δ 193.3, 140.8, 137.0, 136.4, 131.8, 129.2; ^{11}B NMR (128 MHz, acetone-d_6): δ 28.6 ppm; Mp: 168.9-169.7 ℃.

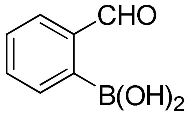

(2-Formylphenyl)boronic acid (6c): Following the *general procedure B*, **6c** was isolated as a white solid (31.5 mg, 84%),

known compound. The NMR spectroscopic data agree those described in ref.[26]. ^{1}H NMR (400 MHz, acetone-d_6): δ 10.21 (s, 1 H), 7.97 (dd, J = 7.2, 1.6 Hz, 1 H), 7.89 (dd, J = 7.2, 1.6 Hz, 1 H), 7.73 (s, 2 H), 7.68-7.60 ppm (m, 1 H); ^{13}C NMR (100 MHz, acetone-d_6): δ 196.3, 141.1, 135.8, 134.0, 132.7, 130.5; ^{11}B NMR (128 MHz, acetone-d_6): δ 29.5 ppm; Mp: 112.7-113.5 ℃.

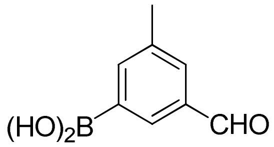

(3-Formyl-5-methylphenyl)boronic acid (6d): Following the *general procedure B*, **6d** was isolated as a white solid (33.2 mg, 81%), known compound (CAS: 870777-33-6). ^{1}H NMR (400 MHz, acetone-d_6): δ 10.02 (s, 1 H), 8.19 (s, 1 H), 7.99 (s, 1 H), 7.76 (s, 1H), 7.39 (s, 2 H), 2.43 ppm (s, 3 H); ^{13}C NMR (100 MHz, acetone-d_6): δ 193.4, 141.7, 138.7, 137.2, 133.9, 132.0, 29.3; ^{11}B NMR (128 MHz, acetone-d_6): δ 28.9 ppm; Mp: 215.4-216.3 ℃.

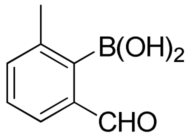

(2-Formyl-6-methylphenyl)boronic acid (6e): Following the *general procedure B*, **6e** was isolated as a yellow solid (32.0 mg, 78%), unknown compound. ^{1}H NMR (400 MHz, acetone-d_6): δ 9.98 (s, 1 H), 7.67 (dd, J = 6.9, 1.0 Hz, 1 H), 7.44-7.38 (m, 2 H), 7.09 (s, 2 H), 2.42 ppm(s, 3 H); ^{13}C NMR (100 MHz, acetone-d_6): δ 194.1, 141.2, 139.9, 135.4, 129.3, 128.9, 21.5 ppm; HRMS (ESI) calcd. for $C_8H_9BO_3Na^+$ [M + Na^+] m/z 187.05370, found m/z 187.05315; IR (KBr, cm^{-1}): ν_{max} 3312, 2964, 2918, 2854, 1683, 1601, 1535, 1345, 784; ^{11}B NMR (128 MHz, acetone-d_6): δ 30.5 ppm; Mp: 103.2-104.1 ℃.

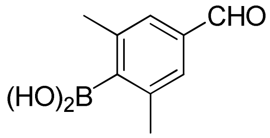

(4-Formyl-2,6-dimethylphenyl)boronic acid (6f): Following the *general procedure B*, **6f** was isolated as a white solid (32.1 mg, 72%), known compound (CAS: 1228829-13-7). ^{1}H NMR (400 MHz, acetone-d_6): δ 9.93 (s, 1 H), 7.50 (s, 2 H), 7.45 (s, 2 H), 2.41 ppm (s, 6 H); ^{13}C NMR (100 MHz, acetone-d_6): δ 193.2, 140.8, 137.3, 127.6, 22.0; ^{11}B NMR (128 MHz, acetone-d_6): δ 30.7 ppm; Mp: 106.4-107.3 ℃.

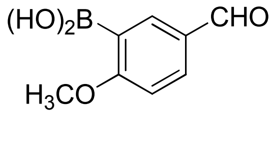

(5-Formyl-2-methoxyphenyl)boronic acid (6g): Following the *general procedure B*, **6g** was isolated as a yellow solid (34.2 mg, 76%), known compound. The NMR spectroscopic data agree those described in ref.[27]. ^{1}H NMR (400 MHz, acetone-d_6): δ 9.94 (s, 1 H), 8.35 (d, J = 2.2 Hz, 1 H), 8.0 (dd, J = 8.8, 2.2 Hz, 1 H), 7.24 (d, J = 8.8 Hz, 1 H), 7.15 (s, 2 H), 4.05 ppm (s, 3 H); ^{13}C NMR (100 MHz, acetone-d_6): δ 191.6, 170.0, 139.6, 134.9, 131.0, 111.7, 56.6; ^{11}B NMR (128 MHz, acetone-d_6): δ 28.6

ppm; Mp: 125.6-126.5 ℃.

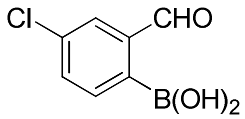

(4-Chloro-2-formylphenyl)boronic acid (6h): Following the *general procedure B*, **6h** was isolated as a white solid (34.1 mg, 74%), known compound (CAS: 913835-76-4). ^{1}H NMR (400 MHz, acetone-d_6): δ 10.29 (s, 1 H), 7.93 (d, J = 2.2 Hz, 1 H), 7.86 (d, J = 8.0 Hz, 1 H), 7.76 (s, 2H), 7.67 ppm (dd, J = 8.0, 2.2 Hz, 1 H); ^{13}C NMR (100 MHz, acetone-d_6): δ 194.5, 142.9, 137.3, 136.2, 133.5, 130.5; ^{11}B NMR (128 MHz, acetone-d_6): δ 29.4 ppm; Mp: 120.1-121.0 ℃.

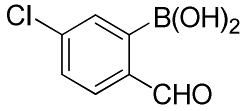

(5-Chloro-2-formylphenyl)boronic acid (6i): Following the *general procedure B*, **6i** was isolated as a yellow solid (34.5 mg, 75%), known compound (CAS: 870238-36-1). ^{1}H NMR (400 MHz, acetone-d_6): δ 10.22 (s, 1 H), 7.97 (d, J = 8.2 Hz, 1 H), 7.82 (s, 2 H), 7.81 (d, J = 2.2 Hz, 1 H), 7.64 ppm (dd, J = 8.2, 2.2 Hz, 1 H); ^{13}C NMR (100 MHz, acetone-d_6): δ 194.7, 140.1, 139.5, 135.2, 133.8, 130.4; ^{11}B NMR (128 MHz, acetone-d_6): δ 29.1 ppm, Mp: 117.3-178.2 ℃.

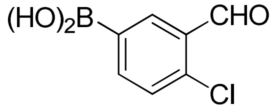

(4-Chloro-3-formylphenyl)boronic acid (6j): Following the *general procedure B*, **6j** was isolated as a yellow solid (35.5 mg, 77%), unknown compound. ^{1}H NMR (400 MHz, acetone-d_6): δ 10.47 (s, 1 H), 8.37 (d, J = 1.7 Hz, 1 H), 8.10 (dd, J = 8.0, 1.7 Hz, 1 H), 7.57 (d, J = 8.0 Hz, 1 H), 7.57 (s, 2 H); ^{13}C NMR (100 MHz, acetone-d_6): δ 190.2, 141.8, 139.9, 136.1, 132.7, 130.8; ^{11}B NMR (128 MHz, acetone-d_6): δ 28.2 ppm; HRMS (ESI) calcd. for $C_7H_6BClO_3Na^+$ [M + Na$^+$] m/z 206.99907, found m/z 206.99994; IR (KBr, cm^{-1}): ν_{max} 3566, 2924, 2853, 1697, 1591, 1507, 1339, 749; Mp: 98.7-99.6 ℃.

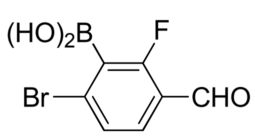

(6-Bromo-2-fluoro-3-formylphenyl)boronic acid (6k): Following the *general procedure B*, **6k** was isolated as a yellow solid (49.2 mg, 80%), known compound (CAS: 1315340-55-6). ^{1}H NMR (400 MHz, acetone-d_6): δ 10.26 (s, 1 H), 8.05 (s, 2 H), 7.71 (d, J = 8.2 Hz, 1 H), 7.56 ppm (d, J = 8.2 Hz, 1 H); ^{13}C NMR (100 MHz, acetone-d_6): δ 187.2 (d, J = 5.8 Hz), 165.9 (d, J = 253.5 Hz), 132.8 (d, J = 13.3 Hz), 130.3 (d, J = 2.7 Hz), 129.5 (d, J = 3.5 Hz), 123.4 ppm (d, J = 10.9 Hz); ^{19}F NMR (376 MHz, acetone-d_6): δ -113.4; ^{11}B NMR (128 MHz, acetone-d_6): δ 28.8 ppm; Mp: 119.5-120.4 ℃.

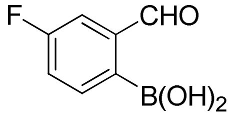

(4-Fluoro-2-formylphenyl)boronic acid (6l): Following the *general procedure B*, **6l** was isolated as a yellow solid (29.8 mg,

71%), known compound. The NMR spectroscopic data agree those described in ref.[28]. ^{1}H NMR (400 MHz, acetone-d_6): δ 10.33 (s, 1 H), 7.95-7.92 (m, 1H), 7.75 (s, 2 H), 7.66 (dd, J = 9.6, 2.4 Hz, 1 H), 7.43 ppm (td, J = 8.8, 2.4 Hz, 1 H); ^{13}C NMR (100 MHz, acetone-d_6): δ 194.6 (d, J = 1.5 Hz), 164.6 (d, J = 246.8 Hz), 143.8 (d, J = 5.6 Hz), 138.2 (d, J = 7.2 Hz), 120.6 (d, J = 20.3 Hz), 116.8 ppm (d, J = 22.4 Hz); ^{19}F NMR (376 MHz, acetone-d_6): δ -112.7 ppm; ^{11}B NMR (128 MHz, acetone-d_6): δ 29.2 ppm; Mp: 113.2-114.1 ℃.

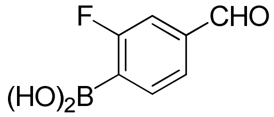

(2-Fluoro-4-formylphenyl)boronic acid (6m): Following the *general procedure B*, **6m** was isolated as a yellow solid (31.9 mg, 76%), known compound (CAS: 871126-22-6). ^{1}H NMR (400 MHz, acetone-d_6): δ 10.06 (s, 1 H), 7.92 (m, 1H), 7.75 (m, 1 H), 7.57 (s, 2 H), 7.54 ppm (d, J = 1.2 Hz, 1 H); ^{13}C NMR (100 MHz, acetone-d_6): δ 192.1 (d, J = 2.1 Hz), 167.6 (d, J = 245.3 Hz), 140.5 (d, J = 7.0 Hz), 137.6 (d, J = 8.8 Hz), 125.8 (d, J = 2.8 Hz), 115.4 ppm (d, J = 25.6 Hz); ^{19}F NMR (376 MHz, acetone-d_6): δ -105.8 ppm; ^{11}B NMR (128 MHz, acetone-d_6): δ 28.4 ppm; Mp: 141.7-142.5 ℃.

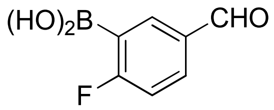

(2-Fluoro-5-formylphenyl)boronic acid (6n): Following the *general procedure B*, **6n** was isolated as a yellow solid (33.6 mg, 80%), known compound (CAS: 352534-79-3). ^{1}H NMR (400 MHz, acetone-d_6): δ 10.03 (s, 1 H), 8.31 (dd, J = 6.0, 2.4 Hz, 1 H), 8.03 (m, 1 H), 7.53 (s, 2 H), 7.29 ppm (t, J = 8.9 Hz, 1 H); ^{13}C NMR (100 MHz, acetone-d_6): δ 191.7, 171.0 (d, J = 253.1 Hz), 139.5 (d, J = 10.7 Hz), 134.3 (d, J = 10.8 Hz), 133.9 (d, J = 2.7 Hz), 116.9 ppm (d, J = 26.1 Hz); ^{19}F NMR (376 MHz, acetone-d_6): δ -97.1 ppm; ^{11}B NMR (128 MHz, acetone-d_6): δ 28.0 ppm; Mp: 147.2-148.1 ℃.

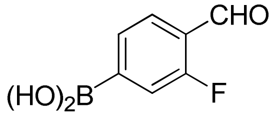

(3-Fluoro-4-formylphenyl)boronic acid (6o): Following the *general procedure B*, **6o** was isolated as a yellow solid (30.7 mg, 73%), known compound. (CAS: 248270-25-9). ^{1}H NMR (400 MHz, acetone-d_6): δ 10.36 (s, 1 H), 7.83 (m, 2 H), 7.69 (d, J = 11.4 Hz, 1 H), 7.63 ppm (s, 2 H); ^{13}C NMR (100 MHz, acetone-d_6): δ 187.7 (d, J = 6.1 Hz), 166.7 (d, J = 258.1 Hz), 143.3 (d, J = 9.4 Hz), 136.1 (d, J = 1.7 Hz), 124.5 (d, J = 7.1 Hz), 116.6 ppm (d, J = 19.4 Hz); ^{19}F NMR (376 MHz, acetone-d_6): δ -125.2 ppm; ^{11}B NMR (128 MHz, acetone-d_6): δ 27.8 ppm; Mp: 203.1-204.0 ℃.

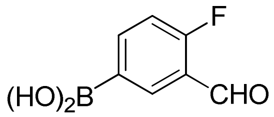

(4-Fluoro-3-formylphenyl)boronic acid (6p): Following the *general procedure B*, **6p** was isolated as a yellow solid (28.6 mg, 68%), known compound (CAS: 374538-01-9). ^{1}H NMR (400 MHz, acetone-d_6): δ 10.34 (s, 1 H), 8.36 (m, 1 H), 8.19 (m, 1 H),

7.47 (s, 2 H), 7.30 ppm (m, 1 H); ^{13}C NMR (100 MHz, acetone-d_6): δ 187.9 (d, J = 6.1 Hz), 166.7 (d, J = 258.1 Hz), 143.3 (d, J = 9.3 Hz), 136.1 (d, J = 1.8 Hz), 124.5 (d, J = 7.1 Hz), 116.6 ppm (d, J = 19.3 Hz); ^{19}F NMR (376 MHz, acetone-d_6): δ -121.2 ppm; ^{11}B NMR (128 MHz, acetone-d_6): δ 28.1 ppm; Mp: 132.1-134.2 ℃.

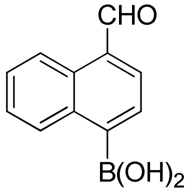

(4-Formylnaphthalen-1-yl)boronic acid (6q): Following the *general procedure B*, **6q** was isolated as a yellow solid (30.0 mg, 60%), known compound. The NMR spectroscopic data agree those described in ref.[29]. ^{1}H NMR (400 MHz, acetone-d_6): δ 10.43 (s, 1 H), 9.28 (d, J = 8.4 Hz, 1 H), 8.54 (d, J = 8.4 Hz, 1 H), 8.10 (d, J = 7.0 Hz, 1 H), 8.01 (d, J = 7.0 Hz, 1 H), 7,78 (s, 2 H), 7.66 ppm (m, 2 H); ^{13}C NMR (100 MHz, acetone-d_6): δ 194.6, 137.1, 136.4, 132.9, 131.6, 130.9, 130.3, 129.0, 127.4, 125.6; ^{11}B NMR (128 MHz, acetone-d_6): δ 29.9 ppm; Mp: 199.7-200.6 ℃.

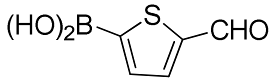

(5-Formylthiophen-2-yl)boronic acid (6r): Following the *general procedure B*, **6r** was isolated as a yellow solid (29.6 mg, 76%), known compound. The NMR spectroscopic data agree those described in ref.[26]. ^{1}H NMR (400 MHz, acetone-d_6): δ 10.01 (s, 1 H), 7.96 (d, J = 3.6 Hz, 1 H), 7.77 (d, J = 3.6 Hz, 1 H), 7.74 ppm (s, 2 H); ^{13}C NMR (100 MHz, acetone-d_6): δ 184.2, 149.3, 137.8, 137.1; ^{11}B NMR (128 MHz, acetone-d_6): δ 26.6 ppm; Mp: 115.8.1-116.8 ℃.

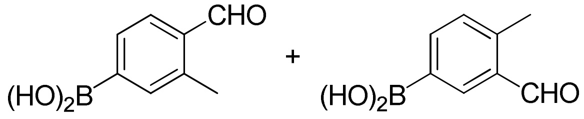

1 : 1

(4-Formyl-3-methylphenyl)boronic acid and (3-formyl-4-methylphenyl)boronic acid (6s:6s' = 1:1): Following the *general procedure B*, **6s** and **6s'** were isolated as a white solid (30.4 mg, 74%, 1:1 mixture), known compound (CAS: 398151-59-2 and 1106869-99-1). ^{1}H NMR (400 MHz, acetone-d_6): δ 10.31 (s, 1 H), 10.29 (s, 1 H), 8.31 (s, 1 H), 8.00 (dd, J = 7.5, 1.2 Hz, 1 H), 7.87 (d, J = 7.6 Hz, 1 H), 7.80 (m, 2 H), 7.42 (s, 2 H), 7.36 (s, 2 H), 7.33 (d, J = 7.6 Hz, 1 H), 2.67 (s, 3 H), 2.66 ppm (s, 3 H); ^{13}C NMR (100 MHz, acetone-d_6): δ 193.8, 193.6, 143.1, 140.0, 139.6, 139.0, 138.3, 136.4, 134.7, 132.7, 131.9, 131.3, 19.8, 19.6; ^{11}B NMR (128 MHz, acetone-d_6): δ 28.8, 28.3 ppm; Mp: 178.1-179.0 ℃.

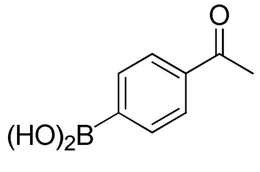

(4-Acetylphenyl)boronic acid (6u): Following the *general procedure B*, **6u** was isolated as a yellow solid (36.5 mg, 89%),

known compound. The NMR spectroscopic data agree those described in ref.[30]. ^{1}H NMR (400 MHz, acetone-d_6): δ 7.99 (d, *J* = 8.4 Hz, 2 H), 7.95 (d, *J* = 8.4 Hz, 2 H), 7.40 (s, 2 H), 2.59 ppm (s, 3 H); ^{13}C NMR (100 MHz, acetone-d_6): δ 198.3, 139.4, 135.1, 127.9, 26.8; ^{11}B NMR (128 MHz, acetone-d_6): δ 28.7ppm; Mp: 222.5-223.3 ℃.

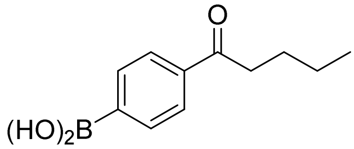

(4-Pentanoylphenyl)boronic acid (6v): Following the *general procedure B*, **6v** was isolated as a yellow solid (37.1 mg, 72%), known compound (CAS: 1106837-79-9). ^{1}H NMR (400 MHz, acetone-d_6): δ 7.99 (d, *J* = 8.4 Hz, 2 H), 7.96 (d, *J* = 8.4 Hz, 2 H), 7,37 (s, 2 H), 3.03 (t, *J* = 7.2 Hz, 2 H), 1.68 (quint, *J* = 7.3 Hz, 2 H), 1.41 (sext, *J* = 7.4 Hz, 2 H), 0.94 ppm (t, *J* = 7.4 Hz, 3 H); ^{13}C NMR (100 MHz, acetone-d_6): δ 200.6, 139.5, 135.1, 127.7, 38.8, 27.1, 23.0, 14.2; ^{11}B NMR (128 MHz, acetone-d_6): δ 28.7 ppm; Mp: 91.1-92.0 ℃.

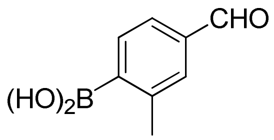

(4-Formyl-2-methylphenyl)boronic acid (6w): Following the *general procedure B*, **6w** was isolated as a white solid (20.5 mg, 50%), known compound (CAS: 156428-81-8). ^{1}H NMR (400 MHz, acetone-d_6): δ 10.18 (s, 1 H), 7.86 (d, *J* = 7.6 Hz, 1 H), 7.84 (s, 2 H), 7.79 (s, 1 H), 7.49 (d, *J* = 7.6 Hz, 1 H), 2.43 ppm (s, 3 H); ^{13}C NMR (100 MHz, acetone-d_6): δ 197.0, 141.3, 140.8, 136.6, 134.8, 133.9, 21.1; ^{11}B NMR (128 MHz, acetone-d_6): δ 29.8 ppm; Mp: 70.3-71.2 ℃.

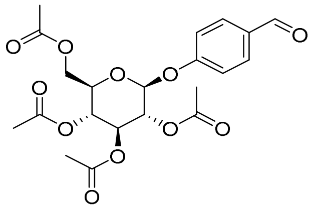

1-*O*-(4'-Formylphenyl)-β-D-tetraacetylglucoside (8a): Following the *general procedure A*, **8a** was isolated as a yellow oil (47.2 mg, 42%), unknown compound. ^{1}H NMR (400 MHz, $CDCl_3$): δ 9.94 (s, 1H), 7.87 (d, *J* = 8.80 Hz, 2H), 7.11 (d, *J* = 8.80 Hz, 2H), 5.36 - 5.30 (m, 2H), 5.24 - 5.17 (m, 2H), 4.28 (d, *J* = 5.6 Hz, 1H), 4.21 - 4.18 (m, 1H), 3.97 - 3.92 (m, 1H), 2.08 (s, 3H), 2.07(s, 3H), 2.071(s, 3H), 2.06 ppm (s, 3H); ^{13}C NMR (100 MHz, $CDCl_3$): δ 190.7, 170.5, 170.2, 169.4, 169.2, 161.2, 131.8, 131.7, 116.7, 98.0, 71.5, 72.3, 70.9, 68.1, 61.8, 20.6, 20.5, 20.5, 20.5 ppm; HRMS (ESI) calcd. for $[M + Na]^+$ *m/z* 475.1211, found *m/z* 475.1239; IR (KBr, cm^{-1}): ν_{max} 2970, 2846, 1747, 1595, 1389, 1237, 1039, 843, 619 cm^{-1}.

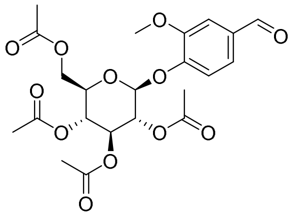

1-*O*-(2'-Methoxy-4'-formylphenyl)-β-D-tetraacetylglucoside (8b): Following the *general procedure A*, **8b** was isolated as a white solid (65.0 mg, 54%), unknown compound. ^{1}H NMR (400 MHz, $CDCl_3$): δ 9.85 (s, 1H), 7.39 (dd, *J* = 8.40, 1.60 Hz, 1H), 7.38 (d, *J* = 1.60 Hz, 1H), 7.20 (d, *J* = 8.40 Hz, 1H), 5.31 - 5.26 (m, 2H), 5.18 - 5.08 (m, 2H), 4.25 (dd, *J* = 12.40, 5.2 Hz, 1H), 4.16 (d, *J* = 12.40 Hz, 1H), 3.86 (s, 3H), 3.82 (m, 1H), 2.05 (s, 3H), 2.04 (s, 3H), 2.02 (s, 3H), 2.021 ppm (s, 3H); ^{13}C NMR (100 MHz, $CDCl_3$): δ 190.8, 170.4, 170.1, 169.3, 169.1, 151.0, 150.9, 132.7, 125.3, 118.1, 110.7, 99.6, 72.3, 72.2, 70.9, 68.2, 61.8, 56.0, 20.6, 20.5, 20.5, 20.4 ppm; HRMS (ESI) calcd. for $[M + Na]^+$ *m/z* 505.1400, found *m/z* 505.1317; IR (KBr, cm^{-1}): ν_{max} 2970, 2880, 1763, 1585, 1379, 1209, 1049, 905, 727 cm^{-1}; mp: 92.2 - 94.5 ℃.

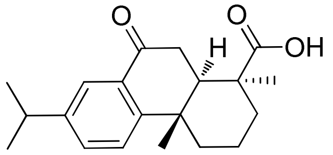

(1S,4aS,10aR)-7-acetyl-1,4a-dimethyl-9-oxo-1,2,3,4,4a,9,10,10a-octahydrophenanthrene-1-carboxylic acid (8c): Following the *general procedure A*, **8c** was isolated as colorless oil (51.0 mg, 65%), known compound. The NMR spectroscopic data agree with those described in ref.[S31]. ^{1}H NMR (400 MHz, $CDCl_3$): δ 7.89 (s, 1H), 7.43 (d, *J* = 8.15 Hz, 1H), 7.31 (d, *J* = 8.15 Hz, 1H), 2.94 (hept, *J* = 6.80 Hz, 1H), 2.81 - 2.59 (m, 2H), 2.50 (d, *J* = 15.20 Hz, 1H), 2.39 (d, *J* = 12.80 Hz, 1H), 1.88 - 1.80 (m, 4H), 1.70 - 1.65 (m, 1H), 1.36 (s, 3H), 1.28 (s, 3H), 1.25 ppm (d, *J* = 6.80 Hz, 6H); ^{13}C NMR (100 MHz, $CDCl_3$): δ 198.9, 183.3, 152.9, 146.9, 132.7, 130.5, 125.1, 123.5, 46.3, 43.5, 37.7, 37.2, 36.9, 36.4, 33.6, 23.8, 23.7, 23.6, 18.0, 16.1 ppm; mp: 182.6 - 184.5 ℃.

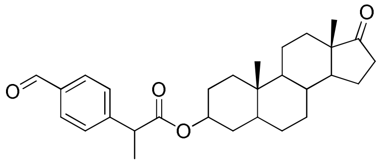

(10S,13S)-10,13-dimethyl-17-oxohexadecahydro-1H-cyclopenta[a]phenanthren-3-yl-2-(4-formylphenyl)propanoate (8d): Following the *general procedure A*, **8d** was isolated as colorless oil (67.5 mg, 60%), unknown compound. ^{1}H NMR (400 MHz, $CDCl_3$): δ 9.99 (s, 1H), 7.84 (d, *J* = 8.18 Hz, 2H), 7.46 (d, *J* = 8.18 Hz, 2H), 4.70 (m, 1H), 3.75 (q, *J* = 7.20 Hz, 1H), 2.43 (dd, *J* = 19.20 Hz, 8.80 Hz, 1H), 2.10 - 2.01 (m, 1H), 1.95 - 1.88 (m, 1H), 1.79 - 1.75 (m, 3H), 1.70 - 1.66 (m, 2H), 1.63 - 1.57 (m, 2H), 1.51 (d, *J* = 7.20 Hz, 3H), 1.48 - 1.42 (m, 2H), 1.31 - 1.27 (m, 6H), 1.18 - 1.15 (m, 1H), 1.06 - 0.93 (m, 2H), 0.85 (s, 3H), 0.81 (s, 3H), 0.72 - 0.66 ppm (m, 1H); ^{13}C NMR (100 MHz, $CDCl_3$): δ 221.7, 192.4, 173.4, 135.3, 130.0, 128.2, 125.0, 74.2, 54.3, 51.3, 47.8, 45.9, 44.6, 36.7, 35.8, 35.6, 34.9, 33.8, 31.5, 30.7, 28.2, 27.1, 21.7, 20.5, 18.4, 13.9, 12.2 ppm; HRMS (ESI) calcd. for $[M + Na]^+$ *m/z* 473.2662, found *m/z* 473.2677; IR (KBr, cm^{-1}): ν_{max} 2898, 2836, 1629, 1397, 1129, 843, 681 cm^{-1}.

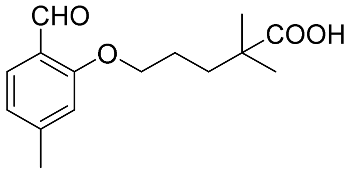

5-(2-Formyl-5-methylphenoxy)-2,2-dimethylpentanoic acid (8e): Following the *general procedure A*, **8e** was isolated as

light yellow oil (60.5 mg, 92%). known compound. The NMR spectroscopic data agree with those described in ref.[S32]. ^{1}H NMR (400 MHz, $CDCl_3$): δ 10.43 (s, 1H), 7.72 (d, *J* = 7.6 Hz, 1H), 6.82 (d, *J* = 7.6 Hz, 1H), 6.74 (s, 1H), 4.05 (t, *J* = 6.0 Hz, 2H), 2.38 (s, 3H), 1.86-1.82 (m, 2H), 1.77-1.73 (m, 2H), 1.26 ppm (s, 6H); ^{13}C NMR (100 MHz, $CDCl_3$): δ 189.6, 183.6, 161.4, 147.4, 128.2, 122.5, 121.6, 112.9, 68.4, 41.9, 36.6, 25.0, 24.9, 22.3 ppm. According to analysis of the ^{1}H NMR spectroscopy, the regioselectivity of **8e** is more than 95%.

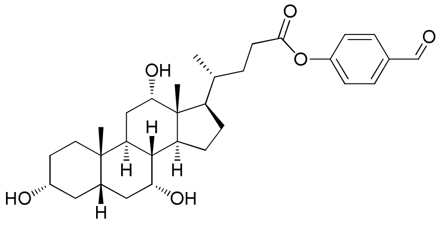

(R)-4-Formylphenyl-4-((3R,5S,7R,8R,9S,10S,12S,13R,14S,17R)-3,7,12-trihydroxy-10,13-dimethylhexadecahydro-1H-cyclopenta[a]phenanthren-17-yl)pentanoate (8f): Following the *general procedure A*, **8f** was isolated as a white solid (70.3 mg, 55%), unknown compound. ^{1}H NMR (400 MHz, $CDCl_3$): δ 9.97 (s, 1H), 7.91 (d, *J* = 8.20 Hz, 2H), 7.26 (d, *J* = 8.20 Hz, 2H), 3.96 (s, 1H), 3.83 (s, 1H), 3.56 (s, 3H), 3.43 (s, 1H), 2.67 - 2.47 (m, 2H), 2.24 (m, 2H), 2.03 - 1.24 (m, 20H), 1.10 - 0.95 (m, 3H), 0.87 (s, 3H), 0.68 ppm (s, 3H); ^{13}C NMR (100 MHz, $CDCl_3$): δ 190.9, 172.0, 155.4, 133.7, 131.1, 122.2, 99.9, 72.9, 71.7, 68.3, 46.8, 46.3, 41.5, 41.4, 39.3, 39.2, 35.2, 34.7, 34.6, 31.3, 30.7, 30.2, 28.1, 27.5, 26.2, 23.1, 22.3, 17.2, 12.4 ppm; HRMS (ESI) calcd. for $[M + Na]^+$ *m/z* 535.3030, found *m/z* 535.3048; IR (KBr, cm^{-1}): ν_{max} 3426, 2934, 1629, 1407, 1111, 735 cm^{-1}; mp: 207.2 - 208.5 ℃.

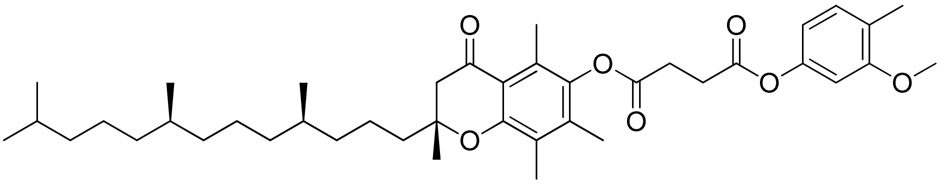

3-Methoxy-4-methylphenyl((R)-2,5,7,8-tetramethyl-4-oxo-2-((4R,8R)-4,8,12-trimethyltridecyl)chroman-6-yl) succinate (**8g**): Following the *general procedure A* except with $K_2S_2O_8$ (204.9 mg, 0.75 mmol), **8g** was isolated asawhite solid (94.5 mg, 57%), unknown compound. ^{1}H NMR (400 MHz, $CDCl_3$): δ 6.90 (d, *J* = 8.0 Hz, 1H), 6.77 (s, 1H), 6.73 (d, *J* = 8.0 Hz, 1H), 3.79 (s, 3H), 3.05 (m, 6H), 2.39 (s, 3H), 2.34 (s, 3H), 2.13 (s, 3H), 2.08 (s, 3H), 2.02 (m, 2H), 1.52 (m, 2H), 1.36 (s, 4H), 1.26 (m, 12H), 1.15 (m, 4H), 0.86 ppm (d, *J* = 6.6 Hz, 12H) ; ^{13}C NMR (100 MHz, $CDCl_3$): δ 194.5, 192.5, 170.6, 170.5, 156.4, 150.5, 141.6, 137.0, 136.9, 129.9, 128.9, 124.3, 123.6, 122.3, 121.2, 113.3, 80.1, 55.8, 39.4, 37.4, 37.3, 37.1, 32.8, 29.8, 29.6, 29.3, 28.84, 28.82, 28.0, 27.2, 25.5, 24.8, 24.4, 22.7, 22.6, 21.4, 19.7, 19.6, 14.1, 14.0, 13.9, 12.1 ppm; HRMS (ESI) calcd. for $C_{41}H_{61}O_7^+$ $[M+H]^+$ *m/z* 665.44118, found *m/z* 665.44110; IR (KBr, cm^{-1}): ν_{max} 3566, 2922, 2850, 1760, 1681, 1646, 1507, 1457, 1417, 1374, 1287, 1269, 1201, 1131, 768; mp: 121-122℃.

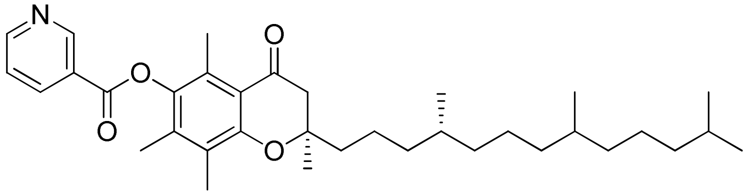

(*2R*)-2,5,7,8-Tetramethyl-4-oxo-2-((*4R*)-4,8,12-trimethyltridecyl)chroman-6-ylnicotinate (8h): Following the *general procedure A* except with $K_2S_2O_8$ (204.9 mg, 0.75 mmol), **6g** was isolated as a brown solid (52.1 mg, 38%), unknown compound. ^{1}H NMR (400 MHz, $CDCl_3$) δ: 9.48 (s, 1H), 8.93 (d, *J* = 4.0 H, 1H), 8.65 (d, *J* = 8.0 Hz, 1H), 7.67-7.64 (m, 1H), 2.77 (t, *J* = 14.2 Hz, 1H), 2.61 (t, *J* = 15.4 Hz, 1H), 2.43 (s, 3H), 2.17 (s, 3H), 2.12 (s, 3H), 1.55-1.46 (m, 2H), 1.40-1.25 (m, 16H),1.15-1.07 (m, 6H), 0.87-0.83 ppm (m, 12H); ^{13}C NMR (100 MHz, $CDCl_3$) δ: 194.4, 162.6, 156.7, 151.5, 148.9, 141.2, 140.1, 136.5, 128.8, 126.2, 124.72, 124.65, 116.9, 80.3, 39.3, 37.4, 37.3, 32.8, 32.6, 28.0, 24.8, 24.4, 23.6, 22.7, 22.6, 21.0, 19.73, 19.66, 19.59, 19.57, 19.52, 19.50, 14.12, 14.03, 12.2 ppm; IR (KBr): 3364, 2922, 2850, 1744, 1682, 1645, 1591, 1460, 1418, 1378, 1266, 1098, 736; HRMS (ESI) m/z calcd for $C_{35}H_{51}NO_4^+$ $(M+H)^+$ 550.3891, found 550.3895; mp: 101.3-102.2℃.

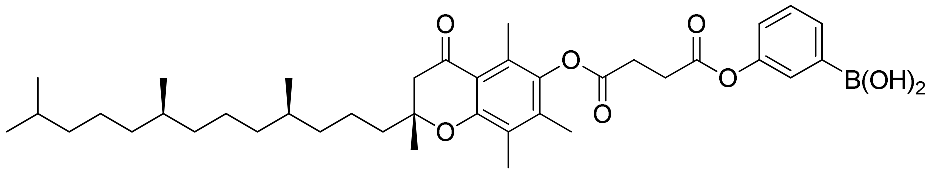

(3-((4-Oxo-4-(((R)-2,5,7,8-tetramethyl-4-oxo-2-((4R,8R)-4,8,12-trimethyltridecyl)chroman-6-yl)oxy)butanoyl)oxy)phenyl)boronic acid (8i): Following the *general procedure B* except with O_2 (1 atm) as the atmosphere and 90 °C as the reaction temperature, **8i** was isolated as a yellow solid (79.7 mg, 48%), unknown compound. ^{1}H NMR (400 MHz, acetone-*d*$_6$): δ 7.75 (d, *J* = 7.4 Hz, 1 H), 7.57 (s, 1 H), 7.39 (t, *J* = 8.0 Hz, 1 H), 7.30 (s, 2 H), 7.16 (d, *J* = 8.0 Hz, 1 H), 3.08 (m, 6 H), 2.37 (s, 3 H), 2.16 (s, 3 H), 2.10 (s, 3 H), 2.0 (m, 1 H), 1.96 (m, 1 H), 1.52 (m, 2 H), 1.39 (s, 4 H), 1.32 (m, 11 H), 1.15 (m, 5 H), 0,87 (m, 12 H); ^{13}C NMR (100 MHz, acetonitrile-*d*$_3$): δ 195.1, 172.3, 172.0, 157.0, 151.4, 142.7, 138.0, 132.4, 129.9, 129.6, 127.8, 126.4, 125.3, 124.7, 81.1, 40.0, 38.0, 37.9, 33.5, 30.3, 29.9, 29.8, 29.5, 29.4, 28.7, 25.5, 25.0, 23.0, 22.9, 20.1, 20.0, 14.3, 14.2, 12.3; ^{11}B NMR (128 MHz, acetone-*d*$_6$): δ 28.5 ppm; HRMS (ESI) calcd. for $C_{39}H_{58}BO_8H^+$ [M + H$^+$] m/z 665.42193, found m/z 665.42145; IR (KBr, cm^{-1}): ν_{max} 3446, 2926, 2867, 1759, 1682, 1601, 1575, 1456, 1428, 1361, 1134, 905, 771, 731, 706, 583; Mp: 102.3-103.1 ℃.

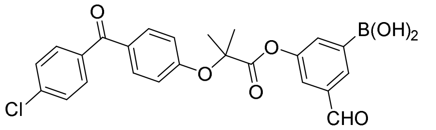

(3-((2-(4-(4-Chlorobenzoyl)phenoxy)-2-methylpropanoyl)oxy)-5-formylphenyl)boronic acid (8j). The reaction was conducted under the normal conditions *as the general procedure B* except with O_2 (1 atm) as the atmosphere and 90 °C as the reaction temperature to provide the title product in 40% yield. During work-up and purification, we observed the product was decomposed easily on silica gel. Therefore, the yield of the product was determined by ^{1}H NMR using nitromethane as an internal standard. And the characterization of the product was analyzed by HRMS shown in the following Figure 1.

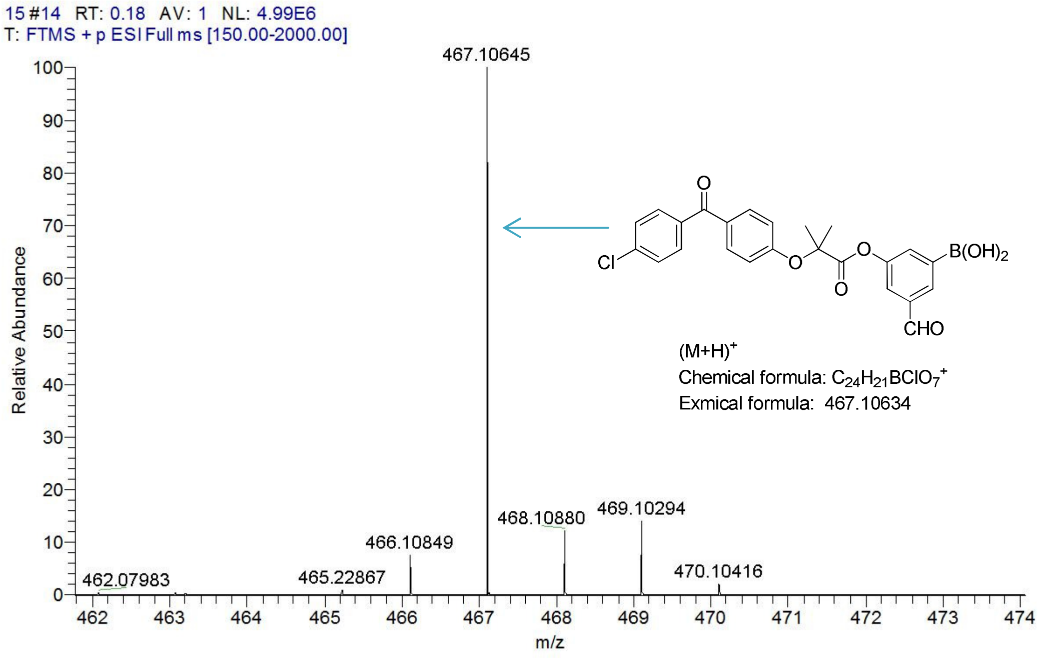

Supplementary Figure 1. HRMS of **8j**.

5 mmol-Scale synthesis of 2e and 6a

For **2e**: A 100-ml flask was charged with Ferrocene (94.9 mg, 0.5 mmol), Fe(II)Pc (29.6 mg, 0.05 mmol), $K_2S_2O_8$ (1.38 g, 5 mmol), **1e** (0.9 mL, 5 mmol), MeCN (7.5 mL), H_2O (7.5 mL), and PMHS (3.4 mL, 15 mmol) before standard three cycles of evacuation and back-filling with dry and pure oxygen. The mixture was stirred under 80℃ and 1 atm of O_2 for 3 h. Then 10 mL ammonia water was added into the mixture with vigorous stirring at room temperature. The reaction mixture was extracted with diethyl ether (3 × 15 ml). The organic phases were combined and evaporated under reduced pressure. The residue was purified by column chromatography (Petroleum ether/ diethyl ether) on silica gel to afford 0.718g (90%) of the title compound as colorless liquid.

For **6a**: A reaction of $FeCl_2$ (64 mg, 0.5 mmol), $K_2S_2O_8$ (1.38 g, 5 mmol), TBAB (814 mg, 2.5 mmol), 4-tolylboronic acid **5a** (700.8 mg, 5.0 mmol), and PMHS (3.4 mL, 15 mmol) in MeCN (7.5 mL) and H_2O (7.5 mL) was carried out in O_2 atmosphere at 80 °C for 12 h. After the mixture was cooled to room temperature, the reaction mixture was diluted with 25 mL brine and extracted with ethyl acetate (3 × 25 mL). The organic phases were combined and concentrated to give the crude product. The

residue was purified by column chromatography (Petroleum ether/ethyl acetate) on silica gel to afford (4-formylphenyl)boronic acid **6a** in 79% yield (0.593 g)

Supplementary Tables

Supplementary Table 1. Conditional optimization for iron-catalyzed oxidation of toluene (**1a**).

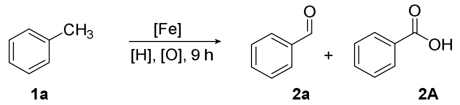

Entry	[Fe]	[H]	[O]	Solvent	% yield (**2a**/**2A**)
1	Fe(acac)$_2$	PMHS	$K_2S_2O_8$	CH_3CN	4/1
2	Fe(acac)$_2$	PMHS	$K_2S_2O_8$	CH_3CN/H_2O	81/3
3	Fe(acac)$_2$	PMHS	$K_2S_2O_8$	Dioxane/H_2O	5/-
4	Fe(acac)$_2$	PMHS	$K_2S_2O_8$	DCE/H_2O	36/5
5	Fe(acac)$_2$	PMHS	$K_2S_2O_8$	DMF/H_2O	-/-
10	-	PMHS	$K_2S_2O_8$	CH_3CN/H_2O	4/-
11	Fe(acac)$_2$	-	$K_2S_2O_8$	CH_3CN/H_2O	16/10
12	Fe(acac)$_2$	(EtO)$_3$SiH	$K_2S_2O_8$	CH_3CN/H_2O	17/2
13	Fe(acac)$_2$	Et$_3$SiH	$K_2S_2O_8$	CH_3CN/H_2O	35/3
16	Fe(acac)$_2$	PMHS	DTBP	CH_3CN/H_2O	3/-
17	Fe(acac)$_2$	PMHS	TBHP	CH_3CN/H_2O	4/-
18	Fe(acac)$_2$	PMHS	*m*CPBA	CH_3CN/H_2O	-/-
19	Fe(acac)$_2$	PMHS	H_2O_2	CH_3CN/H_2O	-/-
20	Fe(acac)$_3$	PMHS	$K_2S_2O_8$	CH_3CN/H_2O	19/3
21	$FeCl_2$	PMHS	$K_2S_2O_8$	CH_3CN/H_2O	27/6
22	Fe(OAc)$_2$	PMHS	$K_2S_2O_8$	CH_3CN/H_2O	46/4
23	$FeSO_4.7H_2O$	PMHS	$K_2S_2O_8$	CH_3CN/H_2O	61/3
24^a	Fe(II)Pc	PMHS	$K_2S_2O_8$	CH_3CN/H_2O	46/1
25	Ferrocene	PMHS	$K_2S_2O_8$	CH_3CN/H_2O	55/1
26	Ferrocene/ Fe(II)Pc(1 mol%)	PMHS	$K_2S_2O_8$	CH_3CN/H_2O	86/2
26^b	Ferrocene/ Fe(II)Pc(1 mol%)	PMHS	$K_2S_2O_8$	CH_3CN/H_2O	66/2
27^c	Ferrocene/ Fe(II)Pc(1 mol%)	PMHS	$K_2S_2O_8$	CH_3CN/H_2O	38/2
28^d	Ferrocene/ Fe(II)Pc(1 mol%)	PMHS	$K_2S_2O_8$	CH_3CN/H_2O	28/2
29^e	Ferrocene/ Fe(II)Pc(1 mol%)	PMHS	$K_2S_2O_8$	CH_3CN/H_2O	89/2
28^f	Ferrocene/ Fe(II)Pc(1 mol%)	PMHS	$K_2S_2O_8$	CH_3CN/H_2O	-/-

Reaction conditions (unless otherwise noted): **1a** (0.25 mmol), [Fe] (0.025 mmol), oxidant (0.25 mmol), [H] (3 equiv), solvent/H_2O= 1mL:1mL, 80℃, 3 h, and air. Yields were determined by ^{1}HNMR using chlorobenzene as the internal standard. ^{a} Fe(II)Pc: Fe(II)phthalocyanine. ^{b} Ferrocene 5 mol%. ^{c} PMHS (2 equiv). ^{d} 50℃. ^{e} Performed under O_2. ^{f} Performed under N_2.

Supplementary Table 2. Optimization studies for iron-catalyzed oxidation of 4-methylphenylboronic acid (**5a**).[a]

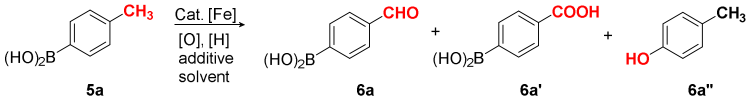

Entry	[Fe]	[O]	[H]	Additive	Solvent	Yield of **6a/6a'/6a''** (%)[b]
1	$FeCl_2$	$K_2S_2O_8$	PMHS	-	MeCN/H_2O	74/4/4
2	$FeCl_3$	$K_2S_2O_8$	PMHS	-	MeCN/H_2O	52/2/5
3	Ferrocene	$K_2S_2O_8$	PMHS	-	MeCN/H_2O	44/7/8
4	$Fe(acac)_2$	$K_2S_2O_8$	PMHS	-	MeCN/H_2O	71/4/6
5	FePc	$K_2S_2O_8$	PMHS	-	MeCN/H_2O	57/2/5
6	-	$K_2S_2O_8$	PMHS	-	MeCN/H_2O	22/3/5
7	$FeCl_2$	-	PMHS	--	MeCN/H_2O	5/0/0
8	$FeCl_2$	$K_2S_2O_8$	-	-	MeCN/H_2O	40/17/5
9	$FeCl_2$	$(NH_4)_2S_2O_8$	PMHS	-	MeCN/H_2O	71/2/3
10	$FeCl_2$	Oxone	PMHS	-	MeCN/H_2O	5/0/64
11	$FeCl_2$	H_2O_2	PMHS	-	MeCN/H_2O	25/0/4
12	$FeCl_2$	DTBP	PMHS	-	MeCN/H_2O	42/1/1
13	$FeCl_2$	$K_2S_2O_8$	$(EtO)_3SiH$	-	MeCN/H_2O	45/0/2
14	$FeCl_2$	$K_2S_2O_8$	TMDSO	-	MeCN/H_2O	73/5/2
15	$FeCl_2$	$K_2S_2O_8$	DEMS	-	MeCN/H_2O	55/2/3
16	$FeCl_2$	$K_2S_2O_8$	$PhSiH_3$	-	MeCN/H_2O	62/1/3
17	$FeCl_2$	$K_2S_2O_8$	Et_3SiH	-	MeCN/H_2O	68/6/4
18	$FeCl_2$	$K_2S_2O_8$	PMHS	-	H_2O	10/0/25
19	$FeCl_2$	$K_2S_2O_8$	PMHS	-	MeCN	0/0/0
20	$FeCl_2$	$K_2S_2O_8$	PMHS	-	*t*BuOH/H_2O	71/5/4
21	$FeCl_2$	$K_2S_2O_8$	PMHS	-	DMSO/H_2O	Trace
22	$FeCl_2$	$K_2S_2O_8$	PMHS	-	CH_2Cl_2/H_2O	26/2/16
**23**	**$FeCl_2$**	**$K_2S_2O_8$**	**PMHS**	**TBAB**	**MeCN/H_2O**	**83**(75)[c](82)[d]/2/2
24	Ferrocene/ Fe(II)Pc(1 mol%)	$K_2S_2O_8$	PMHS	TBAB	MeCN/H_2O	78/2/8
25	$FeCl_2$	$K_2S_2O_8$	PMHS	18-Crown-6	MeCN/H_2O	75/1/8
26	$FeCl_2$	$K_2S_2O_8$	PMHS	TEAB	MeCN/H_2O	82/2/2
27	$FeCl_2$	$K_2S_2O_8$	PMHS	HTAB	MeCN/H_2O	79/3/2

[a] *Reaction conditions* (unless otherwise stated): **5a** (0.25 mmol), [Fe] (10 mol%), oxidant [O] (0.25 mmol), reductant [H] (0.75 mmol), additive (0.125 mmol), solvent (2.0 mL), 80 °C, 12 h, and air. [b] Yields were determined by ^1H NMR using nitromethane as an internal standard. [c] TBAB (0.05 mmol). [d] TBAB (0.25 mmol); HTAB (Hexadecyl trimethyl ammonium bromide); TEAB (Tetraethylammonium bromide).

Supplementary Table 3. Comparison of Catalyst Systems

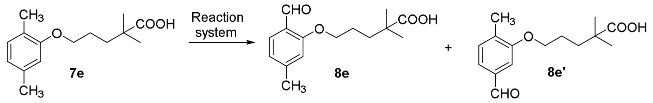

Entry	Reaction system	Ref.	Regioselectivity **8e/8e'**	yield (%)[a]
1	CuI/AcOH/DMSO	30	-	-
2	$Co(OAc)_2$/NHPI/BuOAc	41	1:2	15(69)[b]
3	$Co(OAc)_2$/NHPI/HFIP	16	2:1	67
4	$FeCl_2$/AcOH/DMSO	30	-	-
5	Ferrocene-Fe(II)Pc/$K_2S_2O_8$/PMHS	***This work***	>95:1	95

[a] ^{1}H NMR yields with anisole as an internal standard. [b] A mixture of overoxidized products with a ratio of 1:2.

Entry 1 and 4: A 25 mL flask was charged with the appropriate metal salt (0.025 mmol), Gemfibrozil (64 mg, 0.25 mmol), acetic acid (16 mg, 0.25mmol), DMSO (2 mL) before standard three cycles of evacuation and back-filling with dry and pure oxygen. The reaction mixture was stirred and heated at 100 °C during 4 h with a balloon filled with O_2 through the septum. After cooling down to room temperature, anisole (28 µL, 0.25 mmol) was added to the reaction mixture as an internal standard for ^{1}H NMR analysis of the crude material.

Entry 2: A 25 mL flask was charged with $Co(OAc)_2\cdot 4H_2O$ (0.8 mg, 0.0025 mmol), *N*-Hydroxyphthalimide (8.4 mg, 0.05mmol), Gemfibrozil (64 mg, 0.25 mmol), BuOAc (2 mL) before standard cycles (three times) of evacuation and back-filling with dry and pure Oxygene (balloon). The reaction mixture was stirred and heated at 100 °C during 4 h with a balloon filled with O_2 through the septum. After cooling down to room temperature, anisole (28 µL, 0.25 mmol) was added to the reaction mixture as an internal standard for ^{1}H NMR analysis of the crude material.

Entry 3: The result of entry 3 was reported by Pappo group (*Angew. Chem. Int. Ed.* **2017**, *56*, 5912).

Entry 5: A 25 mL flask was charged with Ferrocene (4.7 mg, 0.025 mmol), Ferrous phthalocyanine (1.5 mg, 0.0025 mmol), $K_2S_2O_8$ (68.4 mg, 0.25 mmol), Gemfibrozil (64 mg, 0.25 mmol), and PMHS (170 µL, 0.75 mmol) in MeCN (1.0 mL) and H_2O (1.0 mL) were carried out in air atmosphere at 80 °C for 4 h. After cooling down to room temperature, anisole (28 µL, 0.25 mmol) was added to the reaction mixture as an internal standard for ^{1}H NMR analysis of the crude material.

Discussion

Insight into aromatic radical cation intermediate

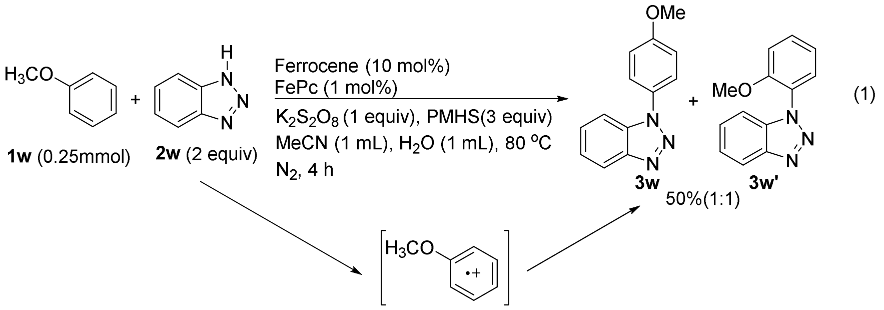

Following the *general procedure A*, a reaction of Ferrocene (4.7 mg, 0.025 mmol), Ferrous phthalocyanine (1.5 mg, 0.0025 mmol), $K_2S_2O_8$ (68.4 mg, 0.25 mmol), **1w** (29 μL, 0.25 mmol), **2w** (60.2 mg, 0.5 mmol), and PMHS (170 μL, 0.75 mmol) in MeCN/H_2O (2.0 mL) were carried out in N_2 atmosphere at 80 °C for 4 h. 1-(2-Methoxyphenyl)-1H-benzo[d][1,2,3]triazole and 1-(4-methoxyphenyl)-1H-benzo [d][1,2,3]triazole (1:1) (**3w** and **3w'**) was isolated as a yellow solid (28.1 mg, 50%), known compound. The NMR spectroscopic data agree those described in ref.[33] ^{1}H NMR (400 MHz, $CDCl_3$): δ 8.13 (dd, *J* = 8.4, 2.8Hz, 2H), 7.66 (m, 3H), 7.52 (m, 3H), 7.45 (m 2H), 7.39 (m 2H), 7.16 (m, 2H), 7.11 (d, *J* = 8.9 Hz, 2H), 3.90 (s, 3H), 3.80 (s, 3H); ^{13}C NMR (100 MHz, $CDCl_3$): δ 159.8, 153.6, 145.9, 145.1, 134.0, 132.6, 131.1, 129.8, 128.08, 128.06, 127.7, 125.1, 124.6, 124.4, 124.1, 121.1, 120.0, 119.6, 114.9, 112.3, 111.2, 110.3, 55.8, 55.6ppm. This result supports formation of aromatic radical cation intermediate in the present catalytic system.[33]

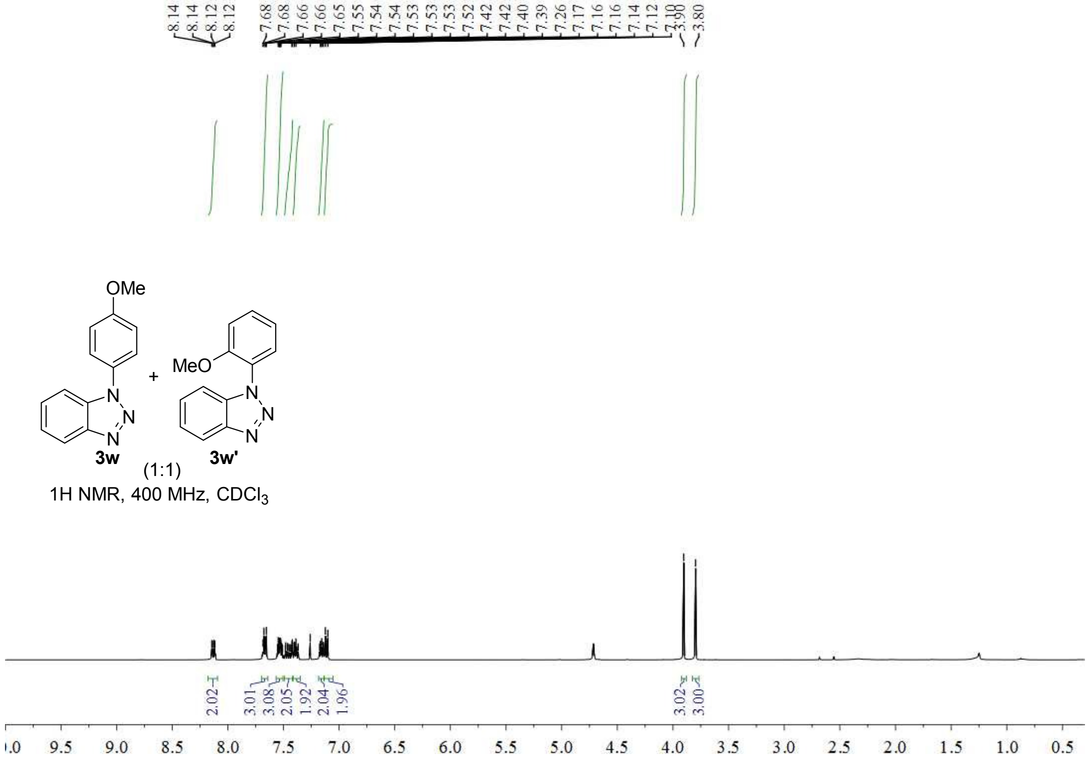

Supplementary Figure 2. ^{1}H NMR (400 MHz, $CDCl_3$) of compound **3w** and **3w' (1:1)**.

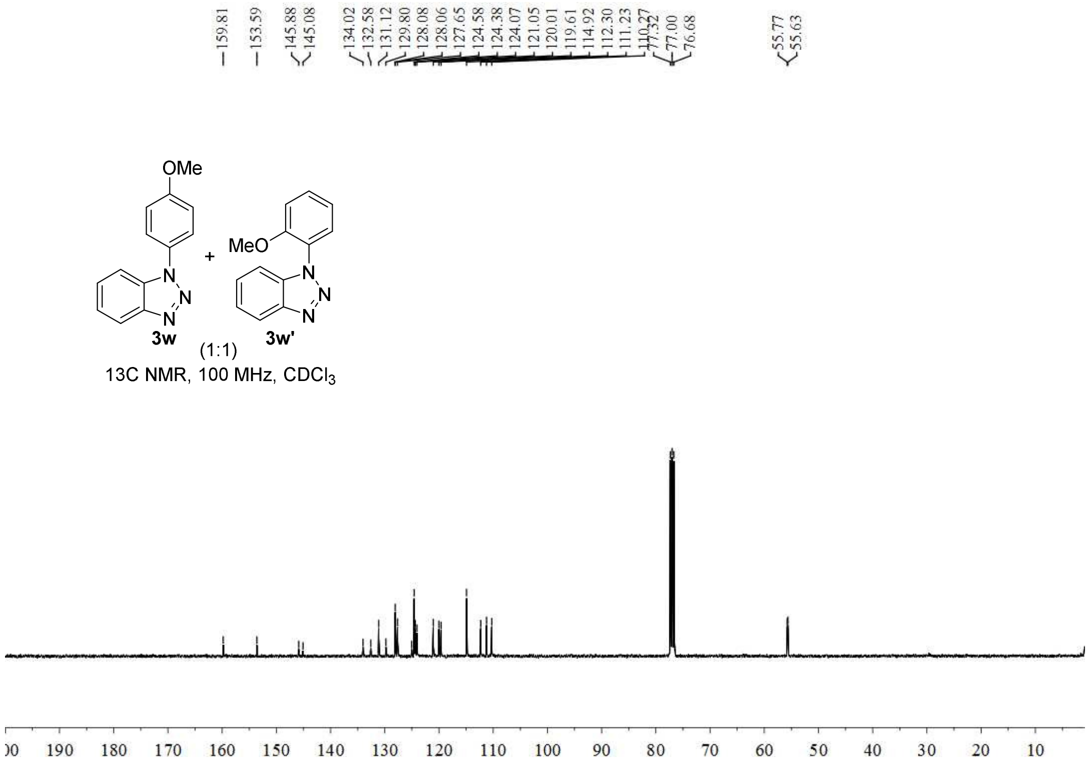

Supplementary Figure 3. ^{13}C NMR (100 MHz, $CDCl_3$) of compound **3w** and **3w' (1:1)**.

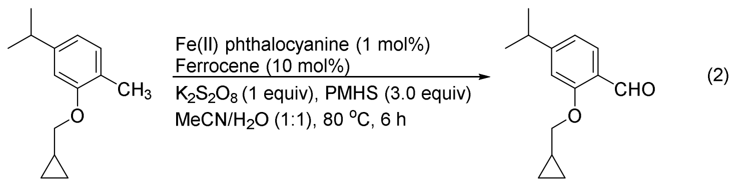

1x (0.25 mmol) **2x** (72 %)

Following the *general procedure A*, a reaction of Ferrocene (4.7 mg, 0.025 mmol), Ferrous phthalocyanine (1.5 mg, 0.0025 mmol), $K_2S_2O_8$ (68.4 mg, 0.25 mmol), **1x** (51.5 mg, 0.25 mmol), and PMHS (170 μL, 0.75 mmol) in MeCN/H_2O (2.0 mL) were carried out in air atmosphere at 80 °C for 6 h. 2-(Cyclopropylmethoxy)-4-isopropylbenzaldehyde (**2x**) was isolated as a colorless liquid (39.4 mg, 72%), known compound (CAS: 1289164-41-5). ^{1}H NMR (400 MHz, $CDCl_3$): δ 10.49 (s, 1H), 7.76 (d, J = 8.0 Hz, 1H), 6.88 (d, J = 8.0 Hz, 1H), 6.78 (s, 1H), 3.94 (d, J = 6.8 Hz, 2H), 2.91 (septuplet, J = 6.9 Hz, 1H), 1.33-1.30 (m, 1H), 1.25 (d, J = 6.9 Hz, 6H), 0.69-0.64 (m, 2H), 0.40-0.36 (m, 2H); ^{13}C NMR (100 MHz, $CDCl_3$): δ 189.7, 161.7, 158.1, 128.3, 123.2, 119.0, 110.9, 73.2, 34.9, 23.5, 10.1, 3.1. The probe substrate (**1x**) containing isopropyl and methyl substitutes was subjected to the standard reaction conditions and give the single methyl oxidation product **2x (**72%**)** with the retention of the isopropyl group. This findings rule out that sulfate radical anion may abstracts a hydrogen atom from alkylarene to produce the benzyl radical **(**Oxidation of tertiary benzylic C-H is much easier than primary benzylic C-H

by hydrogen atom transfer mechanism)[34] and further support the process of SET to produce the alkylaromatic radical cation.[35]

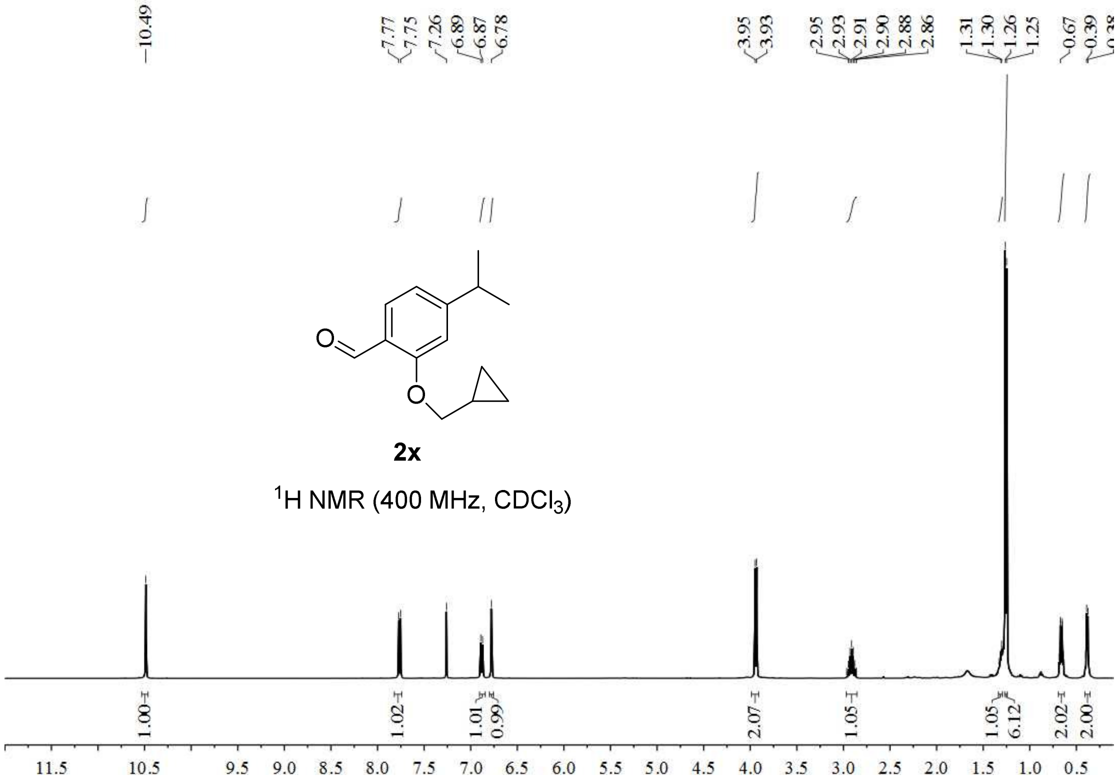

Supplementary Figure 4. ^{1}H NMR (400 MHz, $CDCl_3$) of compound **2x**.

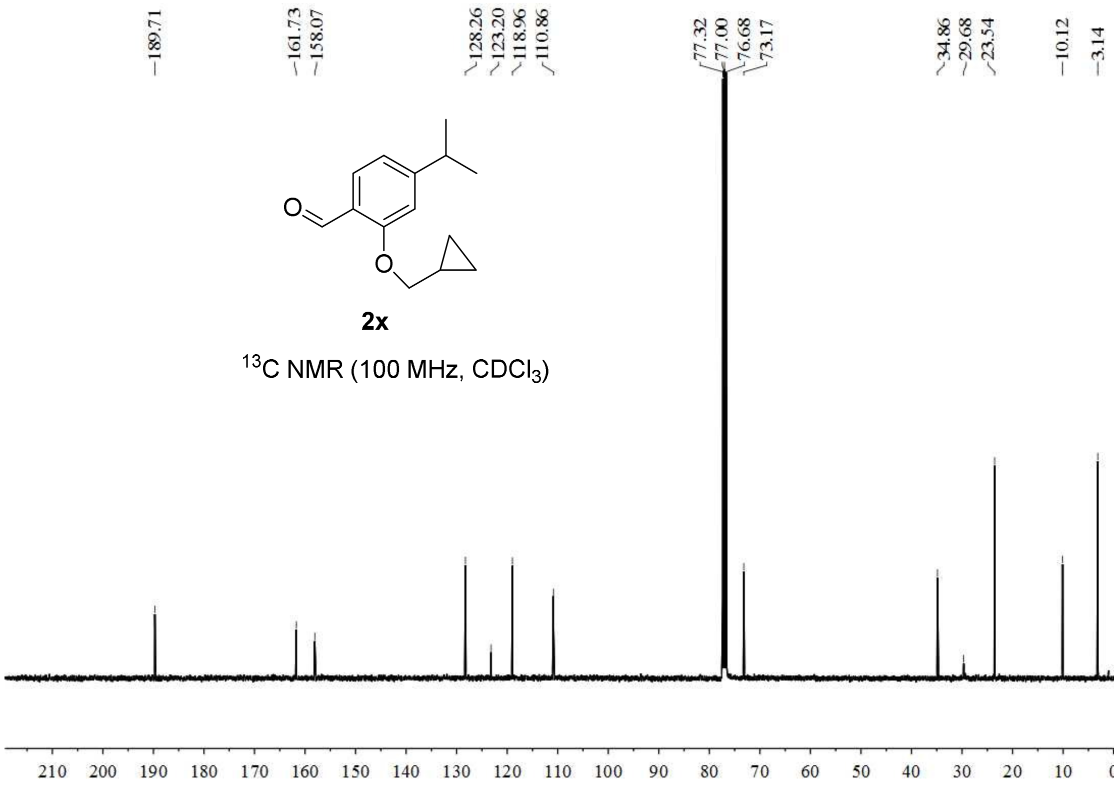

Supplementary Figure 5. ^{13}C NMR (100 MHz, $CDCl_3$) of compound **2x**.

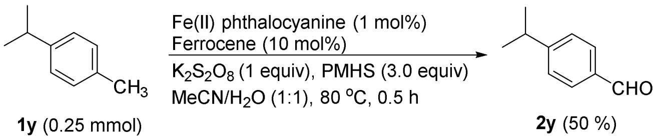

(3)

Following the *general procedure A*, a reaction of Ferrocene (4.7 mg, 0.025 mmol), Ferrous phthalocyanine (1.5 mg, 0.0025 mmol), $K_2S_2O_8$ (68.4 mg, 0.25 mmol), **1y** (41 μL, 0.25 mmol), and PMHS (170 μL, 0.75 mmol) in MeCN/H_2O (2.0 mL) were carried out in air atmosphere at 80 °C for 0.5 h. 4-Isopropylbenzaldehyde (**2y**) was isolated as a colorless liquid (18.5 mg, 50%), known compound. The NMR spectroscopic data agree with those described in ref.[36]. ^{1}H NMR (400 MHz, $CDCl_3$): ^{1}H NMR (400 MHz, $CDCl_3$): δ 9.97 (s, 1H), 7.81 (d, J = 8.0 Hz, 2H), 7.32 (d, J = 8.0 Hz, 2H), 2.99 (septuplet, J = 8.0 Hz, 1H), 1.28 (d, J = 8.0 Hz, 6H); ^{13}C NMR (100 MHz, $CDCl_3$): δ 192.0, 156.2, 134.5, 130.0, 127.1, 34.5, 23.6. The probe substrate (**1y**) containing isopropyl and methyl substitutes was subjected to the standard reaction conditions and also give the single methyl oxidation product **2y** (50%**)** with the retention of the isopropyl group. This findings also rule out that sulfate radical anion may abstracts a hydrogen atom from alkylarene to produce the benzyl radical **(**Oxidation of tertiary benzylic C-H is much easier than primary benzylic C-H by hydrogen atom transfer mechanism**)**[34] and further support the process of SET to produce the alkylaromatic radical cation.[35]

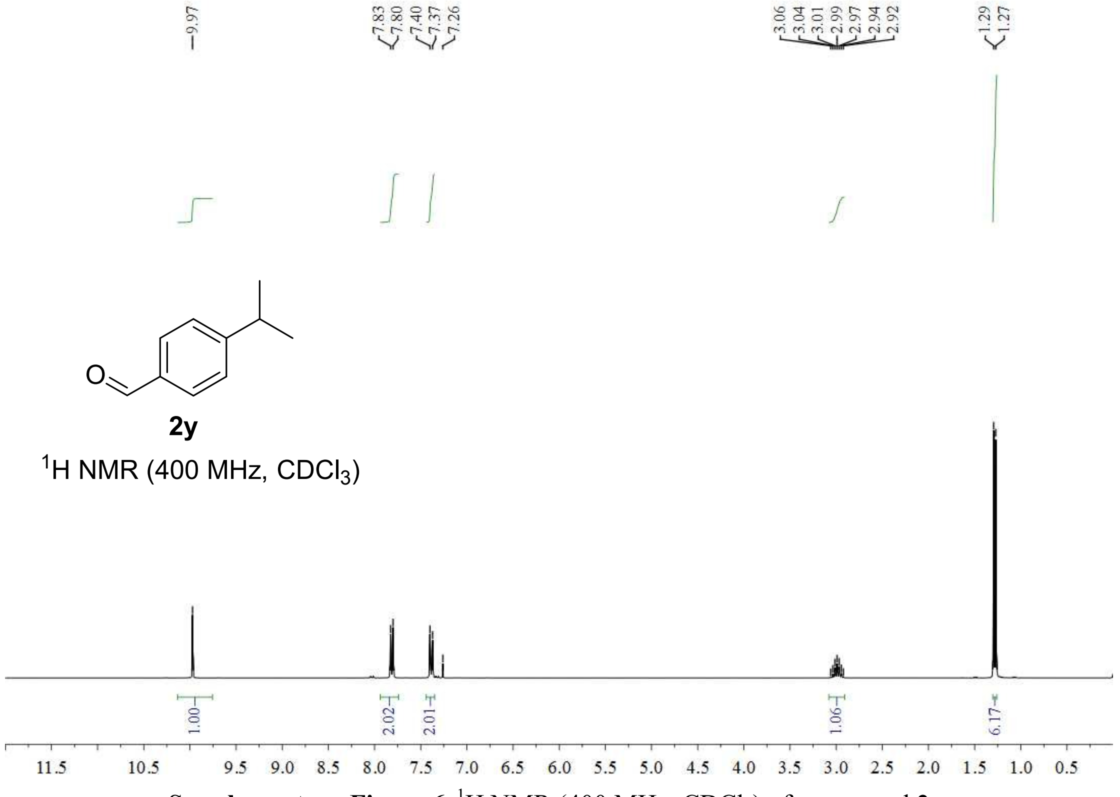

Supplementary Figure 6. ^{1}H NMR (400 MHz, $CDCl_3$) of compound **2y**.

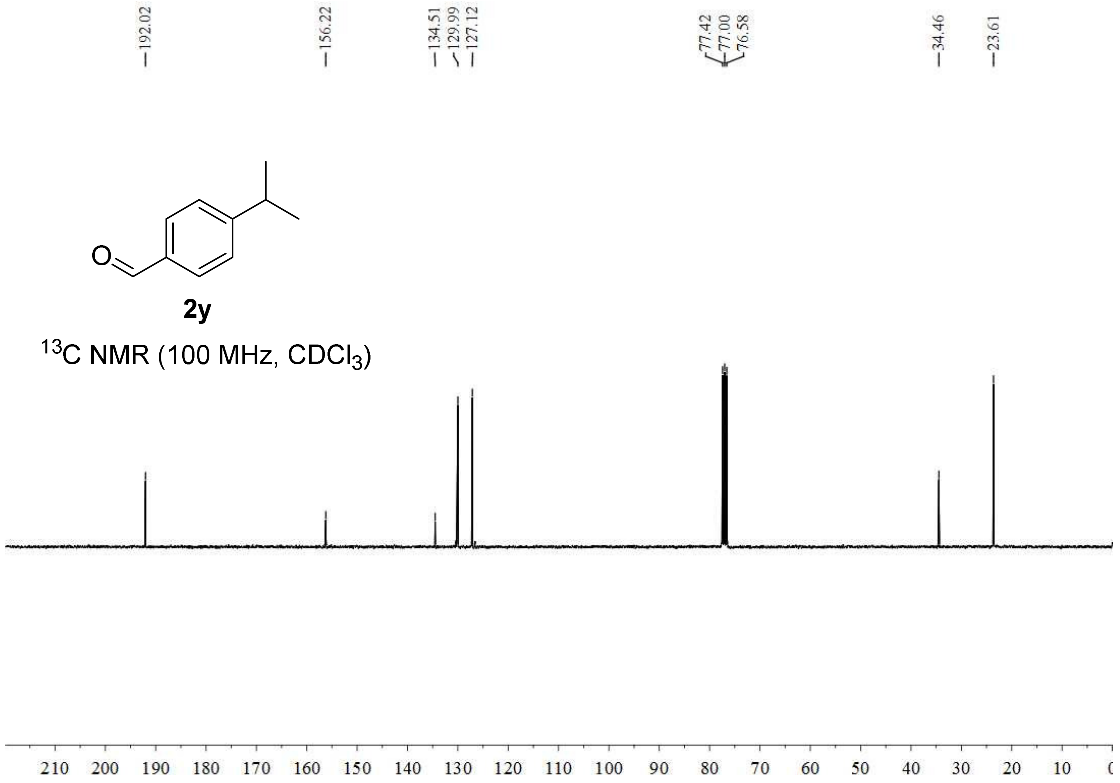

Supplementary Figure 7. ^{13}C NMR (100 MHz, $CDCl_3$) of compound **2y**.

Interception of radical intermediate

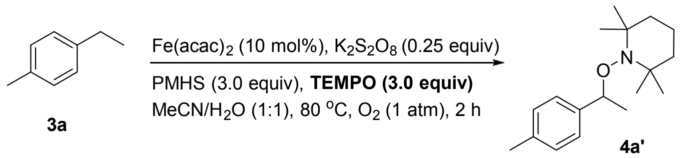

(4)

Following the *general procedure A*, a reaction of $Fe(acac)_2$ (6.4 mg, 0.025 mmol), $K_2S_2O_8$ (17.1 mg, 0.063 mmol), TEMPO (0.75 mmol), **3a** (36μL, 0.25 mmol), and PMHS (170 μL, 0.75 mmol) in $MeCN/H_2O$ (2.0 mL) were carried out in O_2 atmosphere at 80 °C for 2 h. Consequently, the oxidation process is completely inhibited and a benzyl radical intermediate is intercepted by TEMPO to generate **4a'** that is detected according to HRMS (ESI) analysis (Figure S1). This result supports formation of carbon radical intermediate.

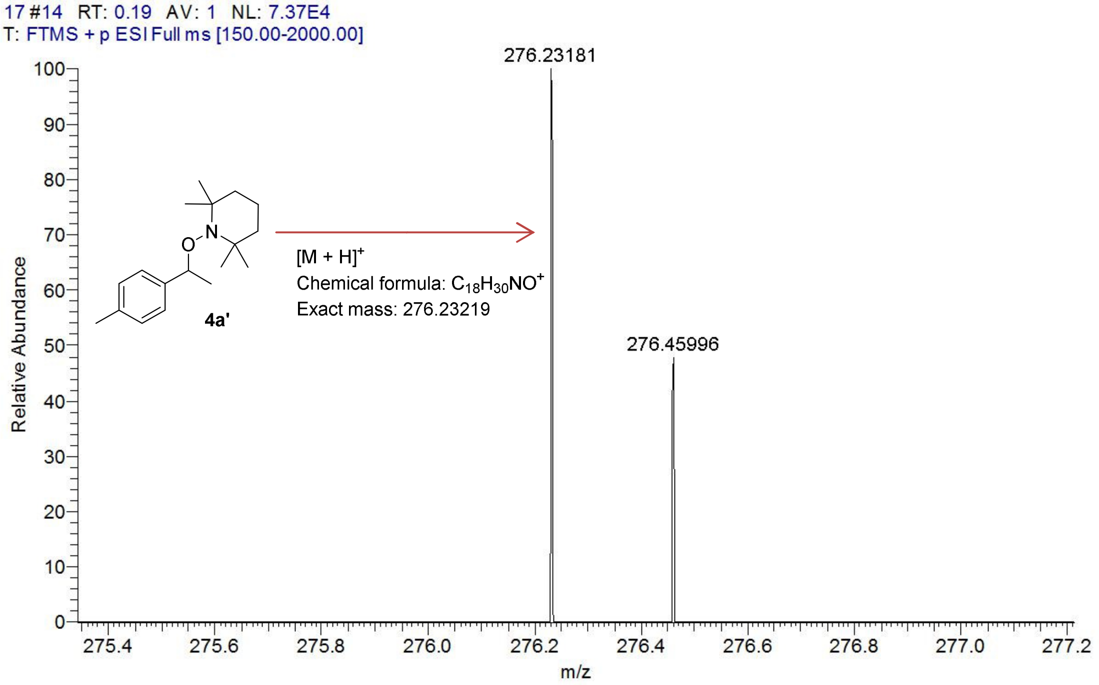

Supplementary Figure 8. HRMS of **4a'**.

Kinetic isotope effect (KIE)

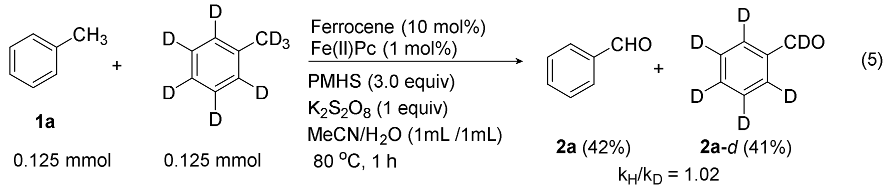

Following the *general procedure A*, we investigated the proton/deuterium KIE of the reaction. The yields were determined by GC/MS analysis of the crude reaction mixture using chlorobenzene as the internal standard. The value of k_H/k_D is 1.02 suggests that the cleavage of the C–H bond is not the overall turnover-limiting step.

The use of copper instead of PMHS as the reductant

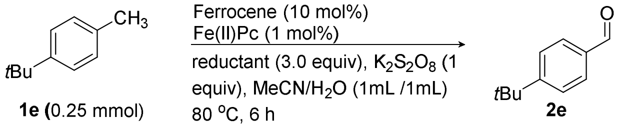

Entry	reductant	yield (%)
1	-	13
2	PMHS	91
3	Cu powder	52
4	Cu powder (without [Fe])	<5

Entry 1: Following the *general procedure A*, a reaction of Ferrocene (4.7 mg, 0.025 mmol), Ferrous phthalocyanine (1.5 mg, 0.0025 mmol), $K_2S_2O_8$ (68.4 mg, 0.25 mmol), and **1e** (45 μL, 0.25 mmol) in MeCN/H_2O (1:1, 2.0 mL) were carried out in air atmosphere at 80 °C for 6 h. After cooling down to room temperature, anisole (28 μL, 0.25 mmol) was added to the reaction mixture as an internal standard for ^1H NMR analysis of the crude material.

Entry 3: Following the *general procedure A*, a reaction of Ferrocene (4.7 mg, 0.025 mmol), Ferrous phthalocyanine (1.5 mg, 0.0025 mmol), $K_2S_2O_8$ (68.4 mg, 0.25 mmol), copper powder (48 mg, 0.75 mmol), and **1e** (45 μL, 0.25 mmol) in MeCN/H_2O (1:1, 2.0 mL) were carried out in air atmosphere at 80 °C for 6 h. After cooling down to room temperature, the mixture was purified by column chromatography (Petroleum ether/ diethyl ether) on silica gel to afford **2e** in 52% yield.

Entry 4: Following the *general procedure A*, a reaction of $K_2S_2O_8$ (68.4 mg, 0.25 mmol), copper powder (48 mg, 0.75 mmol), and **1e** (45 μL, 0.25 mmol) in MeCN/H_2O (1:1, 2.0 mL) were carried out in air atmosphere at 80 °C for 6 h. After cooling down to room temperature, anisole (28 μL, 0.25 mmol) was added to the reaction mixture as an internal standard for ^1H NMR analysis of the crude material.

^{1}H NMR monitoring the reaction of 1a

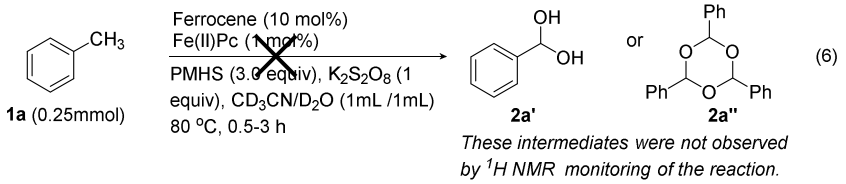

As per the *general procedure A*, three reactions of Ferrocene (4.7 mg, 0.025 mmol), Ferrous phthalocyanine (1.5 mg, 0.0025 mmol), $K_2S_2O_8$ (68.4 mg, 0.25 mmol), **1a** (28 μL, 0.25 mmol), and PMHS (170 μL, 0.75 mmol) in CD_3CN (1.0 mL) and D_2O (1.0 mL) were carried out in air atmosphere at 80 °C for 0.5 h, 2 h and 6 h, respectively, one as a control. Subsequent ^{1}H NMR analysis of the reaction mixtures suggests that the intermediates **2a'** and **2a''** are absent in the reactions.

Benzoic acid as the substrate

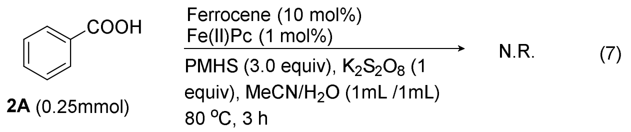

Following the *general procedure A*, a reaction of Ferrocene (4.7 mg, 0.025 mmol), Ferrous phthalocyanine (1.5 mg, 0.0025 mmol), $K_2S_2O_8$ (68.4 mg, 0.25 mmol), **2A** (30.8 mg, 0.25 mmol), and PMHS (170 μL, 0.75 mmol) in MeCN (1.0 mL) and H_2O (1.0 mL) were carried out in air atmosphere at 80 °C 3 h. As shown in equation, 100% **2A** was recovered without any aldehyde **2a**, implying that the high chemoselectivity for aldehyde did not result from reduction of over-oxidized carboxylic acid.

The effect of PMHS on the overoxidation

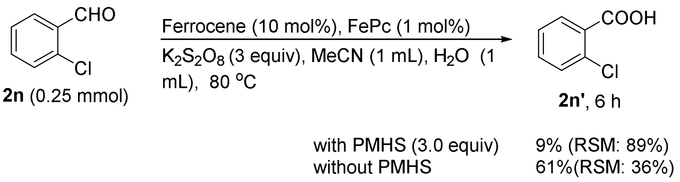

As per the *general procedure A*, two reactions of Ferrocene (4.7 mg, 0.025 mmol), Ferrous phthalocyanine (1.5 mg, 0.0025 mmol), $K_2S_2O_8$ (205 mg, 0.75 mmol), **2n** (35.5 mg, 0.25 mmol) in MeCN/H_2O (1:1, 2.0 mL) were carried out, one as a control. PMHS, 3 equiv (170 μL, 0.75 mmol) and 0 equiv, respectively, was introduced to the reactions. All reaction mixtures were stirred under air atmosphere at 80 °C for 6 h. As shown in equation, these two experimental observations indicate PMHS suppresses the overoxidation of aldehyde, suggesting that PMHS plays an important role in achieving high chemoselectivity for aldehydes in the present transformation.

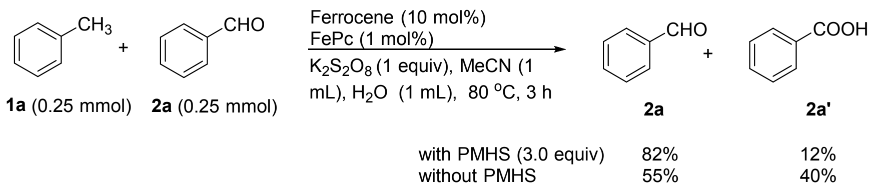

As per the *general procedure A*, two reactions of Ferrocene (4.7 mg, 0.025 mmol), Ferrous phthalocyanine (1.5 mg, 0.0025 mmol), $K_2S_2O_8$ (68.3 mg, 0.25 mmol), **1a** (28 µL, 0.25 mmol), **2a** (27 µL, 0.25 mmol) in MeCN/H_2O (1:1, 2.0 mL) were carried out, one as a control. PMHS, 3 equiv (170 µL, 0.75 mmol) and 0 equiv, respectively, was introduced to the reactions. All reaction mixtures were stirred under air atmosphere at 80 °C for 3 h. As shown in the above equation, these two experimental observations indicate PMHS suppresses the overoxidation of aldehyde, further suggesting that PMHS plays an important role in achieving high chemoselectivity for aldehydes in the present transformation.

Copies of NMR spectra

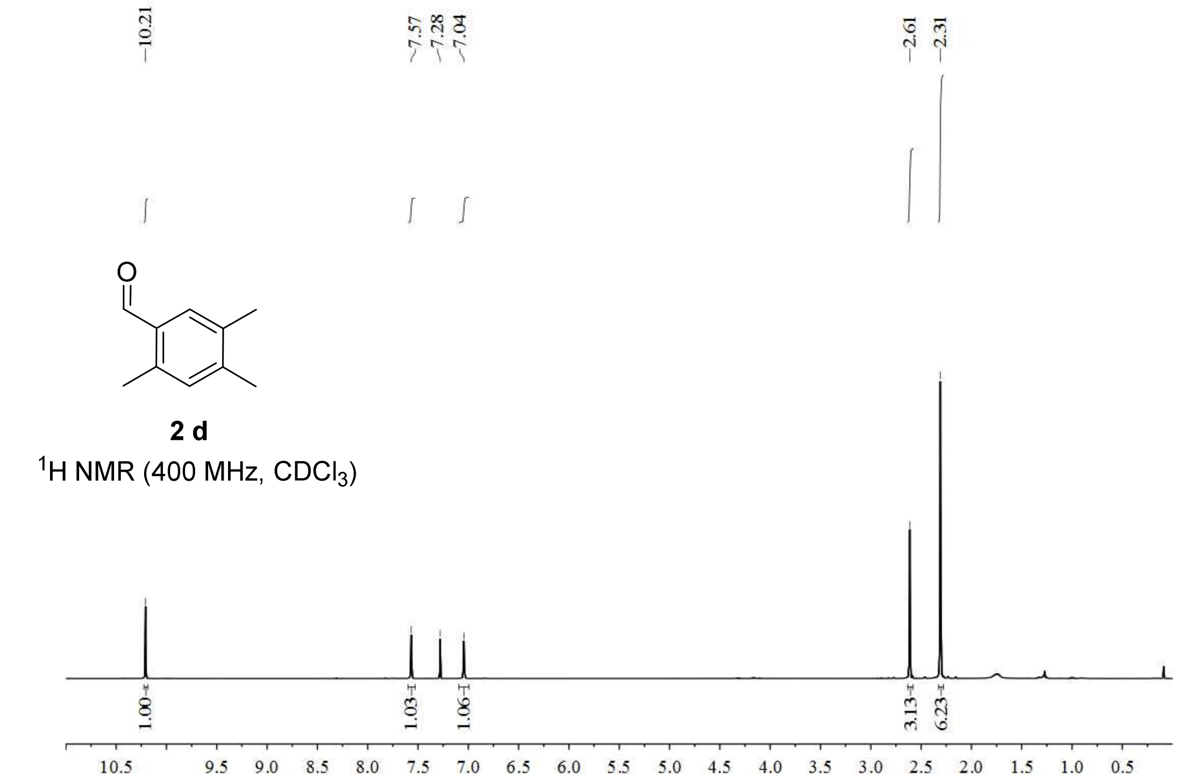

Supplementary Figure 9. ^{1}H NMR (400 MHz, $CDCl_3$) of compound **2d**.

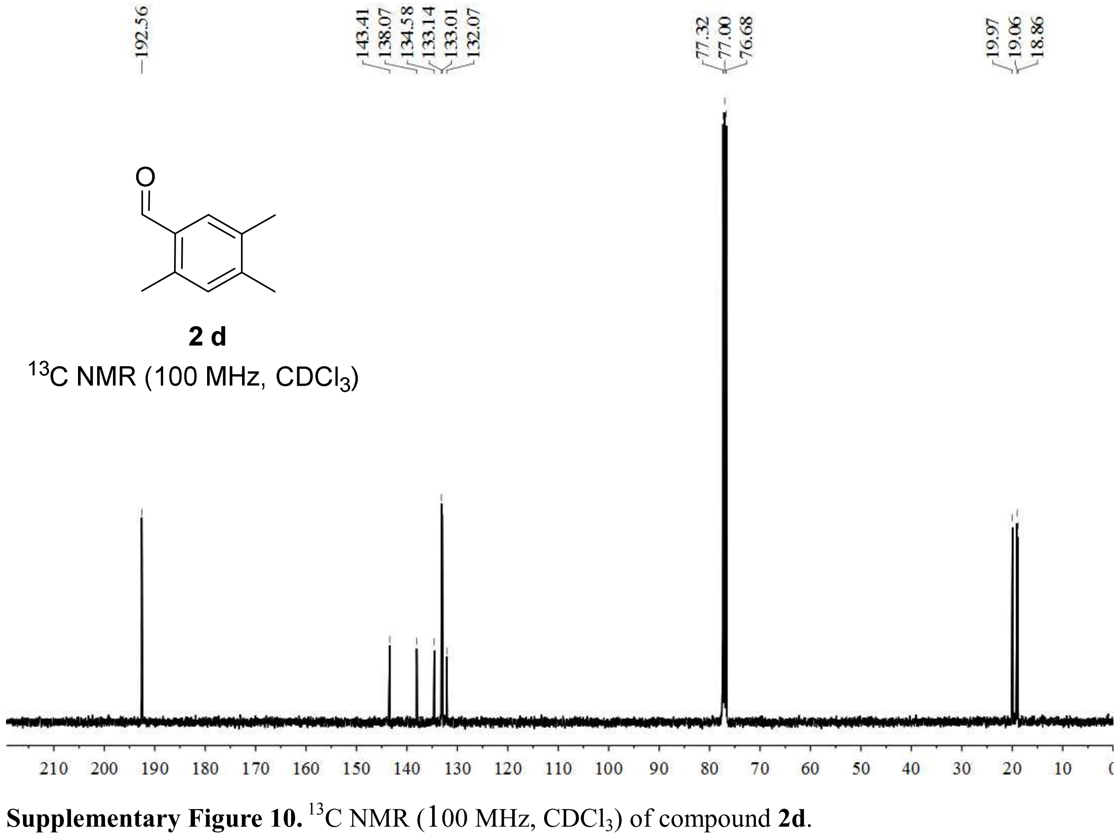

Supplementary Figure 10. ^{13}C NMR (100 MHz, $CDCl_3$) of compound **2d**.

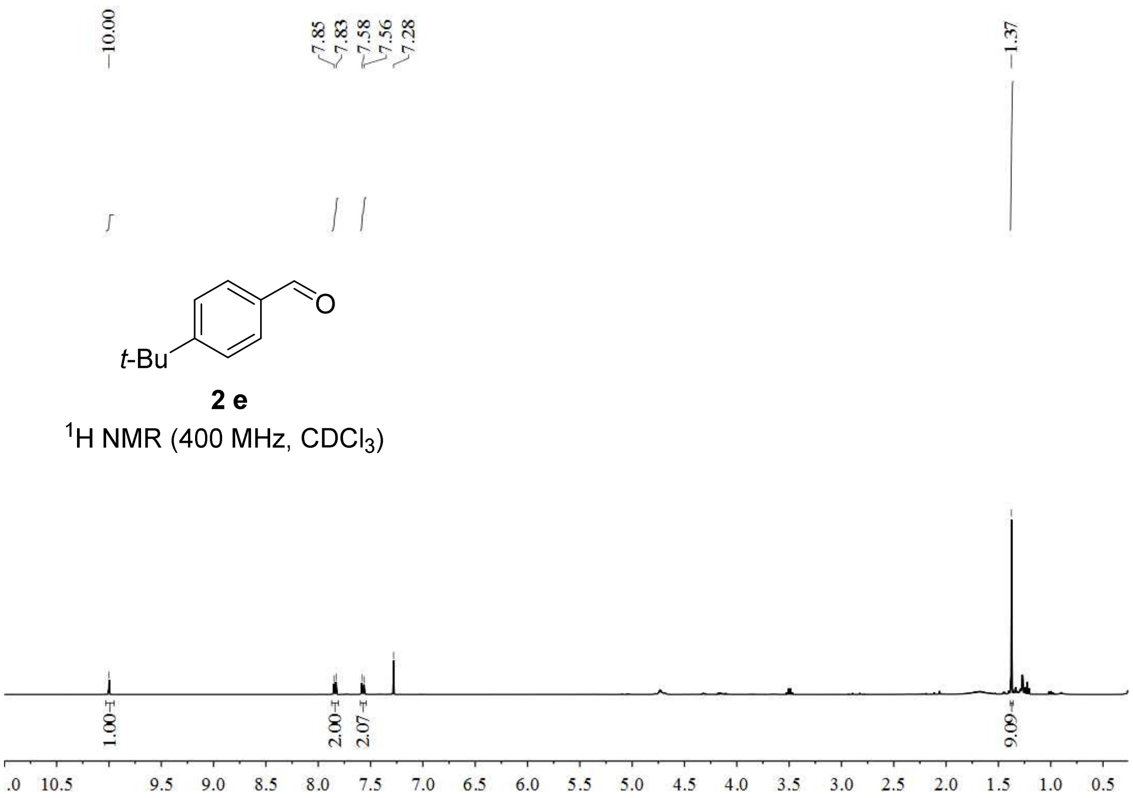

Supplementary Figure 11. ^{1}H NMR (400 MHz, $CDCl_3$) of compound **2e**.

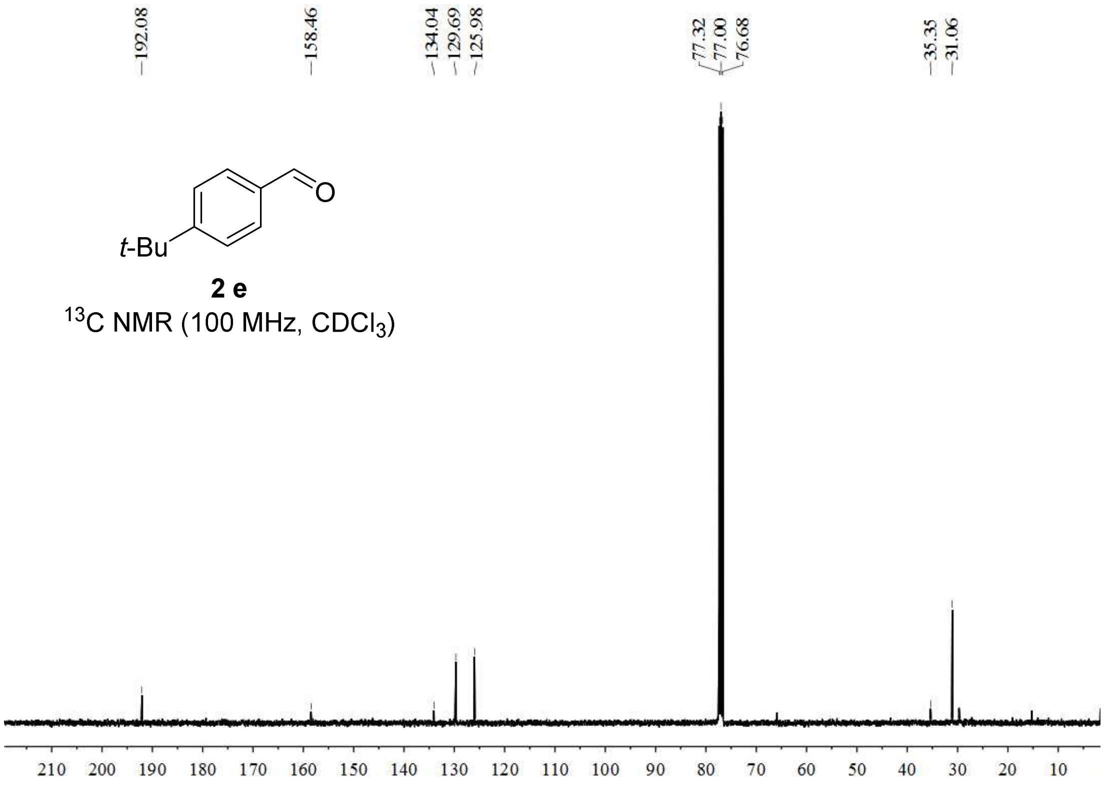

Supplementary Figure 12. ^{13}C NMR (100 MHz, $CDCl_3$) of compound **2e**.

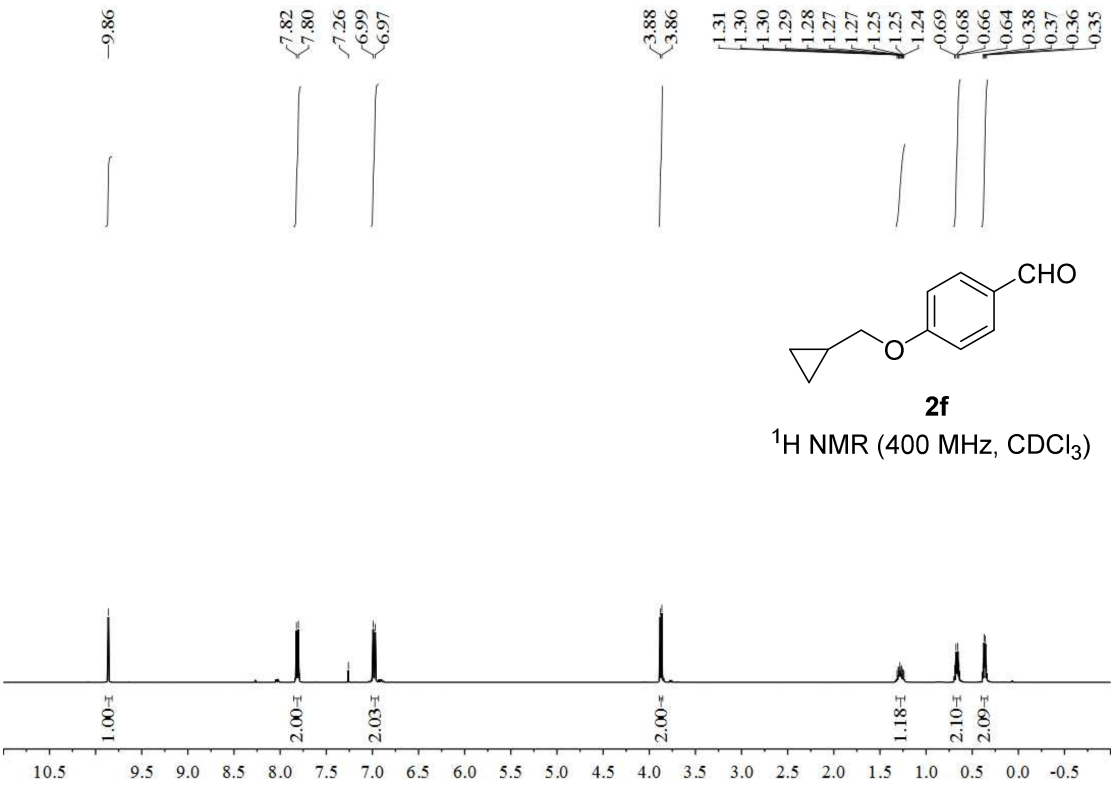

Supplementary Figure 13. ^{1}H NMR (400 MHz, $CDCl_3$) of compound **2f**.

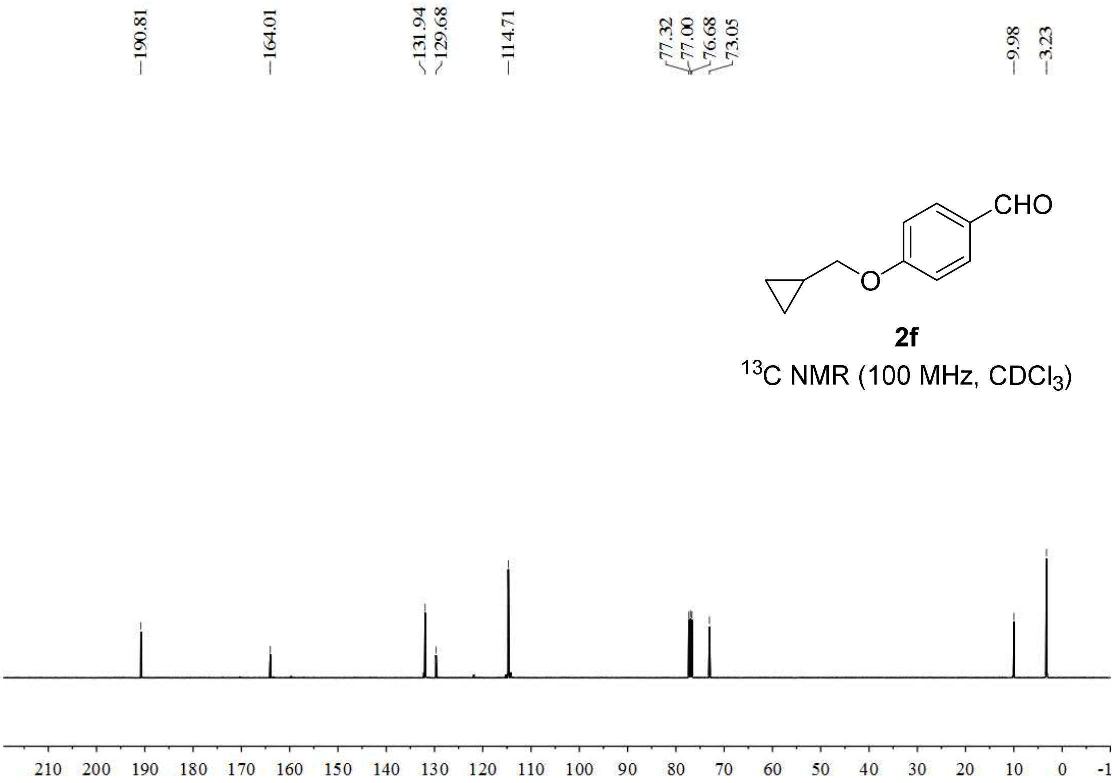

Supplementary Figure 14. ^{13}C NMR (100 MHz, $CDCl_3$) of compound **2f**.

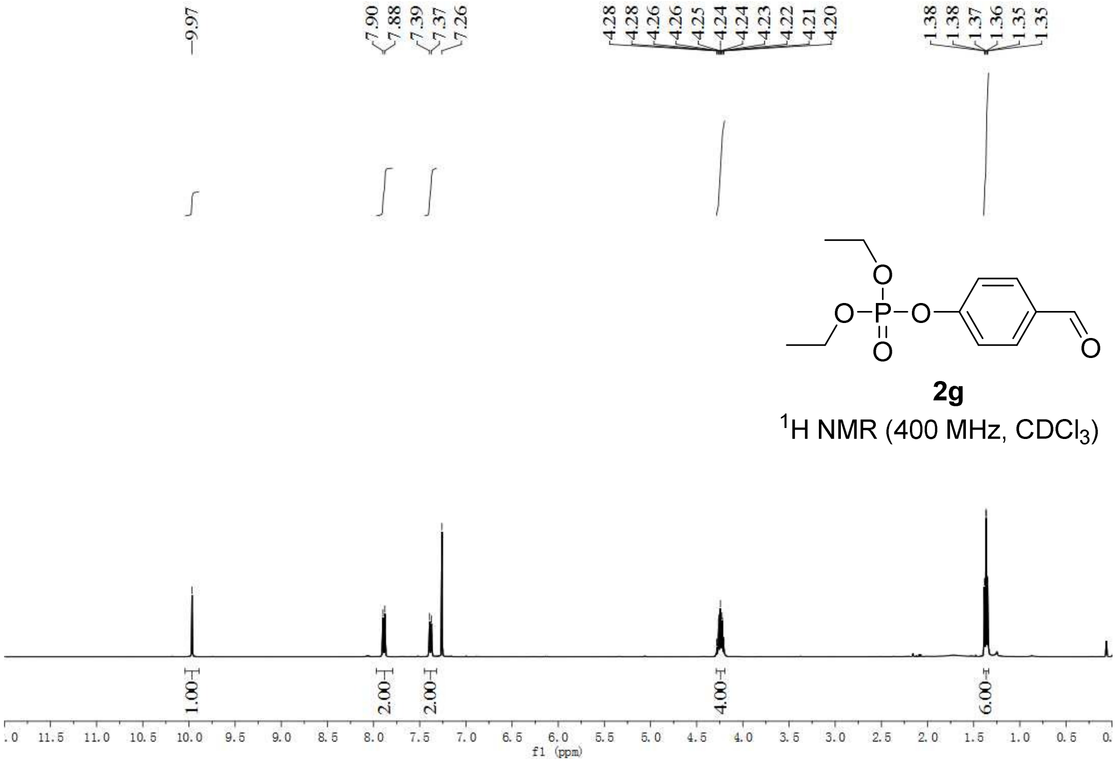

Supplementary Figure 15. ^{1}H NMR (400 MHz, $CDCl_3$) of compound **2g**.

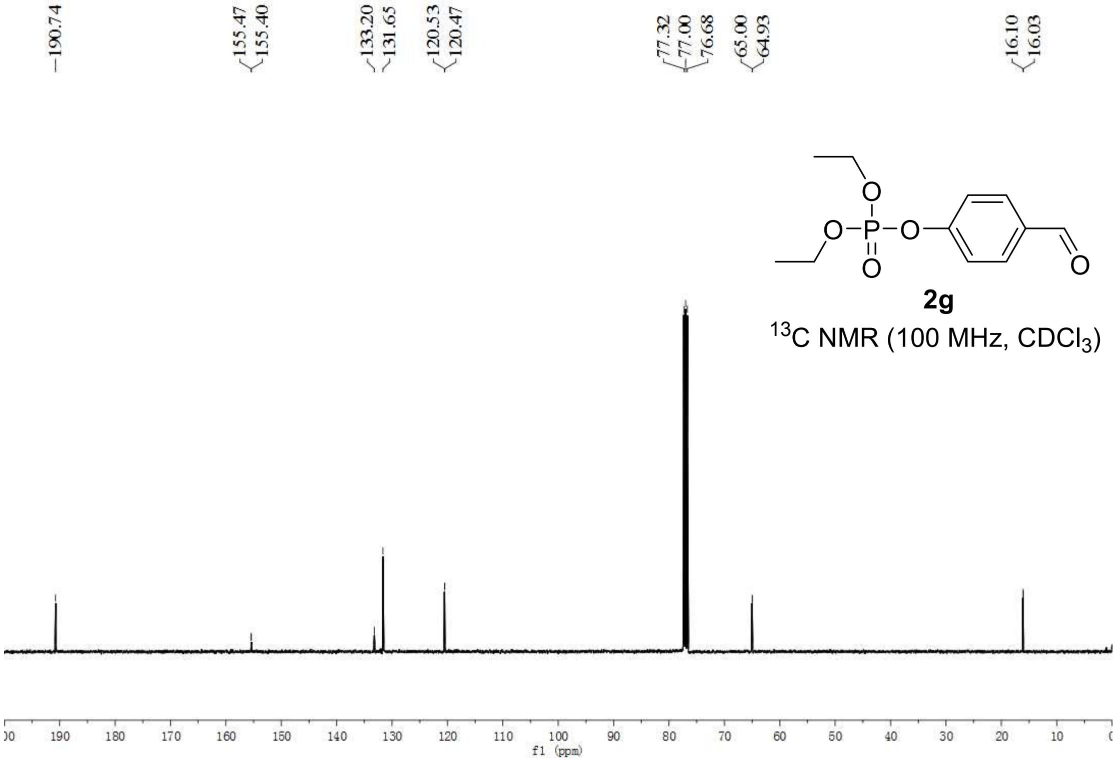

Supplementary Figure 16. ^{13}C NMR (100 MHz, $CDCl_3$) of compound **2g**.

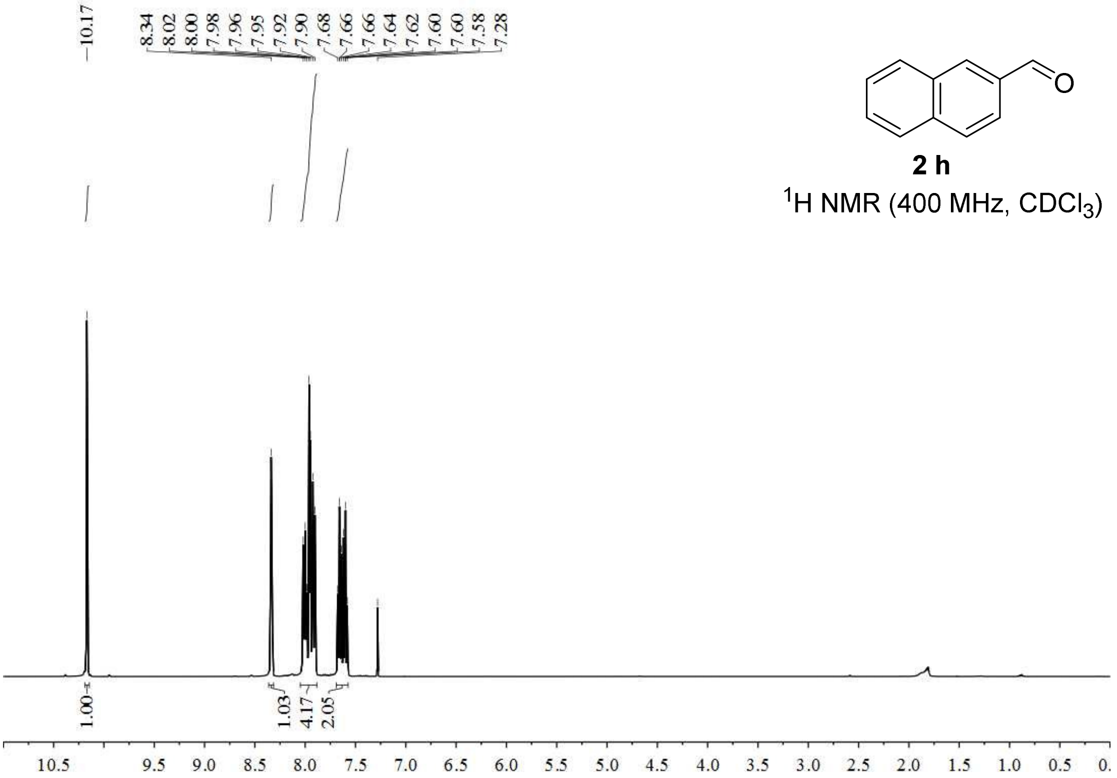

Supplementary Figure 17. ^{1}H NMR (400 MHz, $CDCl_3$) of compound **2h**.

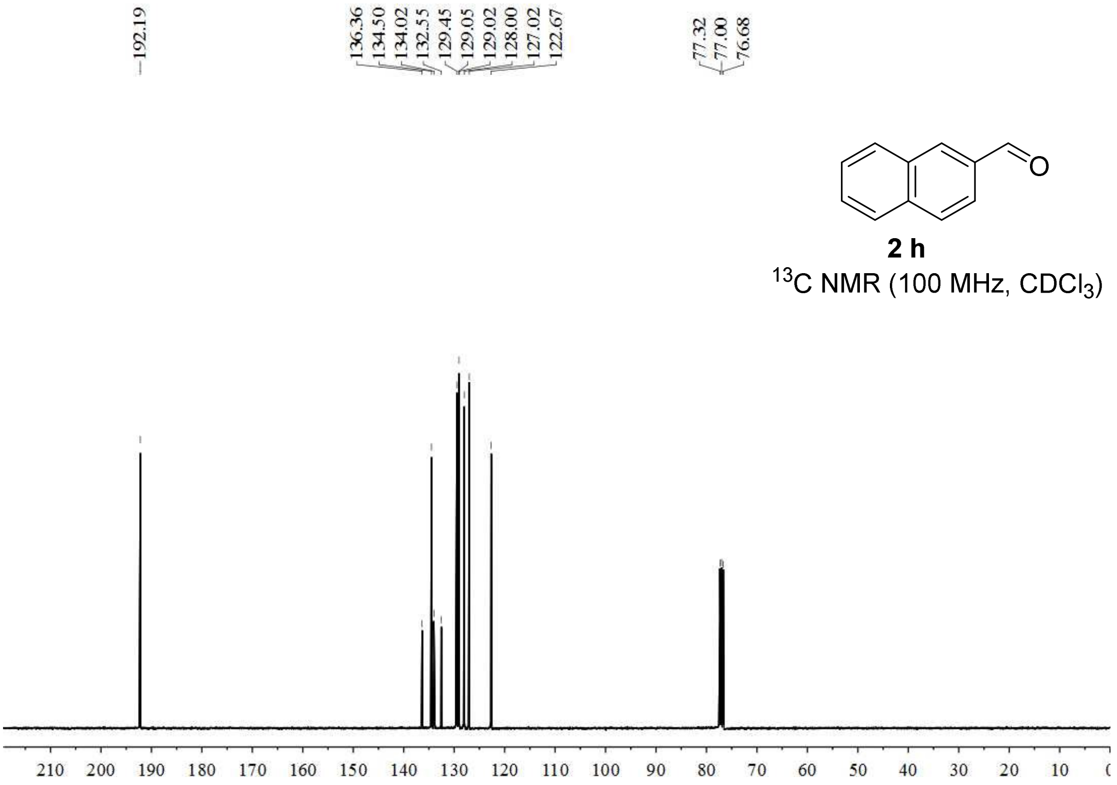

Supplementary Figure 18. ^{13}C NMR (100 MHz, $CDCl_3$) of compound **2h**.

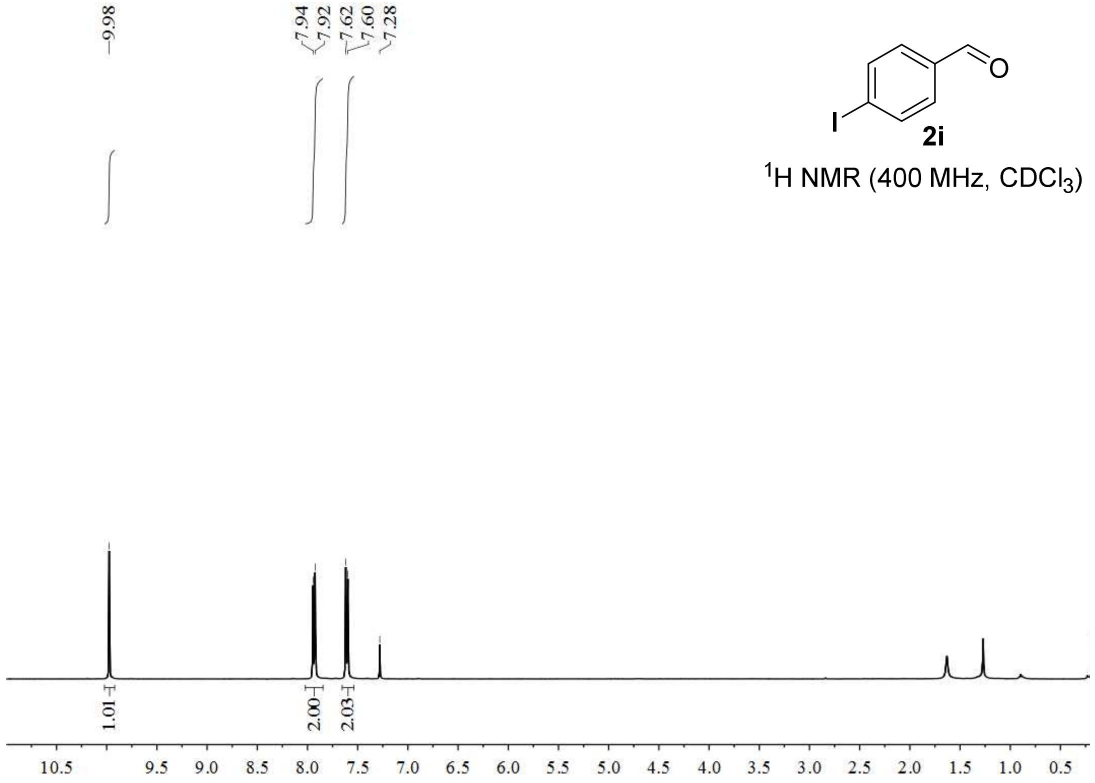

Supplementary Figure 19. ^{1}H NMR (400 MHz, $CDCl_3$) of compound **2i**.

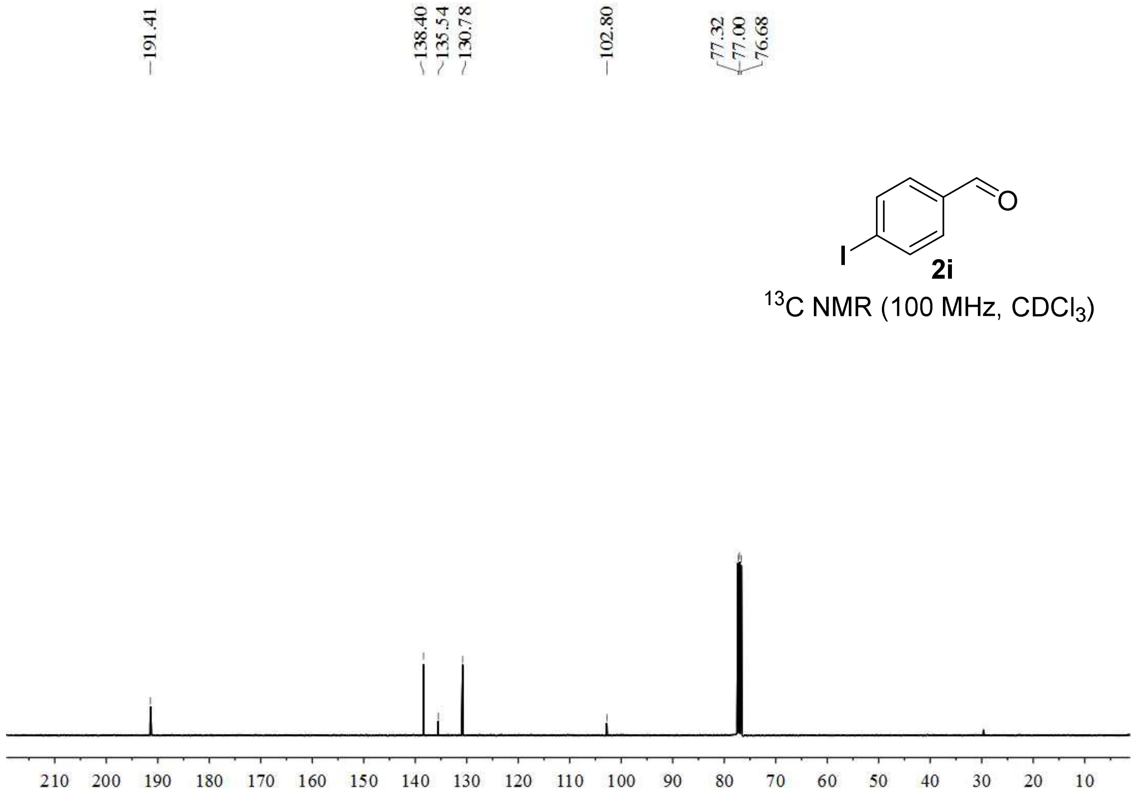

Supplementary Figure 20. ^{13}C NMR (100 MHz, $CDCl_3$) of compound **2i**.

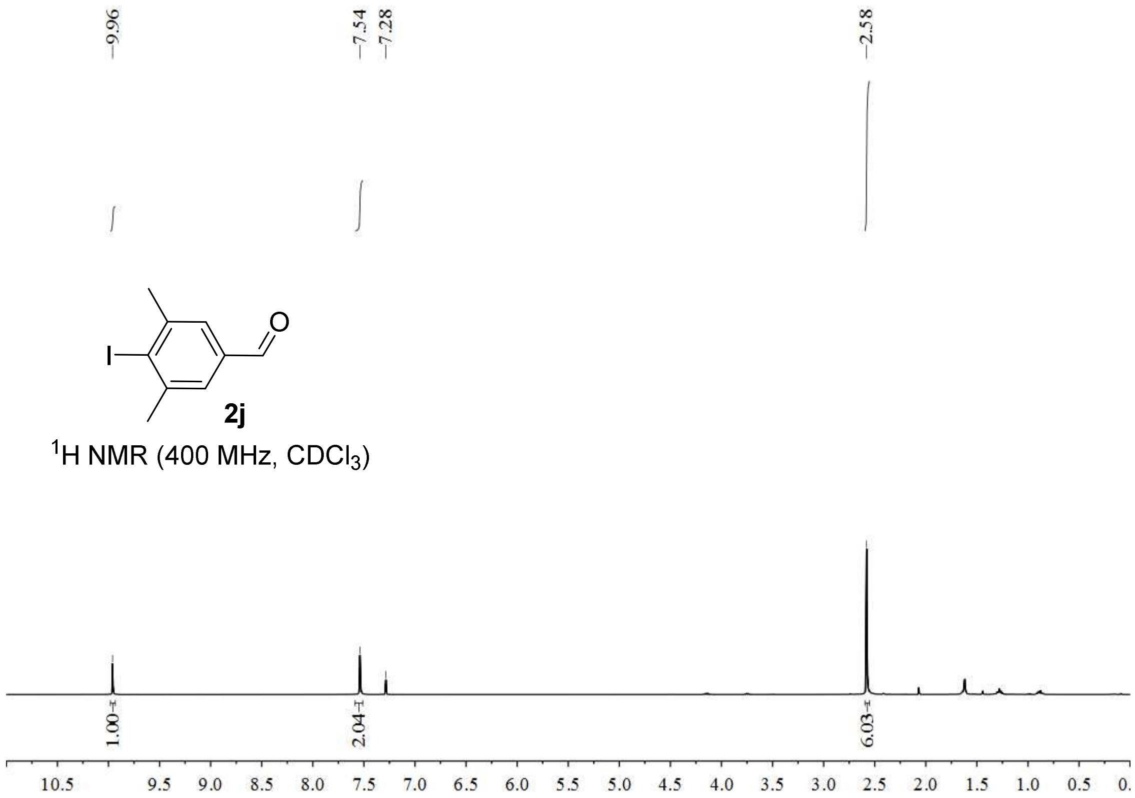

Supplementary Figure 21. ^{1}H NMR (400 MHz, $CDCl_3$) of compound **2j**.

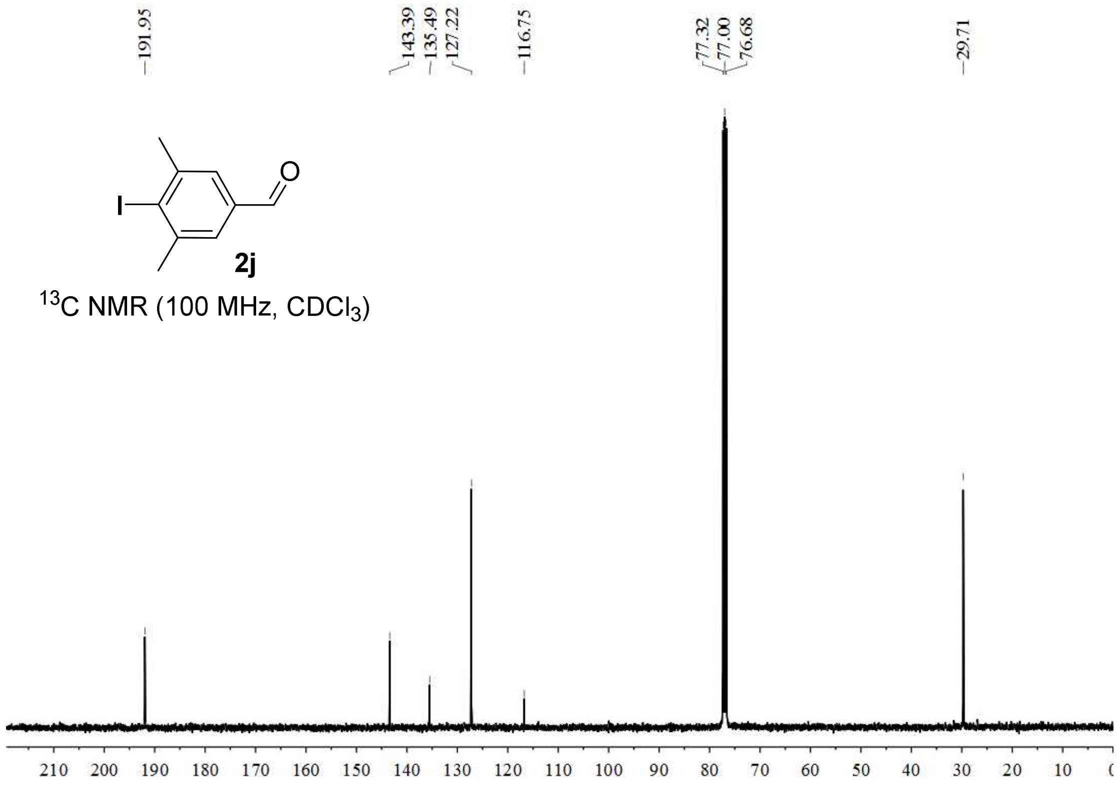

Supplementary Figure 22. ^{13}C NMR (100 MHz, $CDCl_3$) of compound **2j**.

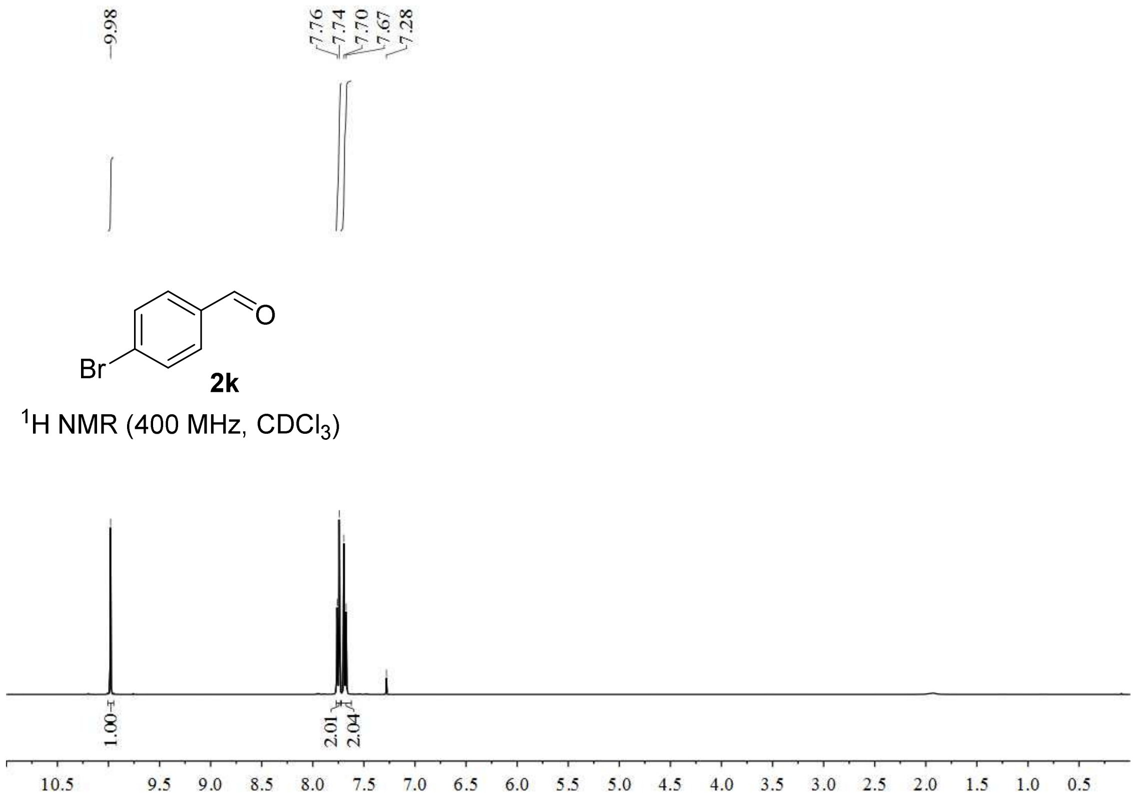

Supplementary Figure 23. ^{1}H NMR (400 MHz, $CDCl_3$) of compound **2k**.

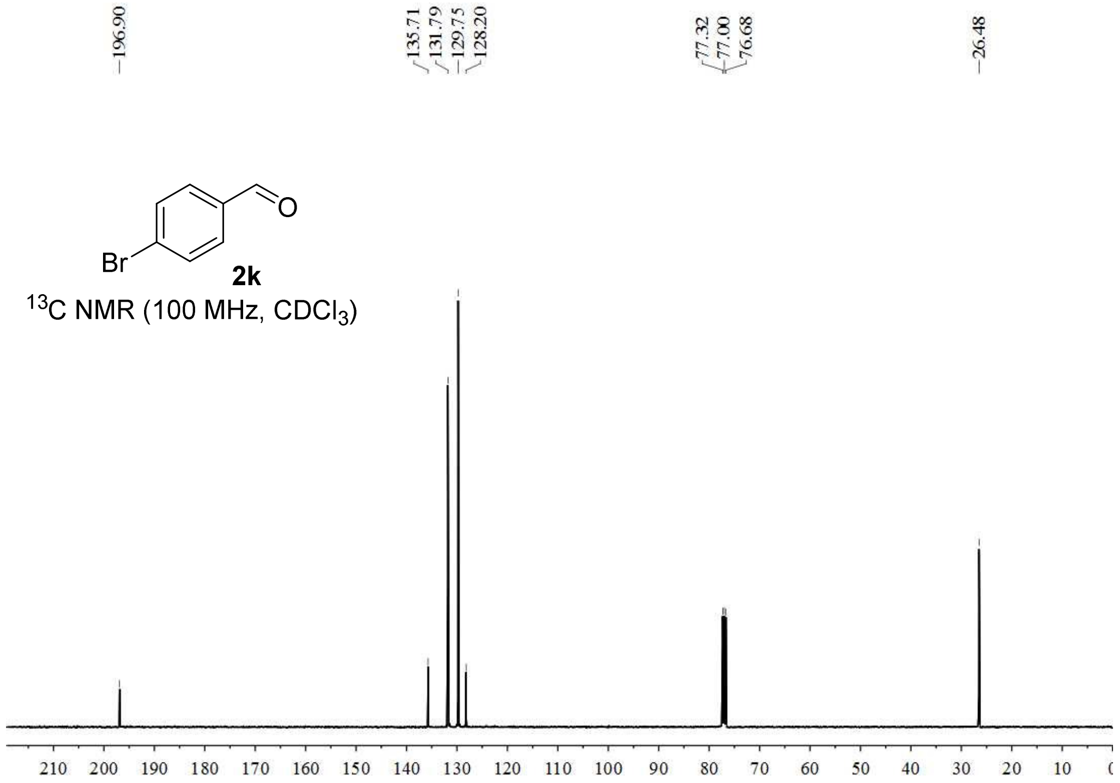

Supplementary Figure 24. ^{13}C NMR (100 MHz, $CDCl_3$) of compound **2k**.

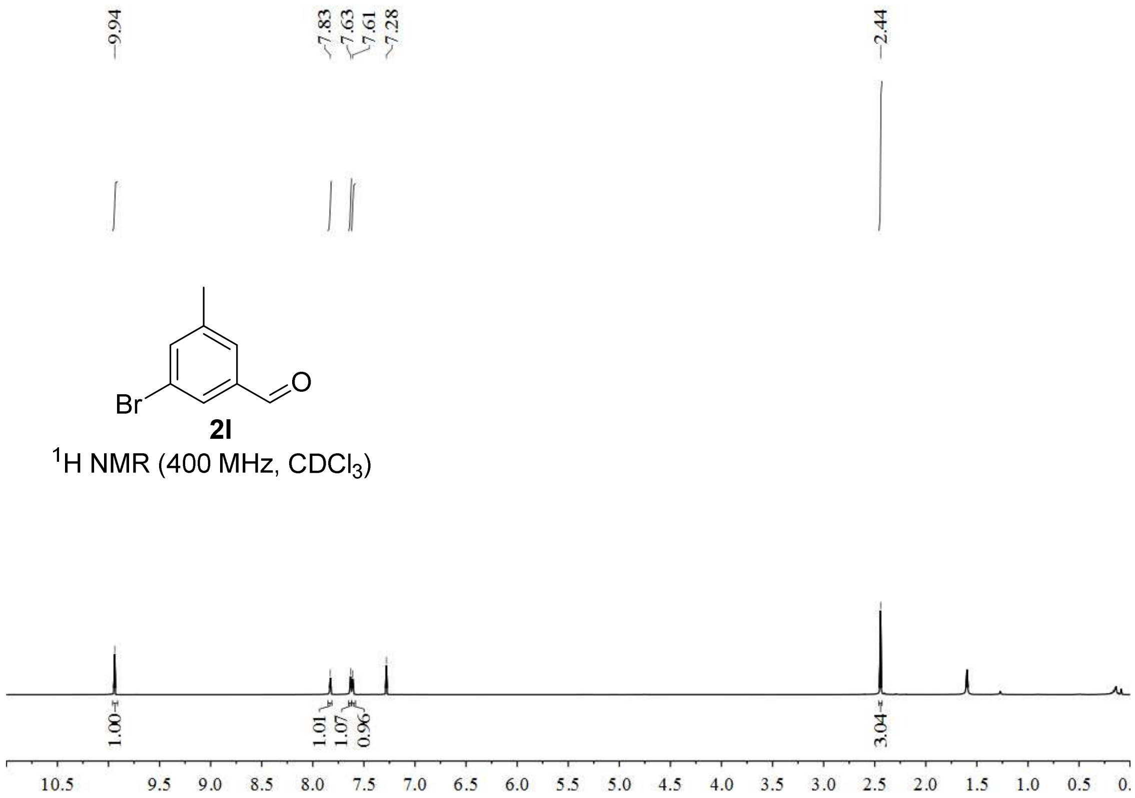

Supplementary Figure 25. ^{1}H NMR (400 MHz, $CDCl_3$) of compound **2l**.

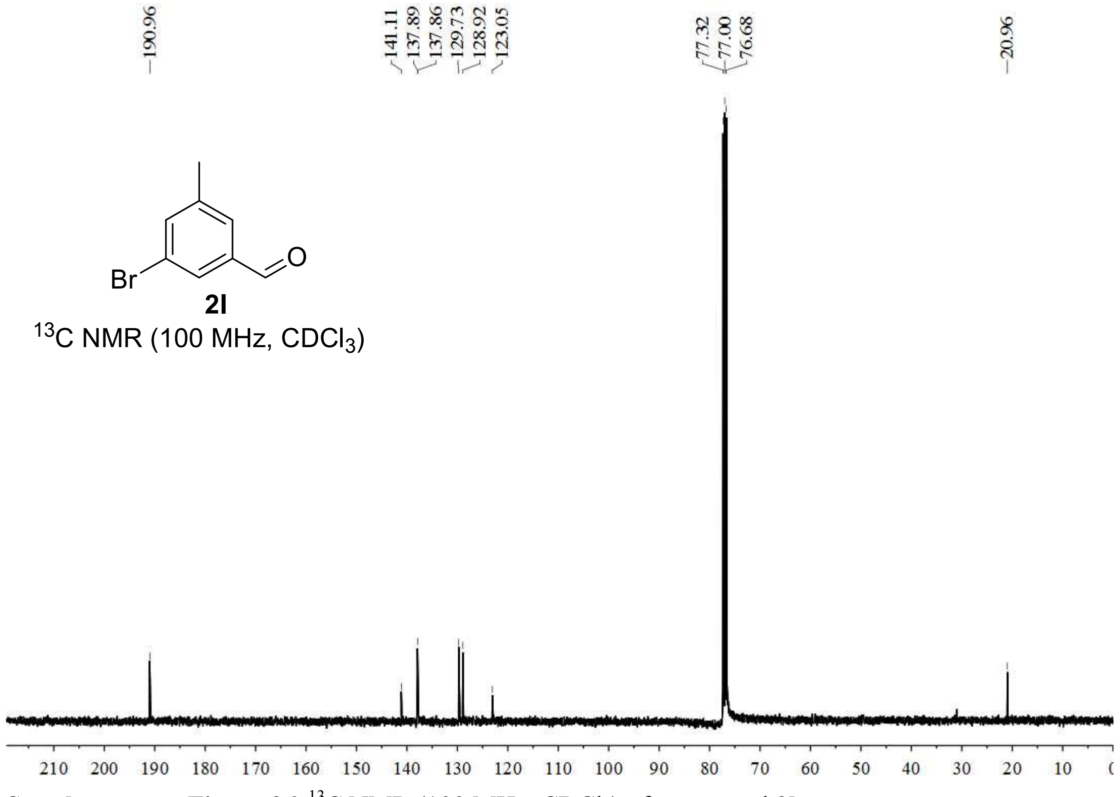

Supplementary Figure 26. ^{13}C NMR (100 MHz, $CDCl_3$) of compound **2l**.

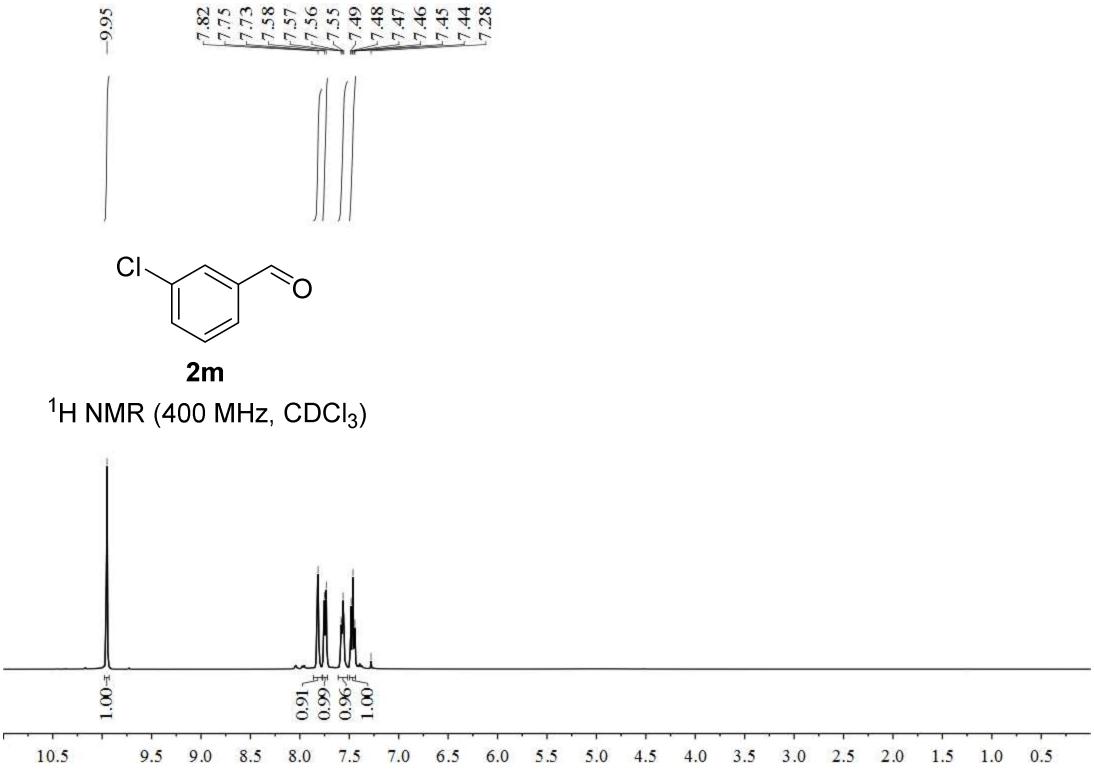

Supplementary Figure 27. ^{1}H NMR (400 MHz, CDCl$_3$) of compound **2m**.

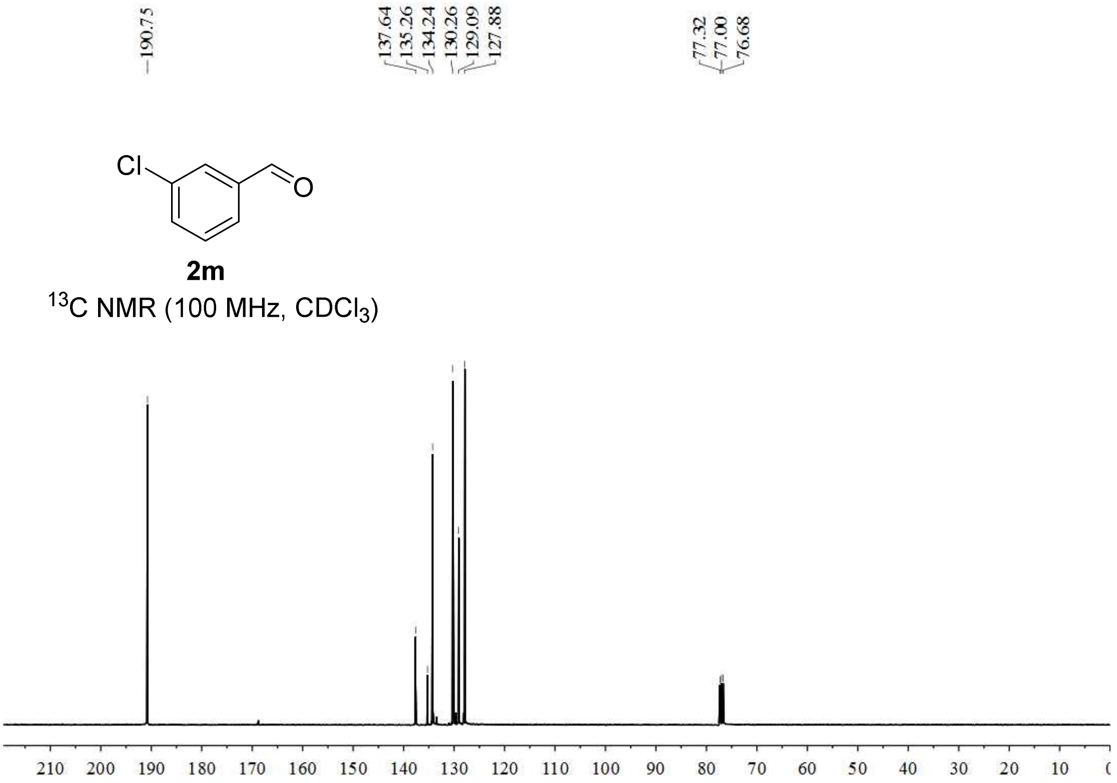

Supplementary Figure 28. ^{13}C NMR (100 MHz, CDCl$_3$) of compound **2m**.

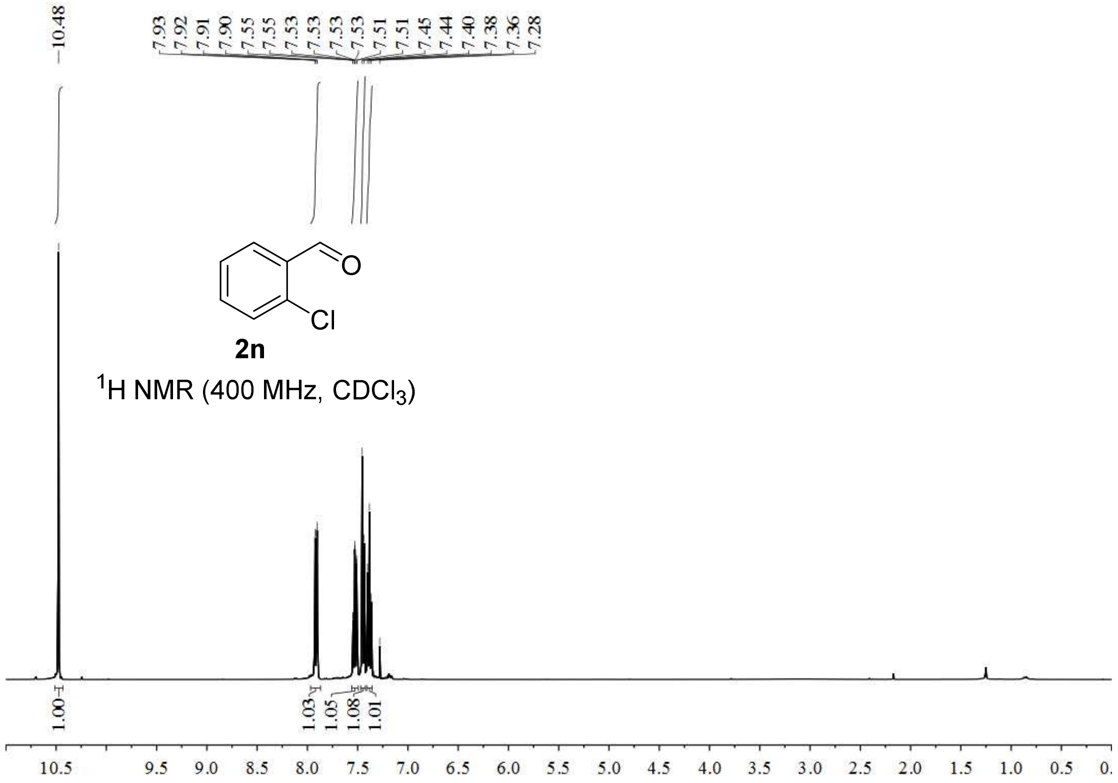

Supplementary Figure 29. ^{1}H NMR (400 MHz, CDCl$_3$) of compound **2n**.

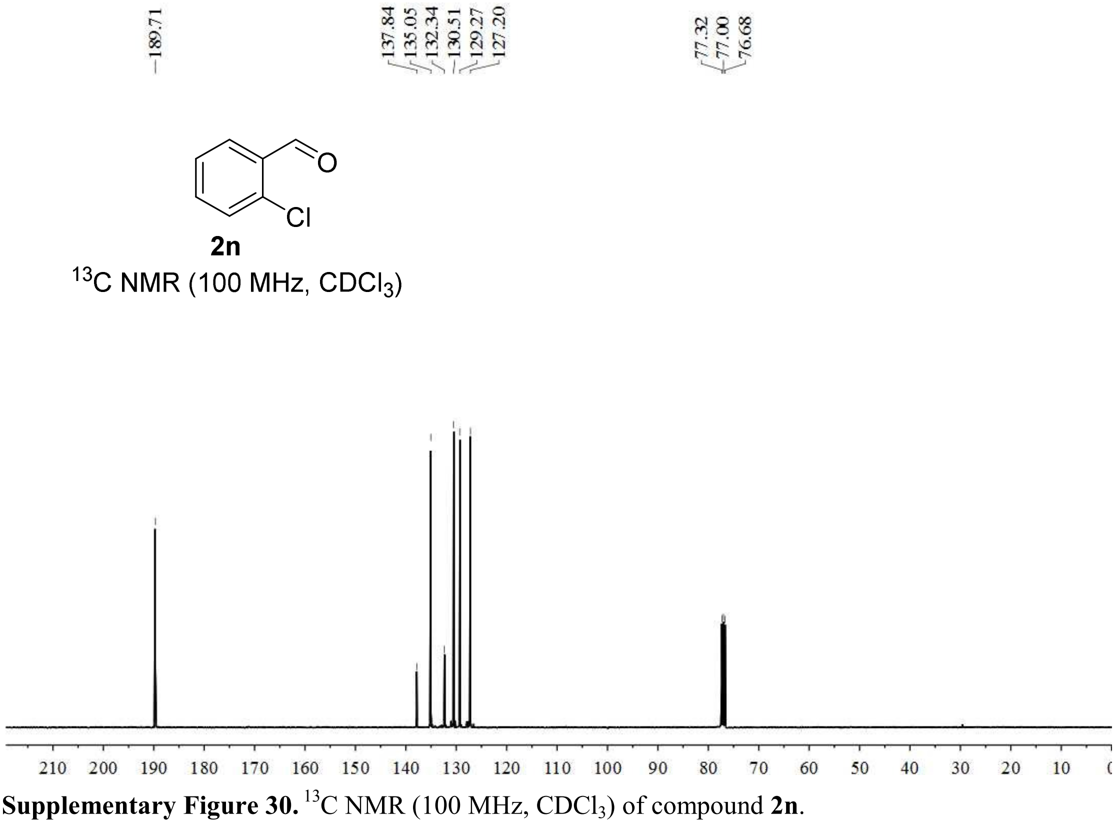

Supplementary Figure 30. ^{13}C NMR (100 MHz, CDCl$_3$) of compound **2n**.

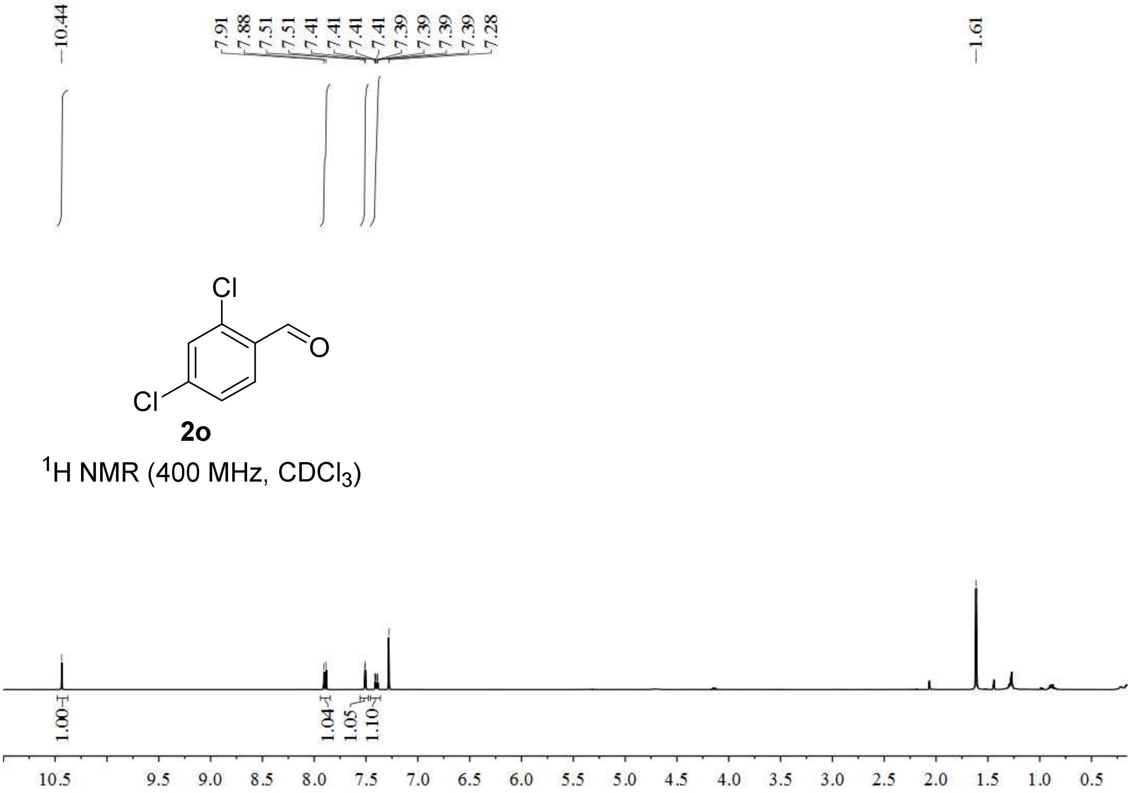

Supplementary Figure 31. ^{1}H NMR (400 MHz, $CDCl_3$) of compound **2o**.

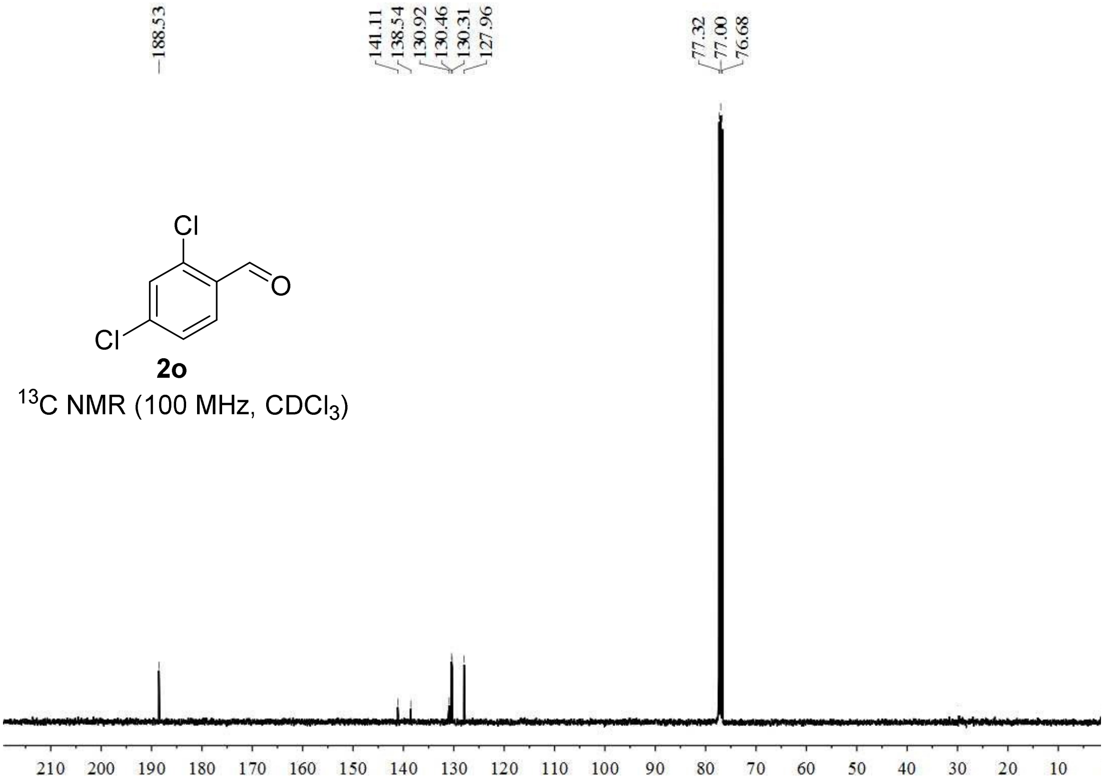

Supplementary Figure 32. ^{13}C NMR (100 MHz, $CDCl_3$) of compound **2o**.

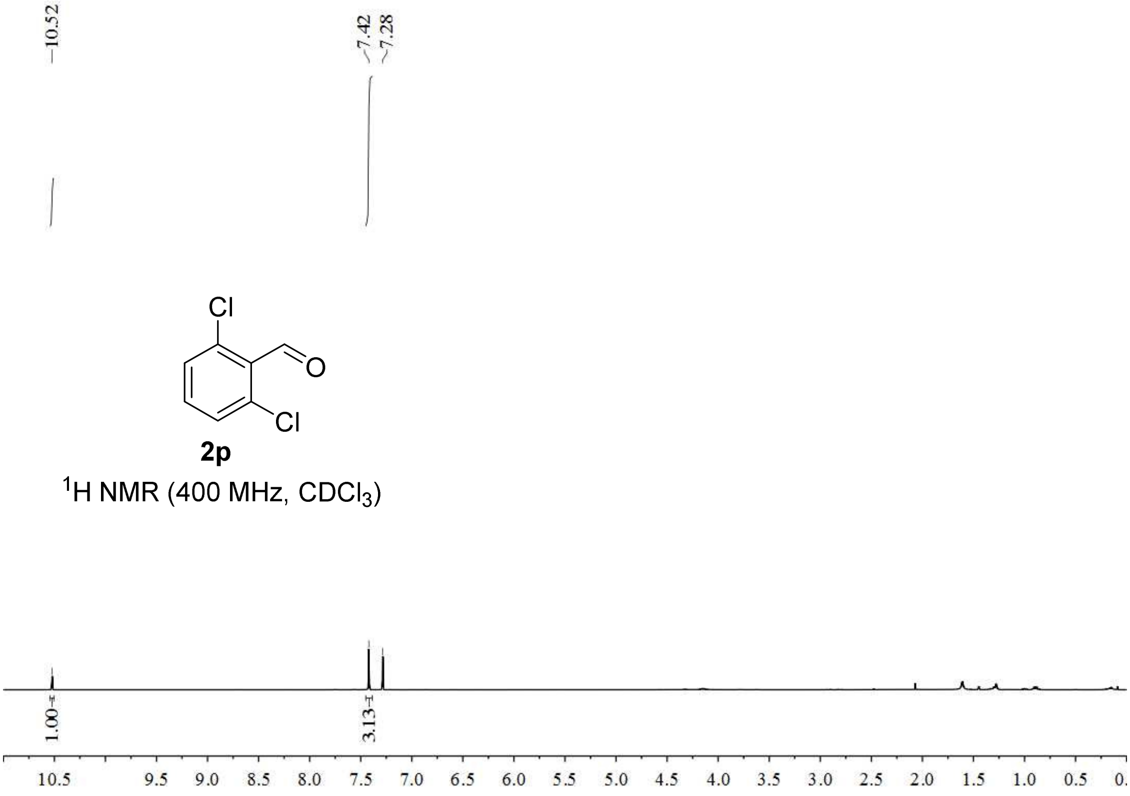

Supplementary Figure 33. ^{1}H NMR (400 MHz, $CDCl_3$) of compound **2p**.

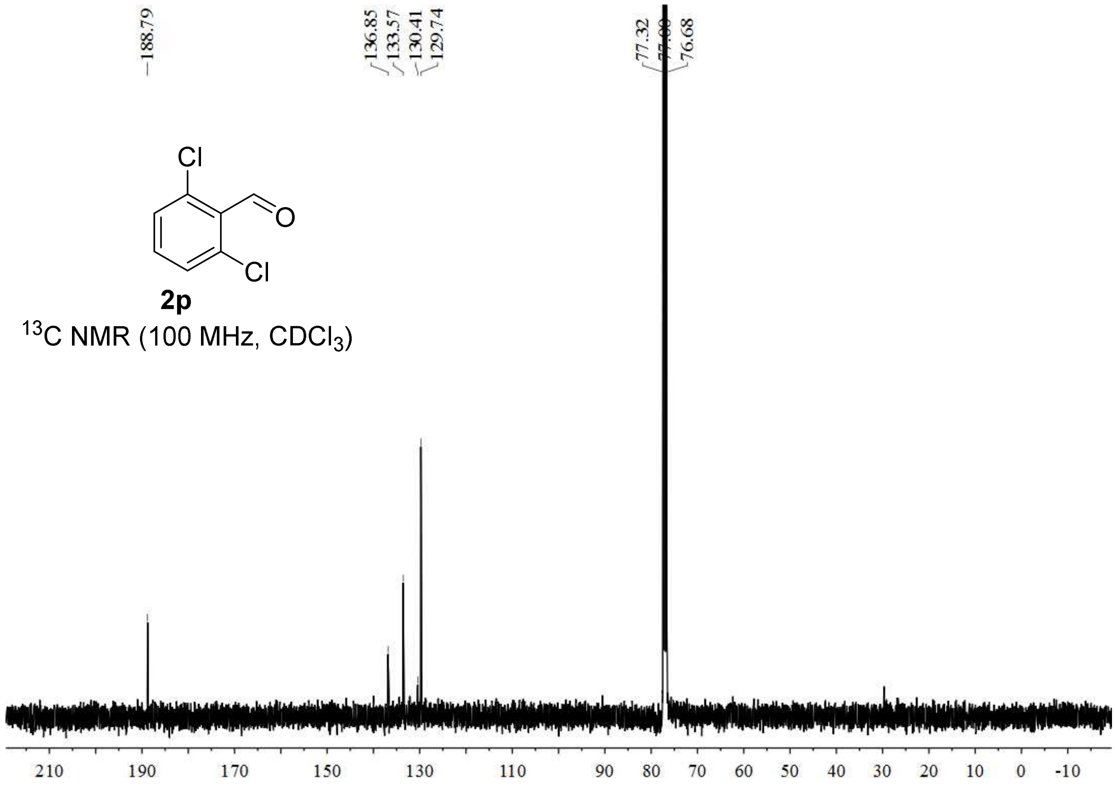

Supplementary Figure 34. ^{13}C NMR (100 MHz, $CDCl_3$) of compound **2p**.

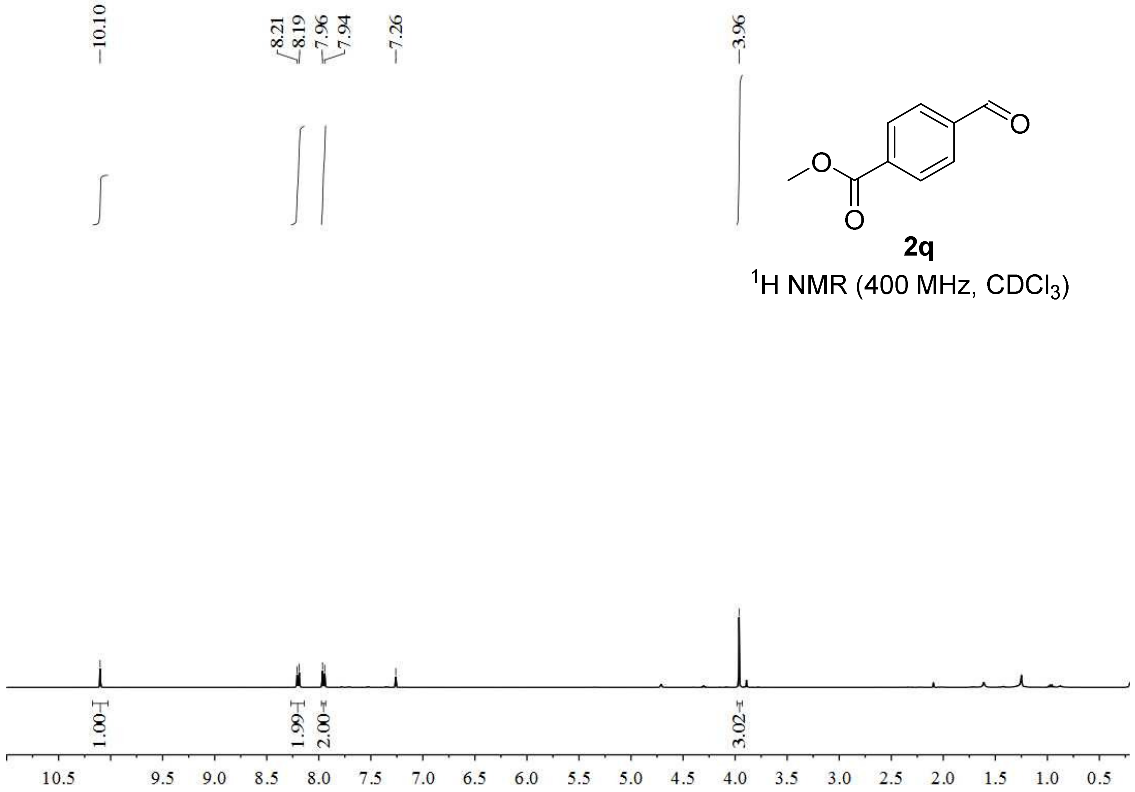

Supplementary Figure 35. ^{1}H NMR (400 MHz, $CDCl_3$) of compound **2q**.

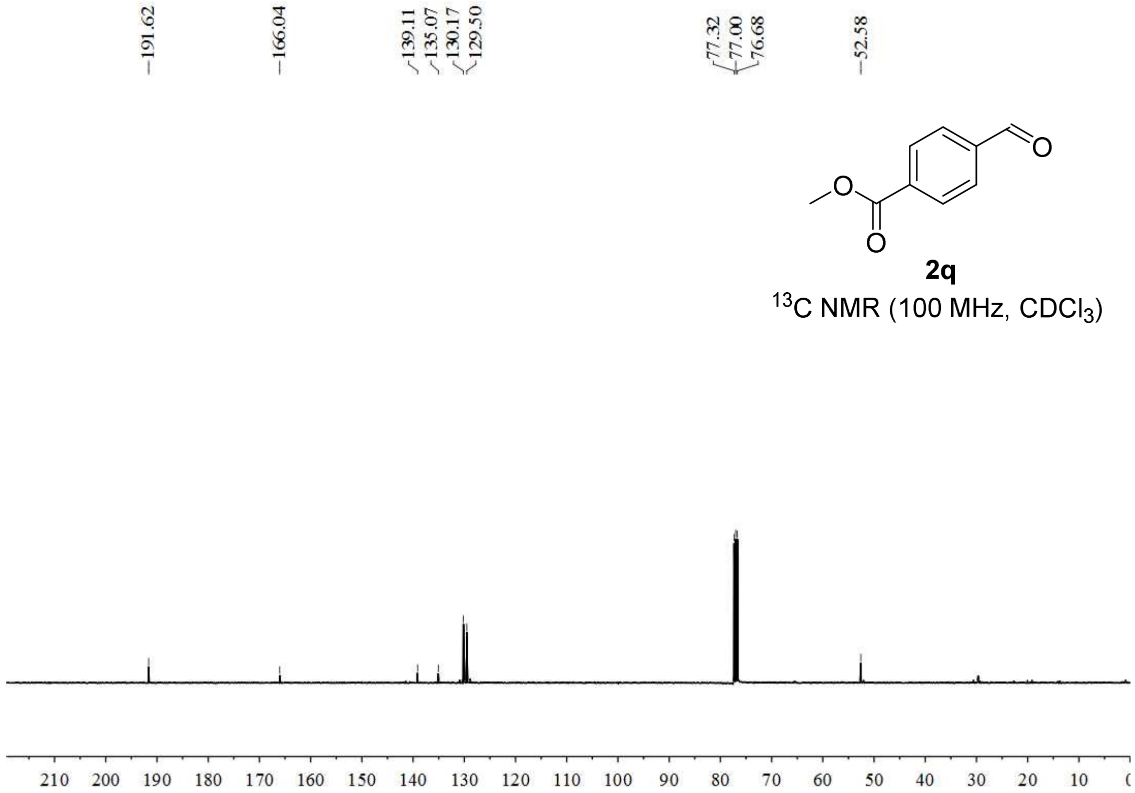

Supplementary Figure 36. ^{13}C NMR (100 MHz, $CDCl_3$) of compound **2q**.

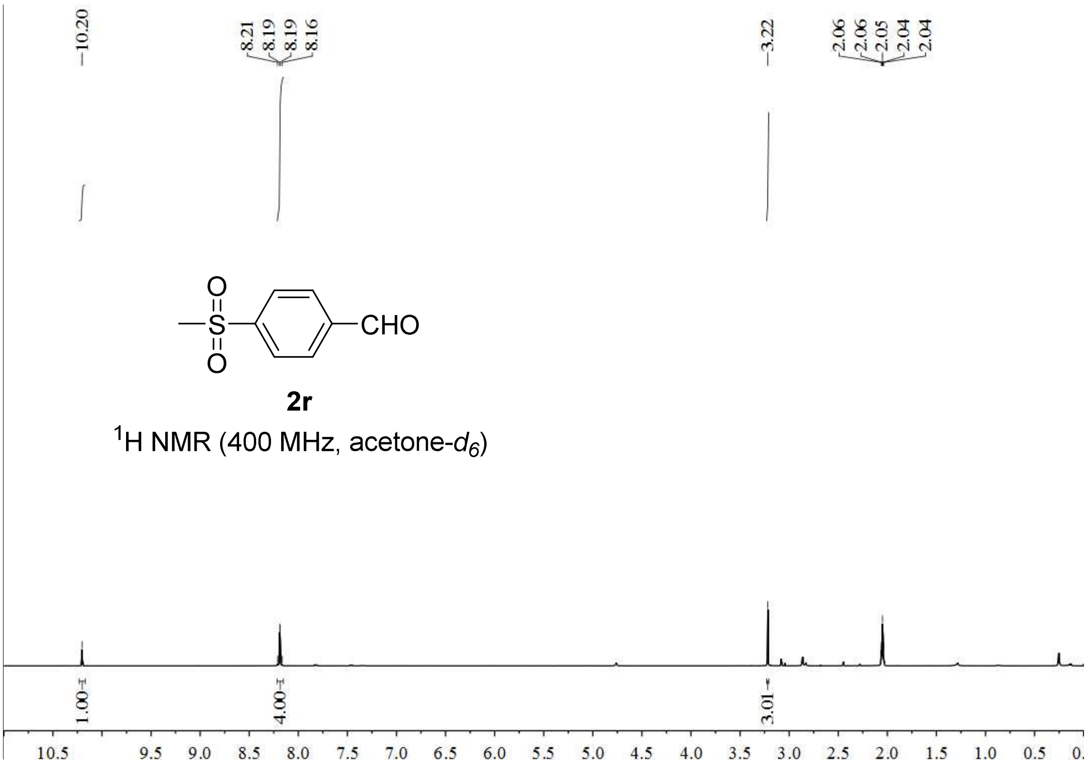

Supplementary Figure 37. ^{1}H NMR (400 MHz, acetone-d_6) of compound **2r**.

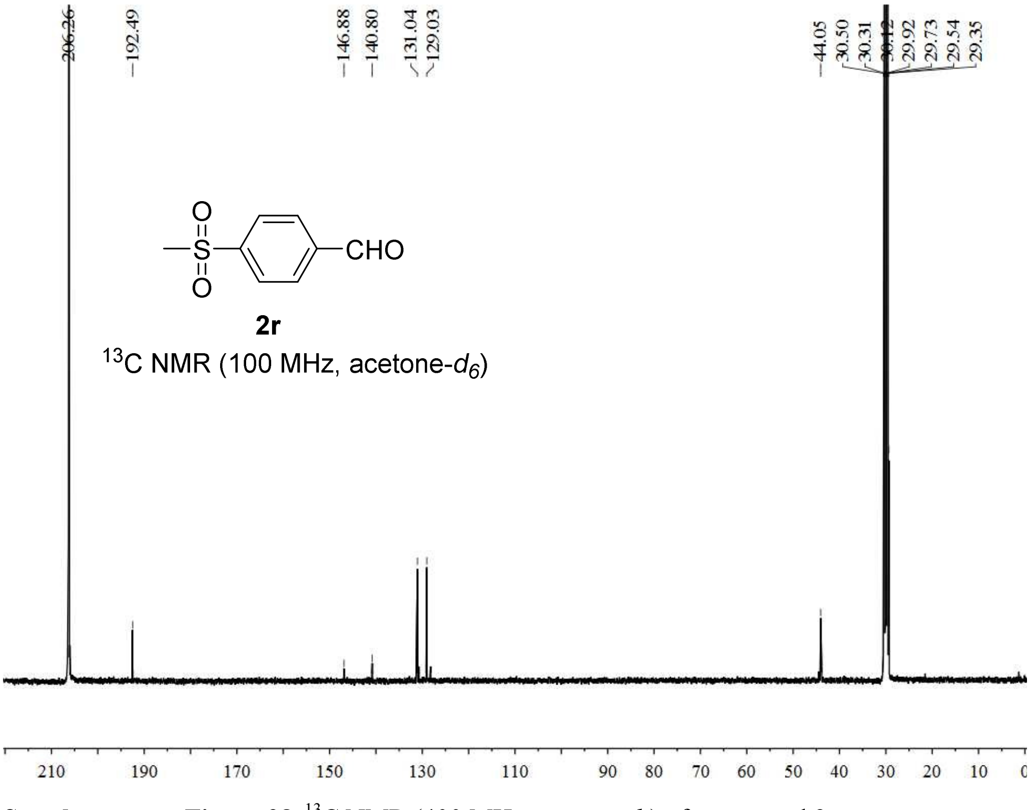

Supplementary Figure 38. ^{13}C NMR (400 MHz, acetone-d_6) of compound **2r**.

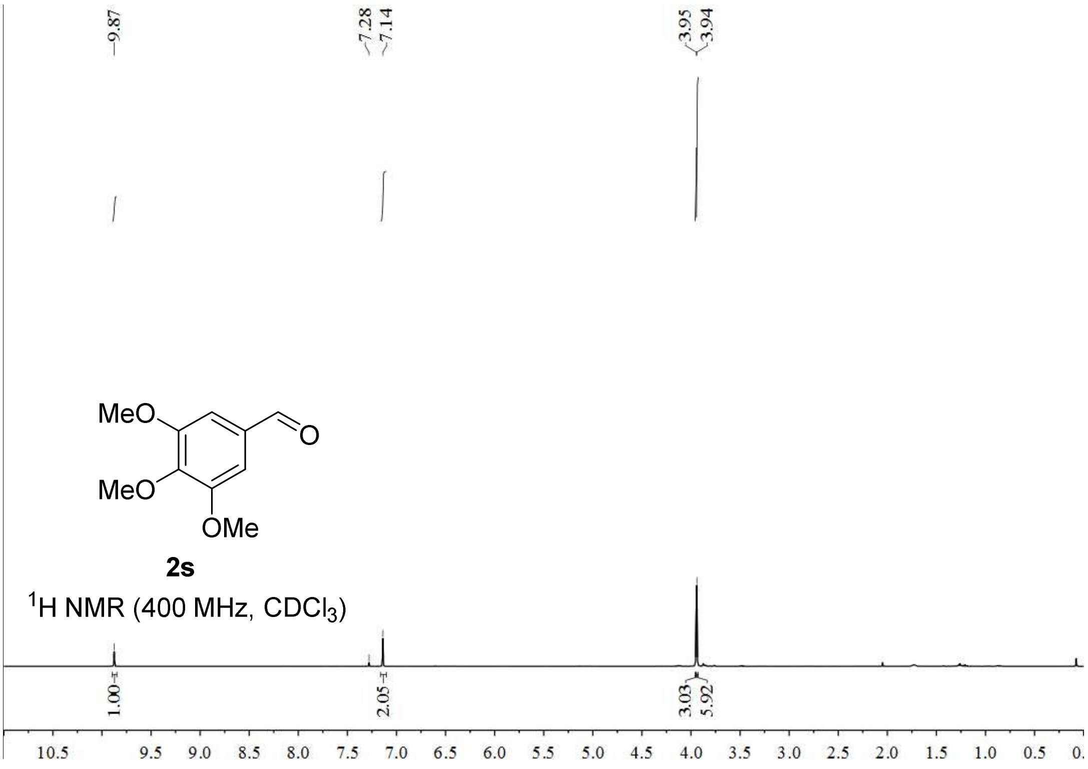

Supplementary Figure 39. ^{1}H NMR (400 MHz, CDCl$_3$) of compound **2s**.

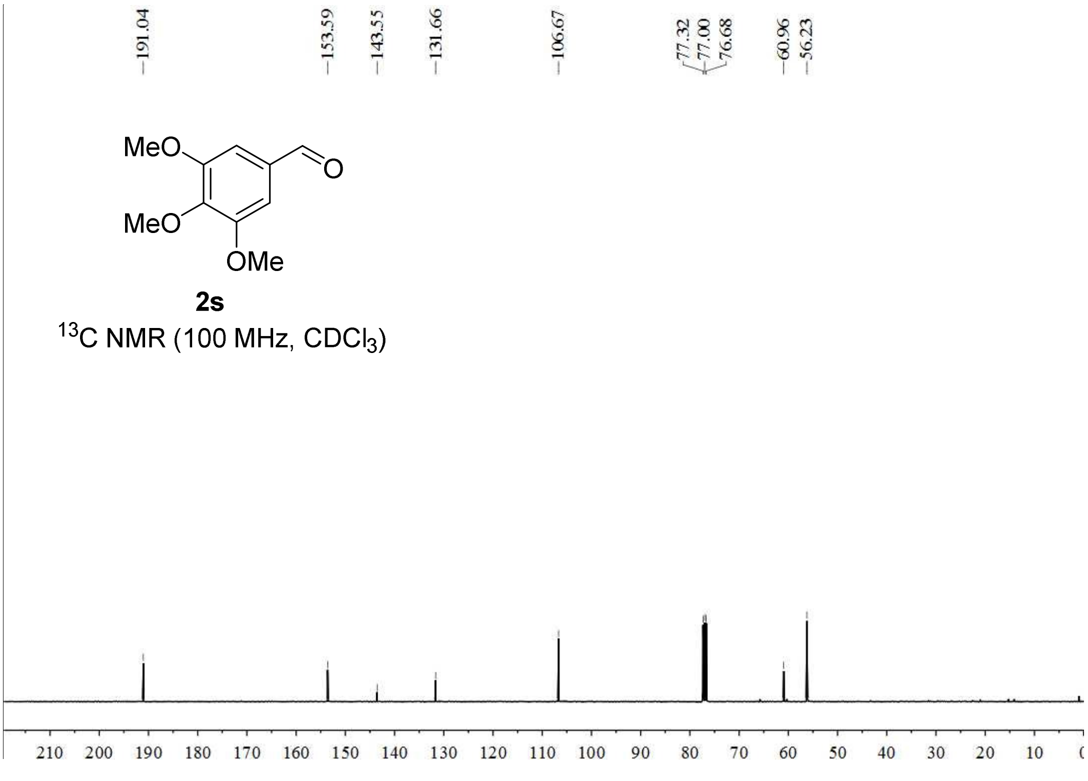

Supplementary Figure 40. ^{13}C NMR (100 MHz, CDCl$_3$) of compound **2s**.

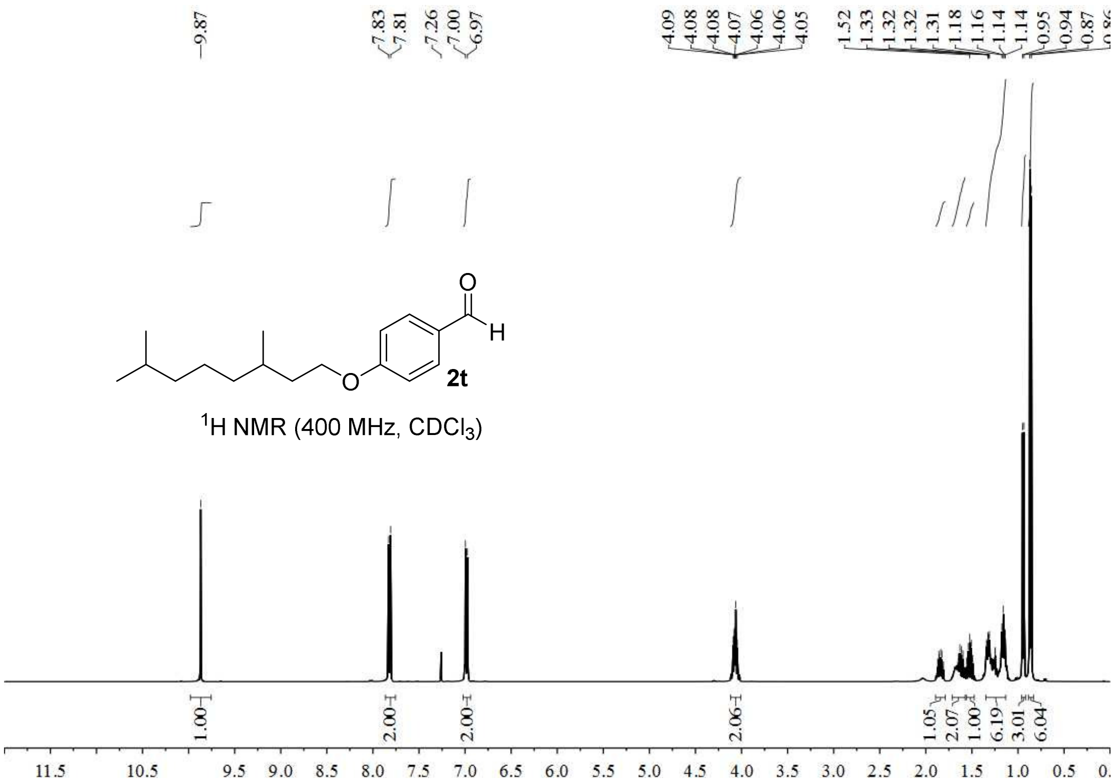

Supplementary Figure 41. ^{1}H NMR (400 MHz, $CDCl_3$) of compound **2t**.

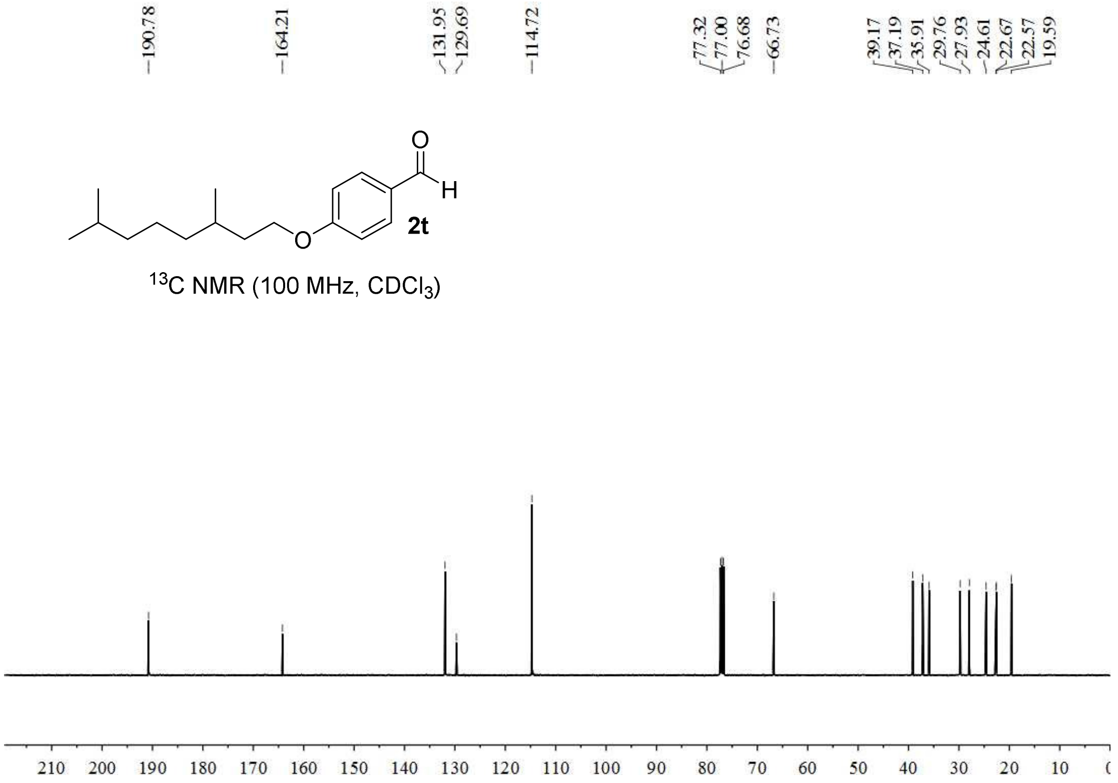

Supplementary Figure 42. ^{13}C NMR (100 MHz, $CDCl_3$) of compound **2t**.

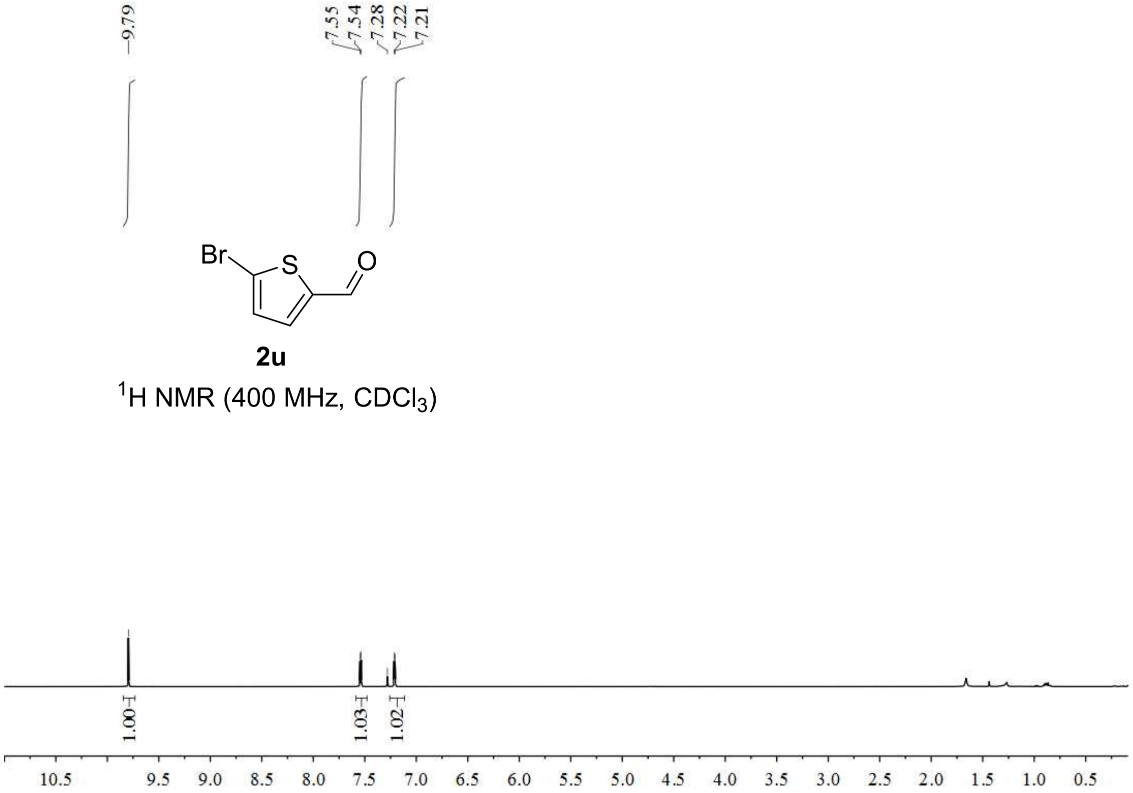

Supplementary Figure 43. ^{1}H NMR (400 MHz, $CDCl_3$) of compound **2u**.

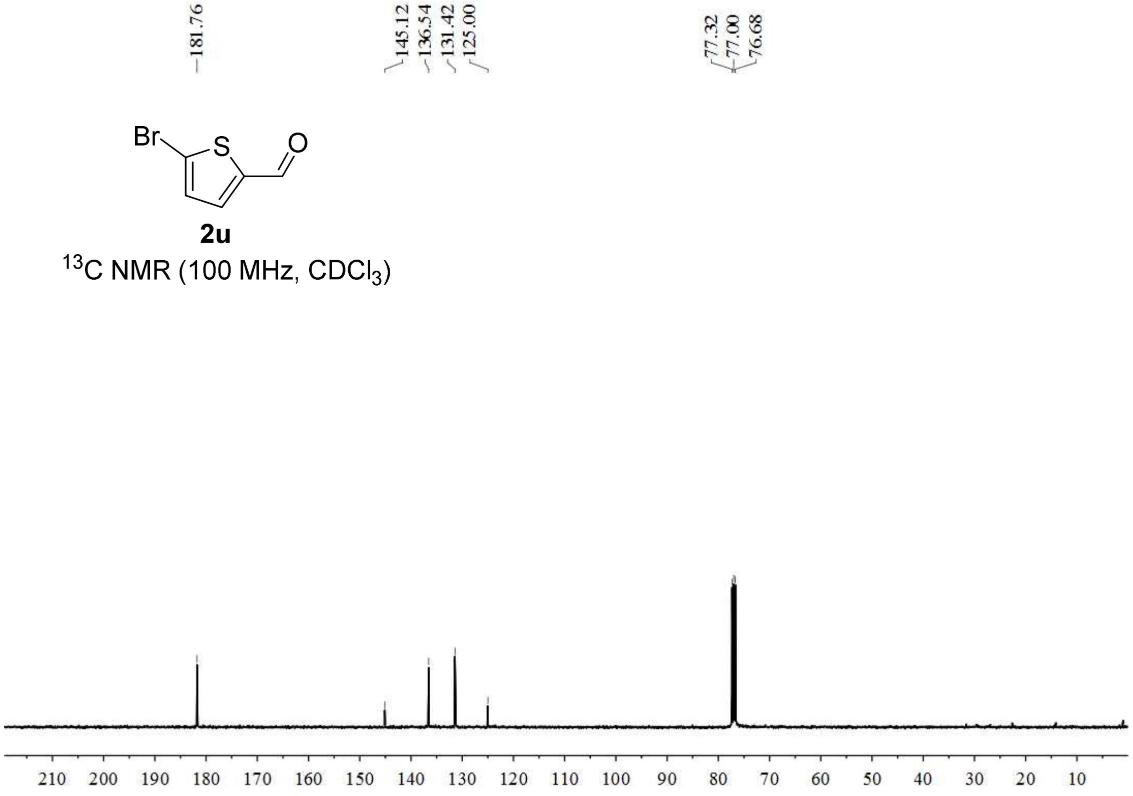

Supplementary Figure 44. ^{13}C NMR (100 MHz, $CDCl_3$) of compound **2u**.

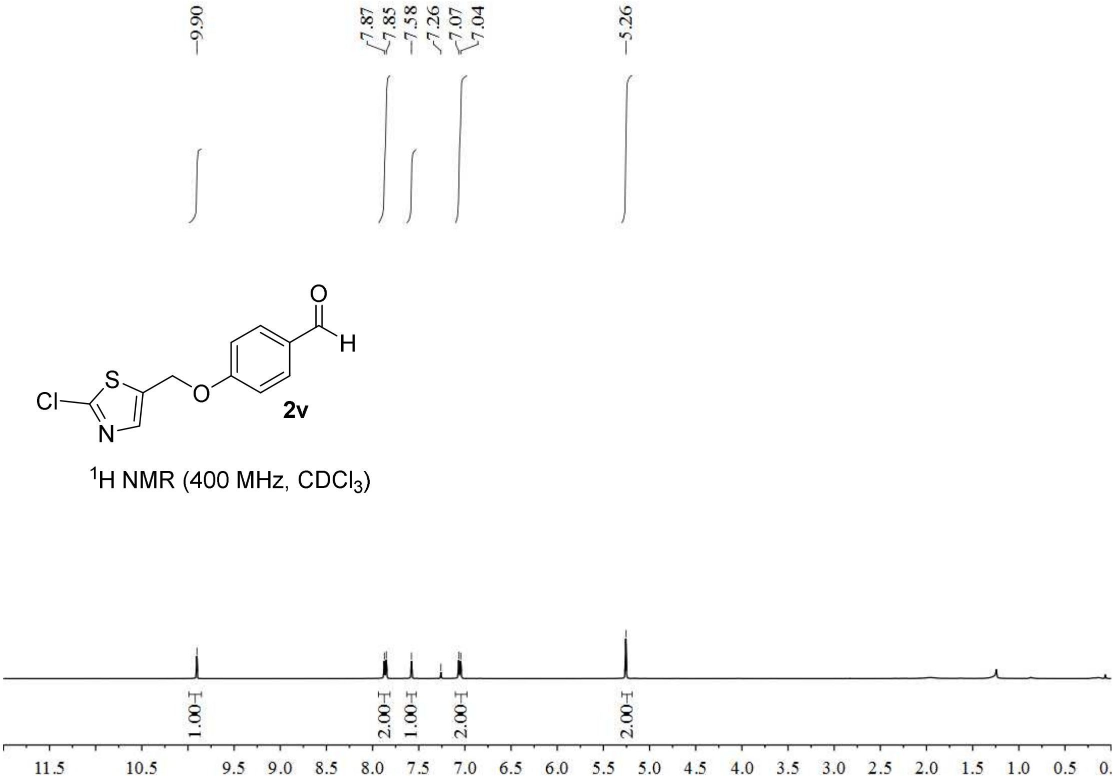

Supplementary Figure 45. ^{1}H NMR (400 MHz, $CDCl_3$) of compound **2v**.

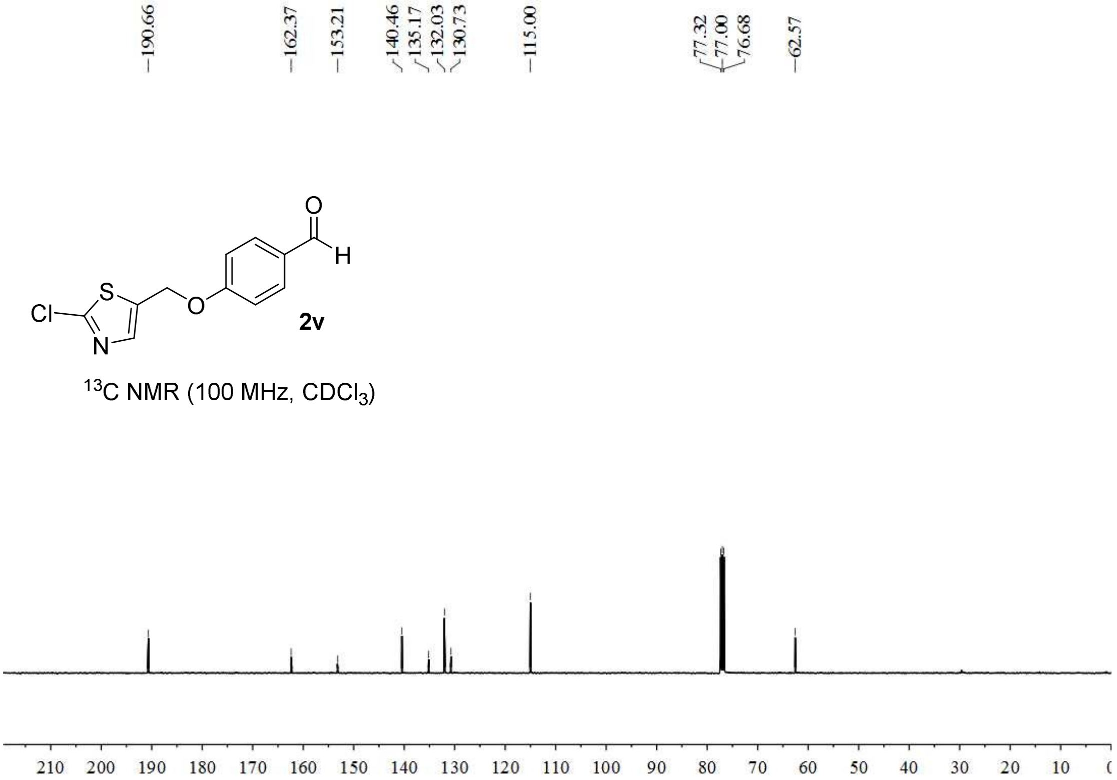

Supplementary Figure 46. ^{13}C NMR (100 MHz, $CDCl_3$) of compound **2v**.

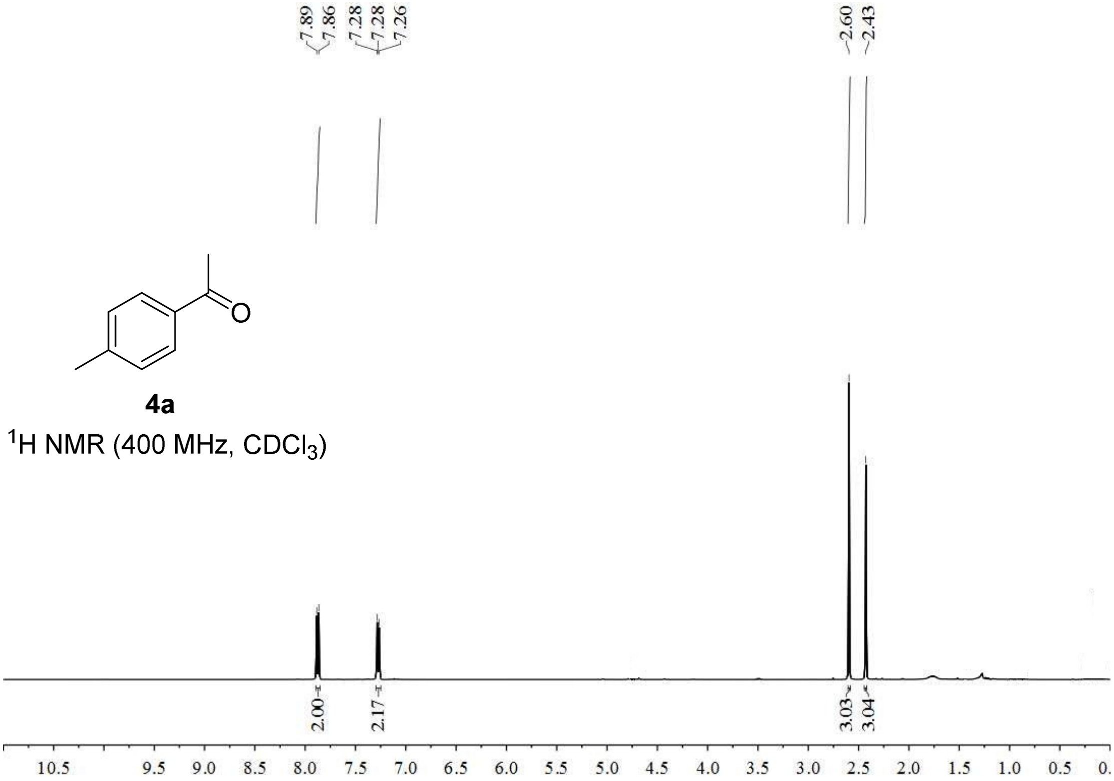

Supplementary Figure 47. ^{1}H NMR (400 MHz, $CDCl_3$) of compound **4a**.

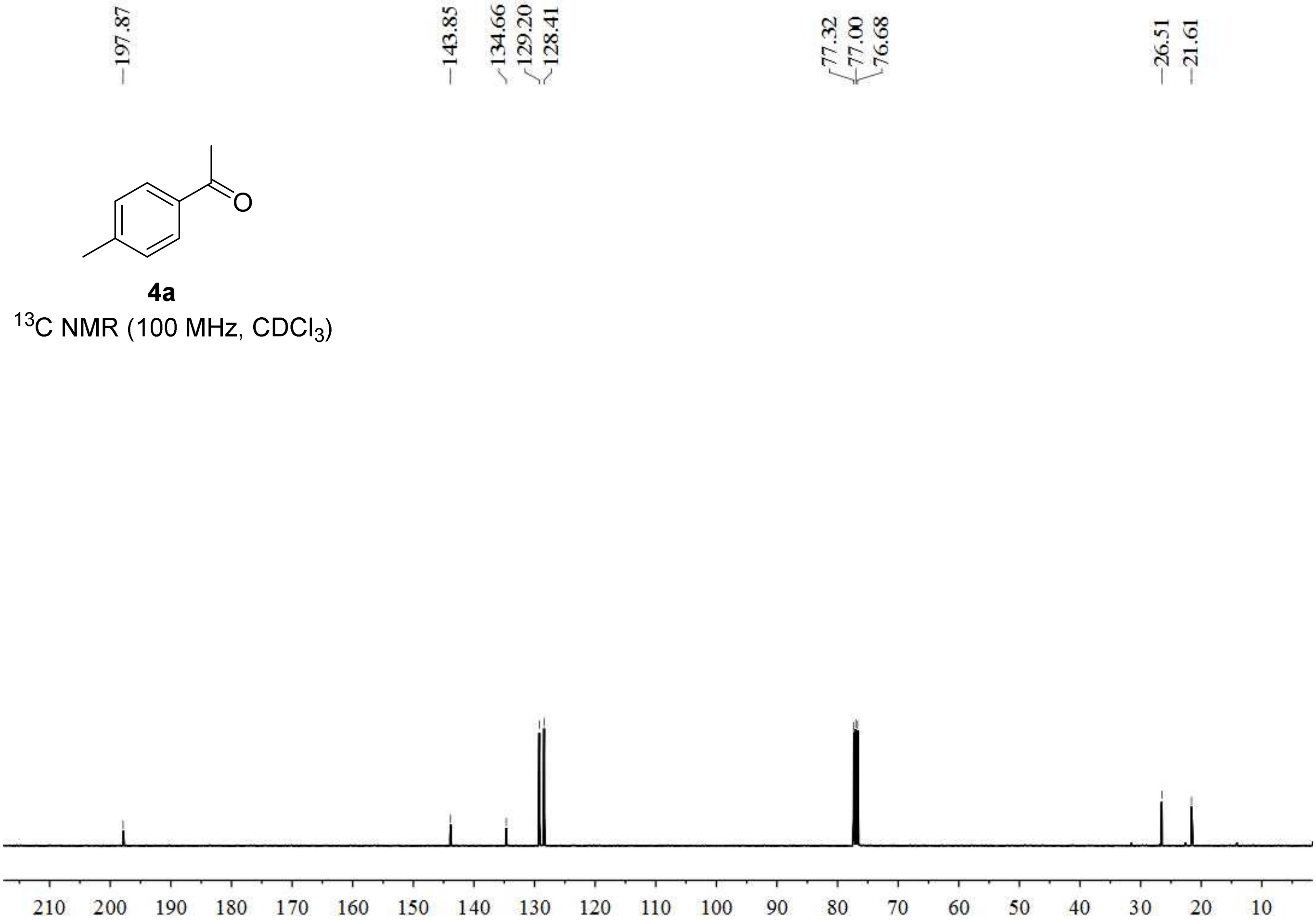

Supplementary Figure 48. ^{13}C NMR (100 MHz, $CDCl_3$) of compound **4a**.

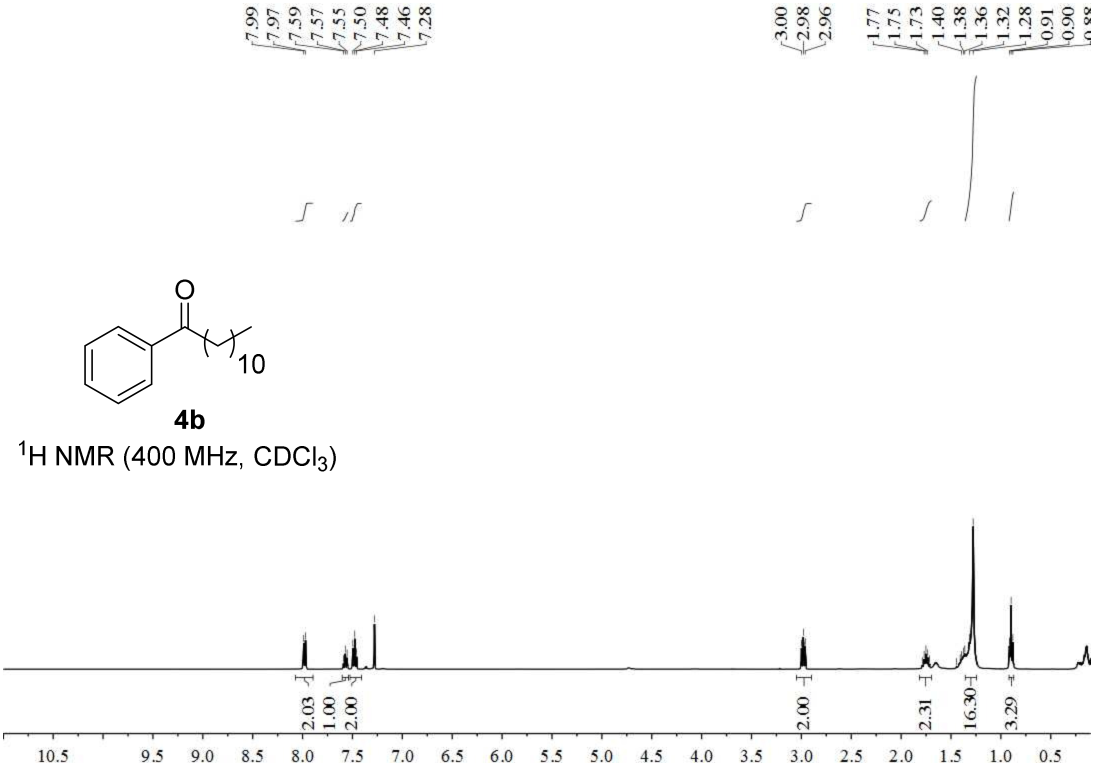

Supplementary Figure 49. ^{1}H NMR (400 MHz, $CDCl_3$) of compound **4b**.

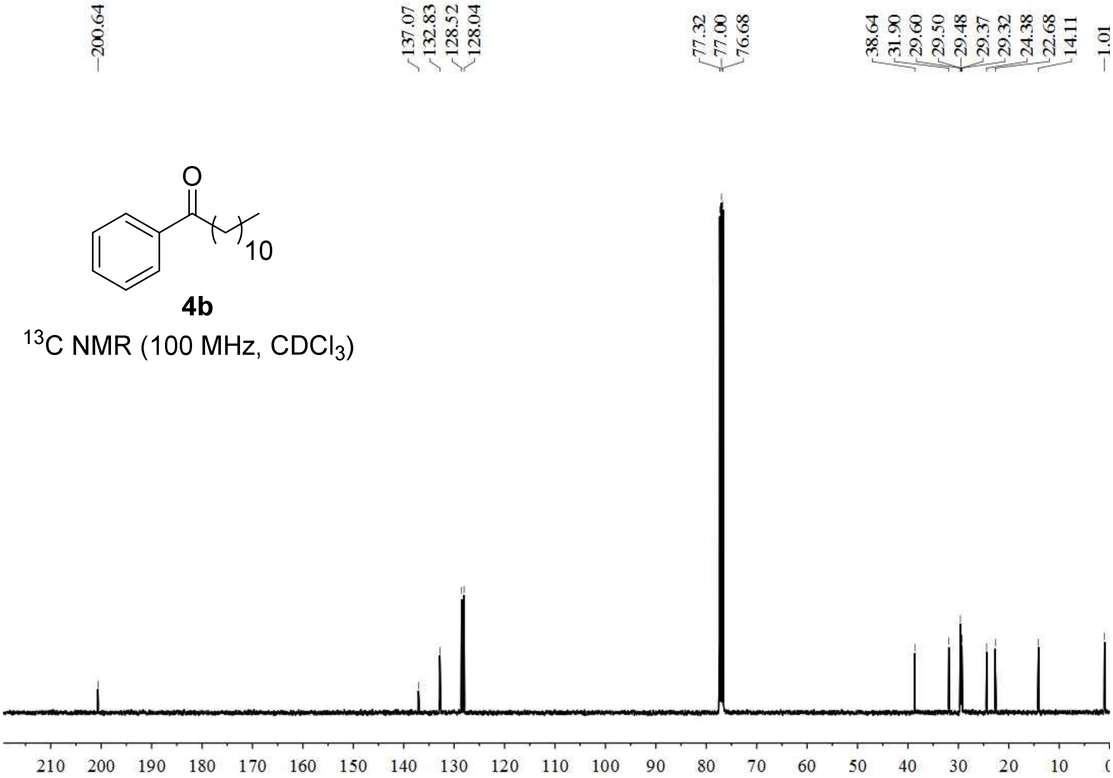

Supplementary Figure 50. ^{13}C NMR (100 MHz, $CDCl_3$) of compound **4b**.

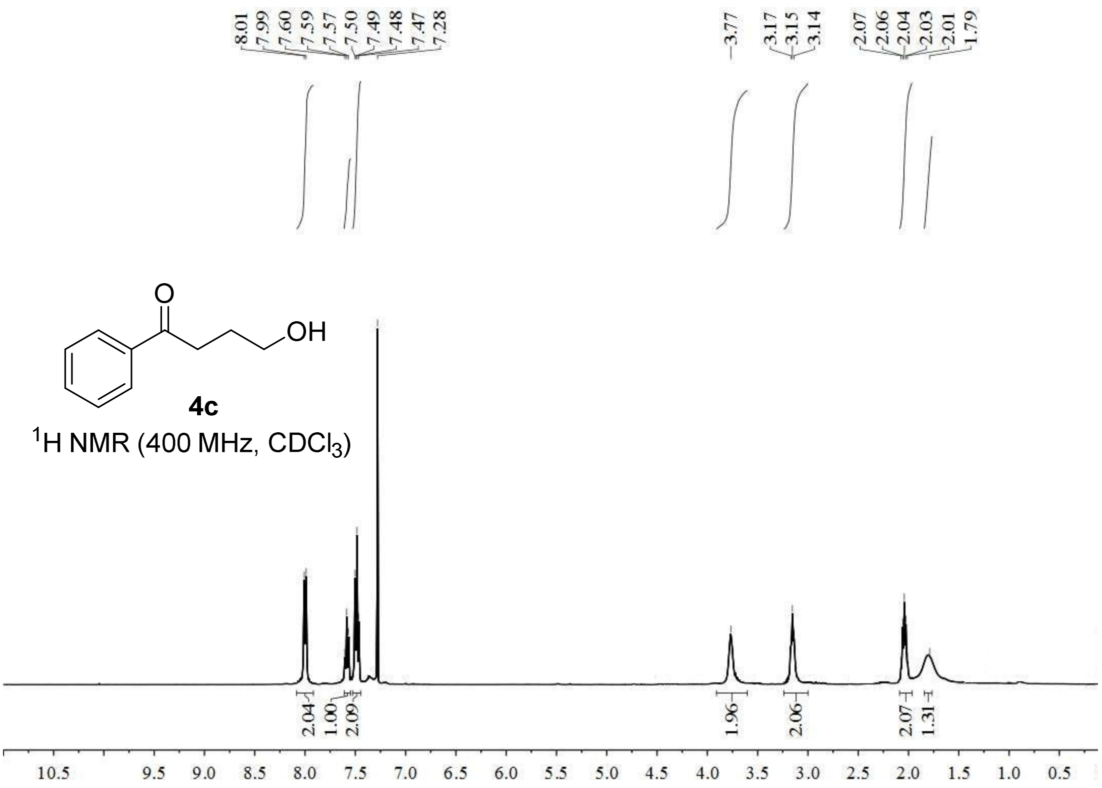

Supplementary Figure 51. ^{1}H NMR (400 MHz, $CDCl_3$) of compound **4c**.

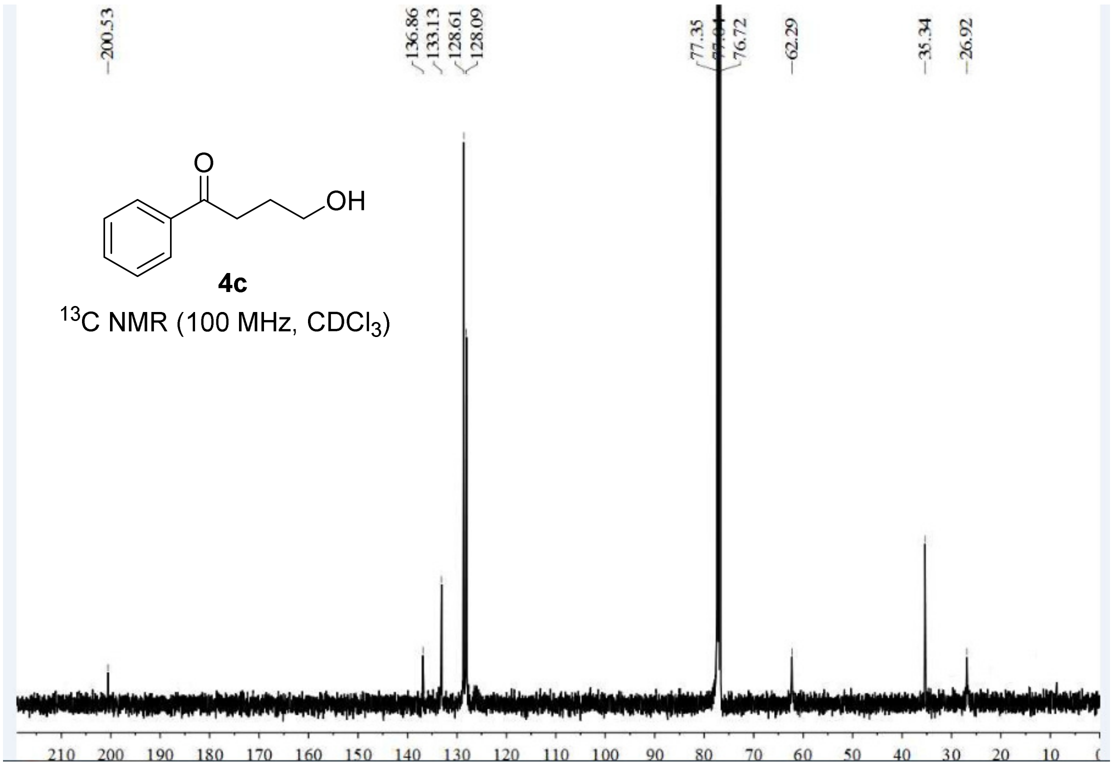

Supplementary Figure 52. ^{13}C NMR (100 MHz, $CDCl_3$) of compound **4c**.

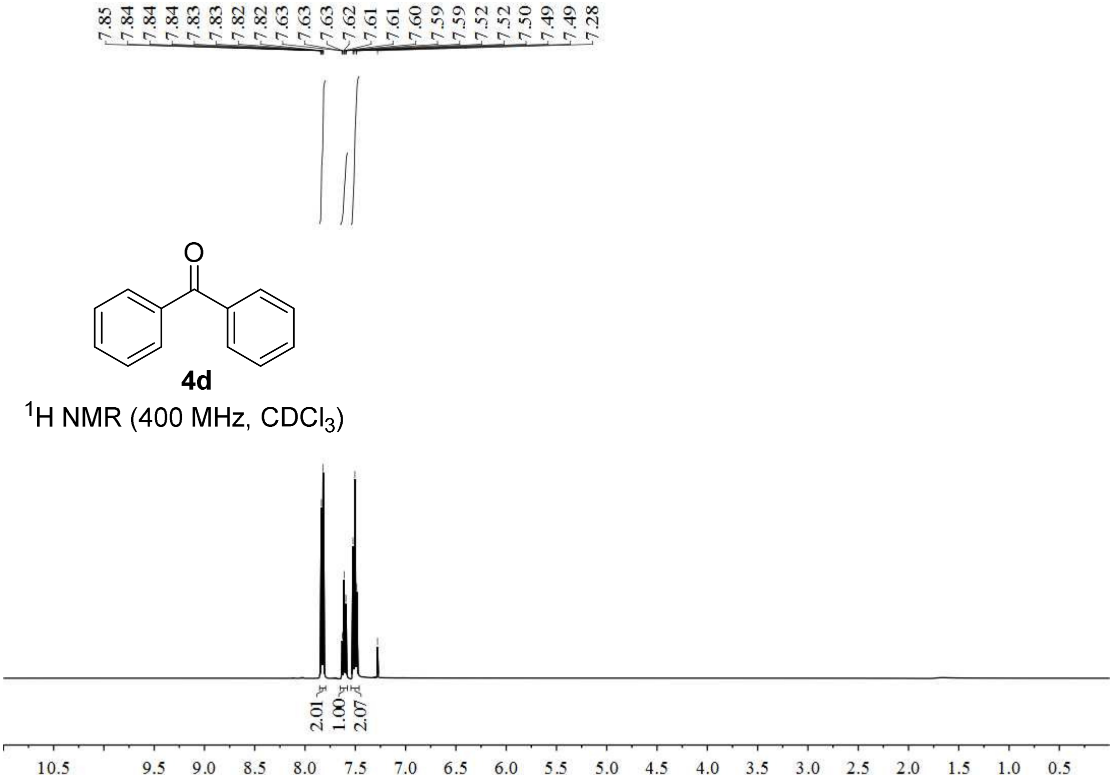

Supplementary Figure 53. ^{1}H NMR (400 MHz, CDCl$_3$) of compound **4d**.

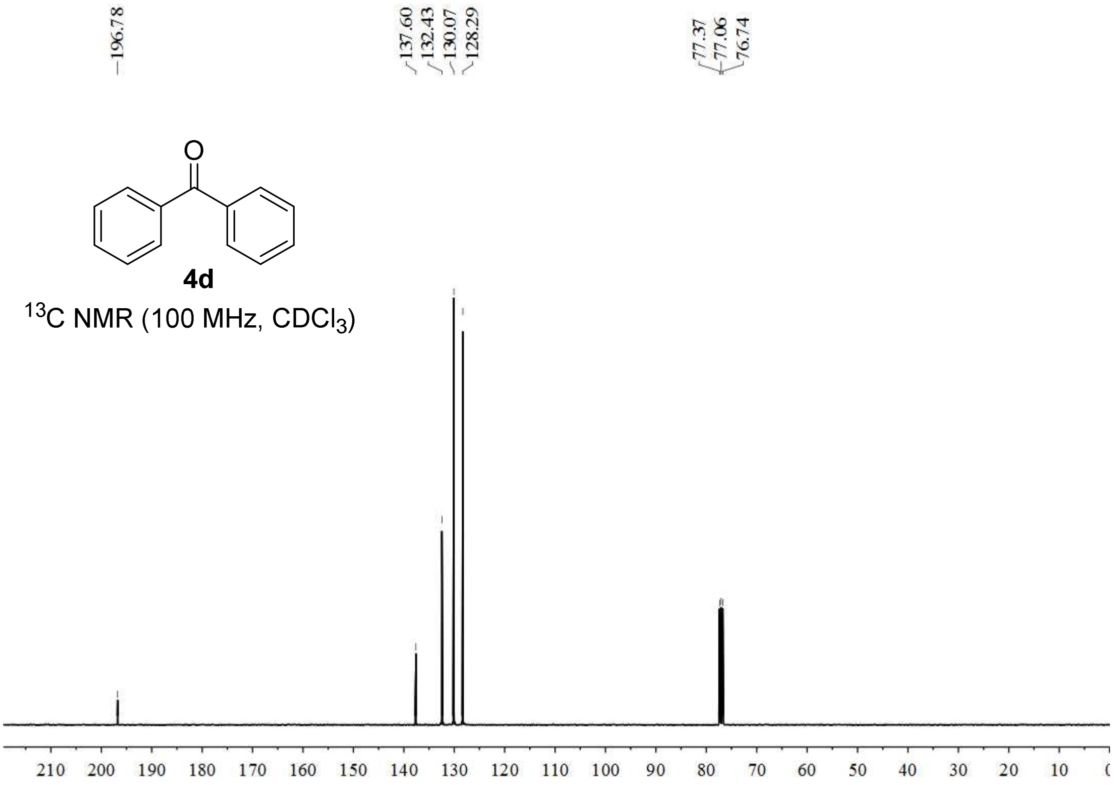

Supplementary Figure 54. ^{13}C NMR (100 MHz, CDCl$_3$) of compound **4d**.

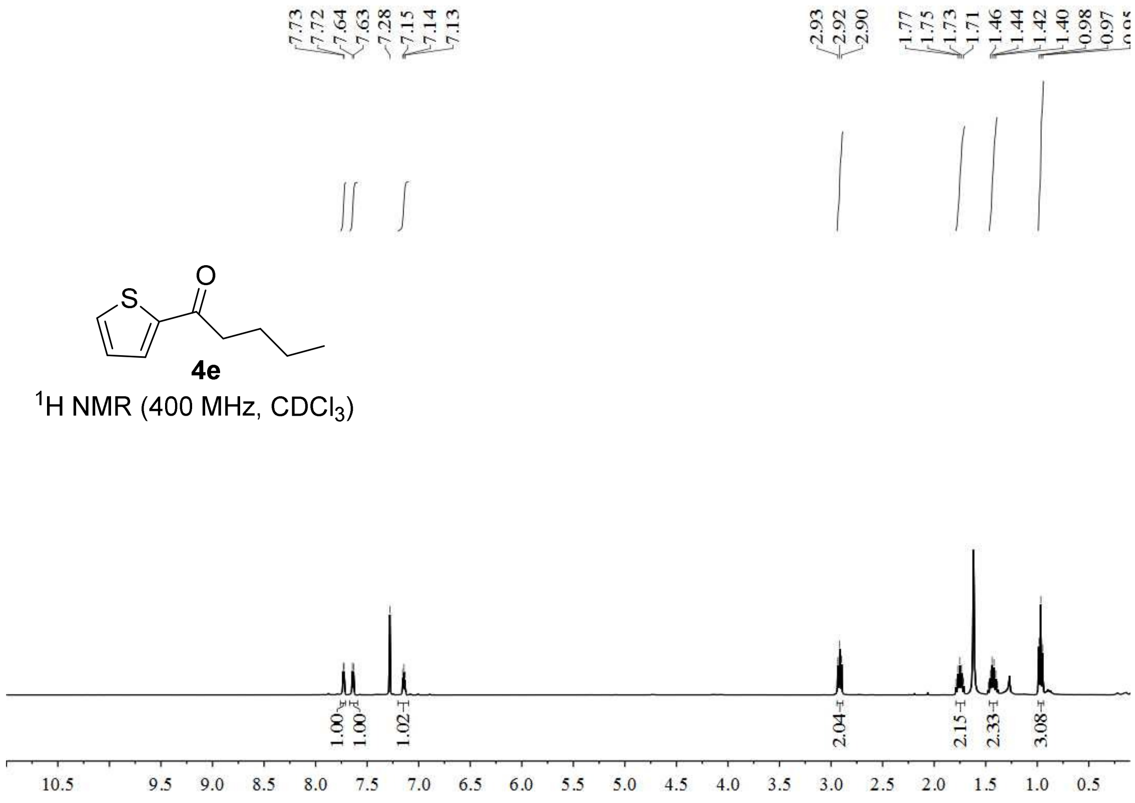

Supplementary Figure 55. ^{1}H NMR (400 MHz, $CDCl_3$) of compound **4e**.

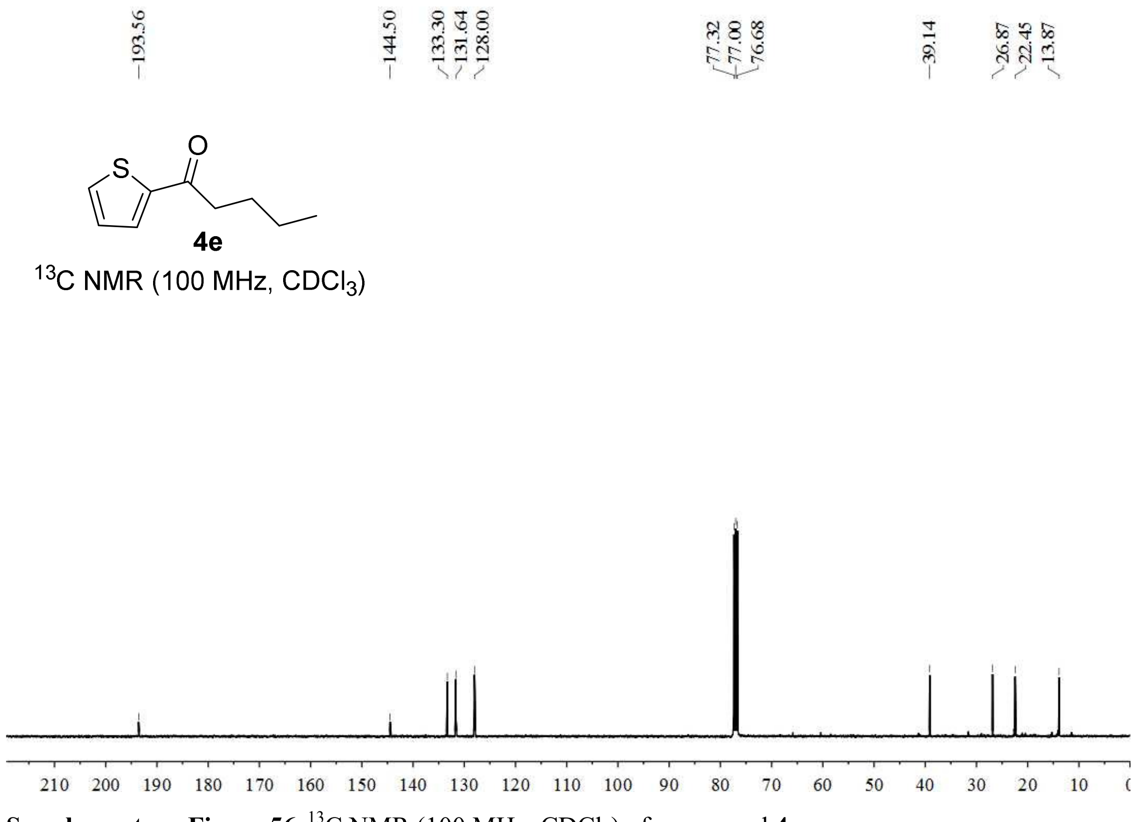

Supplementary Figure 56. ^{13}C NMR (100 MHz, $CDCl_3$) of compound **4e**.

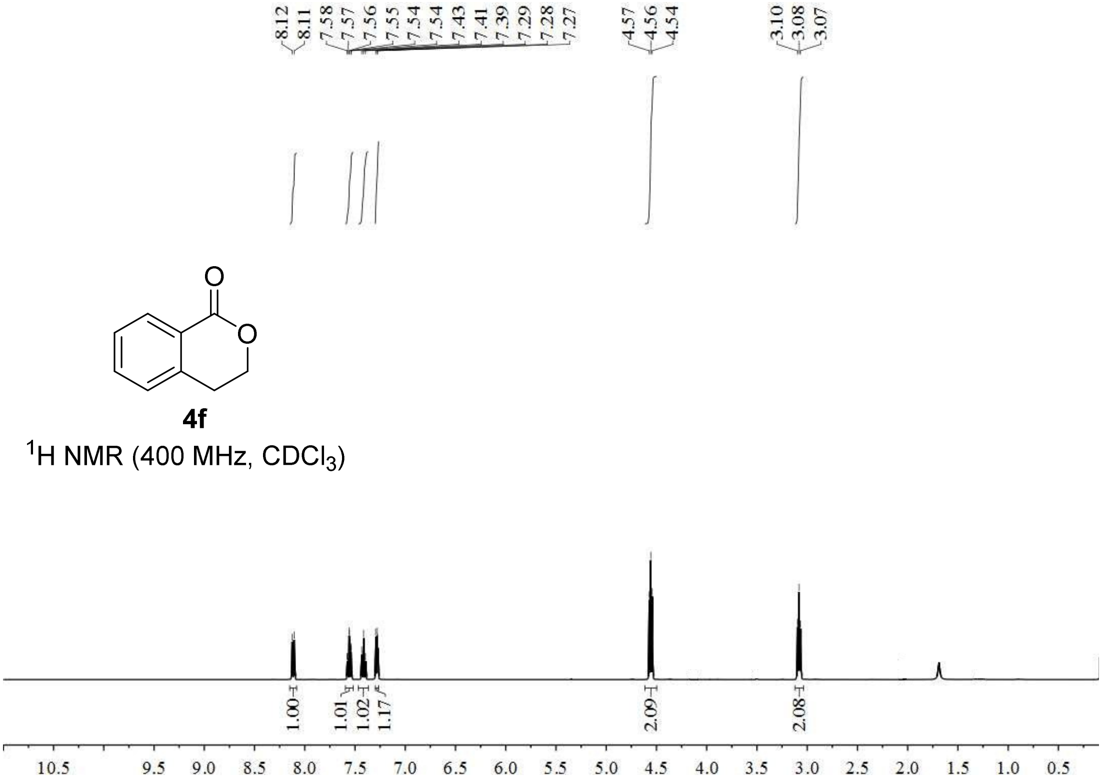

Supplementary Figure 57. ^{1}H NMR (400 MHz, $CDCl_3$) of compound **4f**.

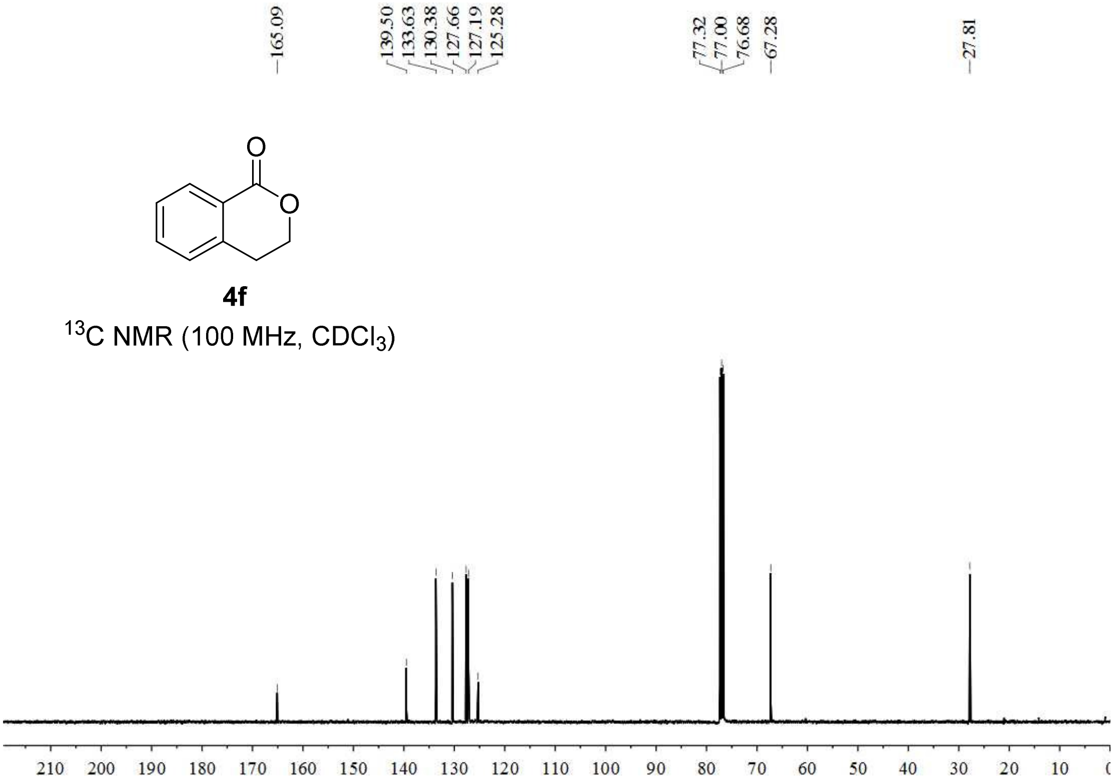

Supplementary Figure 58. ^{13}C NMR (100 MHz, $CDCl_3$) of compound **4f**.

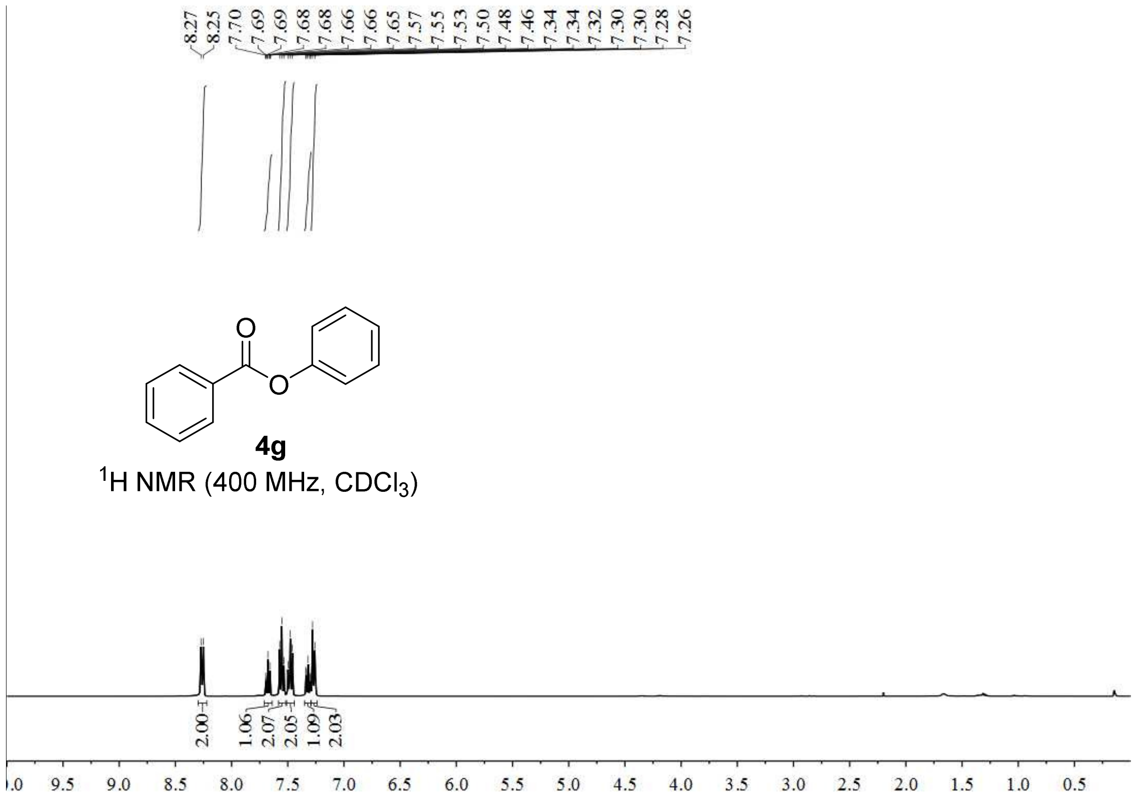

Supplementary Figure 59. ^{1}H NMR (400 MHz, $CDCl_3$) of compound **4g**.

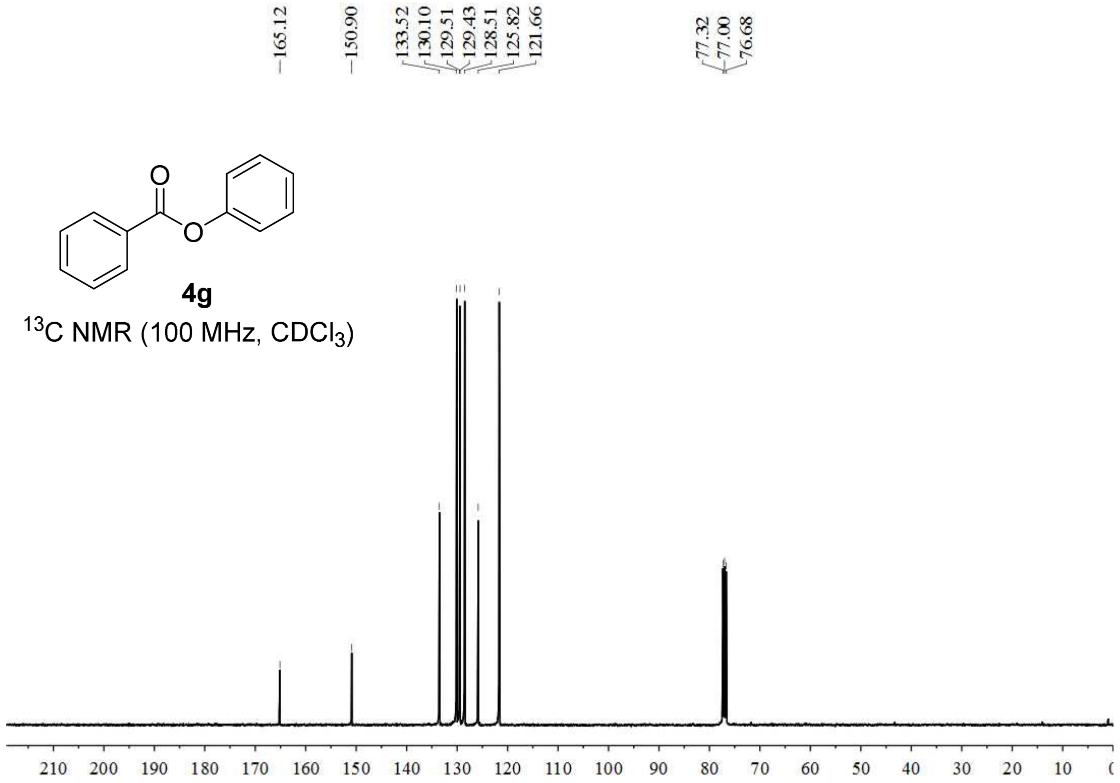

Supplementary Figure 60. ^{13}C NMR (100 MHz, $CDCl_3$) of compound **4g**.

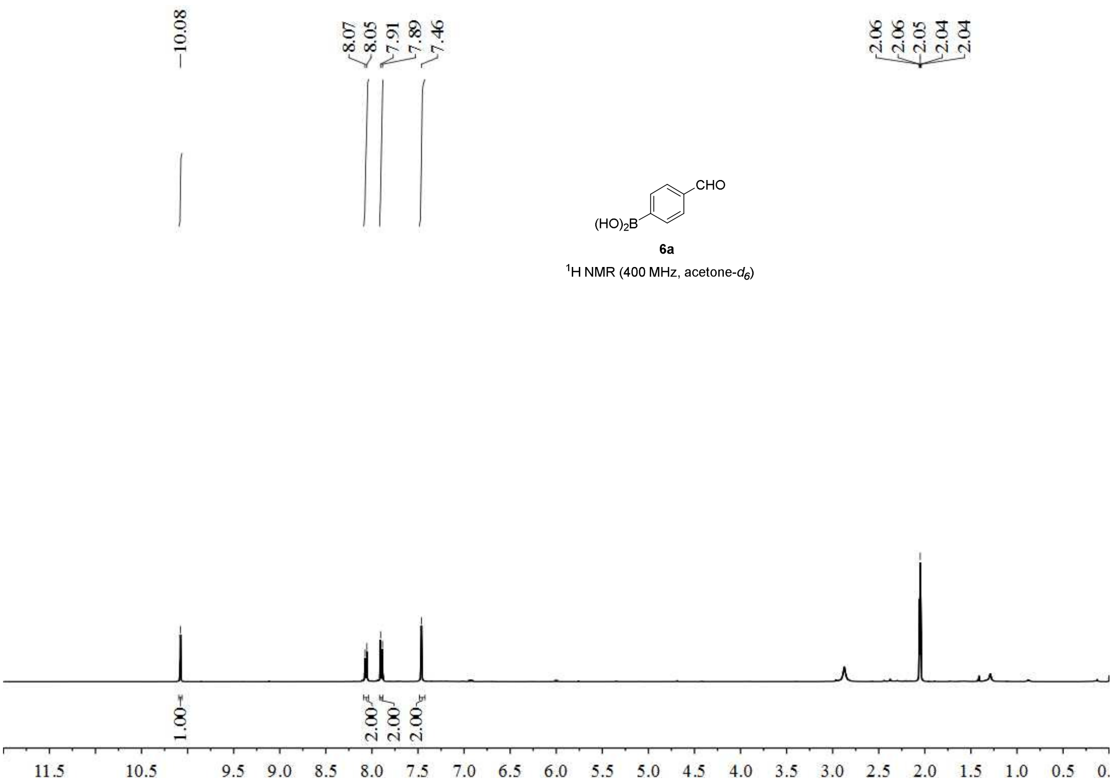

Supplementary Figure 61. ^{1}H NMR (400 MHz, acetone-d_6) of compound **6a**.

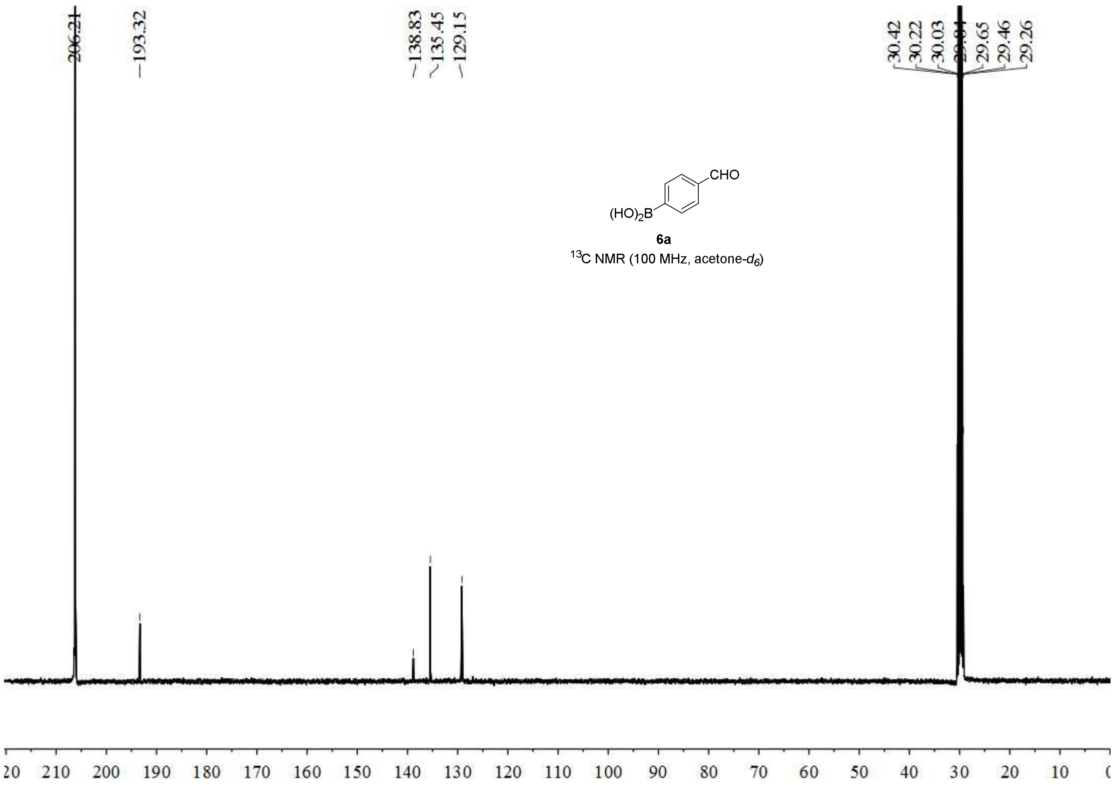

Supplementary Figure 62. ^{13}C NMR (100 MHz, acetone-d_6) of compound **6a**

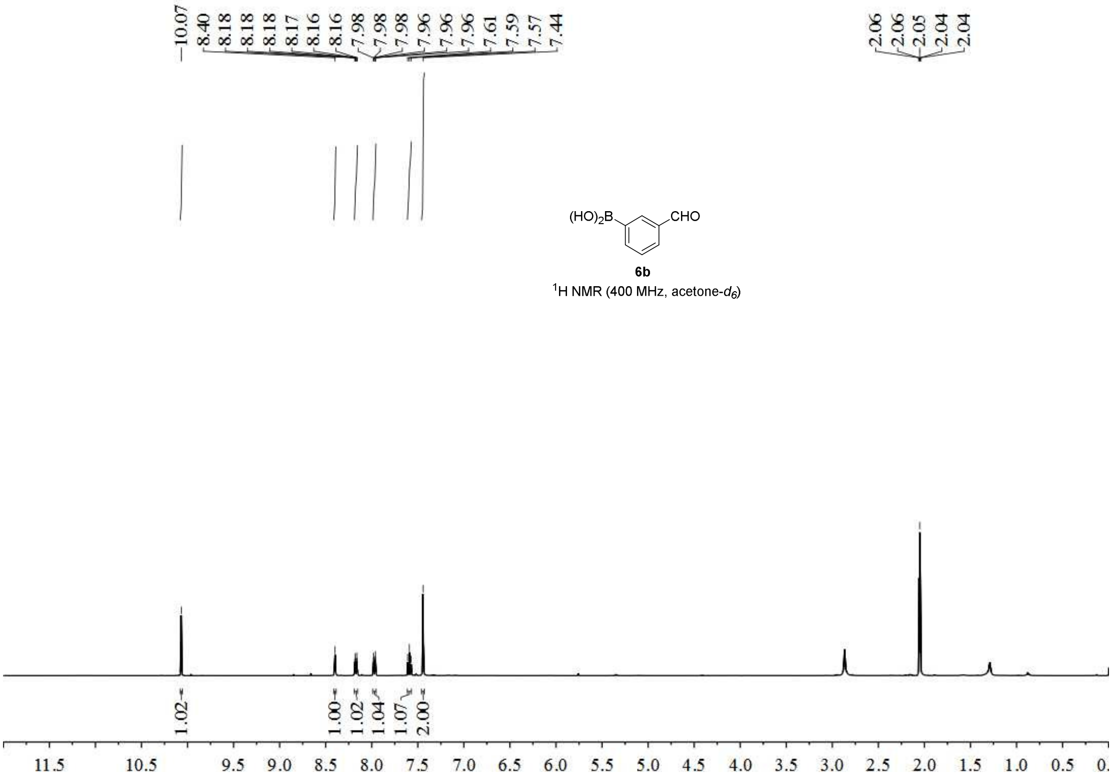

Supplementary Figure 63. ^{1}H NMR (400 MHz, acetone-d_6) of compound **6b**.

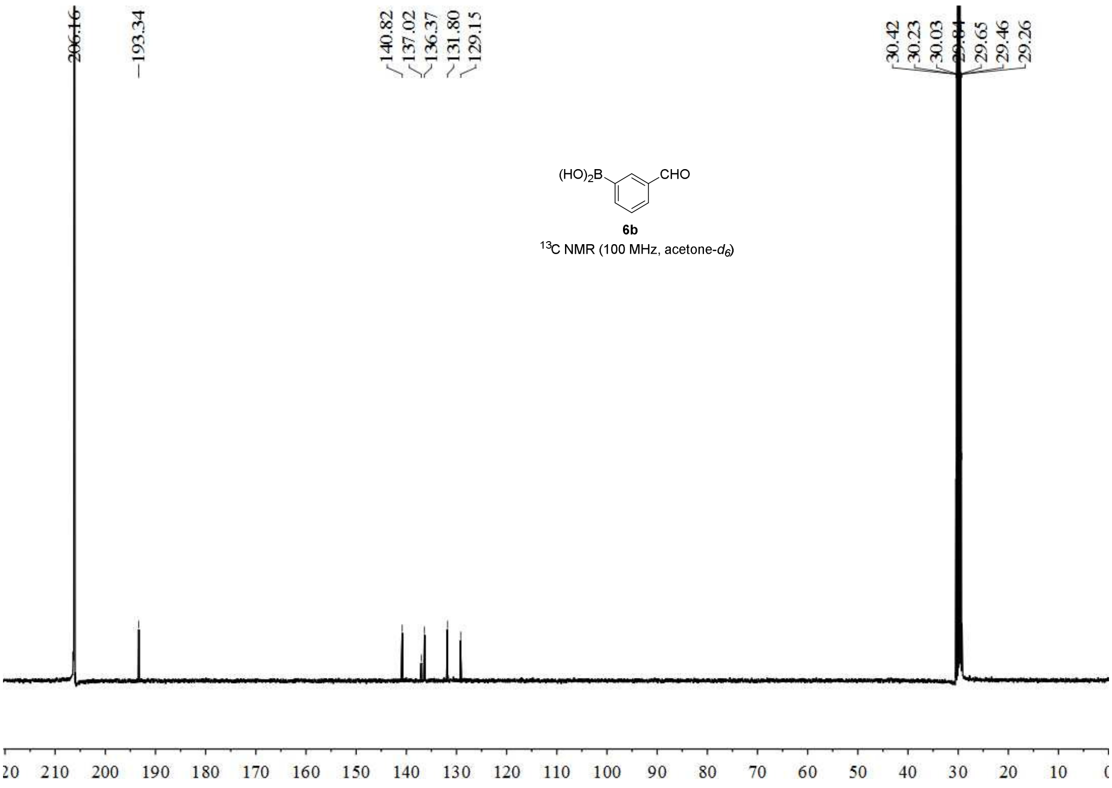

Supplementary Figure 64. ^{13}C NMR (100 MHz, acetone-d_6) of compound **6b**.

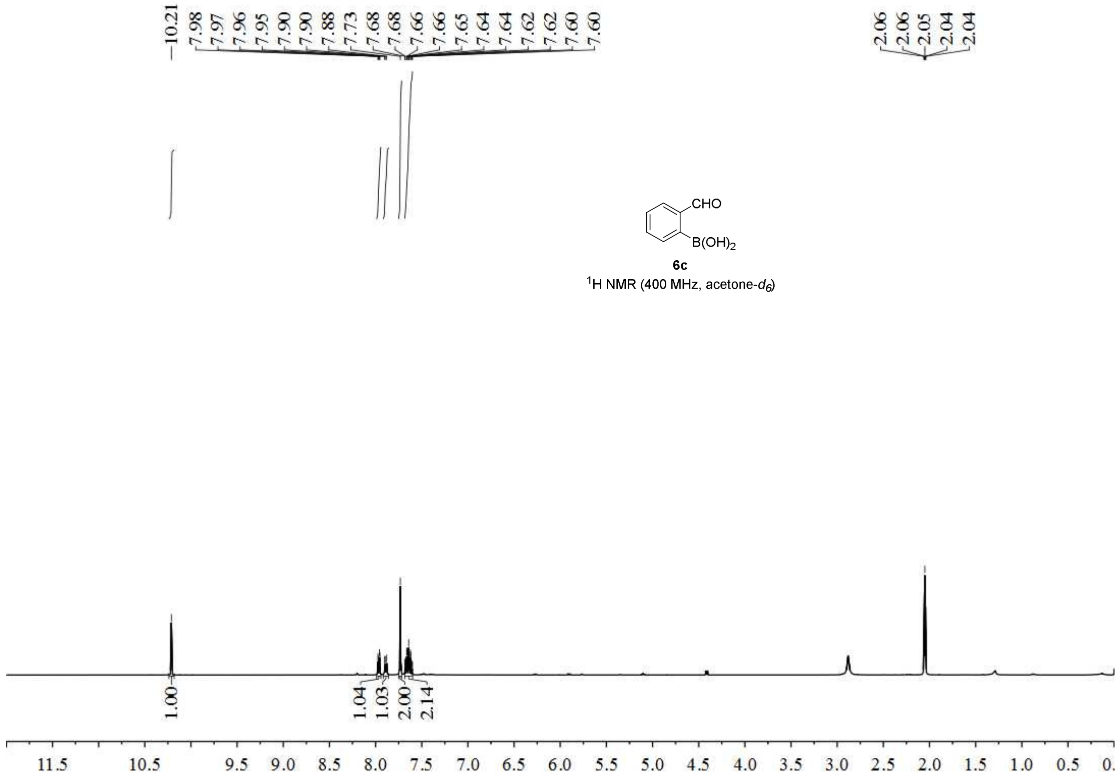

Supplementary Figure 65. ^{1}H NMR (400 MHz, acetone-d_6) of compound **6c**.

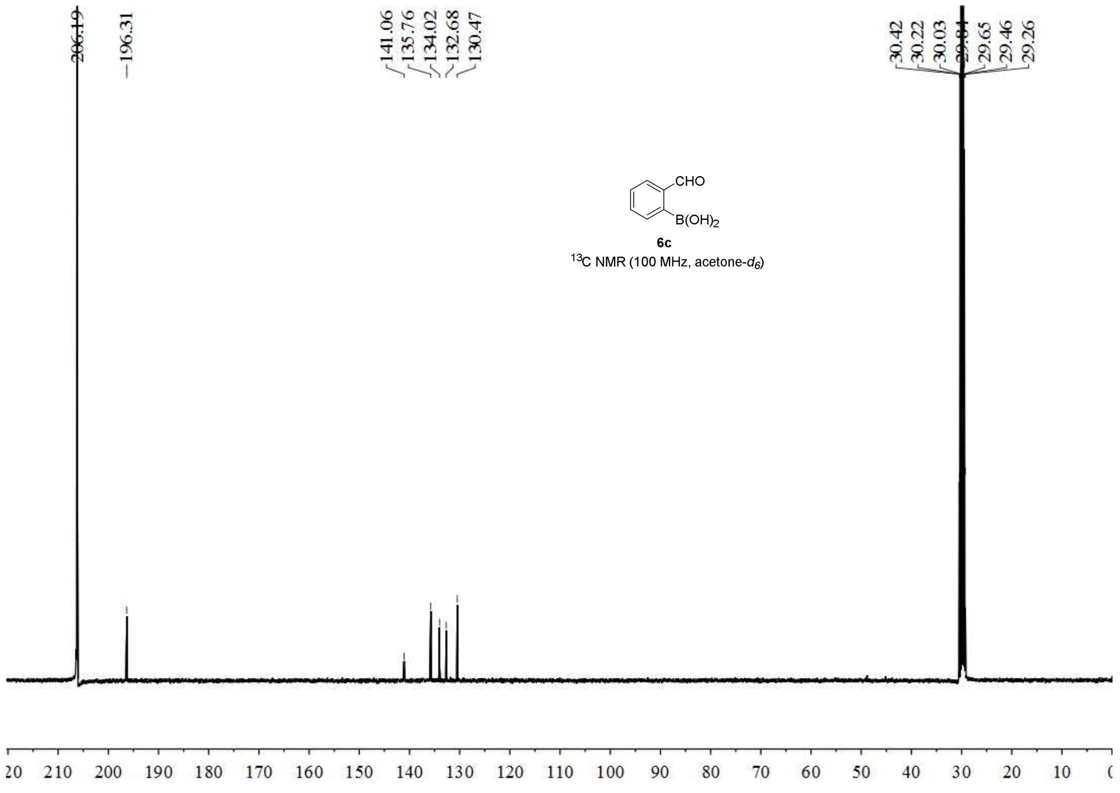

Supplementary Figure 66. ^{13}C NMR (100 MHz, acetone-d_6) of compound **6c**.

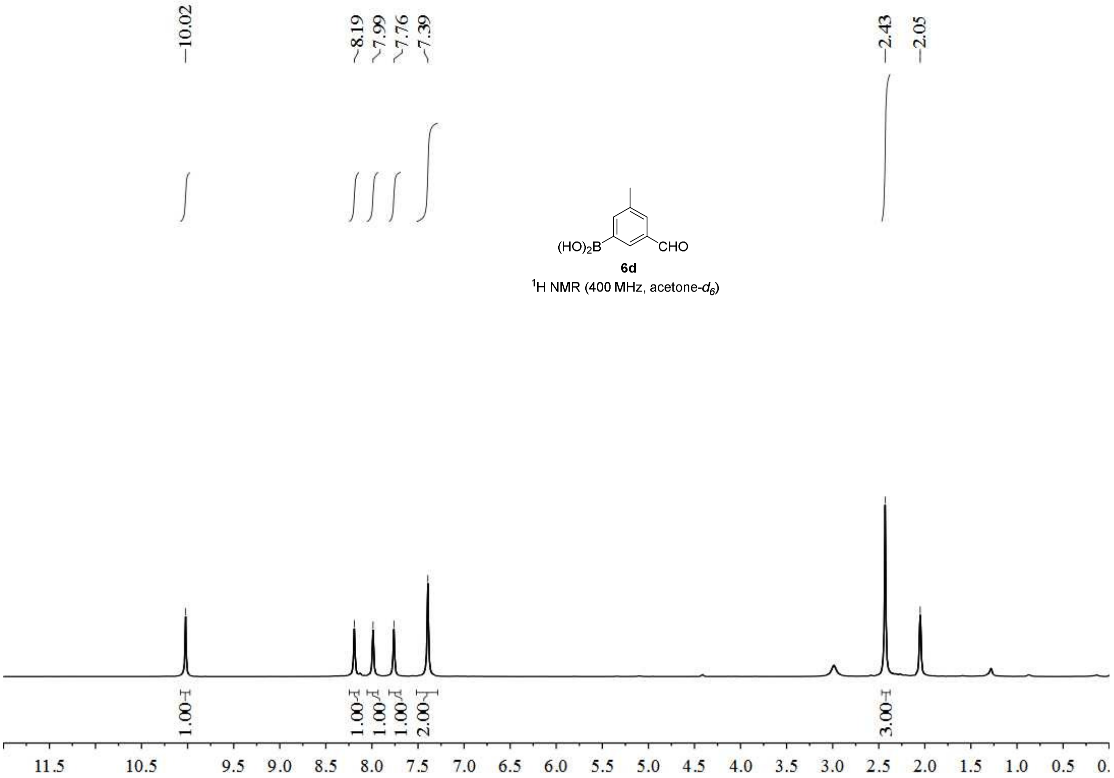

Supplementary Figure 67. ^{1}H NMR (400 MHz, acetone-d_6) of compound **6d**.

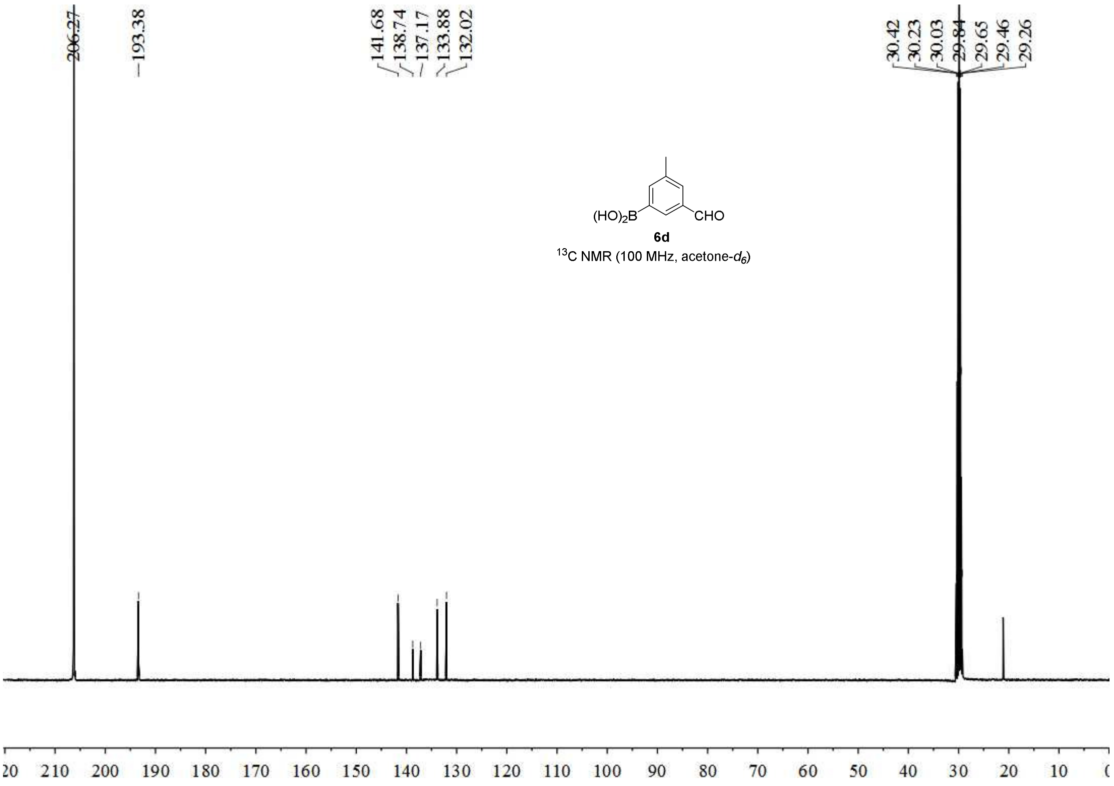

Supplementary Figure 68. ^{13}C NMR (100 MHz, acetone-d_6) of compound **6d**.

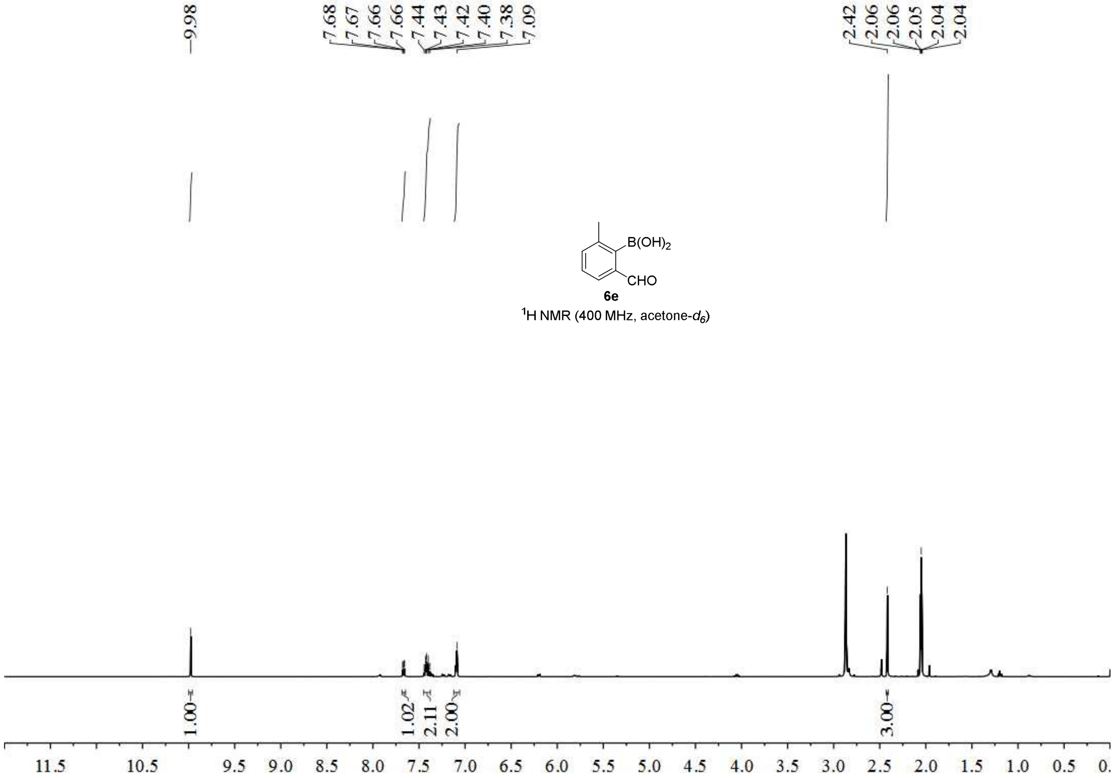

Supplementary Figure 69. ^{1}H NMR (400 MHz, acetone-d_6) of compound **6e**.

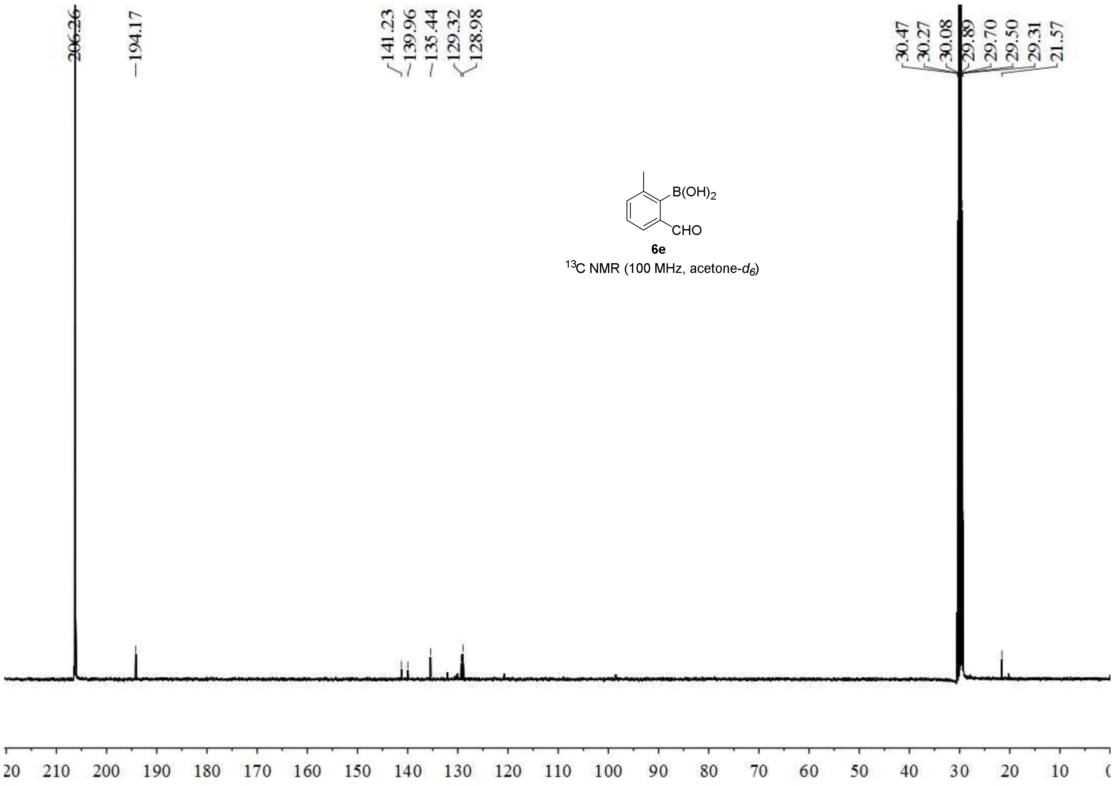

Supplementary Figure 70. ^{13}C NMR (100 MHz, acetone-d_6) of compound **6e**.

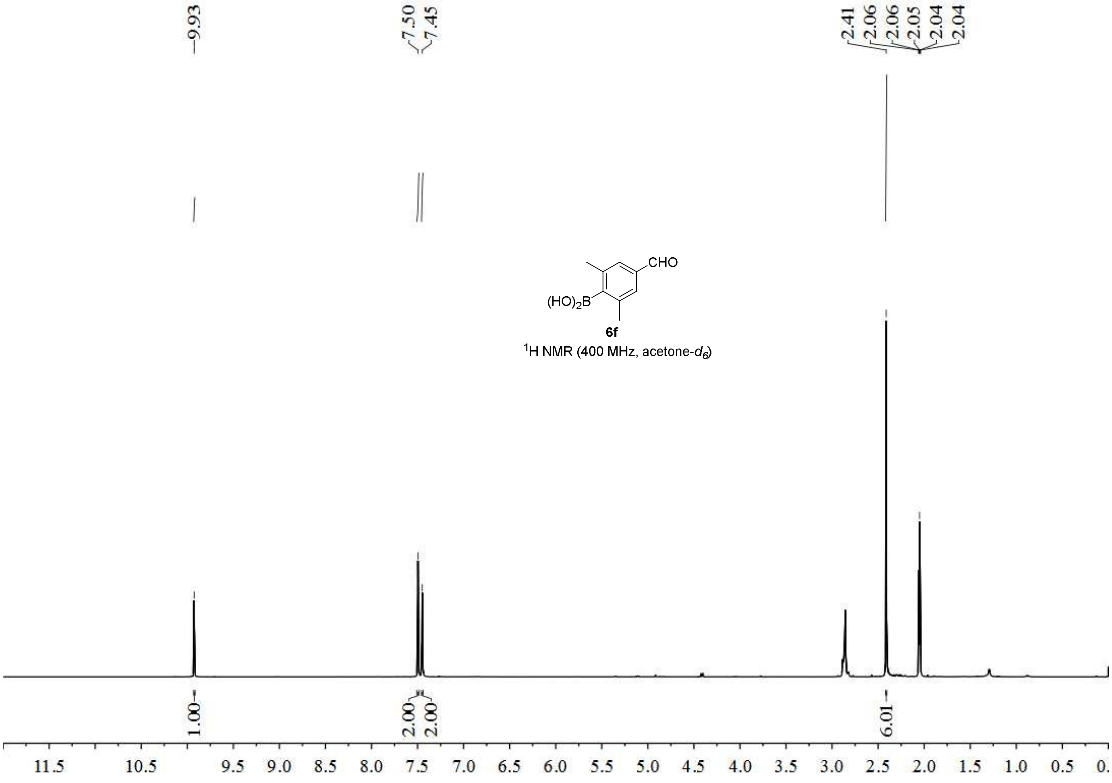

Supplementary Figure 71. ^{1}H NMR (400 MHz, acetone-d_6) of compound **6f**.

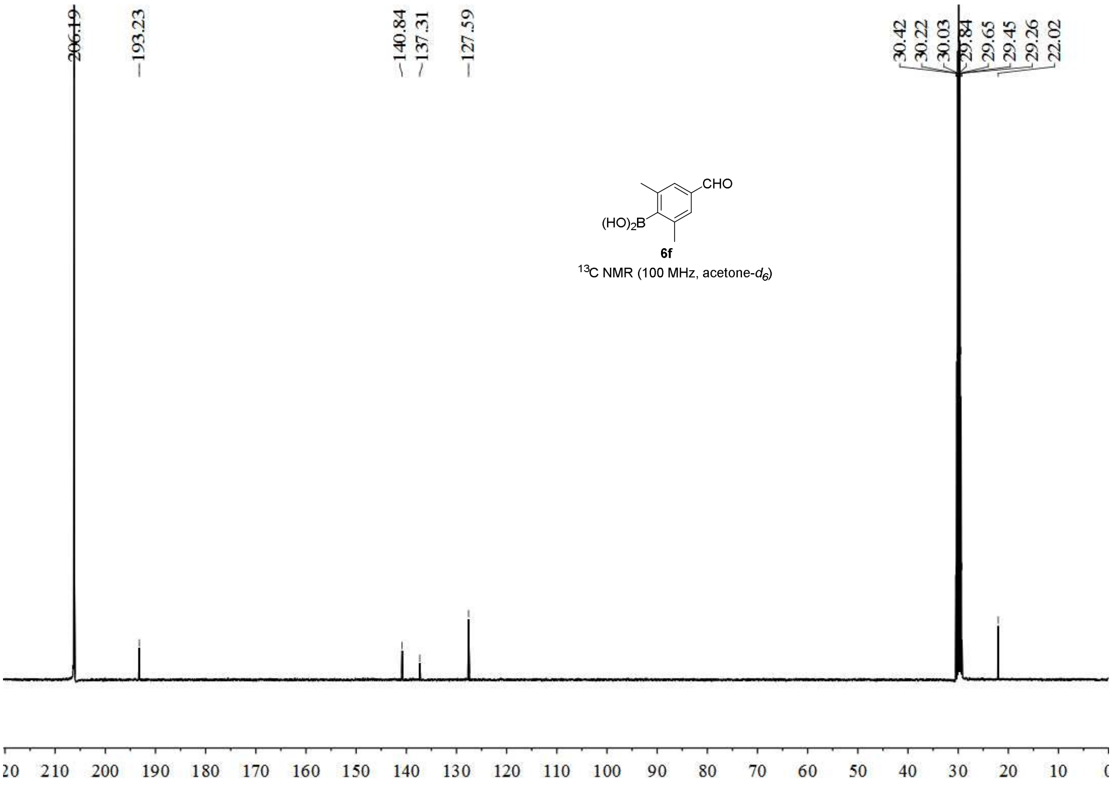

Supplementary Figure 72. ^{13}C NMR (100 MHz, acetone-d_6) of compound **6f**.

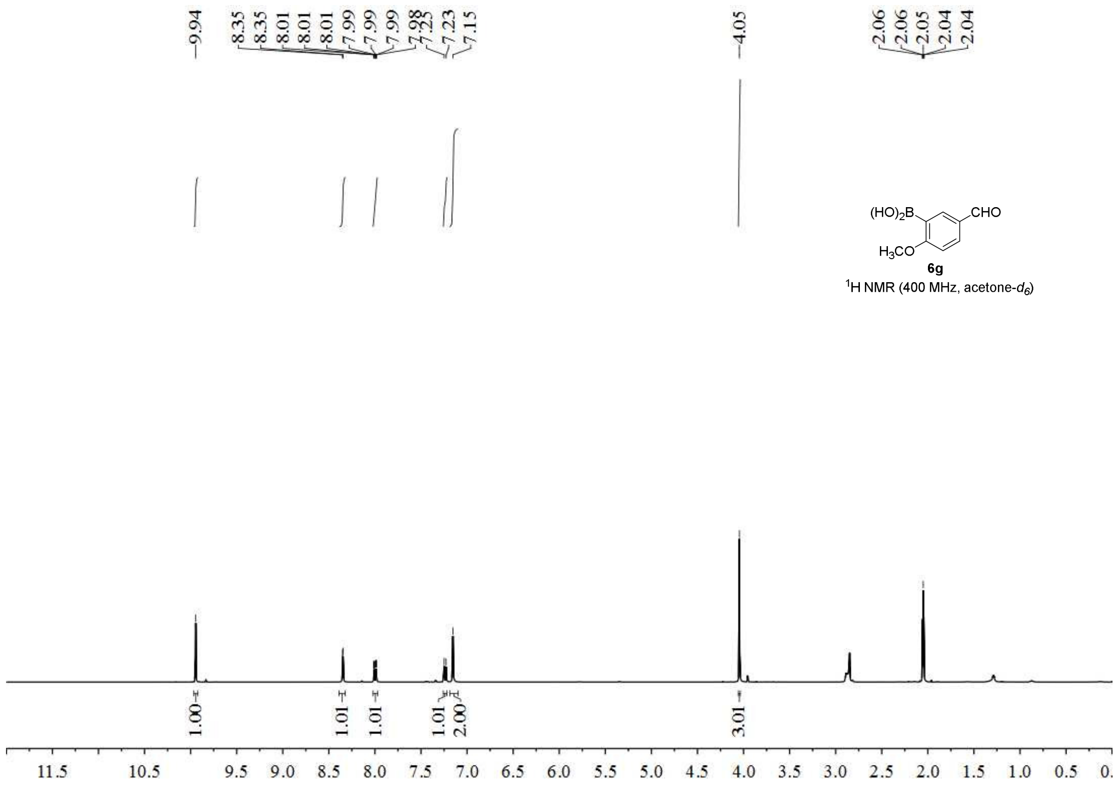

Supplementary Figure 73. ^{1}H NMR (400 MHz, acetone-d_6) of compound **6g**.

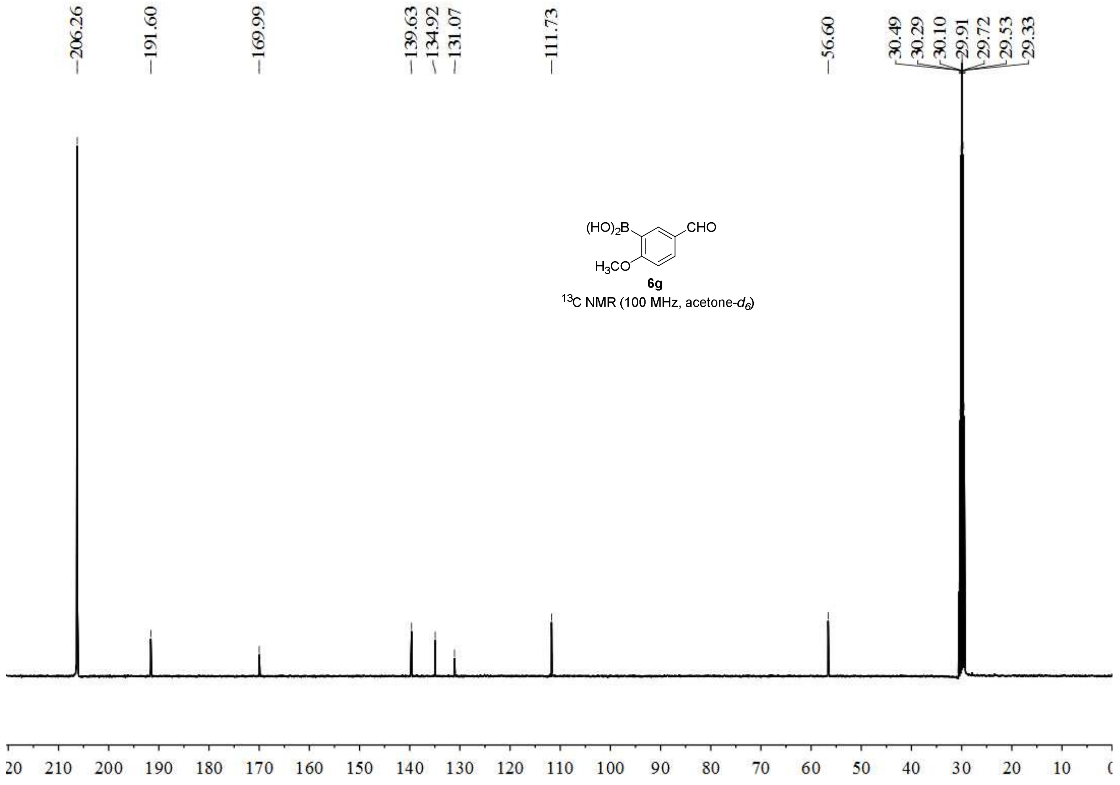

Supplementary Figure 74. ^{13}C NMR (100 MHz, acetone-d_6) of compound **6g**.

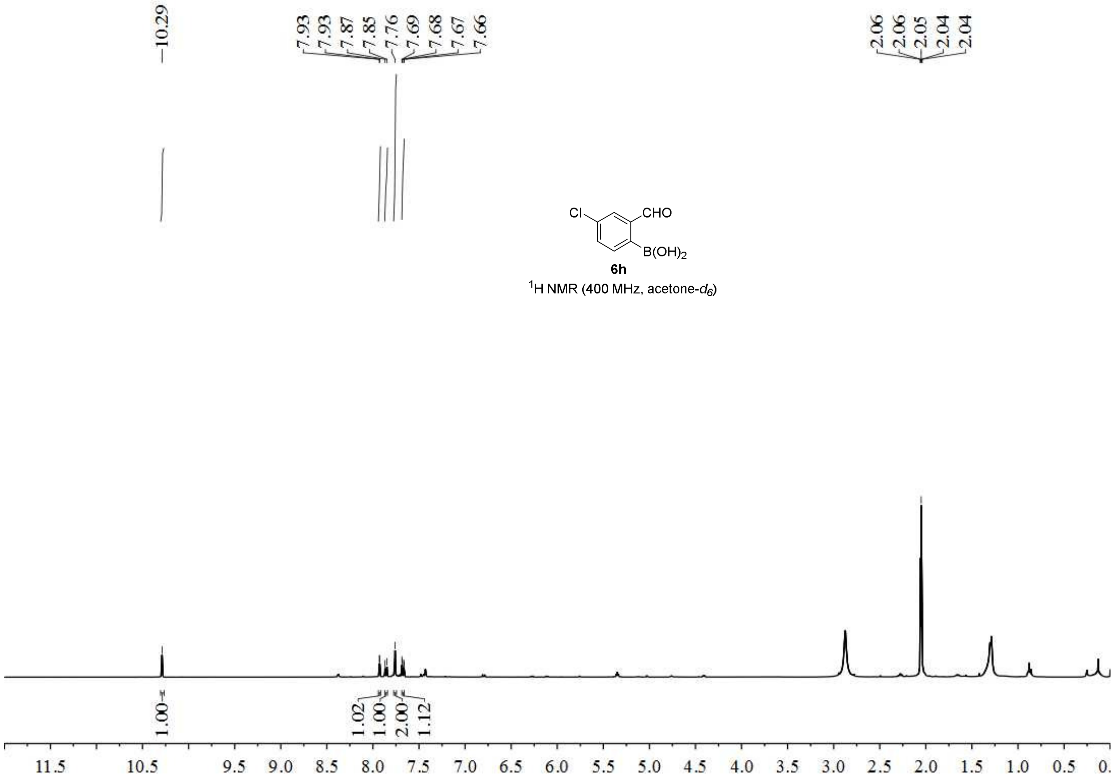

Supplementary Figure 75. ^{1}H NMR (400 MHz, acetone-d_6) of compound **6h**.

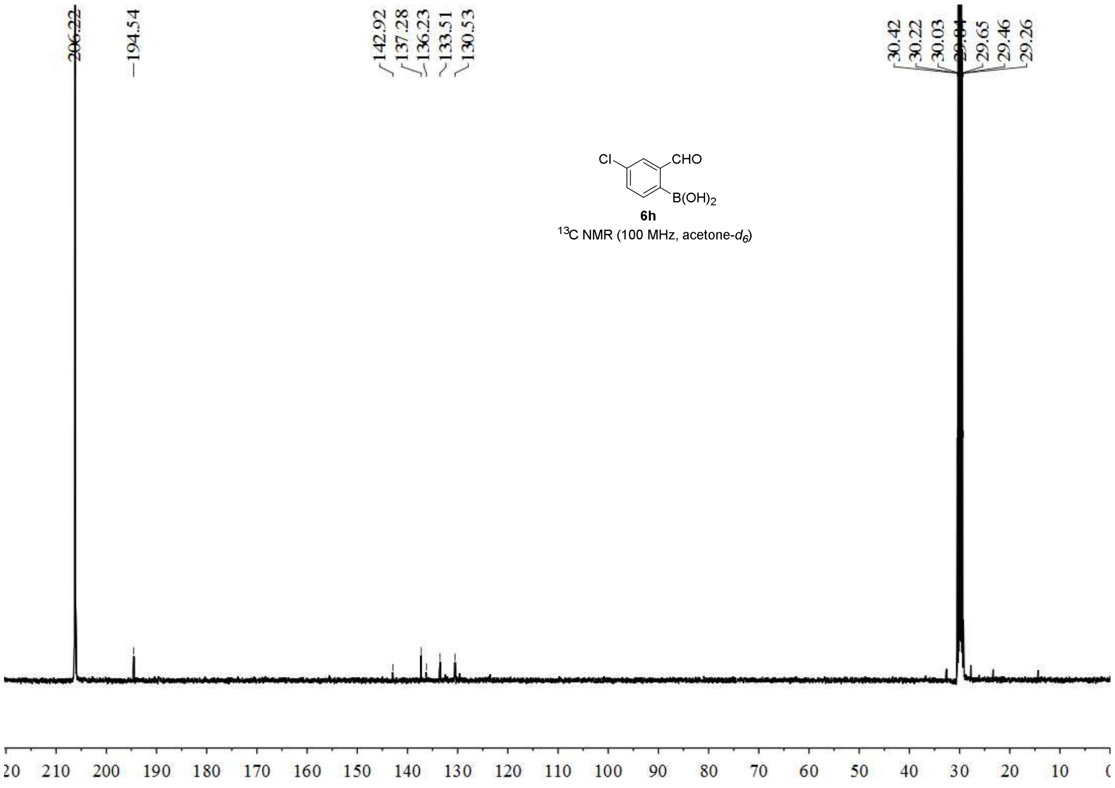

Supplementary Figure 76. ^{13}C NMR (100 MHz, acetone-d_6) of compound **6h**.

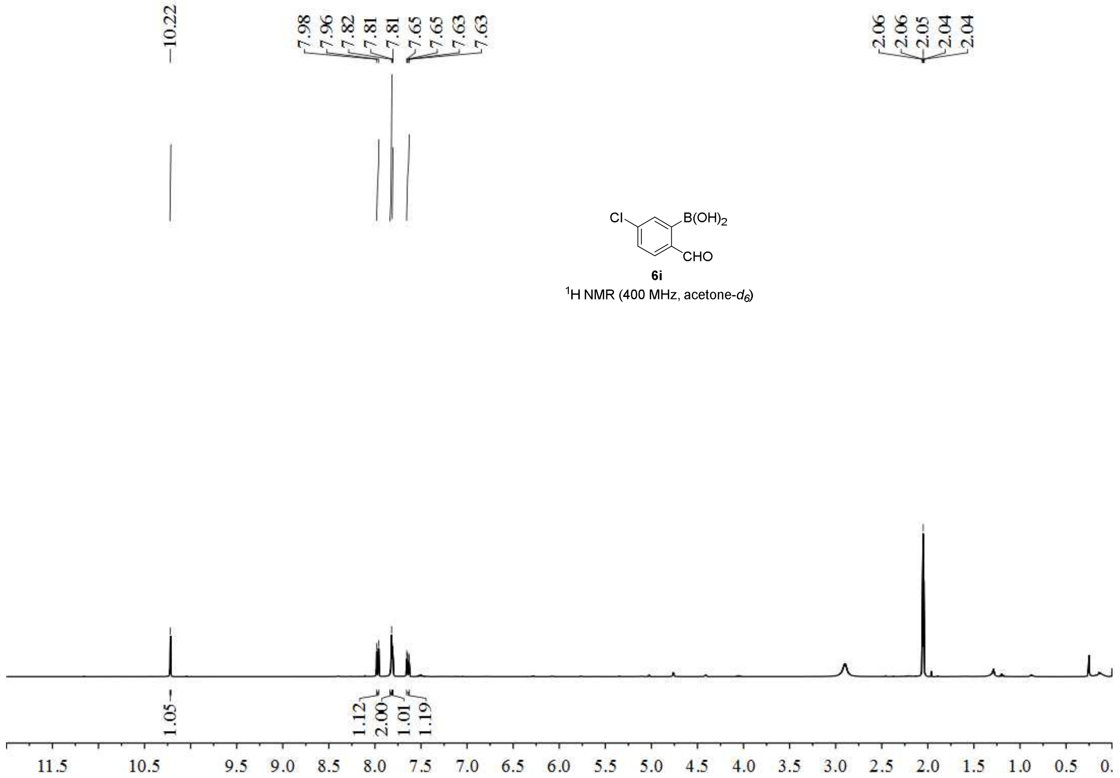

Supplementary Figure 77. ^{1}H NMR (400 MHz, acetone-d_6) of compound **6i**.

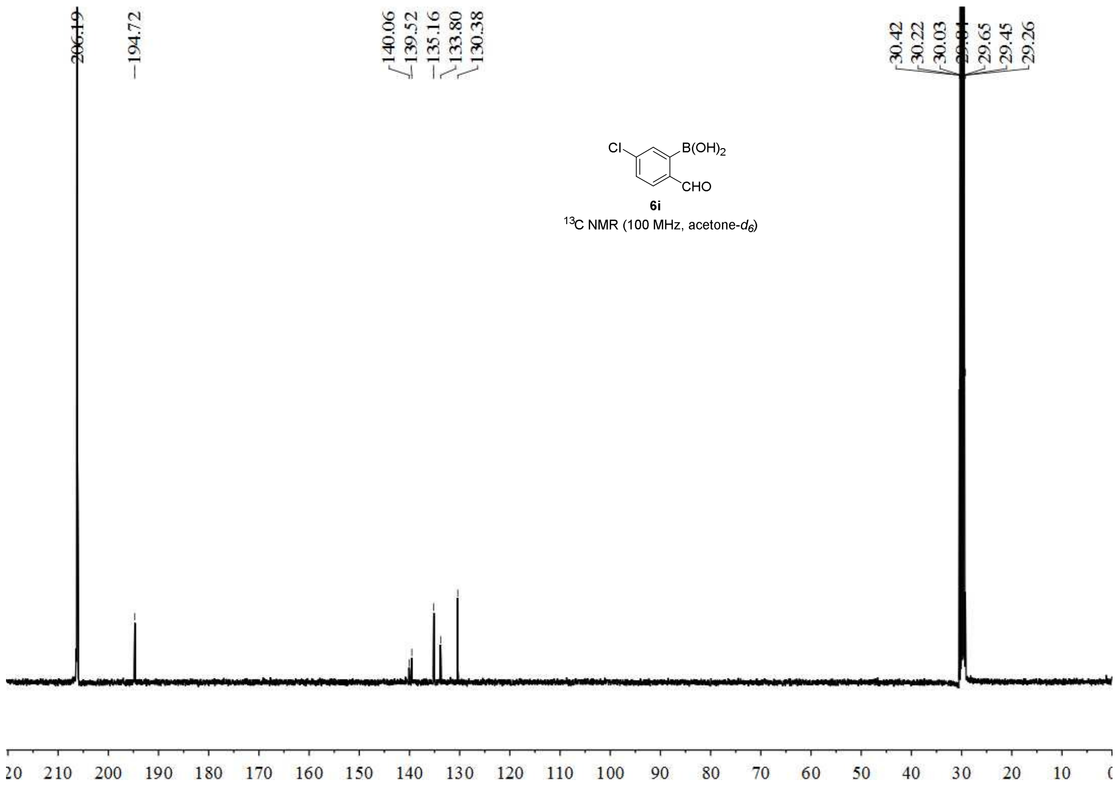

Supplementary Figure 78. ^{13}C NMR (100 MHz, acetone-d_6) of compound **6i**.

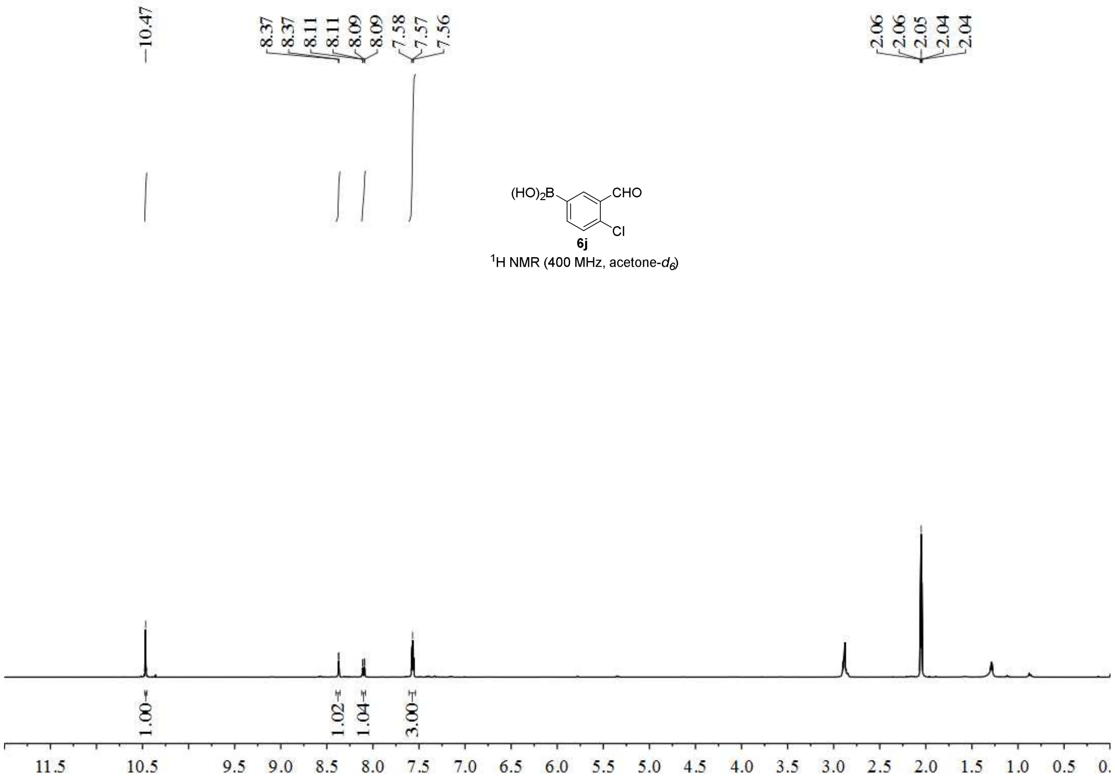

Supplementary Figure 79. ^{1}H NMR (400 MHz, acetone-d_6) of compound **6j**.

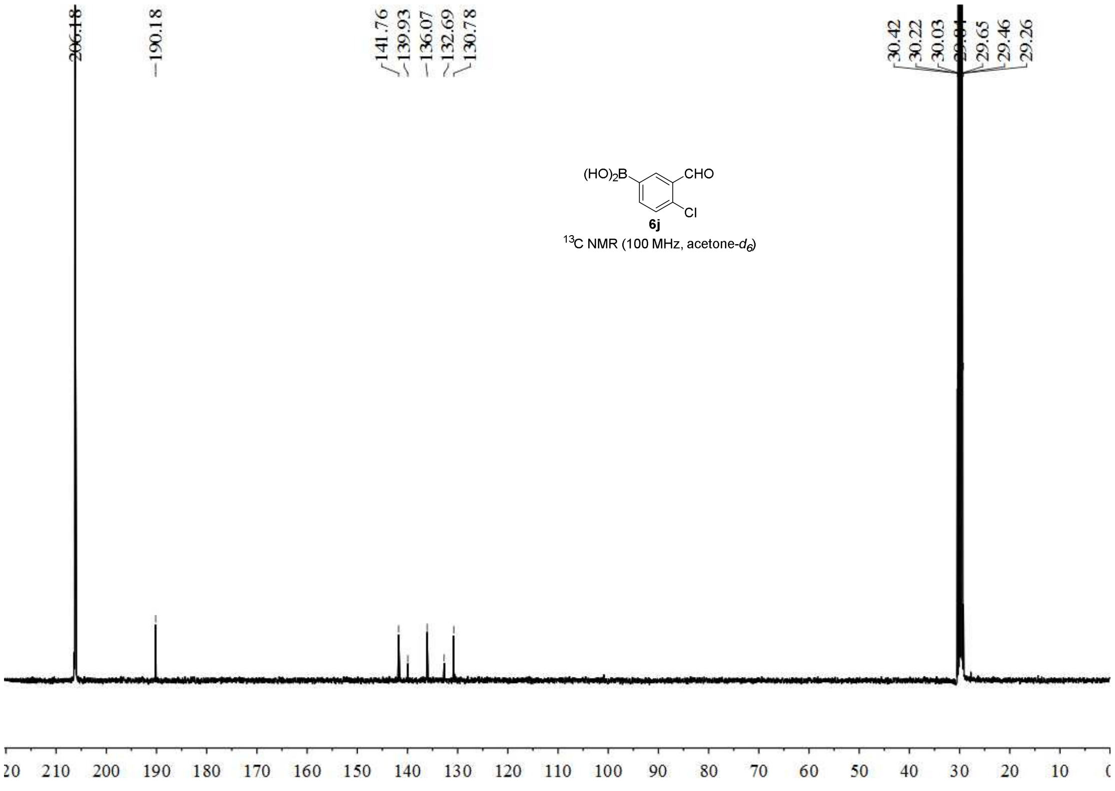

Supplementary Figure 80. ^{13}C NMR (100 MHz, acetone-d_6) of compound **6j**.

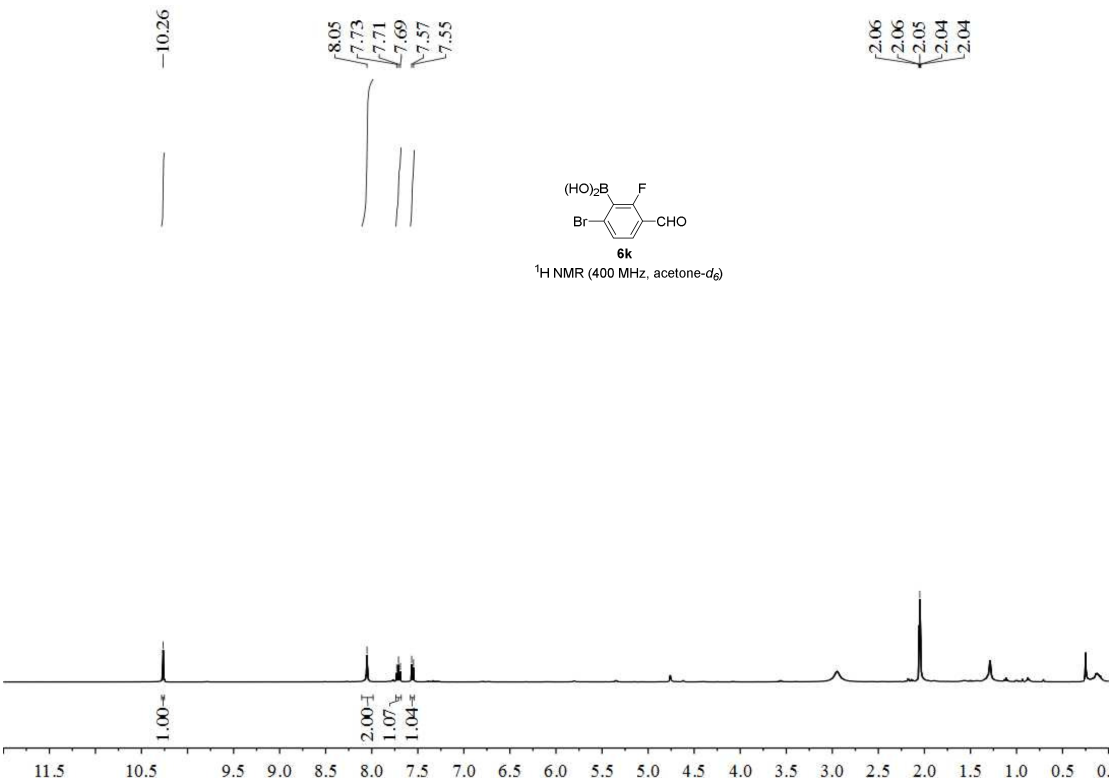

Supplementary Figure 81. ^{1}H NMR (400 MHz, acetone-d_6) of compound **6k**.

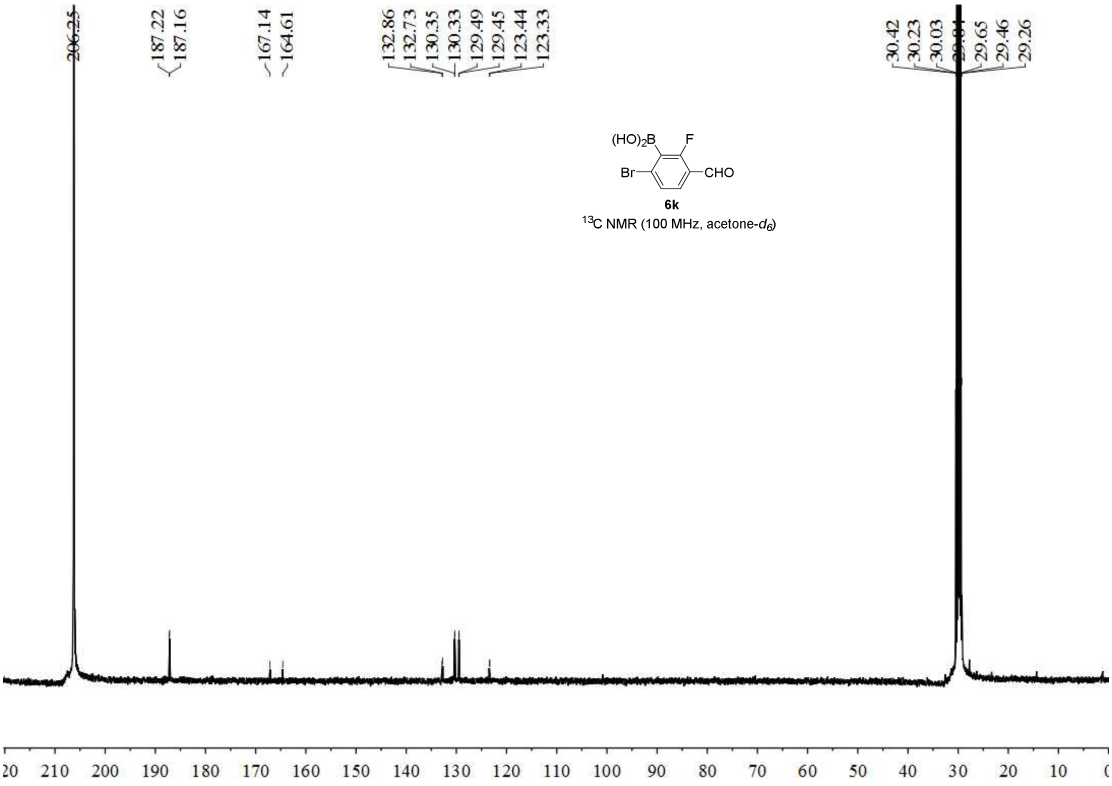

Supplementary Figure 82. ^{13}C NMR (100 MHz, acetone-d_6) of compound **6k**.

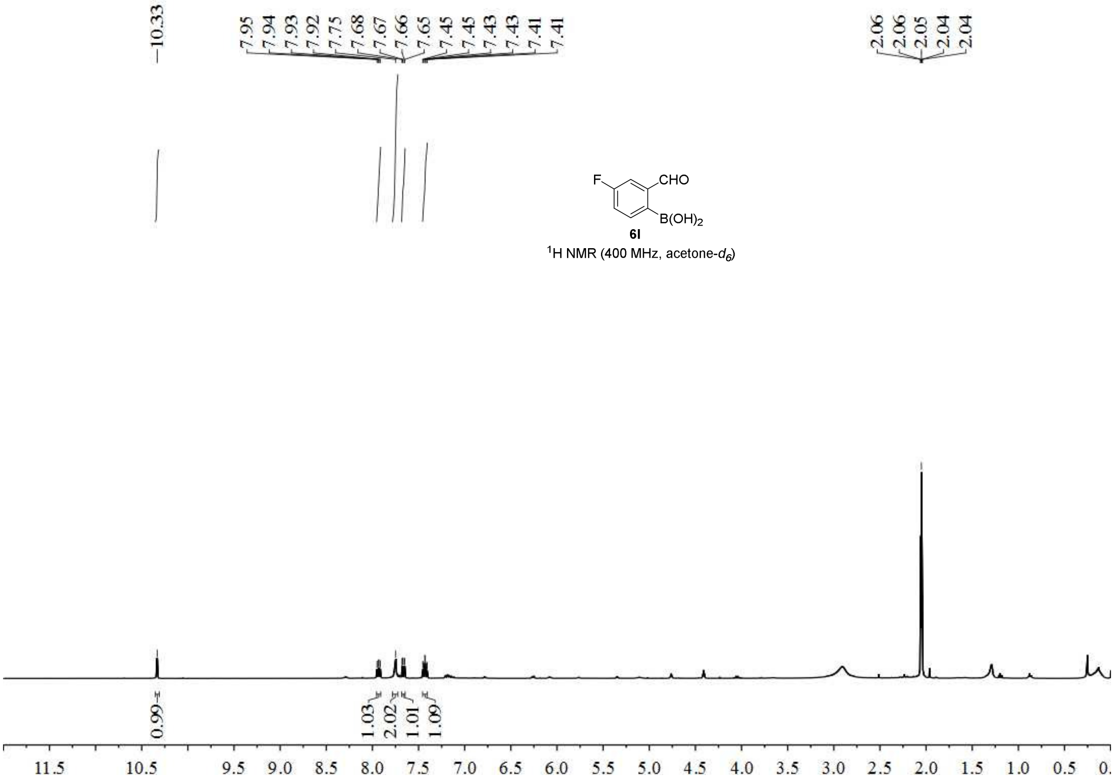

Supplementary Figure 83. ^{1}H NMR (400 MHz, acetone-d_6) of compound **6l**.

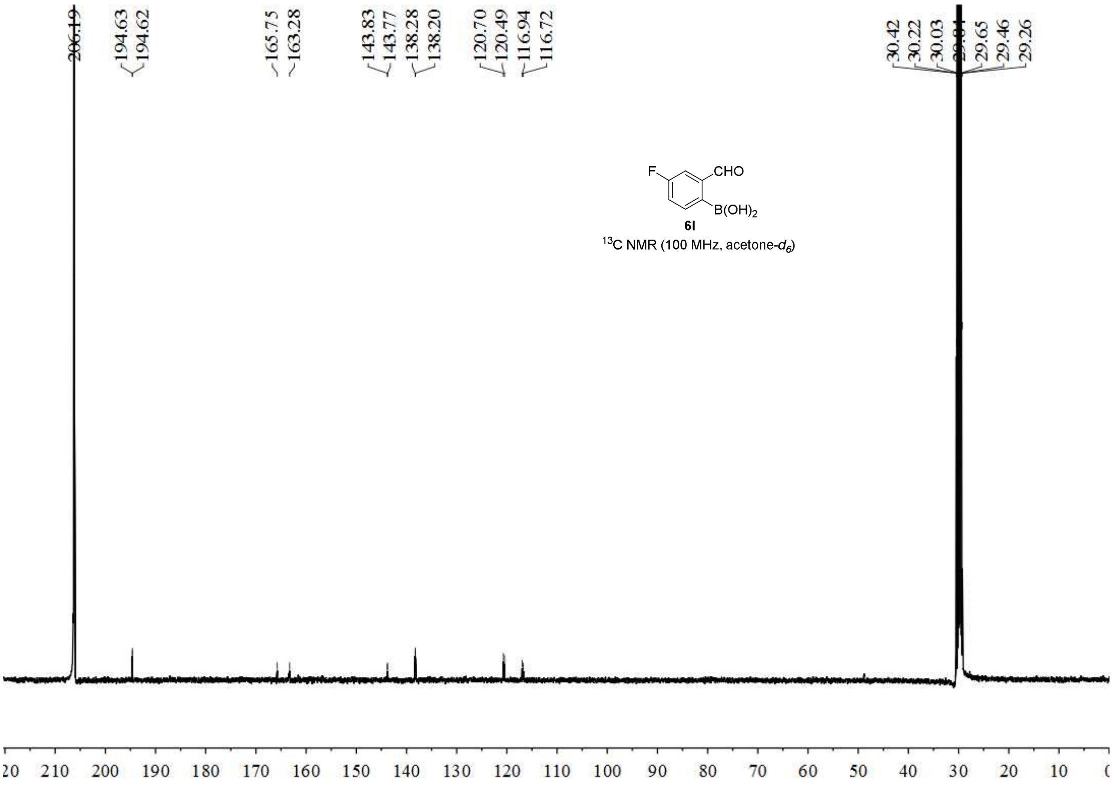

Supplementary Figure 84. ^{13}C NMR (100 MHz, acetone-d_6) of compound **6l**.

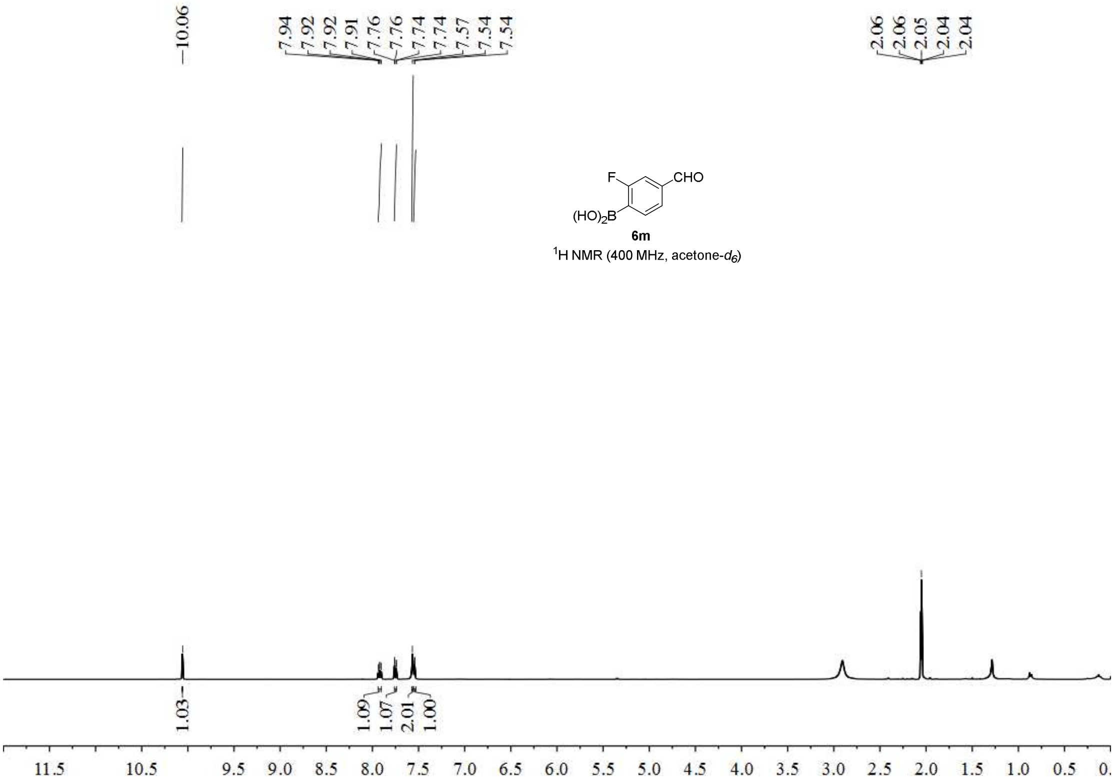

Supplementary Figure 85. ^{1}H NMR (400 MHz, acetone-d_6) of compound **6m**.

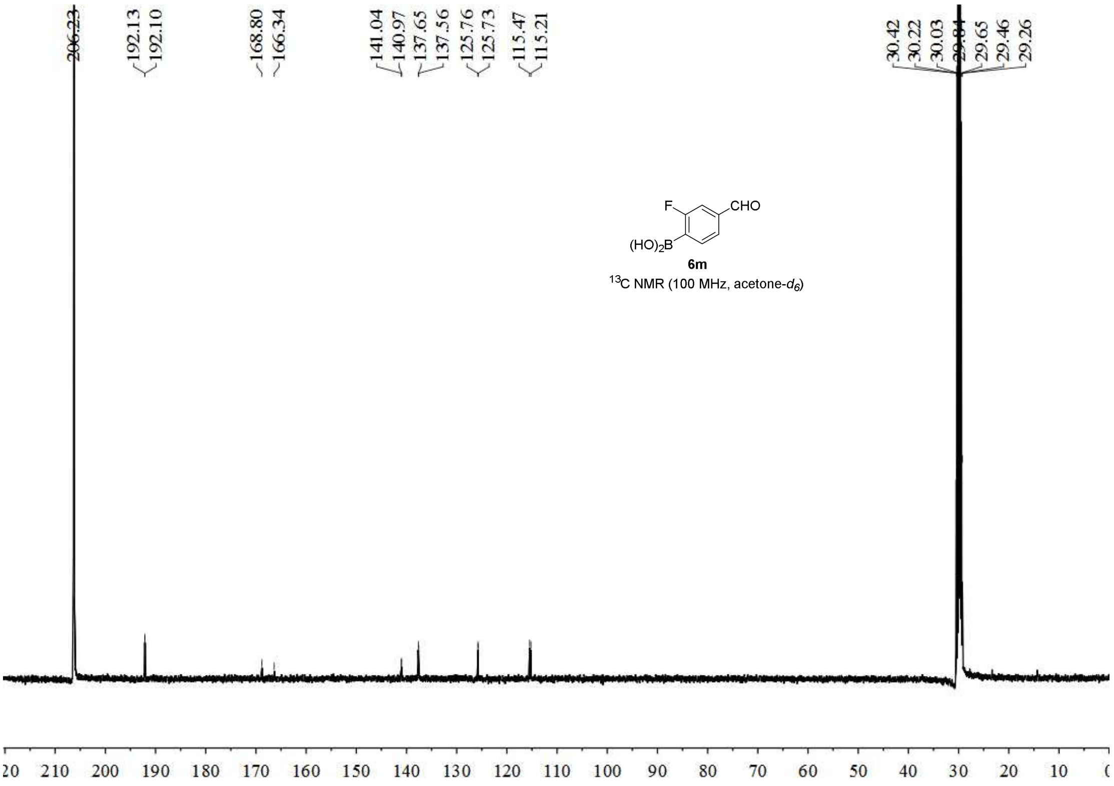

Supplementary Figure 86. ^{13}C NMR (100 MHz, acetone-d_6) of compound **6m**.

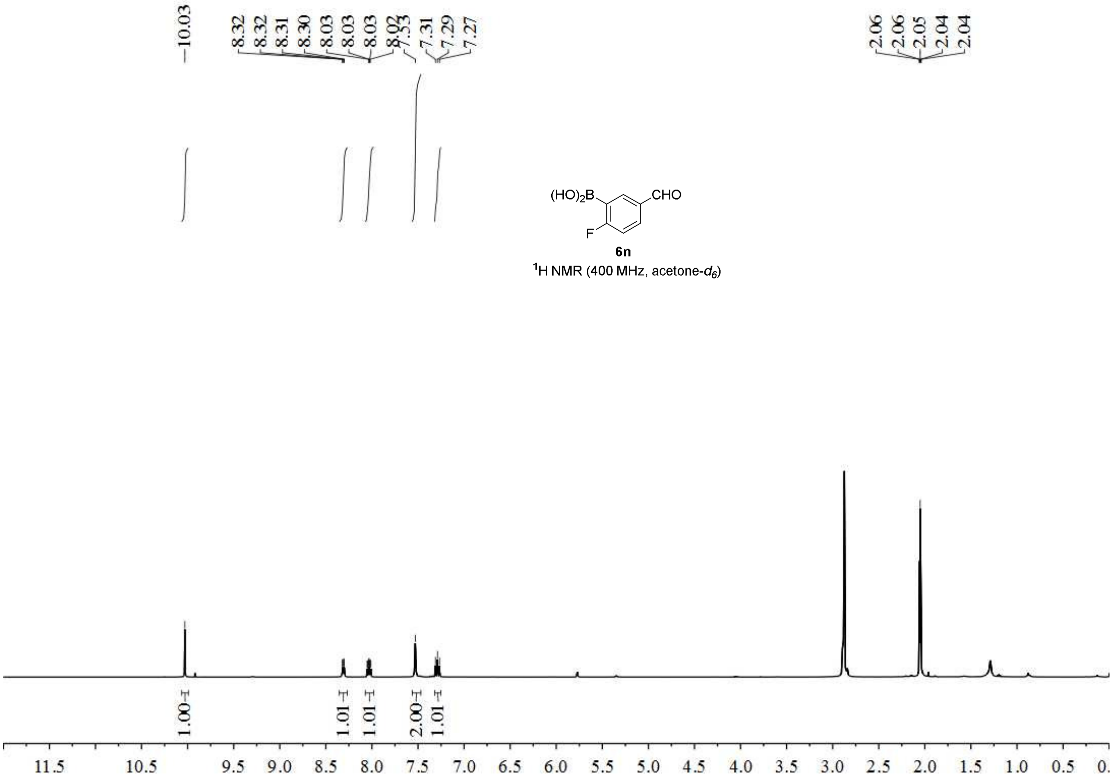

Supplementary Figure 87. ^{1}H NMR (400 MHz, acetone-d_6) of compound **6n**.

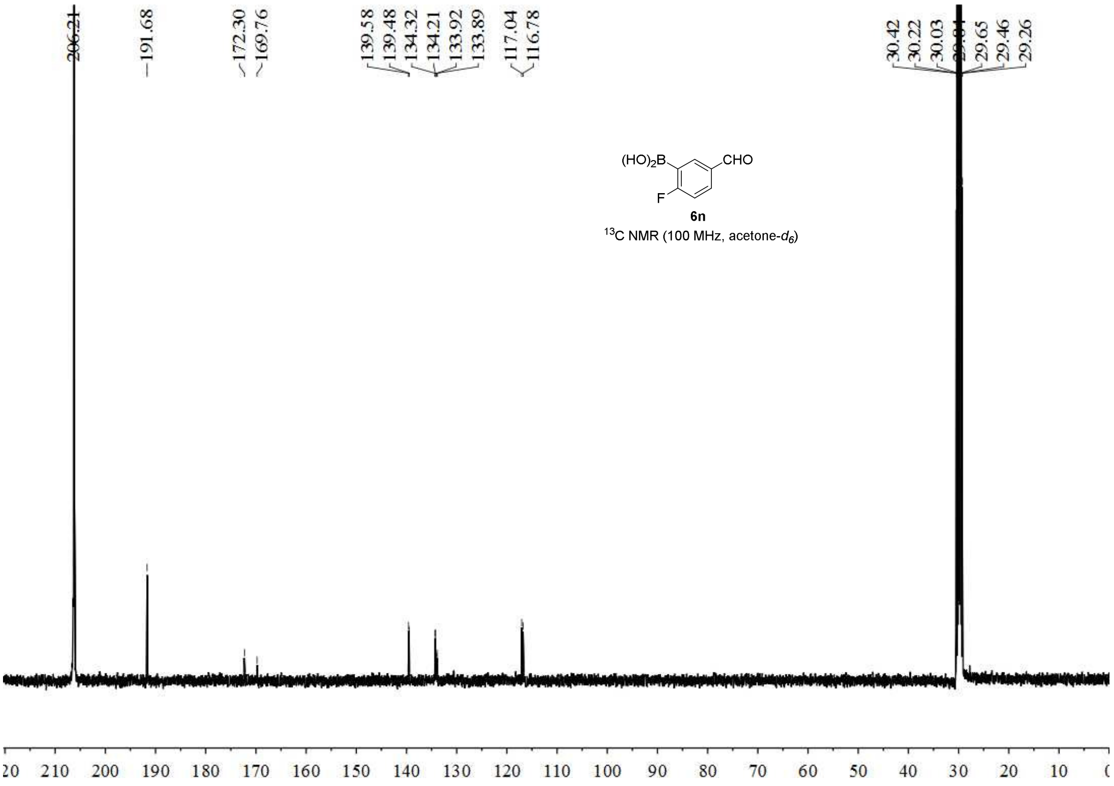

Supplementary Figure 88. ^{13}C NMR (100 MHz, acetone-d_6) of compound **6n**.

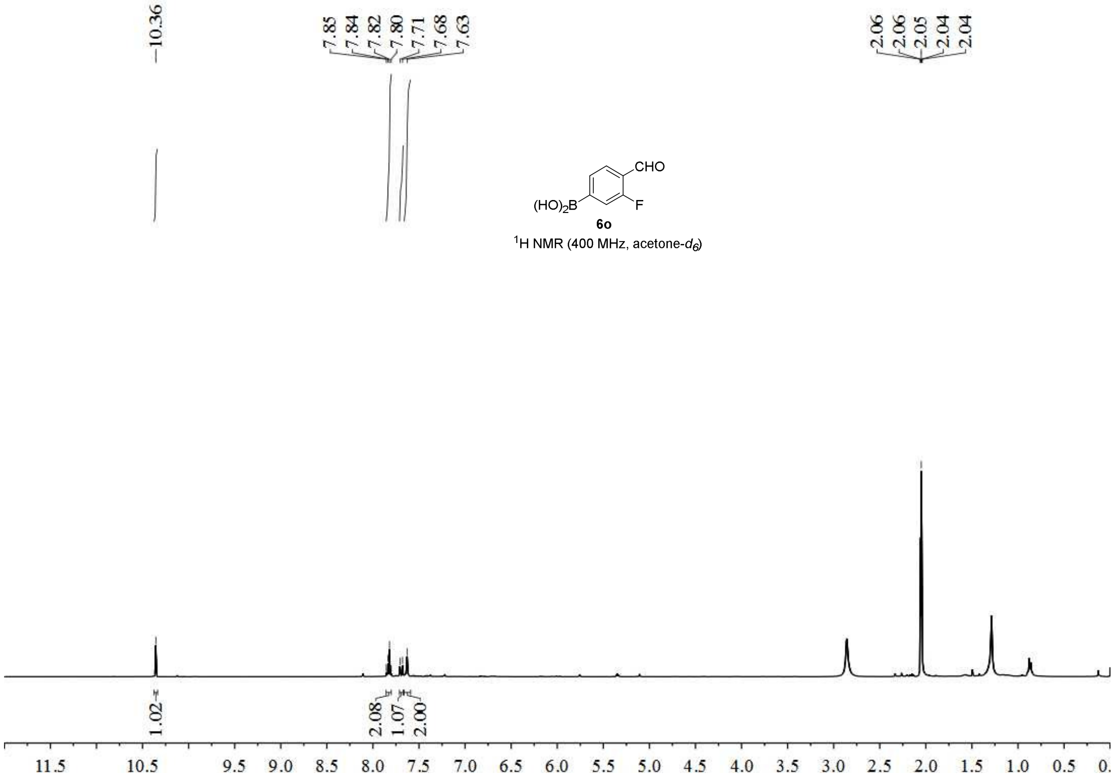

Supplementary Figure 89. ^{1}H NMR (400 MHz, acetone-d_6) of compound **6o**.

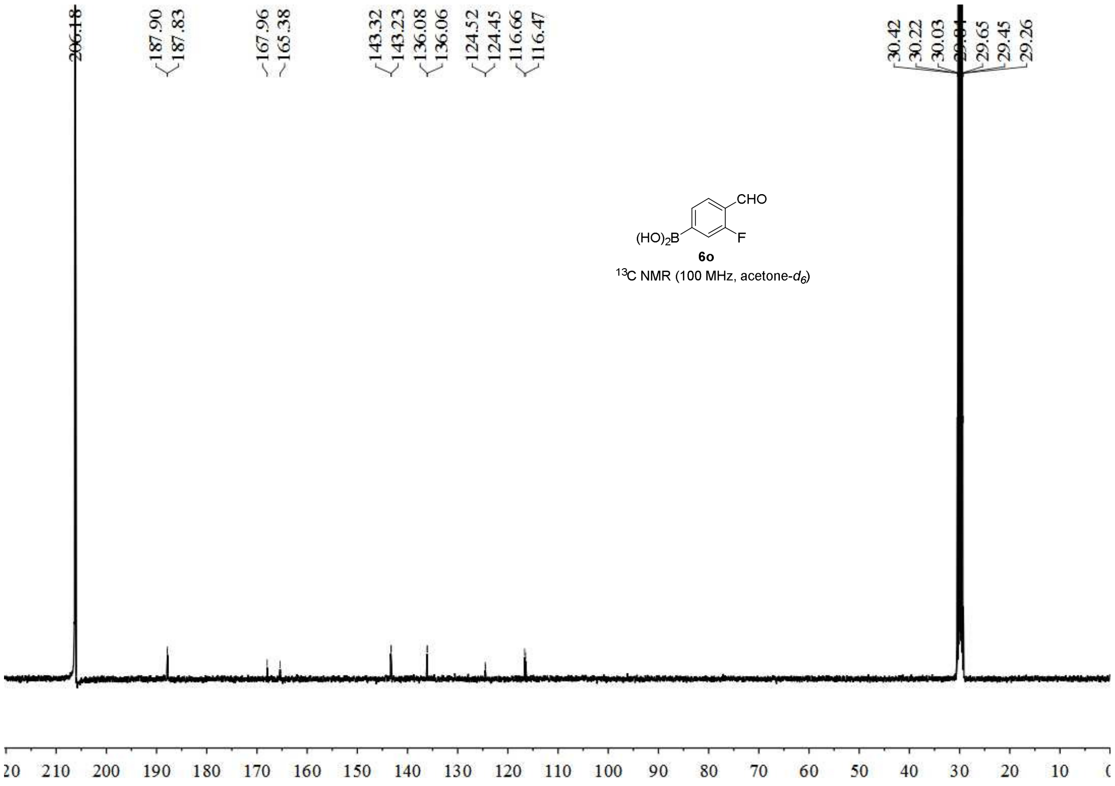

Supplementary Figure 90. ^{13}C NMR (100 MHz, acetone-d_6) of compound **6o**.

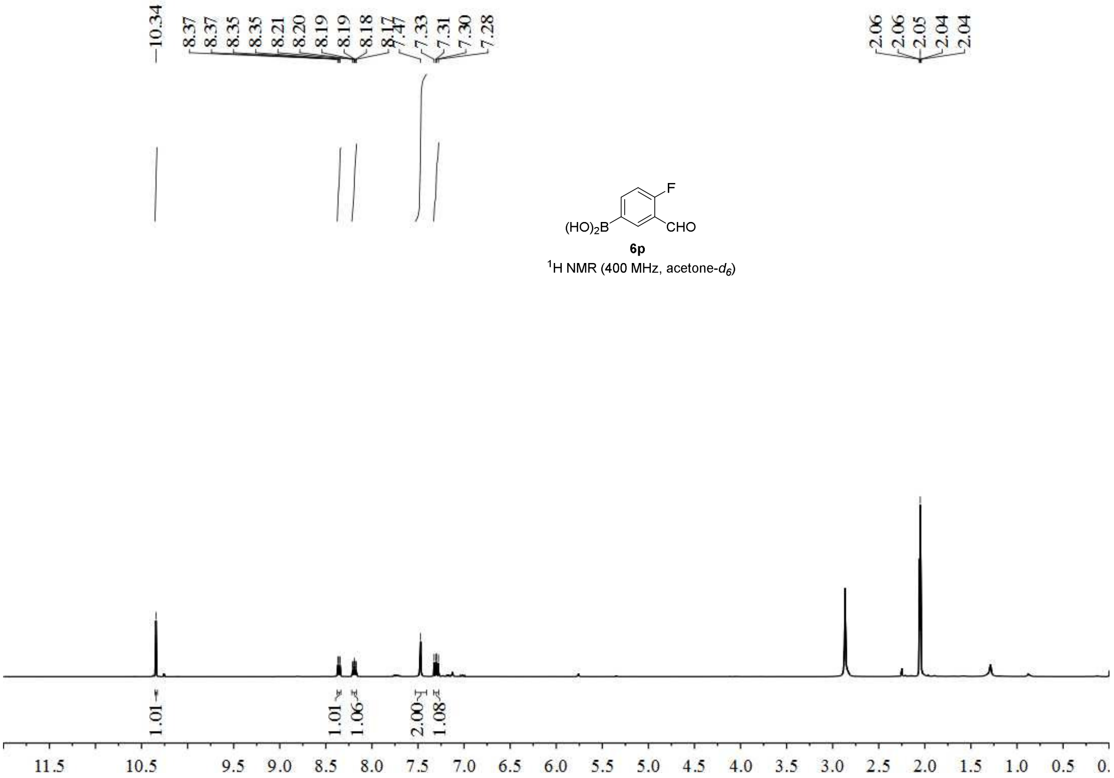

Supplementary Figure 91. ^{1}H NMR (400 MHz, acetone-d_6) of compound **6p**.

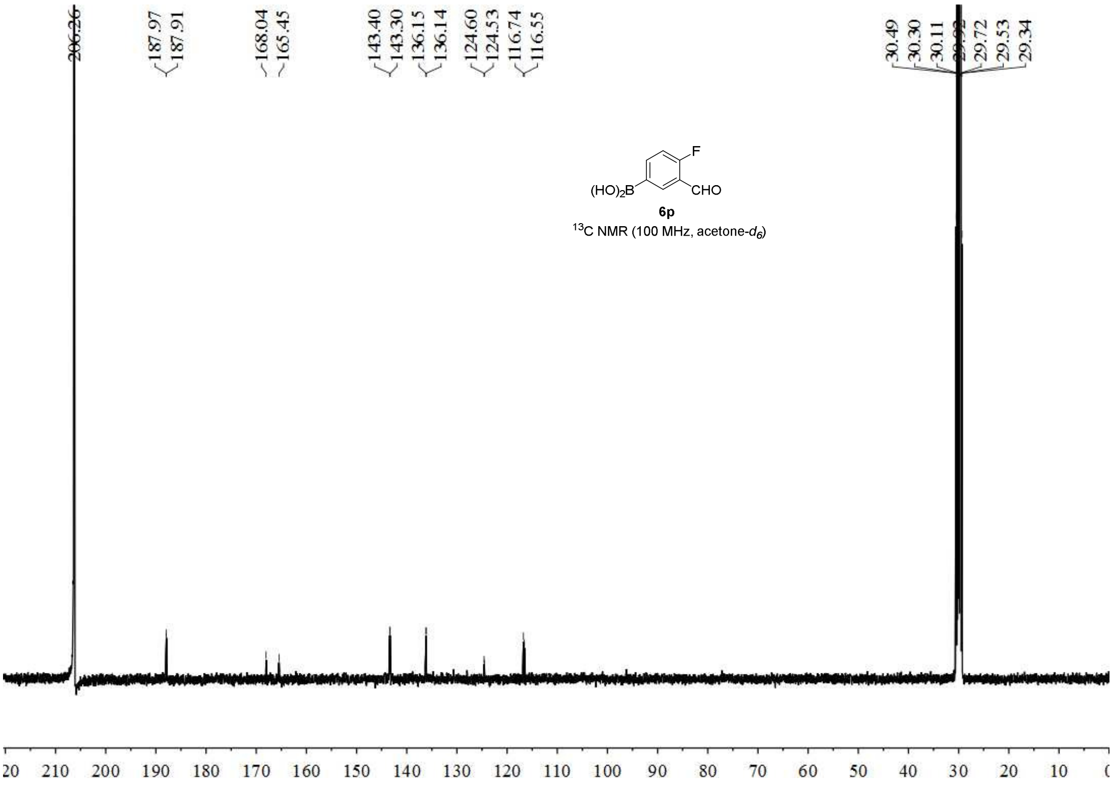

Supplementary Figure 92. ^{13}C NMR (100 MHz, acetone-d_6) of compound **6p**.

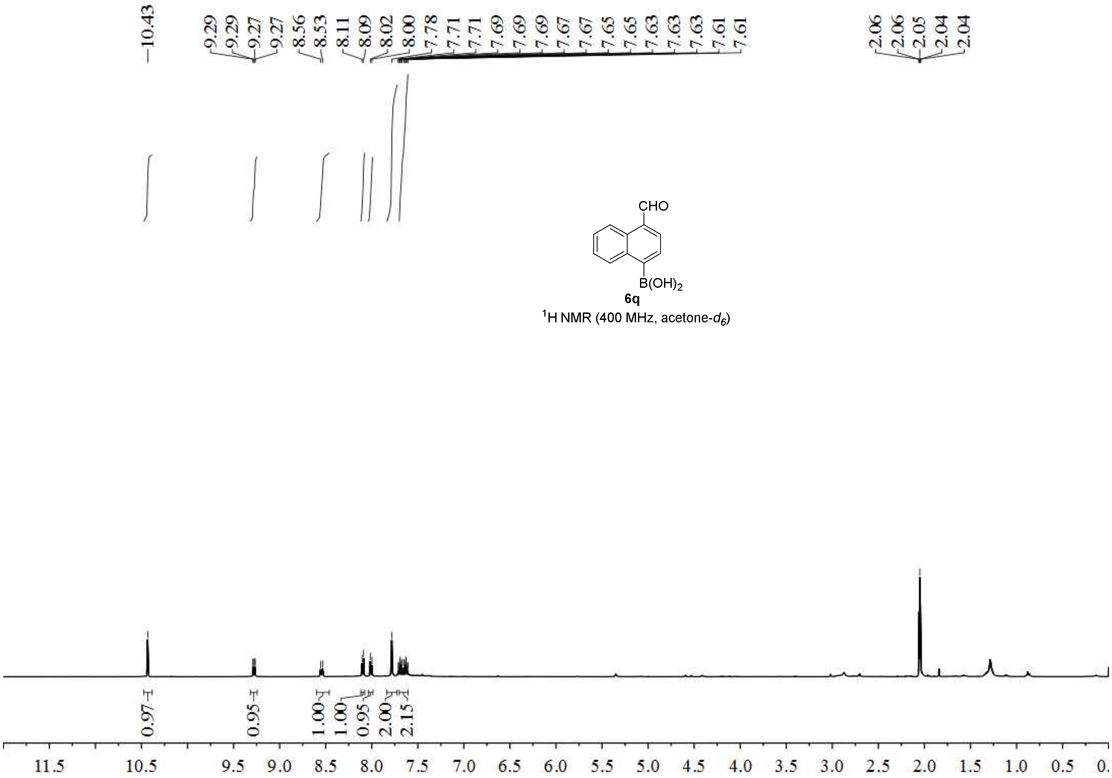

Supplementary Figure 93. ^{1}H NMR (400 MHz, acetone-d_6) of compound **6q**.

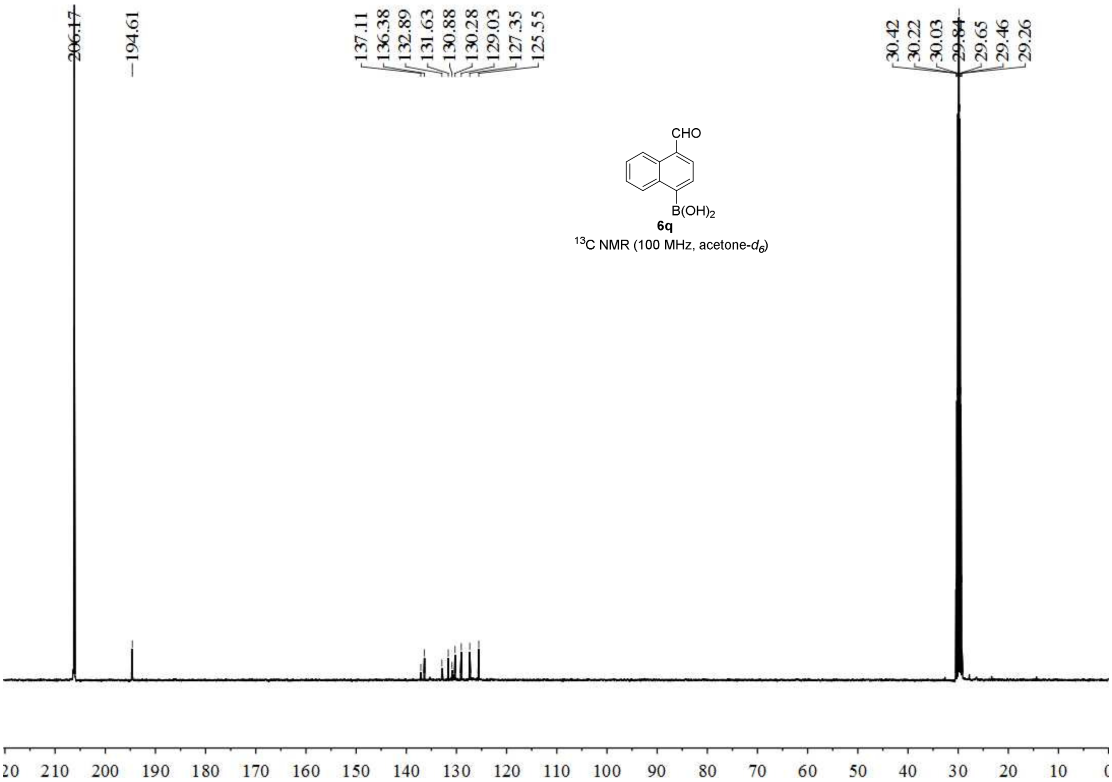

Supplementary Figure 94. ^{13}C NMR (100 MHz, acetone-d_6) of compound **6q**.

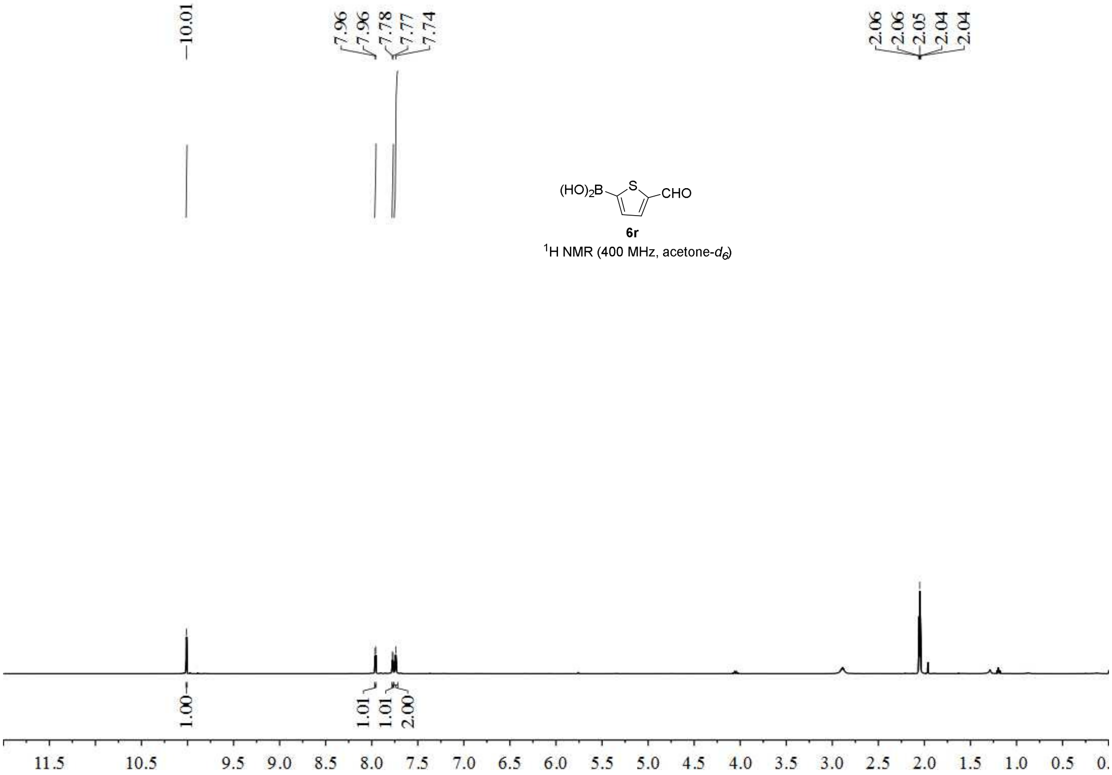

Supplementary Figure 95. ^{1}H NMR (400 MHz, acetone-d_6) of compound **6r**.

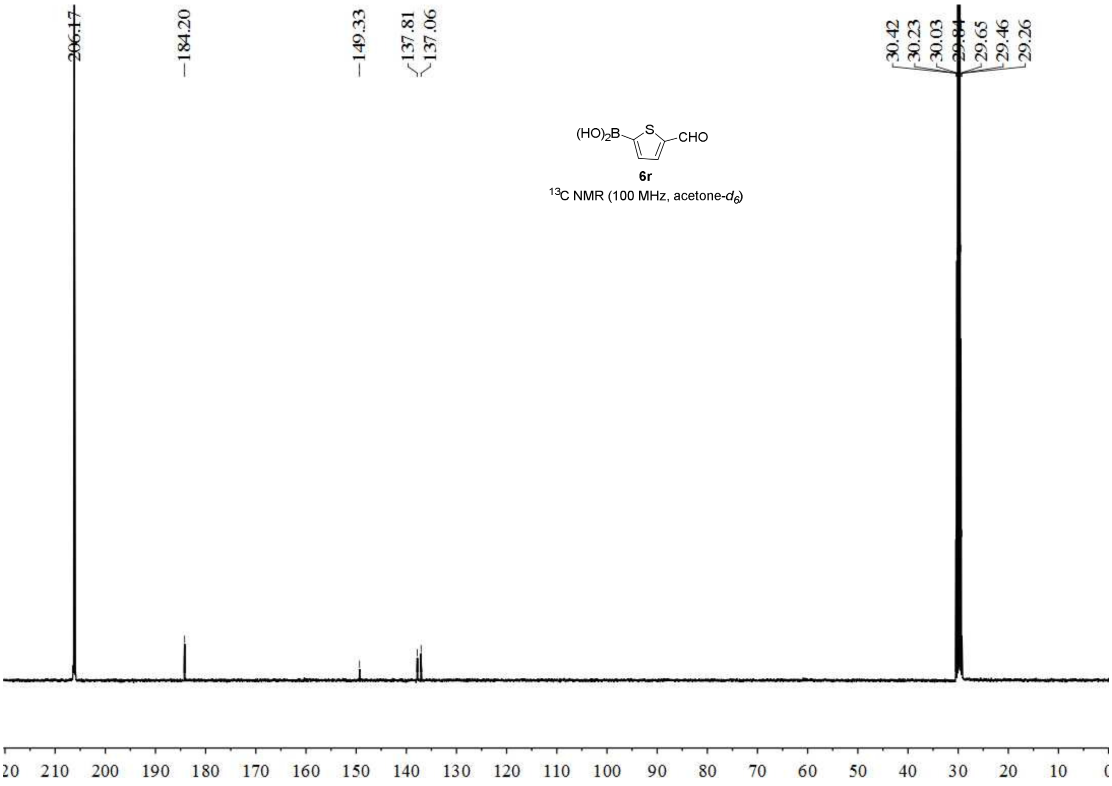

Supplementary Figure 96. ^{13}C NMR (100 MHz, acetone-d_6) of compound **6r**.

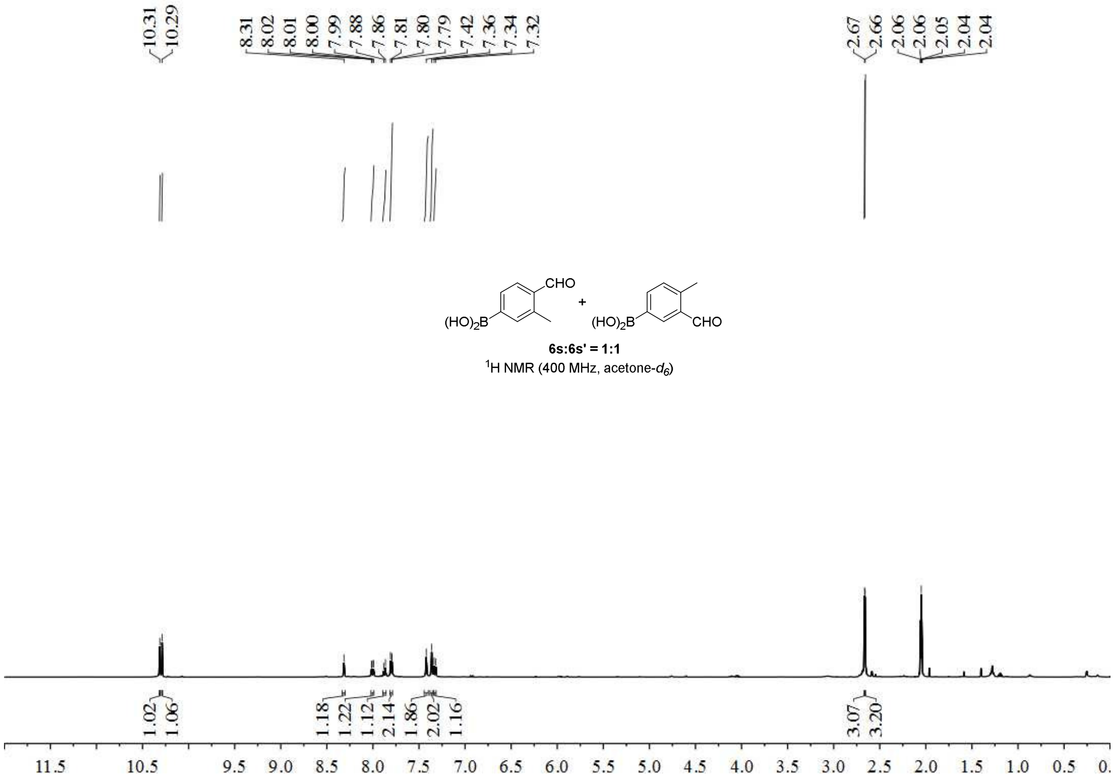

Supplementary Figure 97. ^{1}H NMR (400 MHz, acetone-d_6) of compound **6s**.

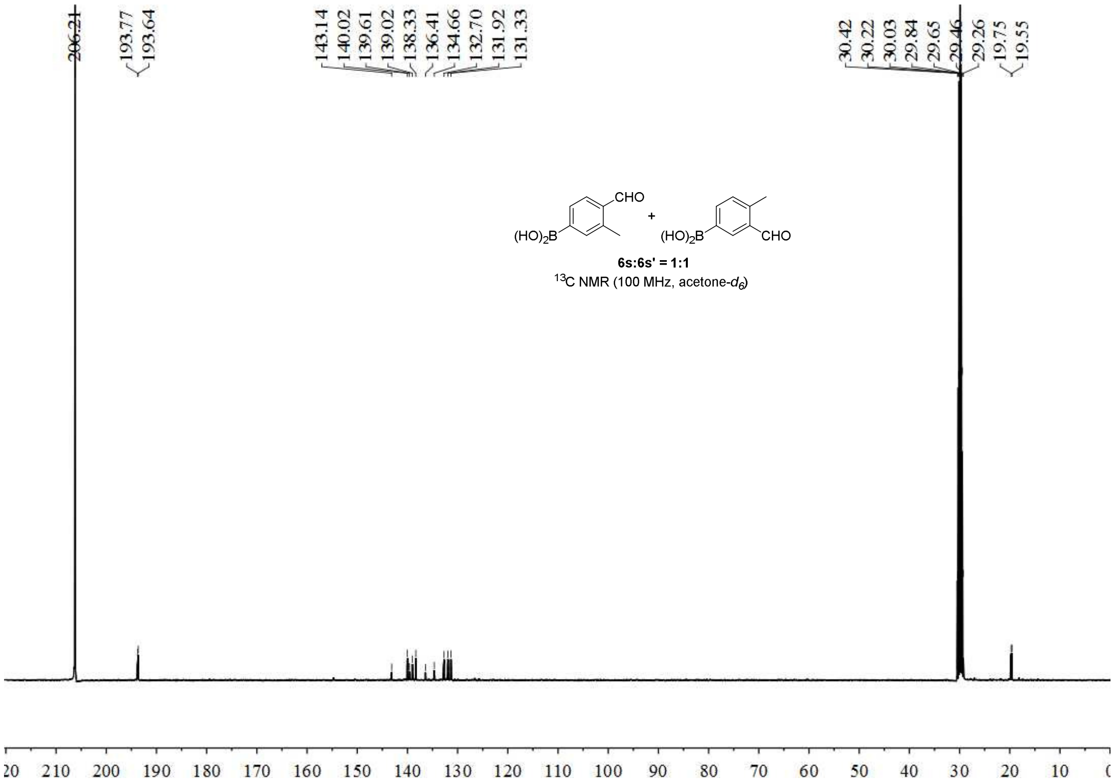

Supplementary Figure 98. ^{13}C NMR (100 MHz, acetone-d_6) of compound **6s**.

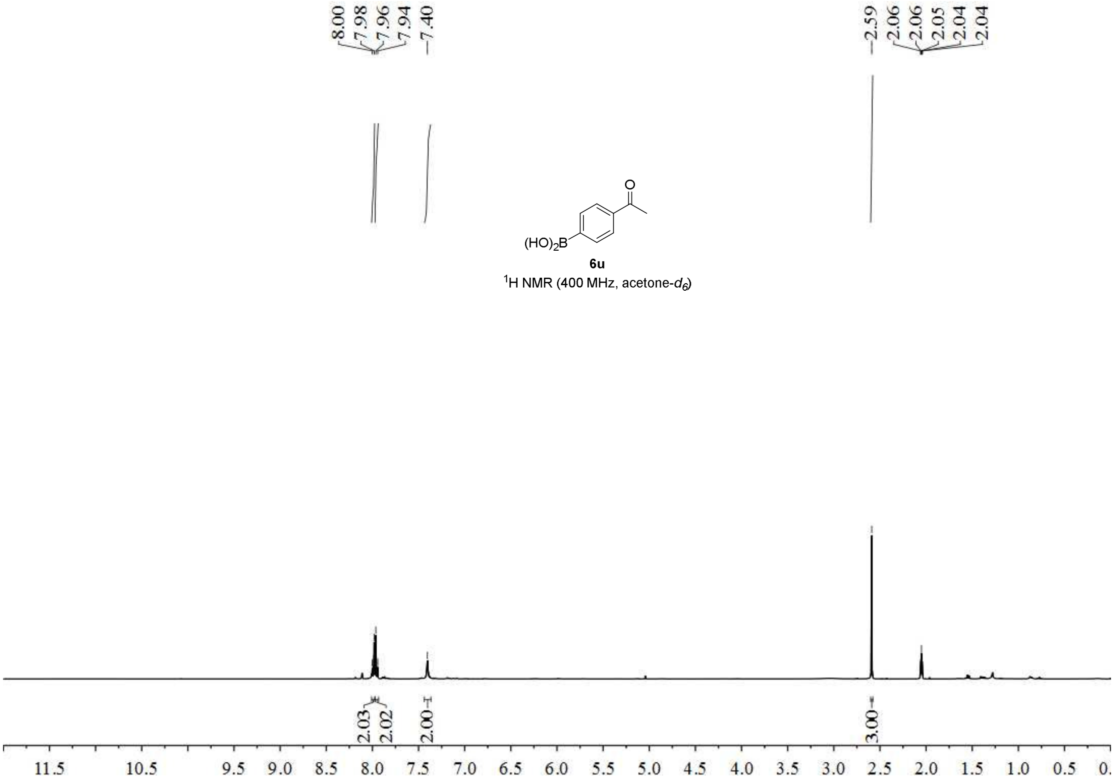

Supplementary Figure 99. ^{1}H NMR (400 MHz, acetone-d_6) of compound **6u**.

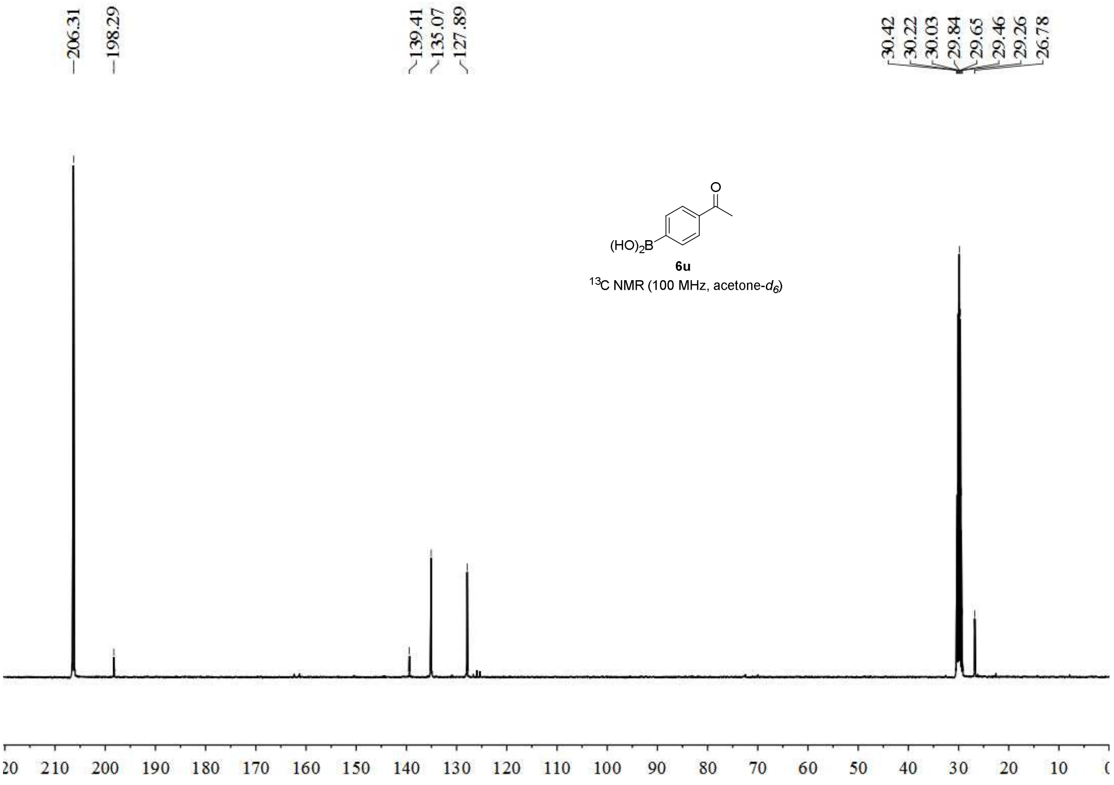

Supplementary Figure 100. ^{13}C NMR (100 MHz, acetone-d_6) of compound **6u**.

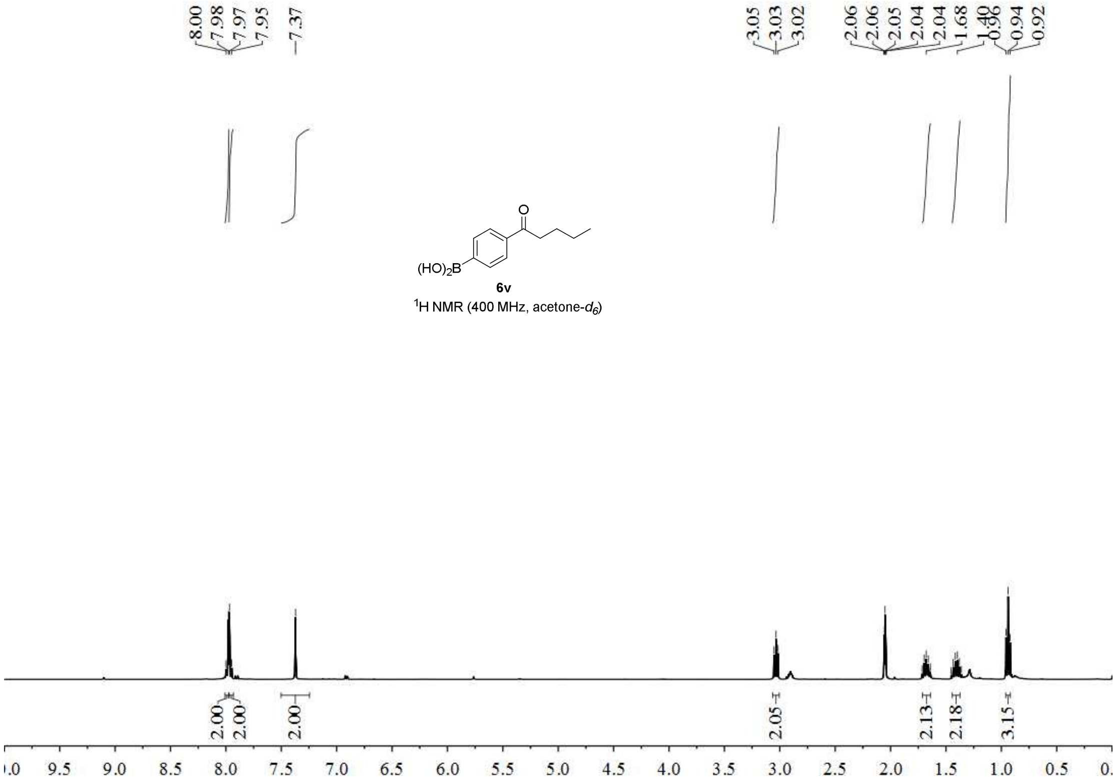

Supplementary Figure 101. ^{1}H NMR (400 MHz, acetone-d_6) of compound **6v**.

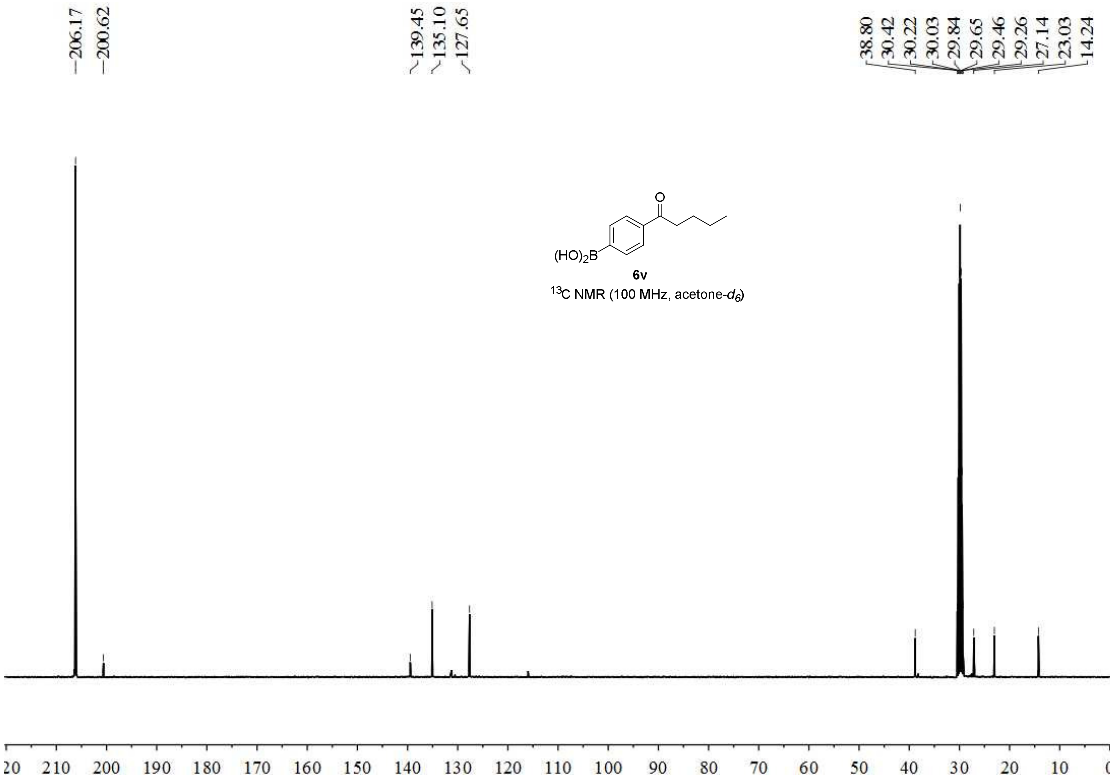

Supplementary Figure 102. ^{13}C NMR (100 MHz, acetone-d_6) of compound **6v**.

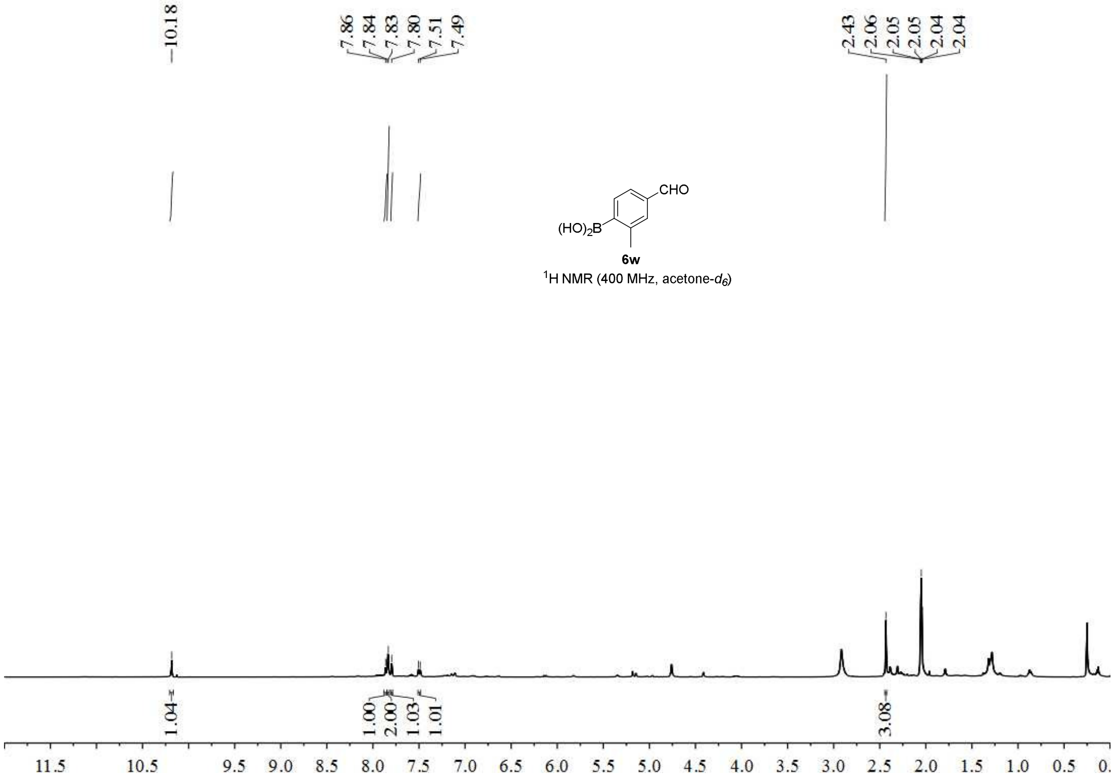

Supplementary Figure 103. ^{1}H NMR (400 MHz, acetone-d_6) of compound **6w**.

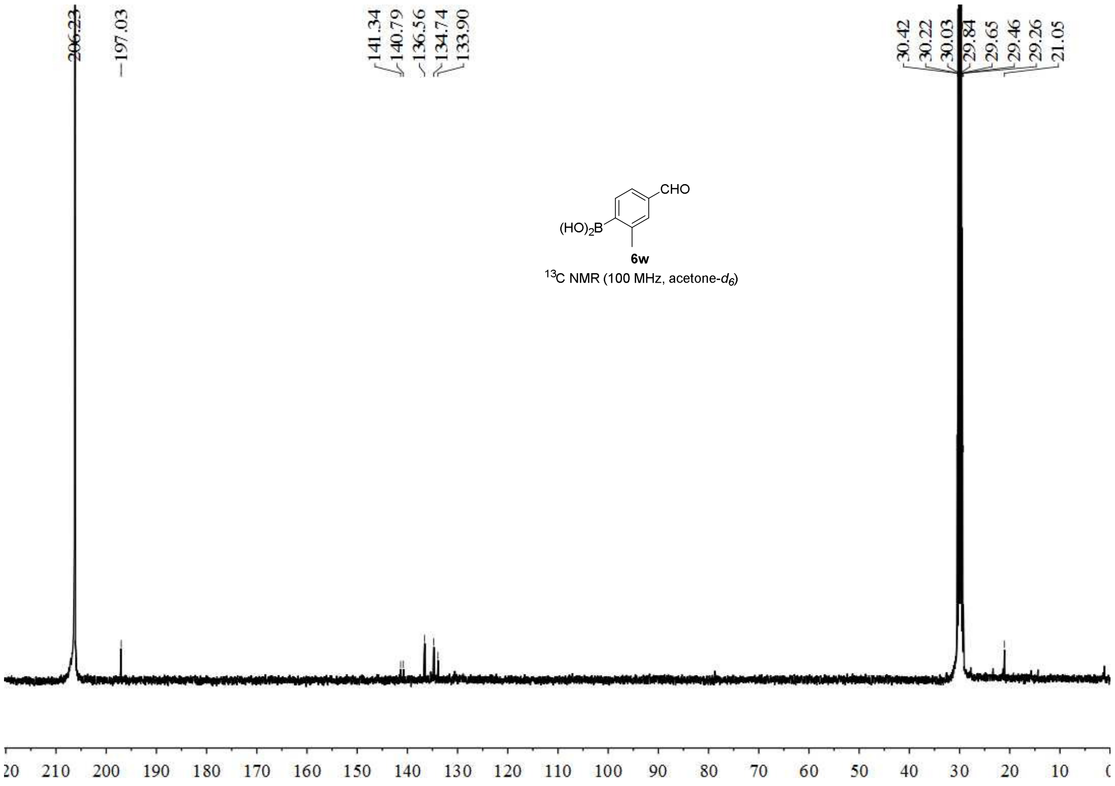

Supplementary Figure 104. ^{13}C NMR (100 MHz, acetone-d_6) of compound **6w**.

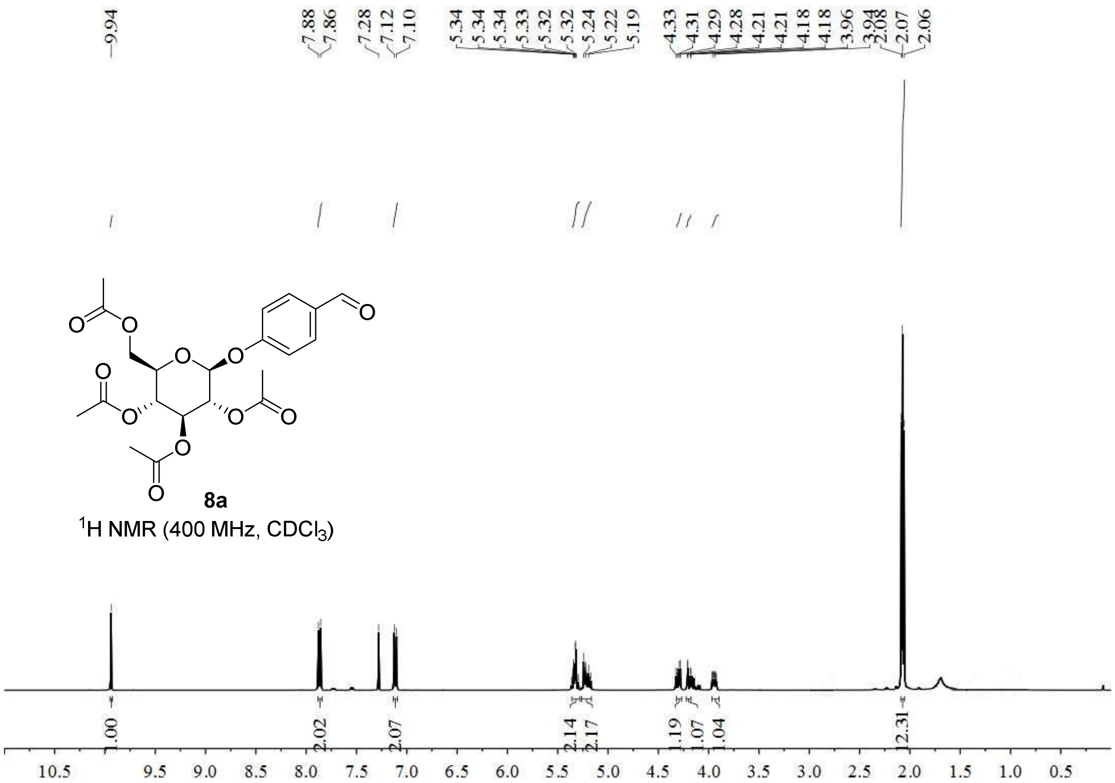

Supplementary Figure 105. ^{1}H NMR (400 MHz, $CDCl_3$) of compound **8a**.

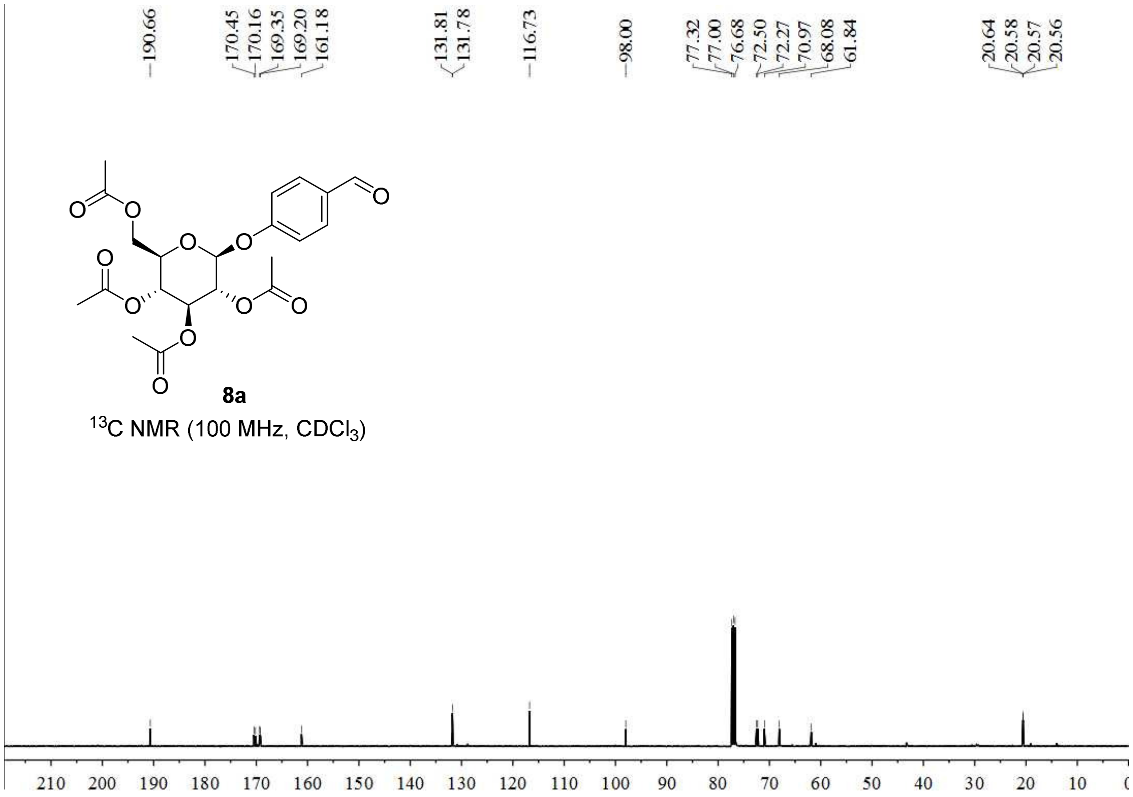

Supplementary Figure 106. ^{13}C NMR (100 MHz, $CDCl_3$) of compound **8a**.

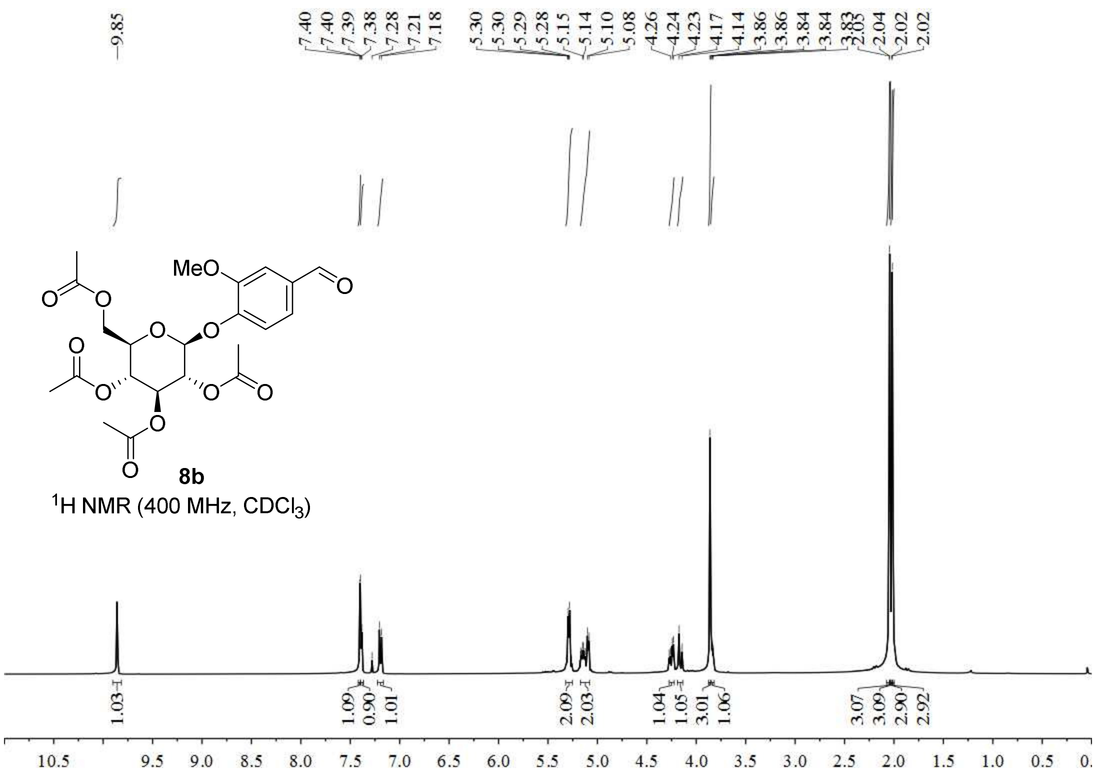

Supplementary Figure 107. ^{1}H NMR (400 MHz, CDCl$_3$) of compound **8b**.

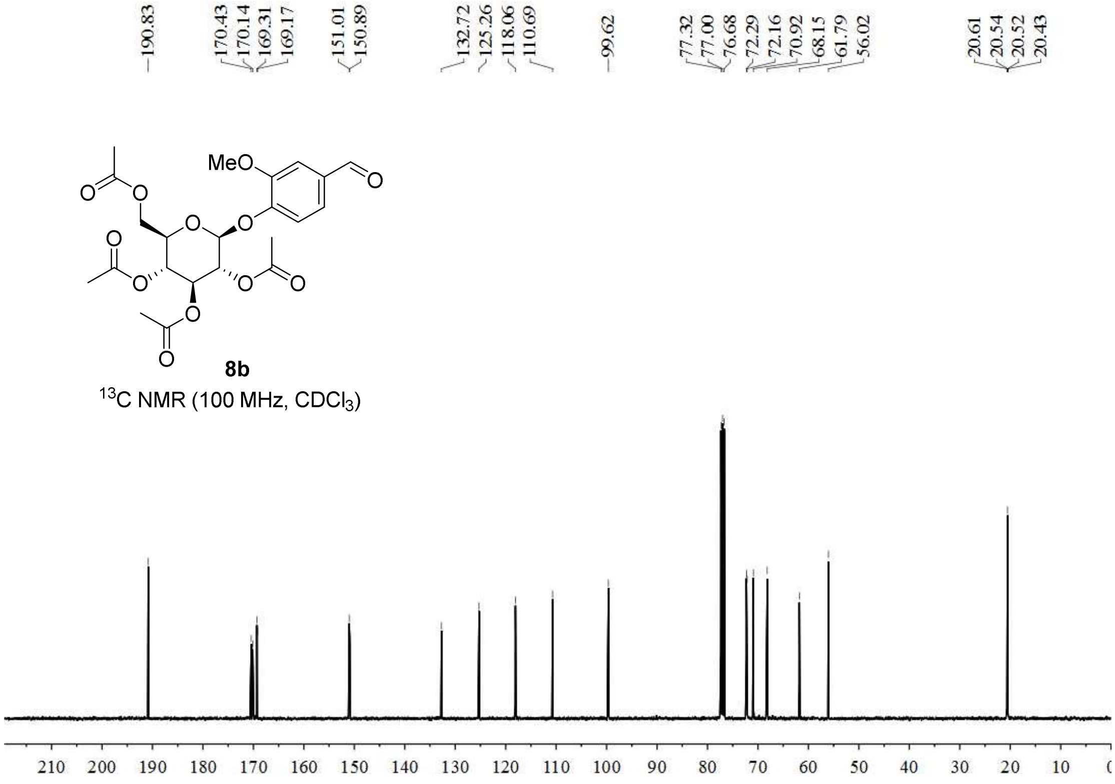

Supplementary Figure 108. ^{13}C NMR (100 MHz, CDCl$_3$) of compound **8b**.

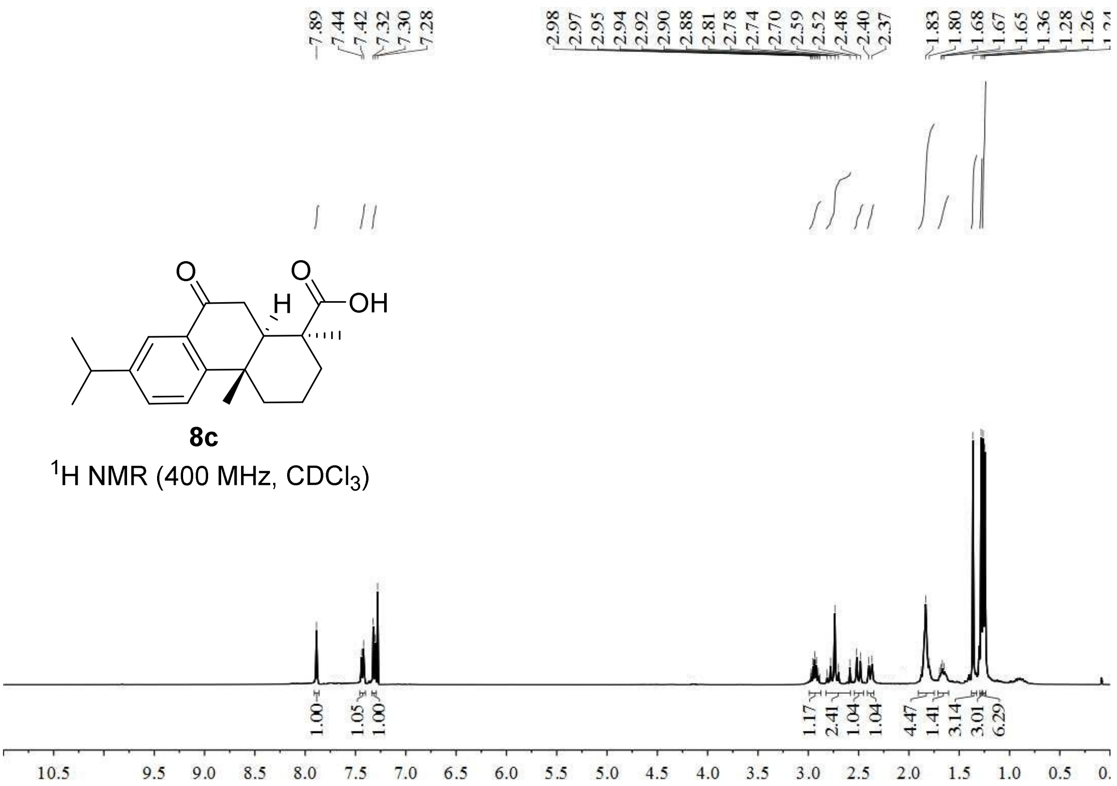

Supplementary Figure 109. ^{1}H NMR (400 MHz, $CDCl_3$) of compound **8c**.

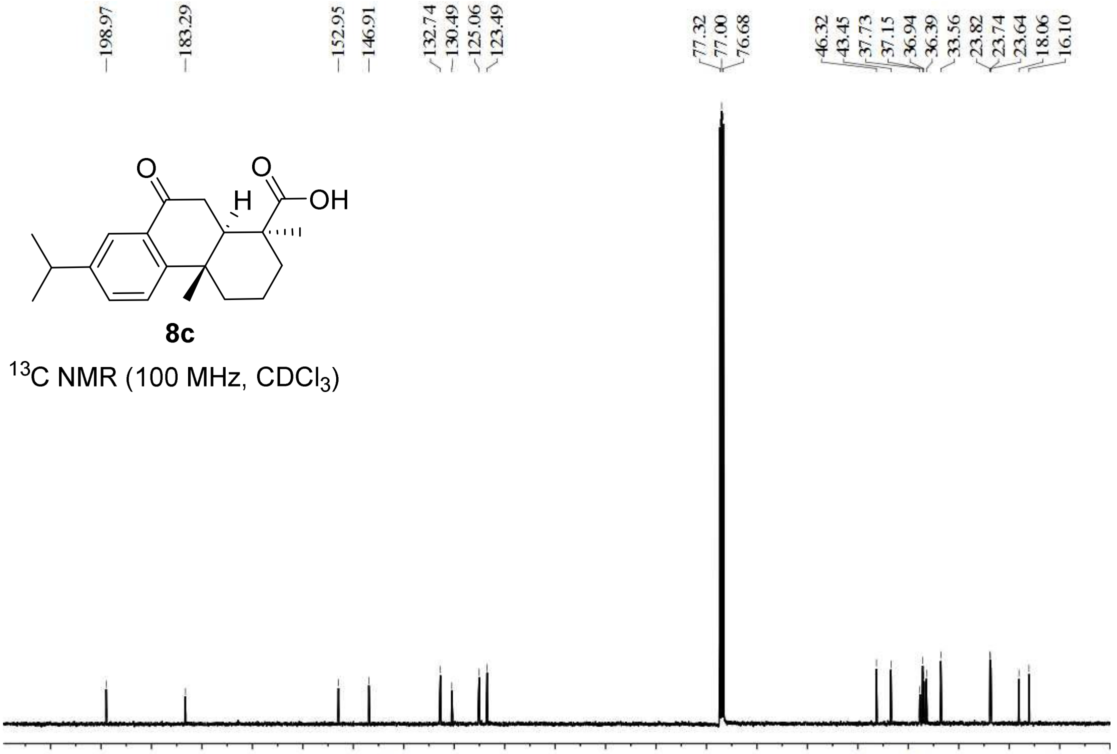

Supplementary Figure 110. ^{13}C NMR (100 MHz, $CDCl_3$) of compound **8c**.

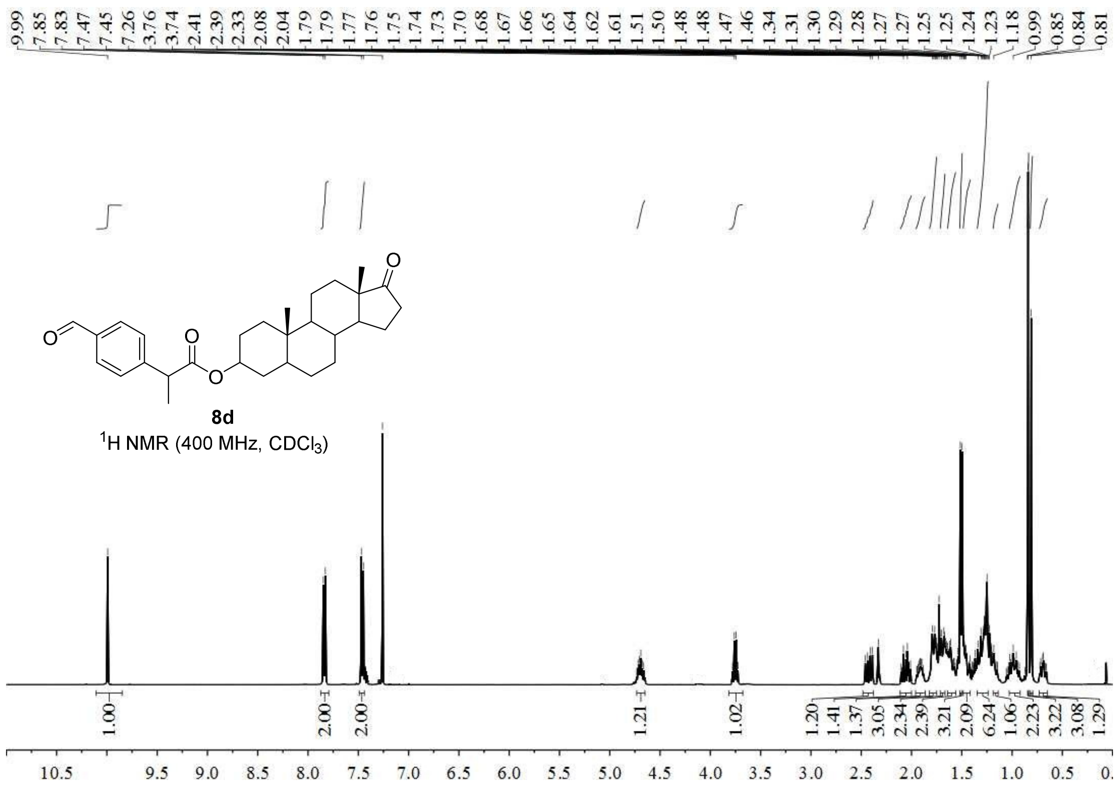

Supplementary Figure 111. ^{1}H NMR (400 MHz, $CDCl_3$) of compound **8d**.

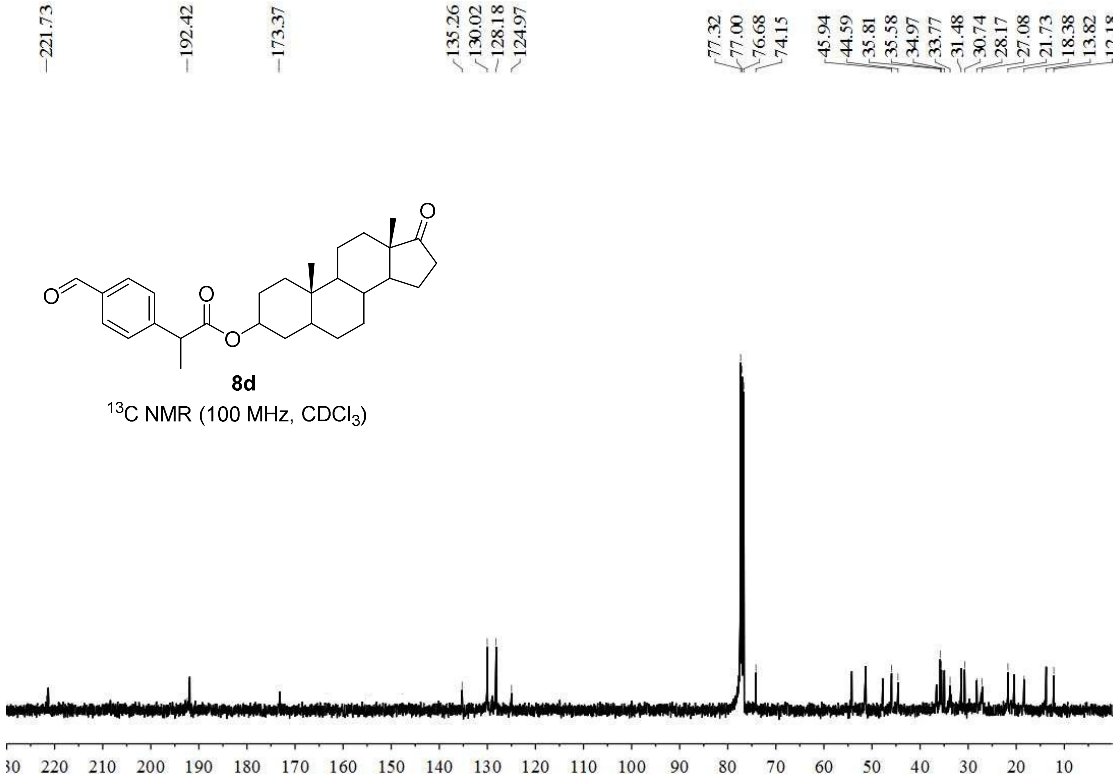

Supplementary Figure 112. ^{13}C NMR (100 MHz, $CDCl_3$) of compound **8d**.

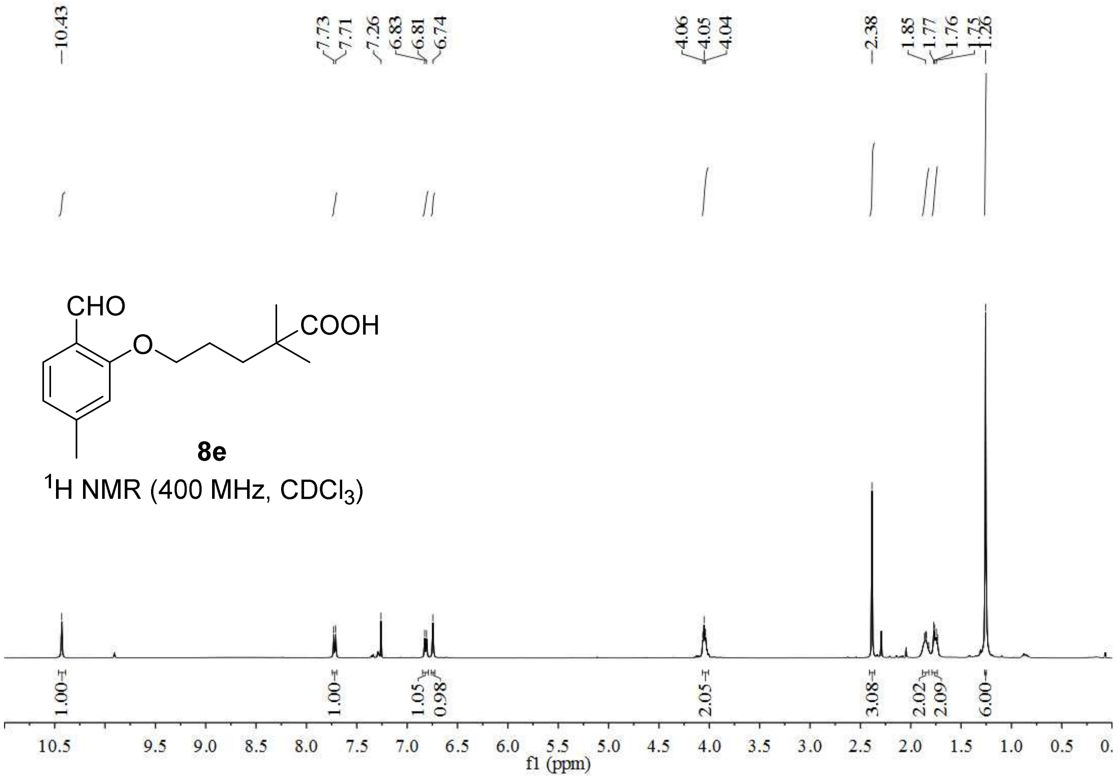

Supplementary Figure 113. ^{1}H NMR (400 MHz, $CDCl_3$) of compound **8e**.

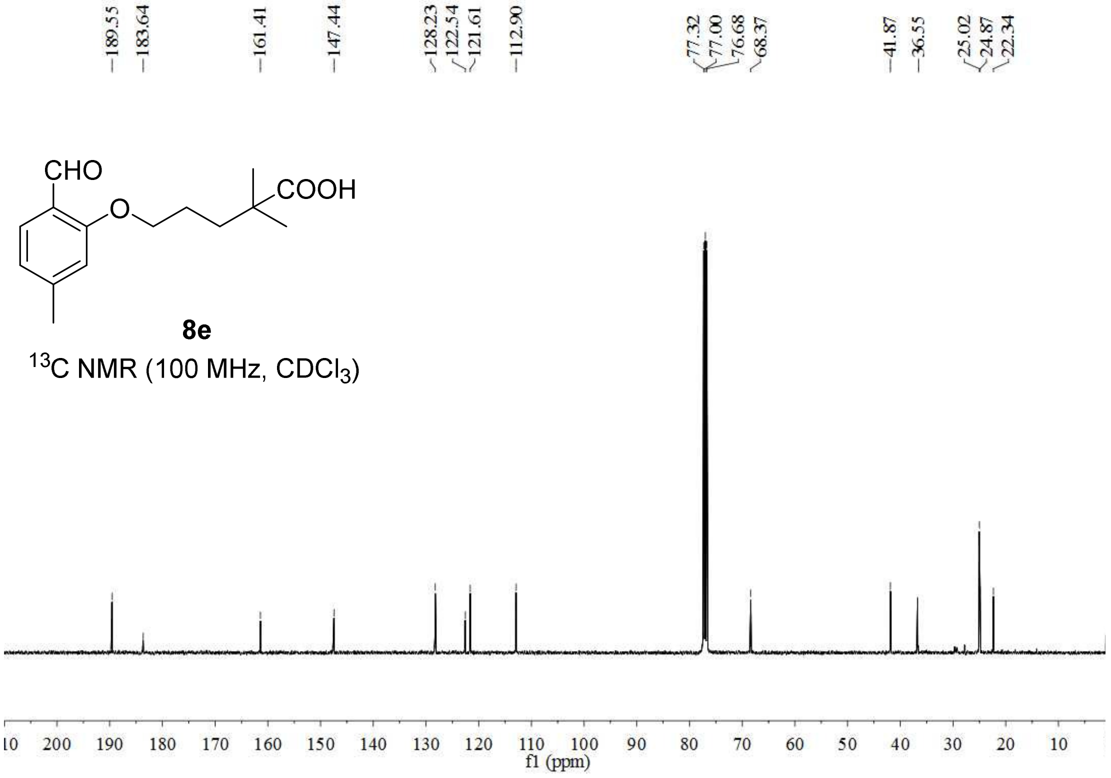

Supplementary Figure 114. ^{13}C NMR (100 MHz, $CDCl_3$) of compound **8e**

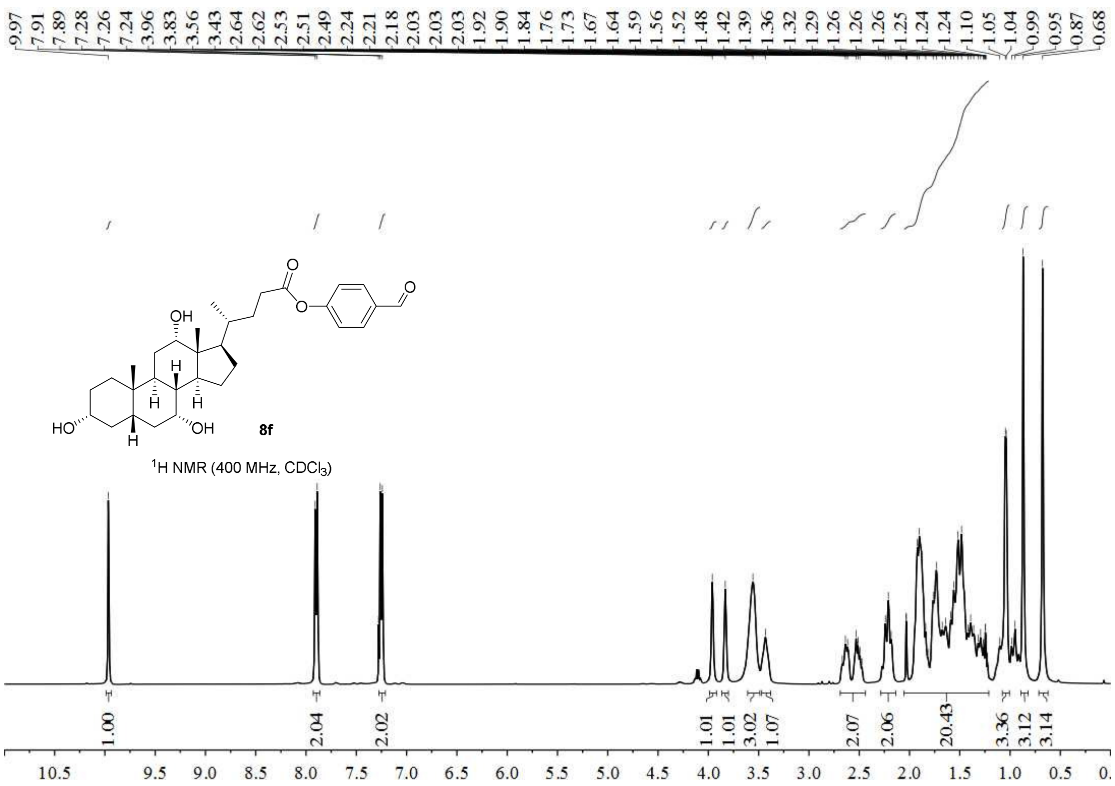

Supplementary Figure 115. ^{1}H NMR (400 MHz, $CDCl_3$) of compound **8f**.

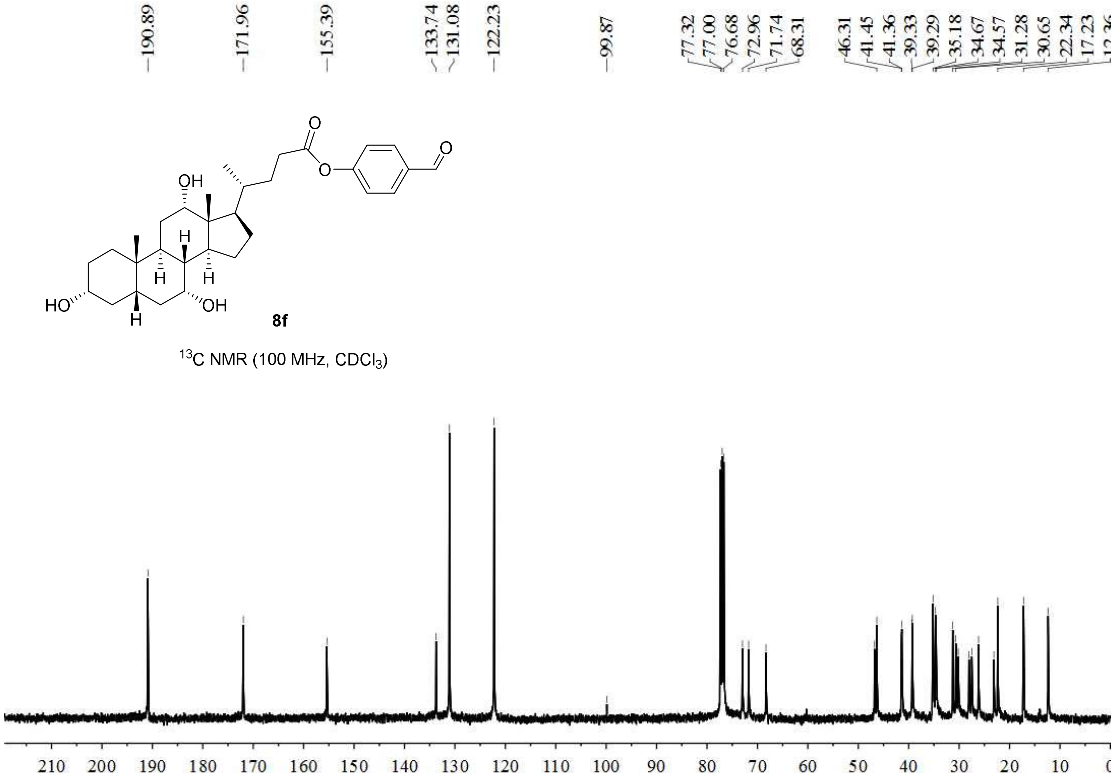

Supplementary Figure 116. ^{13}C NMR (100 MHz, $CDCl_3$) of compound **8f**.

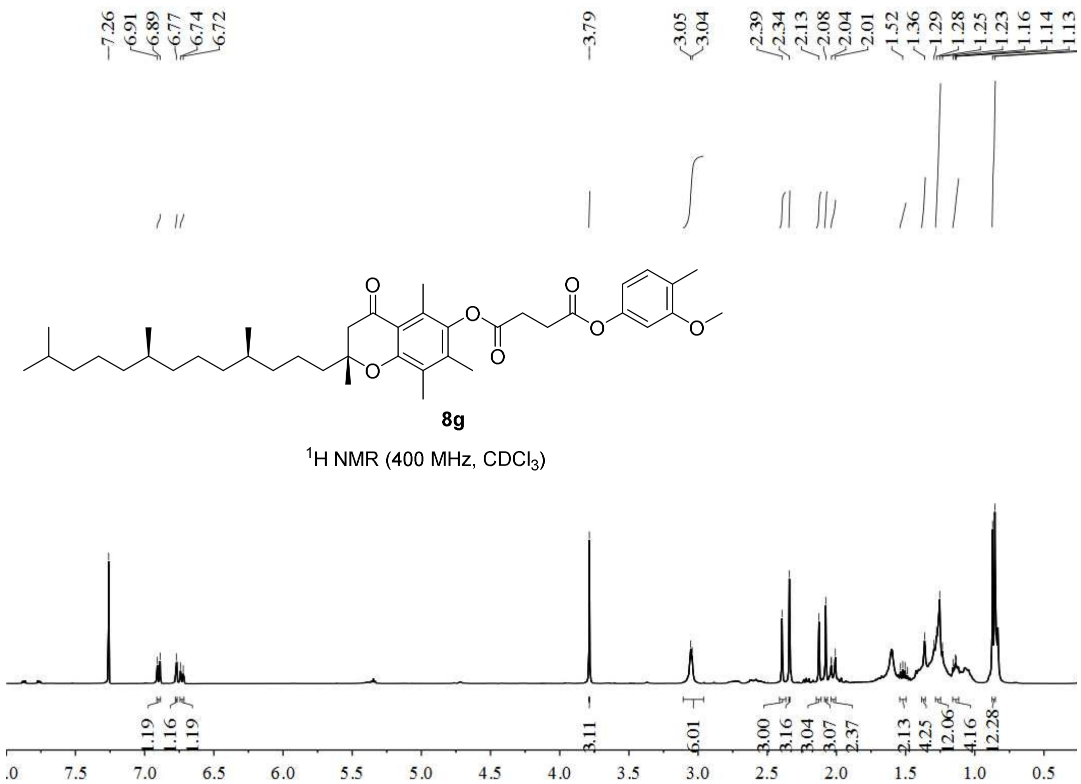

Supplementary Figure 117. ^{1}H NMR (400 MHz, $CDCl_3$) of compound **8g**.

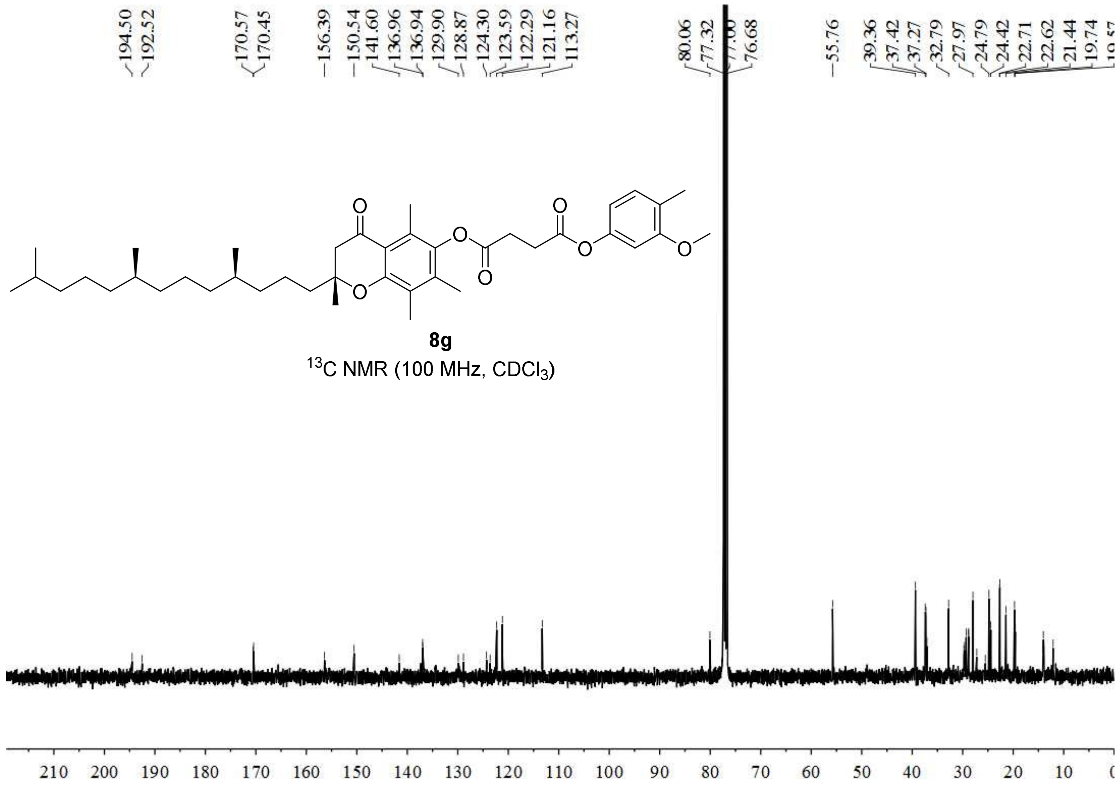

Supplementary Figure 118. ^{13}C NMR (100 MHz, $CDCl_3$) of compound **8g**.

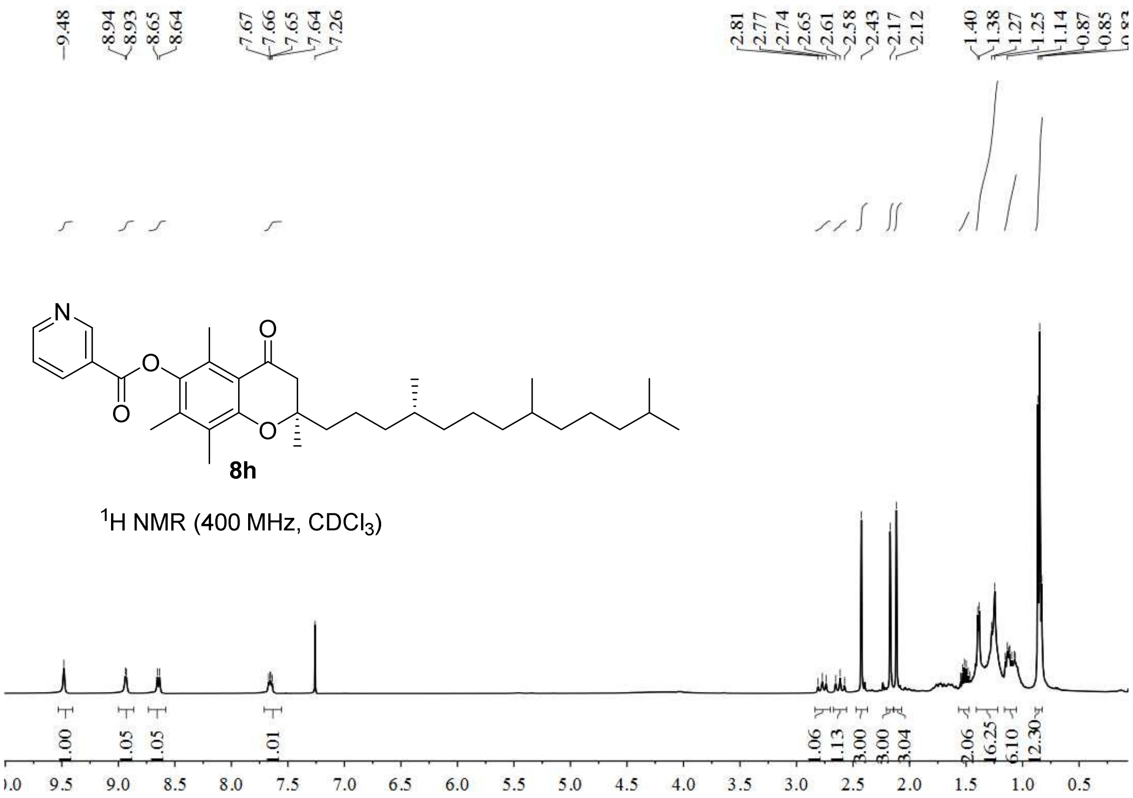

Supplementary Figure 119. ^{1}H NMR (400 MHz, CDCl$_3$) of compound **8h**.

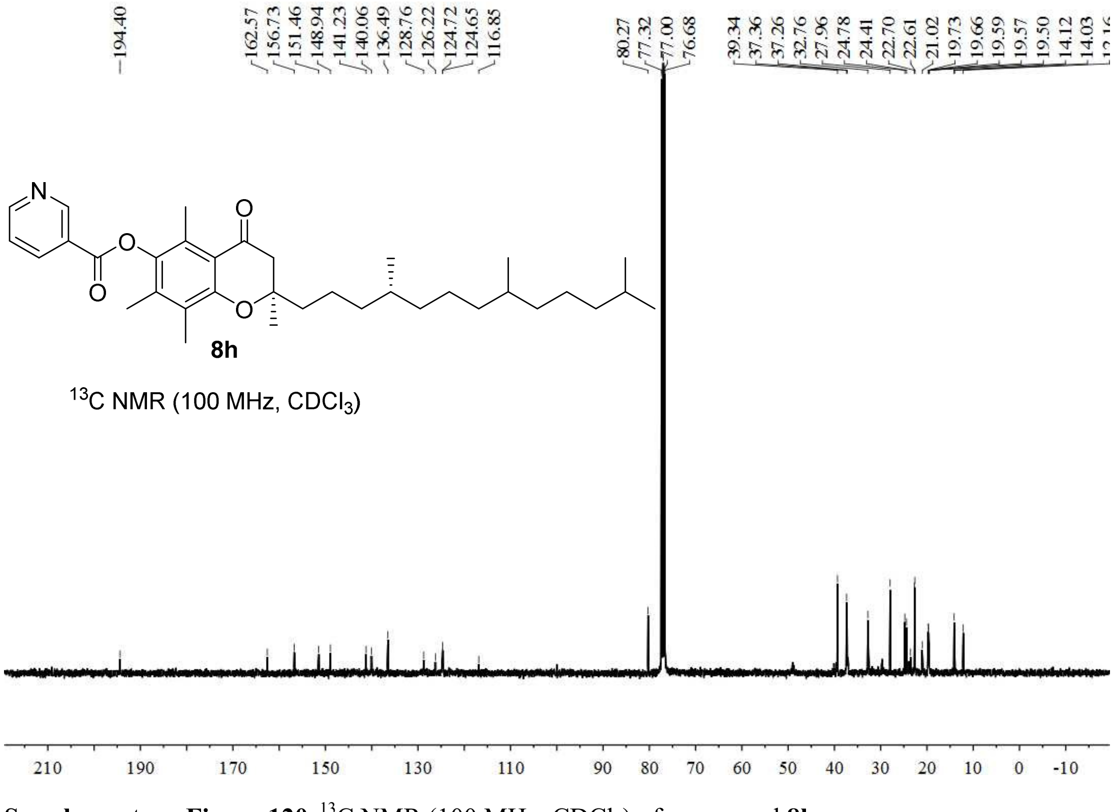

Supplementary Figure 120. ^{13}C NMR (100 MHz, CDCl$_3$) of compound **8h**.

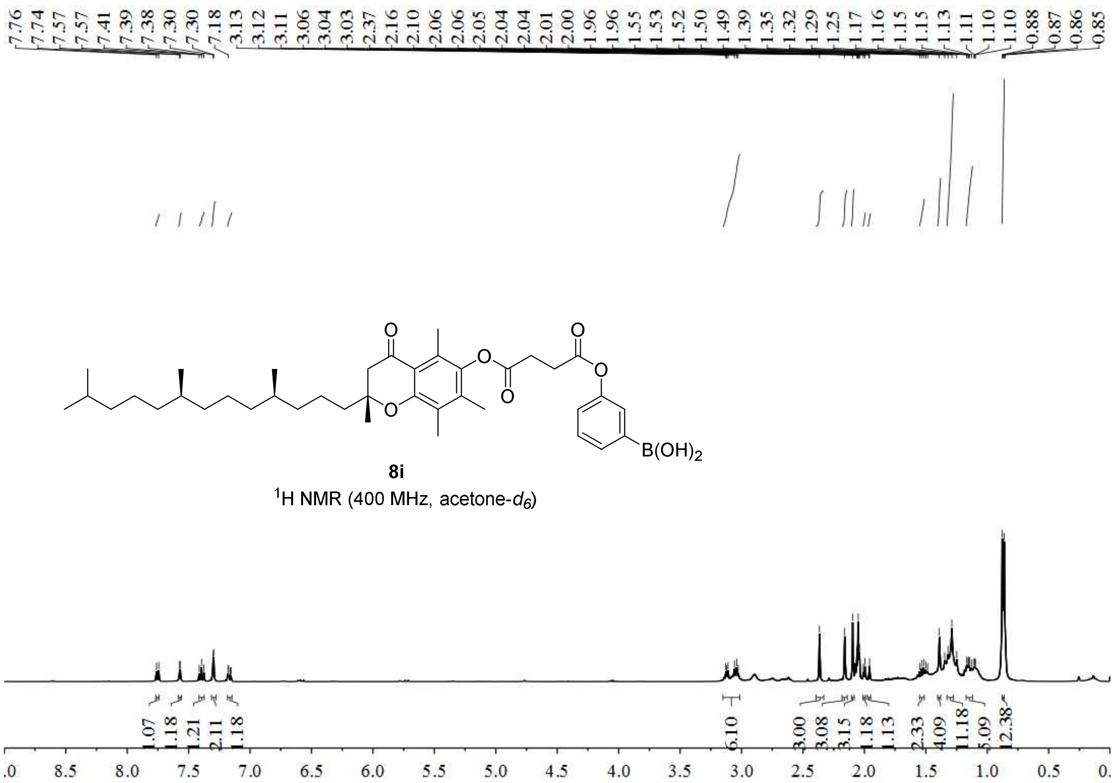

Supplementary Figure 121. ^{1}H NMR (400 MHz, acetone-d_6) of compound **8i**.

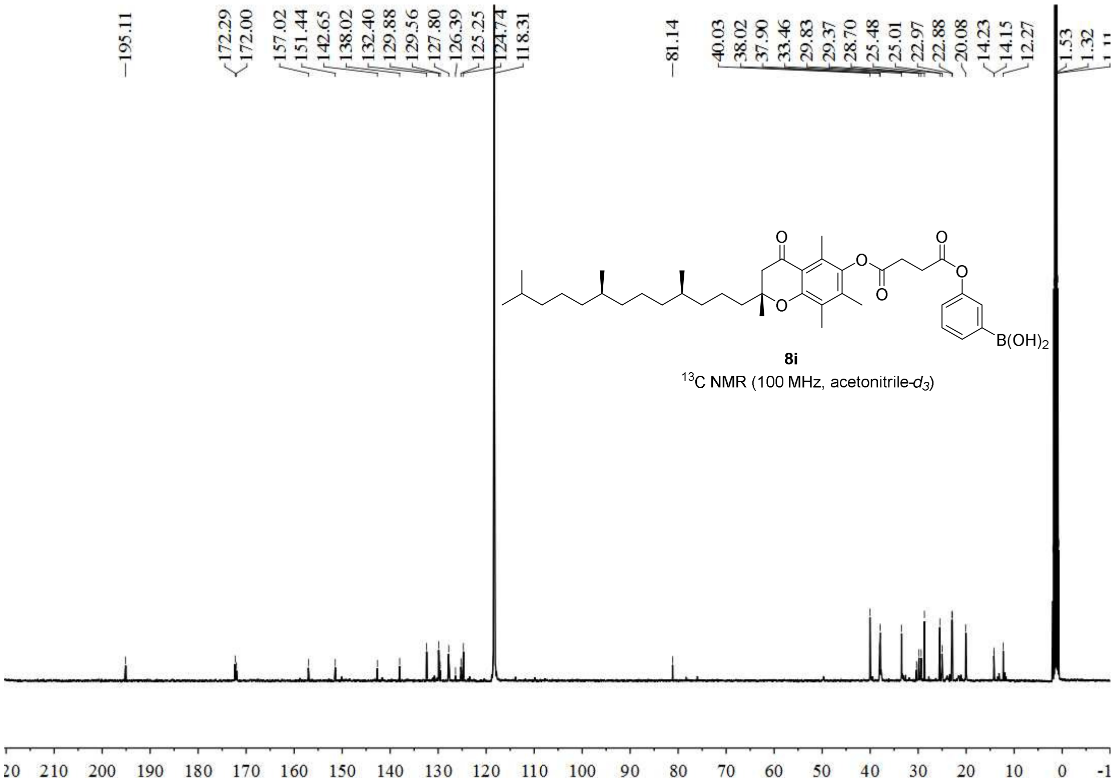

Supplementary Figure 122. ^{13}C NMR (100 MHz, acetonitrile-d_3) of compound **8i**.

Supplementary References

1. Fujiya, A., Kariya, A., Nobuta, T., Tada, N., Miura, T. & Itoh, A. Photooxidative cleavage of aromatic alkenes into aldehydes using catalytic iodine and molecular oxygen under visible light irradiation. *Synlett* **25**, 884–888 (2014).
2. Cui, L. Q., Liu, K. & Zhang, C. Effective oxidation of benzylic and alkane C–H bonds catalyzed by sodium *o*-iodobenzenesulfonatewith Oxone as a terminal oxidant under phase-transfer conditions. *Org. Biomol. Chem.* **9,** 2258–2265 (2011).
3. Lindgren, A. E. G., Öberg, C. T., Hillgren, J. M. & Elofsson, M. Total synthesis of the resveratrol oligomers (±)-Ampelopsin B and (±)-ε-Viniferin. *Eur. J. Org. Chem.* **2016**, 426–429 (2016).
4. Li, L., Ge, J. Y., Wu, H., Xu, Q.-H. & Yao, S. Q. Organelle-specific detection of phosphatase activities with two-photon fluorogenic probes in cells and tissues. *J. Am. Chem. Soc.* **134,** 12157–12167 (2012).
5. Mi, C., Li, L., Meng, X.-G., Yang, R.-Q. & Liao, X.-H. Highly selective oxidation of unsaturated hydrocarbons to carbonyl compounds by two-phase catalysis. *Tetrahedron* **72**, 6705–6710 (2016).
6. Yang, H. J., Li, Y., Jiang, M., Wang, J. M. & Fu, H. General copper-catalyzed transformations of functional groups from arylboronic acids in water. *Chem. Eur. J.* **17**, 5652–5660 (2011).
7. Quadri, S. A. I., Das, T. C., Malik, M. S., Seddigi, Z. S. & Farooqui, M. Efficient ferric citrate-catalyzed synthesis of novel dihydropyrimidin-(1H)-ones sulfonamide conjugates and their evaluation as potential antimicrobials. *ChemistrySelect* **2**, 6818–6822 (2017).
8. Payra, S., Saha, A. & Banerjee, S. Nano-$NiFe_2O_4$ as an efficient catalyst for regio- and chemoselective transfer hydrogenation of olefins/alkynes and dehydrogenation of alcohols under Pd-/Ru-free conditions. *RSC Adv.* **6**, 52495–52499 (2016).
9. Yeh, C.-H., Chen, W.-C., Gandeepan, P., Hong, Y.-C., Shih, C.-H. & Cheng, C.-H. Rh^{III}-catalyzed dual directing group assisted sterically hindered C–H bond activation: a unique route to *meta* and *ortho* substituted benzofurans. *Org. Biomol. Chem.* **12**, 9105–9108 (2014).
10. Imai, S. & Togo, H. Synthetic utility of iodic acid in the oxidation of benzylic alcohols to aromatic aldehydes and ketones. *Tetrahedron* **72**, 6948–6954 (2016).
11. Sheykhan, M., Moafi, H. F. & Abbasina, M. Novel access to carbonyl and acetylated compounds: the role of the *tetra-n*-butylammonium bromide/sodium nitrite catalyst. *RSC Adv.* **6**, 51347–51355 (2016).
12. Giovani, S., Singh, R. & Fasan, R. Efficient conversion of primary azides to aldehydes catalyzed by active site variants of myoglobin. *Chem. Sci.* 7, 234–239 (2016).
13. Huang, H., Yu, C. G., Li, X. M., Zhang, Y. Q., Zhang, Y. T., Chen, X. B., Mariano, P. S., Xie, H. X. & Wang, W. Synthesis of aldehydes by organocatalytic formylation reactions of boronic acids with glyoxylic acid. *Angew. Chem. Int. Ed.* **56**, 8201–8205 (2017).
14. Guan, M. Y., Wang, C., Zhang, J. Y. & Zhao, Y. S. Practical organic solvent-free $Cu(OAc)_2$ /DMAP/TEMPO-catalyzed aldehyde and imine formation from alcohols under air atmosphere. *RSC Adv.* **4**, 48777–48782 (2014).
15. Maras, N., Polanc, S. & Kocevar, M. Microwave-assisted methylation of phenols with tetramethylammonium chloride in the presence of K_2CO_3 or Cs_2CO_3. *Tetrahedron* **64**, 11618–11624 (2008).
16. Kawashima, H., Kawabata, K. & Goto, H. Synthesis and double doping behavior of a poly(*p*-phenylenevinylene)s bearing conjugated side chains. *J. Polym. Sci. Pol.Chem.* **50**, 1530–1538 (2012).
17. Zhao, Y., Jiang, K. J., Xu, W. & Zhu, D. B. Macrocyclic triphenylamine based organic dyes for efficient dye-sensitized solar Cells. *Tetrahedron* **68**. 9113–9118 (2012).
18. Wang, T., Bing, G., Zhang, X., Qin, Z., Yu, H., Qin, X., Dai, H., Miao, W., Wu, S. & Fang, J. Synthesis and herbicidal activities of 2-cyano-3-benzylaminoacrylates containing thiazole moiety. *Bioorg. Med.Chem. Lett.* **20**, 3348–3351 (2010).

19. Olivo, G., Giosia, S., Barbieri, A., Lanzalunga, O. & Stefano, S. D. Alcohol oxidation with H_2O_2 catalyzed by a cheap and promptly available imine based iron complex. *Org. Biomol. Chem.* **14**, 10630–10635 (2016).

20. Yu, Y. & Liebeskind, L. S. Copper-Mediated, palladium-catalyzed coupling of thiol esters with aliphatic organoboron reagents. *J. Org. Chem.* **69**, 3554–3557 (2004).

21. Li, D. Y., Chen, H. J. & Liu, P. N. Tunable cascade reactions of alkynols with alkynes under combined $Sc(OTf)_3$ and rhodium catalysis. *Angew. Chem. Int. Ed.* **55**, 373–377 (2016).

22. Yu, A. J., Shen, L., Cui, X. L., Peng, D. P. & Wu, Y. J. Palladacycle-catalyzed cross-coupling reactions of arylboronic acids with carboxylic anhydrides or acyl chlorides. *Tetrahedron* **68**, 2283–2288 (2012).

23. Huang, P.-Q., Wang, Y., Xiao, K.-J. & Huang, Y. H. A general method for the direct transformation of common tertiary amides into ketones and amines by addition of Grignard reagents. *Tetrahedron* **71**, 4248–4254 (2015).

24. Yang, Y. W. & Ma, H. X. Room-temperature direct benzylic oxidation catalyzed by cobalt(II) Perchlorate. *Tetrahedron Lett.* **57**, 5278–5280 (2016).

25. Liu, H. X., Dang, Y. Q., Yuan, Y. F., Xu, Z. F., Qiu, S. X. & Tan, H. B. Diacyl disulfide: A reagent for chemoselective acylation of phenols enabled by 4‑(*N,N*‑dimethylamino)pyridine catalysis. *Org. Lett.* **18**, 5584–5587 (2016).

26. Molander, G. A., Cavalcanti, L. N., Canturk, B., Pan, P. -S. & Kennedy, L. E. Efficient hydrolysis of organotrifluoroborates via silica gel and water. *J. Org. Chem*. **74**, 7364-7369 (2009).

27. Jalily, P. H., Hadfield, J. A., Hirst, N. & Rossington, S. B. Novel cyanocombretastatins as potent tubulin polymerisation inhibitors. *Bioorg. Med. Chem. Lett.* **22**, 6731-6734 (2012).

28. Yeste, S. L., Powell, M. E., Bull, S. D. & James, T. D. Simple chiral derivatization protocols for ^{1}H NMR and ^{19}F NMR spectroscopic analysis of the enantiopurity of chiral diols. *J. Org. Chem.* **74**, 427-430 (2009).

29. Fujita, K., Hatano, S., Kato, D. & Abe, J. Photochromismof a radical diffusion-inhibited hexaarylbiimidazole derivative with intense coloration and fast decoloration performance. *Org. Lett.* ***10***, 3105-3108 (2008).

30 Liu, B. B., Jin, F. L., Wang, T. J., Yuan, X. R. & Han, W. Wacker-type oxidation using an iron catalyst and ambient air: Application to late-stage oxidation of complex molecules. *Angew. Chem. Int. Ed.* **56,** 12712–12717 (2017).

31. Rontani, J. F., Aubert, C. & Belt, S. T. EIMS fragmentation pathways and MRM quantification of 7α/β-hydroxy-dehydroabietic acid TMS derivatives. *J. Am. Soc. Mass Spectrom.* **26**, 1606–1616 (2015).

32. Gaster, E., Kozuch, S. & Pappo, D. Selective aerobic oxidation of methylarenes to benzaldehydes catalyzed by *N*-hydroxyphthalimide and cobalt(II) acetate in Hexafluoropropan-2-ol. *Angew. Chem. Int. Ed.* **56**, 5912–5915 (2017).

33 Yi, H., Tang, Z., Bian, C., Chen, H., Qi, X., Yue, X., Lan, Y., Lee, J. & Lei, A. Oxidation-induced C–H amination leads to a new avenue to build C–N bonds. *Chem. Commun.* **53**, 8984–8987 (2017).

34 Al-hunaiti, A., Räisänen, M. & Repo, T. From DNA to catalysis: a thymine-acetate ligated non-heme iron(III) catalyst for oxidative activation of aliphatic C–H bonds. *Chem. Commun.* **52**, 2043–2046 (2016).

35 Minisci, F. & Citterio, A. Electron-transfer processes: peroxydisulfate, a useful and versatile reagent in organic chemistry. *Acc. Chem. Res.* **16**, 27–32 (1983).

36 Zhang, M., Zhai, Y., Ru, S., Zang, D., Han, S., Yu, H. & Wei, Y. Highly practical and efficient preparation of aldehydes and ketones from aerobic oxidation of alcohols with an inorganic-ligand supported iodine catalyst. *Chem. Commun.* **54**, 10164–10167 (2018).
